# Amyotrophic lateral sclerosis transcriptomics reveals immunological effects of low-dose interleukin-2

**DOI:** 10.1093/braincomms/fcab141

**Published:** 2021-06-29

**Authors:** Ilaria Giovannelli, Nadhim Bayatti, Abigail Brown, Dennis Wang, Marius Mickunas, William Camu, Jean-Luc Veyrune, Christine Payan, Cecilia Garlanda, Massimo Locati, Raul Juntas-Morales, Nicolas Pageot, Andrea Malaspina, Ulf Andreasson, Carey Suehs, Safa Saker, Christophe Masseguin, John de Vos, Henrik Zetterberg, Ammar Al-Chalabi, P Nigel Leigh, Timothy Tree, Gilbert Bensimon, Paul R Heath, Pamela J Shaw, Janine Kirby

**Affiliations:** 1 Department of Neuroscience, Sheffield Institute for Translational Neuroscience, University of Sheffield, Sheffield S10 2HQ, UK; 2 Department of Computer Science, University of Sheffield, Sheffield S1 4DP, UK; 3 Department of Immunobiology, Faculty of Life Science and Medicine, King's College London, London SE1 9RT, UK; 4 Clinique du Motoneurone, CHU Gui de Chaliac, University of Montpellier, Montpellier 34295, France; 5 Department of Cell and Tissue Engineering, University of Montpellier, CHU Montpellier, Montpellier 34000, France; 6 Department of Biostatistics, Clinical Epidemiology, Public Health and Innovation in Methodology (BESPIM), Nîmes University Hospital, Nîmes 30029, France; 7 Department of Pharmacology, AP-HP Sorbonne University, Pitié-Salpêtrière Hospital, F-75013 Paris, 75013 France; 8 Humanitas Clinical & Research Center—IRCCS, Milan 20089, Italy; 9 Humanitas University, Pieve Emanuele, Milan 20090, Italy; 10 Department of Medical Biotechnologies and Translational Medicine, University Milan, Milan 20133, Italy; 11 Department of Neuroimmunology, Barts and the London School of Medicine and Dentistry, Neuroscience and Trauma Centre, Institute of Cell and Molecular Medicine, London E1 2AT, UK; 12 Department of Psychiatry & Neurochemistry, University of Gothenburg, Mölndal 41345, Sweden; 13 Department of Medical Information, University of Montpellier, CHU Montpellier, Montpellier, France; 14 Department of Respiratory Diseases, University of Montpellier, CHU Montpellier, Montpellier 34090, France; 15 DNA and Cell Bank, Genethon, Evry 91000, France; 16 Delegation for Clinical Research and Innovation, Nîmes University Hospital, Nîmes 30029, France; 17 Clinical Neurochemistry Laboratory, Sahlgrenska University Hospital, Mölndal 43180, Sweden; 18 Department of Neurodegenerative Disease, UCL Institute of Neurology, Queen Square, London WC1N 3BG, UK; 19 UK Dementia Research Institute at UCL, London WC1E 6BT, UK; 20 Department of Basic and Clinical Neuroscience, Maurice Wohl Clinical Neuroscience Institute, King’s College London, London SE5 9RX, UK; 21 Department of Neurology, King’s College Hospital, London SE5 9RS, UK; 22 Brighton and Sussex Medical School, The Trafford Centre for Biomedical Research, Falmer, Brighton BN1 9RY, UK; 23 NIHR Biomedical Research Centre, Guy's and St Thomas' NHS Foundation Trust and King's College London, London SE1 9RT, UK; 24 Department of Pharmacology, Sorbonne University Médecine, F-75013 Paris 75013, France

**Keywords:** amyotrophic lateral sclerosis, transcriptomics, clinical trial, low-dose interleukin 2, regulatory T cells

## Abstract

Amyotrophic lateral sclerosis is a fatal neurodegenerative disease causing upper and lower motor neuron loss and currently no effective disease-modifying treatment is available. A pathological feature of this disease is neuroinflammation, a mechanism which involves both CNS-resident and peripheral immune system cells. Regulatory T-cells are immune-suppressive agents known to be dramatically and progressively decreased in patients with amyotrophic lateral sclerosis. Low-dose interleukin-2 promotes regulatory T-cell expansion and was proposed as an immune-modulatory strategy for this disease. A randomized placebo-controlled pilot phase-II clinical trial called Immuno-Modulation in Amyotrophic Lateral Sclerosis was carried out to test safety and activity of low-dose interleukin-2 in 36 amyotrophic lateral sclerosis patients (NCT02059759). Participants were randomized to 1MIU, 2MIU-low-dose interleukin-2 or placebo and underwent one injection daily for 5 days every 28 days for three cycles. In this report, we describe the results of microarray gene expression profiling of trial participants' leukocyte population. We identified a dose-dependent increase in regulatory T-cell markers at the end of the treatment period. Longitudinal analysis revealed an alteration and inhibition of inflammatory pathways occurring promptly at the end of the first treatment cycle. These responses are less pronounced following the end of the third treatment cycle, although an activation of immune-regulatory pathways, involving regulatory T-cells and T helper 2 cells, was evident only after the last cycle. This indicates a cumulative effect of repeated low-dose interleukin-2 administration on regulatory T-cells. Our analysis suggested the existence of inter-individual variation amongst trial participants and we therefore classified patients into low, moderate and high-regulatory T-cell-responders. NanoString profiling revealed substantial baseline differences between participant immunological transcript expression profiles with the least responsive patients showing a more inflammatory-prone phenotype at the beginning of the trial. Finally, we identified two genes in which pre-treatment expression levels correlated with the magnitude of drug responsiveness. Therefore, we proposed a two-biomarker based regression model able to predict patient regulatory T-cell-response to low-dose interleukin-2. These findings and the application of this methodology could be particularly relevant for future precision medicine approaches to treat amyotrophic lateral sclerosis.

## Teaser

Amyotrophic lateral sclerosis patients included in the IMODALS clinical trial (NCT02059759) were transcriptionally profiled to assess longitudinal blood expression changes. Low-dose-interleukin-2 was shown to promote dose and time-dependent Treg-marker upregulation. However, inter-individual variations were reported in the magnitude of Treg expansion and a two-biomarker model to predict target-engagement was proposed.

## Graphical Abstract

**Graphical Abstract:**
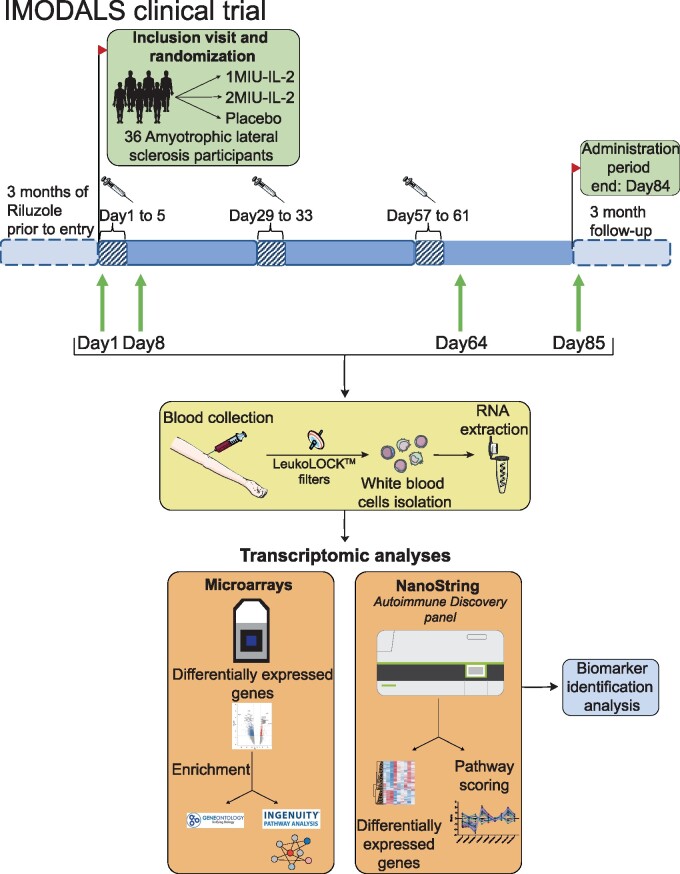

## Introduction

Amyotrophic lateral sclerosis (ALS) is a devastating neurodegenerative disease affecting upper and lower motor neurons, causing death usually within 3–5 years from the diagnosis.^1^ Currently, there is no effective disease modifying treatment available. The only approved drugs are riluzole and edaravone. However, these treatments produce only modest effects on life expectancy and disability progression.^2–6^

ALS is considered a multifactorial disease, as a series of mechanisms are implicated in its onset and progression.^7–9^ Amongst these, neuroinflammation, which involves both CNS-resident and peripheral immune cells, is currently of increasing interest.^10^ Regulatory T-cells (Tregs) represent a T-cell subset with immune-regulatory properties. They suppress excessive inflammatory responses, preventing the establishment of autoimmune disorders.^11^ Tregs were reported to be dramatically and progressively decreased in the peripheral blood of ALS patients, and these perturbations significantly correlated with disease progression and survival.^12–16^ In particular, at early disease stages, blood CD25^+^FOXP3^+^Tregs increase in number. This is perceived as an initial protective mechanism to suppress inflammation, whilst, as disease progresses, this attempted compensatory mechanism fails and Treg counts gradually and significantly decrease. Specifically, a shift from protective Tregs/T-helper(Th)2 and anti-inflammatory (M2) microglia phenotype to the pro-inflammatory Th1/Th17 and M1 microglia has been reported.^17–20^ Furthermore, ALS Tregs were functionally impaired and less effective at promoting immune-suppression.^14^^,^^16^^,^^21^ However, evidence of pharmacological restoration of Treg immune-modulatory functions by *in vitro* culturing in the presence of interleukin (IL)-2 and rapamycin was reported.^21^ Additionally, studies involving ALS mouse models showed that either the passive transfer of endogenous Tregs^18^ or intraperitoneal injection of rapamycin and IL-2c (IL-2 together with its monoclonal antibody)^16^ can efficiently expand the Treg count and prolong survival.

IL-2 is crucial for the differentiation, function and survival of Treg cells.^22^^,^^23^ In particular, low-dose IL-2 (ld-IL-2) has been shown to be safe and to promote significant Treg expansion in healthy volunteers included in a phase I clinical trial^24^ as well as in phase I/II^25–29^ or II^30^^,^^31^ studies involving patients suffering from several autoimmune disorders.

Given this background, ld-IL-2 was proposed as an immune-modulatory treatment for ALS. A randomized, placebo-controlled pilot phase-II clinical trial, Immuno-Modulation in Amyotrophic Lateral Sclerosis (IMODALS) was carried out to understand the safety and activity of ld-IL-2 in ALS patients. Thirty-six participants were recruited and randomly divided into three treatment arms: placebo, 1MIU and 2MIU IL-2. Ld-IL-2 appeared to be safe and well tolerated, with no serious adverse events reported.^32^

In this report, we examined the gene expression changes that occurred in the IMODALS cohort. In particular, the leukocyte population from participants has been transcriptionally profiled to assess differences in the response to the two treatment doses and to determine the longitudinal profile of gene expression changes throughout the trial. Consistent with the study of Camu et al., we found a dose-dependent and time-dependent upregulation of Treg-specific transcripts. Moreover, a downregulation of inflammatory-related pathways was evident in response to IL-2 administration. Finally, a regression model was generated to stratify participants and to predict the Treg-response to ld-IL-2 in patients with ALS.

## Materials and methods

### Trial design

IMODALS (NCT02059759) is a randomized, placebo-controlled phase-IIa clinical trial (Ethical approval number = 2014,09,01-ter). Thirty-six participants between 18 and 75 years of age were recruited and randomly assigned to one of three treatment arms: 1 million International Units (MIU) or 2MIU-IL-2 (Human recombinant IL-2 also known as aldesleukin or with the commercial name Proleukin^®^, Novartis. This will be subsequently referred to in the text as ld-IL-2) or placebo (5% glucose solution) as previously described.^32^ Participant characteristics are summarized in Table 1. Briefly, patients received subcutaneous injections once daily for 5 days every 28 days for a total of three administration cycles. For the purpose of transcriptomic profiling, blood was taken at four timepoints: day(D)1 or baseline; D8 - three days after the first injection cycle; D64 - three days after the last treatment cycle; and D85 - 24 days after the last treatment.

**Table 1:** Patient characteristics and response type.

Patient ID	Gender	Treatment type	Age at D1	Disease decline per month	Treg count at D64	2MIU-treated response classification
C01P001	Male	1MIU-IL-2	40.2	0.114	115.14	–
C01P005	Male	1MIU-IL-2	42.5	0.514	145.97	–
C01P008	Female	1MIU-IL-2	47.7	0.400	120.59	–
C01P010	Female	1MIU-IL-2	65.4	0.378	151.50	–
C01P013	Male	1MIU-IL-2	44.7	0.643	114.18	–
C01P020	Female	1MIU-IL-2	46.4	0.296	160.49	–
C01P023	Male	1MIU-IL-2	53.8	0.075	87.79	–
C01P024	Female	1MIU-IL-2	55.8	0.600	156.26	–
C01P027	Male	1MIU-IL-2	64.4	0.800	154.40	–
C01P033	Male	1MIU-IL-2	59.1	0.250	91.62	–
C01P035	Female	1MIU-IL-2	75.4	0.424	146.48	–
C01P038	Male	1MIU-IL-2	64.4	0.224	81.65	–
C01P003	Male	2MIU-IL-2	36.5	1.000	406.72	High
C01P004	Female	2MIU-IL-2	47.3	0.688	169.13	Moderate
C01P009	Female	2MIU-IL-2	68.8	0.692	434.47	High
C01P011	Male	2MIU-IL-2	76.6	1.500	144.34	Low
C01P016	Male	2MIU-IL-2	63.4	0.375	88.99	Low
C01P018	Male	2MIU-IL-2	59.8	0.444	117.88	Low
C01P021	Female	2MIU-IL-2	62.7	0.226	505.70	High
C01P026	Male	2MIU-IL-2	72.5	0.417	222.66	Moderate
C01P028	Male	2MIU-IL-2	63.4	0.148	176.08	Moderate
C01P032	Male	2MIU-IL-2	44.4	0.667	181.13	Moderate
C01P036	Male	2MIU-IL-2	56.5	0.209	52.22	Low
C01P037	Male	2MIU-IL-2	40.3	1.375	263.93	High
C01P002	Male	Placebo	47.5	0.375	30.08	–
C01P006	Male	Placebo	44.2	0.277	34.22	–
C01P007	Male	Placebo	49.2	0.351	55.14	–
C01P012	Female	Placebo	64.6	0.455	35.41	–
C01P014	Male	Placebo	52.6	0.350	90.37	–
C01P017	Male	Placebo	42.2	0.143	60.33	–
C01P022	Male	Placebo	63.6	0.429	32.99	–
C01P025	Male	Placebo	61.7	0.909	84.88	–
C01P030	Male	Placebo	58.5	0.313	25.63	–
C01P031	Male	Placebo	53.9	1.600	46.73	–
C01P034	Female	Placebo	69.7	0.579	57.52	–
C01P039	Female	Placebo	69.7	0.241	32.49	–

This table illustrates the gender, age, disease decline per month and treatment regimen of each participant. Moreover, Treg count at D64 is displayed and these data have been used to classify the patient response to 2MIU-IL-2 (high responders: Treg level at D64 > 250 cells/µl; moderate responders: between 150 and 250 cells/µl; low responders: < 150 cells/µl).

### Blood collection, processing and RNA extraction

Blood samples were collected at each study visit. LeukoLOCK^TM^ filters (Ambion^TM^) allowed white blood cell isolation and cells were stabilized in RNAlater (Thermofisher Scientific). Samples were stored at –80°C and shipped in dry ice to the Sheffield Institute for Translational Neuroscience (SITraN). Total RNA was extracted using LeukoLOCK^TM^ Total RNA isolation kit (Ambion^TM^) according to the manufacturer's instructions. RNA quantity and quality were assessed using Nanodrop ND-1000 (Thermo Scientific) and Agilent 2100 Bioanalyser (Agilent Technologies), respectively.

### Microarray preparation, normalization and quality control

Affymetrix ClariomD human microarrays (Applied Biosystem^TM^) were produced from white blood cell total RNA (200 ng of RNA with RNA integrity number > 8) as per manufacturer's instructions (GeneChip^TM^ WT PLUS Reagent Kit, Applied Biosystems and GeneChip^TM^ hybridization, Wash and Stain Kit, Applied Biosystems). A total of 107 microarrays were generated: 24 arrays form samples at D1 (12 placebo and 12 2MIU-IL-2-treated patients), 23 from D8 (12 placebo and 11 2MIU-IL-2), 36 from D64 (12 placebo, 12 1MIU and 12 2MIU-IL-2) and 24 from D85 (12 placebo and 12 2MIU-IL-2). One sample from D8, C01P011 failed RNA quality control (QC) and was therefore excluded from the analysis. Microarrays were normalized using the signal space transformation robust multi-chip analysis method. Expression Console^TM^ (Affymetrix) software was used to perform QC. All 107 microarrays passed QC and were used for downstream analysis.

### Differential expression analysis

Microarray data from D64 were analysed using Transcriptome Analysis Console 4.0 (Affymetrix) to identify differential expression between placebo, 1MIU-IL-2 and 2MIU-IL-2 treated patients. Gene-level analyses were performed and RefSeq annotations were used for enrichment analyses.

To accomplish complex multifactorial-designed expression analyses, the R package Limma was used.^33^ A RefSeq filtering was applied before launching the analyses. Three differential expression tests were performed in which treated patient transcriptomes at specific time points (D8, D64 and D85) were compared to baseline levels (D1) and normalised to the same comparison within the placebo group. These analyses are referred to as: ΔD8; ΔD64; ΔD85. Owing to samples being processed at different times, two batches were recognizable in our data. For this reason, data were adjusted for batch effects including batch identifiers as variables within the Limma statistical model.^33^^,^^34^

### Gene enrichment and pathway analysis

Lists of differentially expressed genes (DEGs), previously obtained from either TAC or Limma, were inputted into Enrichr^35^^,^^36^ to enrich for Gene Ontology Biological Process (GO BP). Long lists of altered GO BPs were produced with often redundant terms. To allow an easier interpretation of data, the software reduce and visualize gene ontology (REVIGO) was used. This grouped GO terms into clusters depending on their semantic similarity (cut-off = 0.5). A GO process was then chosen as a cluster representative to recapitulate similar terms.^37^ Finally, we used Ingenuity Pathway Analysis^®^ (IPA^®^) (Qiagen Inc.) and the Ingenuity Knowledge Base to retrieve significantly altered pathways in a data set allowing predictions of their activation/inhibition through the calculation of *z*-scores (activation: *z*-score > 0, inhibition: *z*-score < 0).^38^ DEG lists from the Limma comparisons ΔD8 and ΔD64 were submitted to IPA and analysed. Plots were generated using ggplot2 v3.2.2, gplots v3.1.1 or treemap v2.4.2 R libraries.

### Quantitative real-time PCR

Quantitative real-time polymerase chain reaction (qRT-PCR) was performed for microarray data validation. Four key Treg transcripts were selected: *FOXP3* (Hs.PT.58.3671186), *IL2RA* (Hs.PT.58.2187899), *CTLA4* (Hs.PT.58.3907580) and *IKZF2* (Hs.PT.58.2960172) (Integrated DNA Technologies Inc.). *GAPDH* (Hs.PT.39a.22214836) was used as a reference gene as, from microarray data, its expression was stable across all time points, with no variations amongst patient groups. All the 2MIU-IL-2 and placebo patient samples from the four time points were screened. Three technical replicates were completed for each condition.

200 ng of total RNA were retrotranscribed to cDNA using 5× qScript^TM^ DNA Supermix (Quantobio) by incubating samples at 25°C for 5 min, at 42°C for 30 min and at 85°C for 5 min. Subsequently, cDNA was mixed with 20× predesigned PrimeTime^®^ qPCR Assay (Integrated DNA Technologies Inc.) and 2× Luna^®^ Universal qPCR Master Mix (New England BioLabs^®^ Inc.). The mixture was incubated at 95°C for 3 min and 40 cycles of amplification at 95°C for 10 s and 60°C for 30 s were performed using C1000 Touch^TM^ Thermal cycler (Bio-Rad). Raw Ct values were retrieved using CFX Maestro^TM^ software (Bio-Rad). ΔCt, ΔΔCt and relative concentration (*R*) values were computed as follows:

ΔCt=Ct gene of interest – Ct reference gene

ΔΔCT=ΔCT – (average ΔCT placebo sample)

$$R=2^{-}{}^{\Delta \Delta \text{Ct}}.$$

### NanoString

Samples from four high (Treg level at D64 > 250 cell/µl blood), four low-Treg-responders (Treg level at D64 < 150 cell/µl) and four placebo patients at D1, D8 and D64 were investigated using the NanoString platform. Patient classification is illustrated in Table 1. Briefly, 300 ng of total RNA was mixed with capture and reporter probes from the auto-immune discovery panel. A hybridization period was allowed for 16 h at 65°C. Samples were scanned using nCounter^®^ SPRINT profiler (NanoString Technologies Inc.). The autoimmune discovery panel contains 755 mRNA targets: 740 immune-related transcripts and 15 housekeeping genes. NanoString data were analysed using nSolver^TM^4.0 and nCounter Advanced Analysis 2.0 (NanoString Technologies Inc.). Moreover, to identify which selection of transcripts were responsible for patient group differences, data were imported into Qlucore Omics Explorer (Qlucore) and multigroup comparison statistical analysis was performed.

### Multiple linear regression model

To identify predictive biomarkers of the outcome of the disease, each patient's baseline expression of genes included in the nanoString panel were correlated to their Treg level at D64 (flow cytometry data).^32^ Pearson's correlation tests were performed with associate *t*-test statistics. Six biomarker candidates—*SBNO2, BTLA, CD27, TRAF2, BLNK, TLR9*—were selected. Given that nanoString experiments were conducted only on a selection of patients, qRT-PCRs were performed in order to obtain data at D1 from all 12 2MIU-IL-2 treated patients and on three placebo samples as controls. qRT-PCR analyses were carried out as previously described and the following primers were used: *SBNO2* (Hs.PT.58.14833003), *BTLA* (Hs.PT.58.20005939), *CD27* (Hs.PT56a.27441991), *TRAF2* (Hs.PT.583116982), *BLNK* (Hs.PT.58.1645191), *TLR9* (Hs.PT.58.40576968) (Integrated DNA Technologies Inc.). Their averaged expression values from three replicates were correlated (Pearson's correlation) with their associated Treg counts at D64. Two transcripts—*TLR9* and *CD27*—were selected and a multiple linear regression model was generated using the R lm() function. Scatterplot3d package in R was used for 3D-plot generation. Finally, to test our model, a leave-one-out cross-validation (LOOCV) was performed using the package caret v6.0.86.

### Statistical analysis

Differential expression analysis was performed using TAC and Limma which use *F*-test and Empirical Bayes statistics to retrieve significant DEGs. In both cases, *P*-value (<0.05) and fold change (1.2 ≤ FC ≤ −1.2) cut-offs were applied. Fisher's exact test was used for gene enrichment by Enrichr and IPA (*P*-value < 0.05). To identify statistical differences between sample groups in qRT-PCR data, a two-way ANOVA analysis with either Tukey's or Sidak's correction for multiple comparisons was performed when analysing differences between treatment types at a single time or across different time points within the same administration regimen, respectively. Significant (*P* < 0.05) differentially expressed nanoString transcripts were assessed using Qlucore's multigroup comparison statistical analysis. Finally, the caret package in R was used for regression model statistics (lm() function, *P* < 0.05) and LOOCV.

### Data availability statement

Data supporting these study findings is available from the corresponding author, upon reasonable request. Array data can be found in Gene Expression Omnibus under the code GSE163560.

## Results

### Dose-dependency at D64

**Figure 1:**
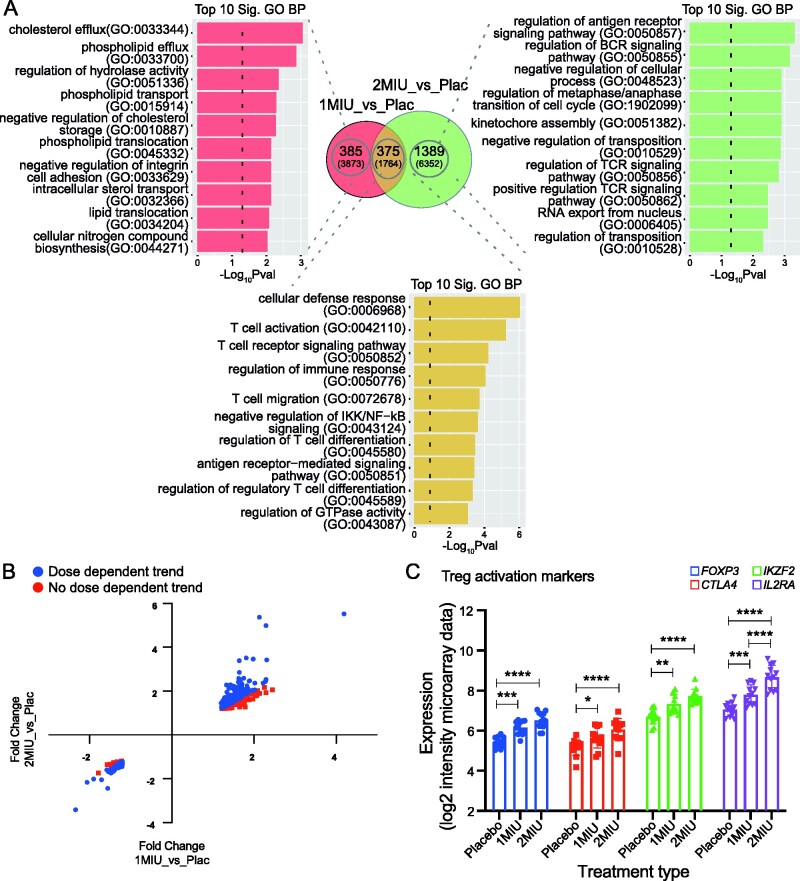
**End of the treatment (D64) analysis and dose-dependency.(A)** Venn diagram showing significant (*P*-value <0.05) differentially expressed genes (DEGs) from either 1MIU_vs_Placebo or 2MIU_vs_Placebo TAC (Transcriptome analysis console) comparisons. All altered transcripts are reported in brackets while RefSeq annotated transcripts are shown in bold. Overlapping common DEGs are also shown. For each RefSeq transcript list shown in the Venn diagram, the top 10 significant enriched Gene Ontology (GO) biological processes are plotted. *X*-axis: −Log_10_ (enrichment *P*-value); *y*-axis: GO term. (**B**) Scatter plot displaying 375 RefSeq DEGs altered in common within the two treatment groups and their fold changes resulting from either 1MIU_vs_Placebo (*X*-axis) or 2MIU_vs_Placebo (*Y*-axis) comparisons. 260 out of 375 genes (69.3%) show a dose-dependent expression (in blue) while transcripts showing no dose-dependent trend are represented in red. (**C**) The expression levels (SST-RMA normalized log_2_ of signal intensity from the microarrays) of 4 Treg activation markers—*FOXP3*, *CTLA4*, *IKZF2* and *IL2RA—*are shown. A significant dose-dependent upregulation of these transcripts is detected. Box plots show mean ± SD. A two-way ANOVA with Tukey's correction for multiple comparisons was conducted. *: Adjusted *P*-value < 0.05, **: Adjusted *P*-value < 0.01, ***: Adjusted *P*-value < 0.001, ****: Adjusted *P*-value < 0.0001. SST-RMA, signal space transformation robust multi-chip analysis method for microarray data normalization.

Differences in patient response to the two IL-2 doses were evaluated comparing participants' gene expression at D64, when the drug reaction was hypothesized to peak. To this end, microarrays from 1MIU and 2MIU-treated patients were compared to placebo (1MIU_vs_Placebo and 2MIU_vs_Placebo analyses) using TAC software. Following treatment with 1MIU-IL-2, 3873 transcripts (760 RefSeq-annotated) were significantly differentially expressed (2097 decreased, 1776 increased). The 2MIU_vs_Placebo comparison identified 6352 significantly differentially expressed transcripts (3530 decreased, 2822 increased), of which 1764 were RefSeq. Of importance, 1160 transcripts (375 RefSeq-annotated), were commonly differentially expressed (Fig. 1A).

To investigate the biological functions of the DEGs, gene ontology analyses were performed importing RefSeq lists into Enrichr. Sixty-two GO biological processes (BPs) were found to be significantly enriched in the unique list of DEGs characteristic of 1MIU_vs_Placebo, which were summarized into 27 clusters using REVIGO. In contrast, transcripts exclusively differentially expressed in 2MIU_vs_Placebo comparison were enriched in 129 GO BPs which formed 35 REVIGO clusters. Only a few immune-related processes were identified amongst 1MIU_vs_placebo unique list, whereas, evidence of immune-modulation (including regulation of T and B-cell receptors and regulation of antigen receptor mediated signalling) was identified amongst the 2MIU_vs_placebo-exclusive list (Fig. 1A and Supplementary Fig. 1). Importantly, 138 GO BPs were significantly enriched from the list of commonly DEGs in both comparisons (summarised in 26 REVIGO clusters). Interestingly, the large majority of these clusters were involved in immune-modulation: an extensive T-cell-subset regulation was reported together with modulation of production and secretion of multiple cytokines (Supplementary Fig. 1). This suggested that both doses were possibly able to promote Treg expansion and/or activation. Interestingly, transcripts involved in ganglioside and lipid metabolism were also reported as commonly altered (upregulated) following the administration of both ld-IL-2 doses (Supplementary Fig. 1).

To investigate potential differences in the magnitude of Treg activation, FCs from the two comparisons (1MIU_vs_Placebo and 2MIU_vs_Placebo) were compared. A dose-dependent reaction was found for 260 out of 375 (69.3%) RefSeq common DEGs: increased transcripts showed a greater level of upregulation, and similarly, decreased DEGs were more downregulated following the higher dose administration of IL-2 (Fig. 1B). Consistently with this, the expression of four key Treg activation markers (*FOXP3*, *CTLA4*, *IKZF2*, *IL2RA*) showed a dose-proportional upregulation (Fig. 1C). Given the greater degree of immune-regulation promoted by 2MIU-IL-2, we focussed on this higher-dose treatment for further analyses.

### Gene expression changes during 2MIU-IL-2 treatment

Patients' transcriptomic differences between treatment and placebo groups throughout the administration period were assessed from the microarray data using Limma. Two comparisons, ΔD8 and ΔD64, identified specific 2MIU-IL2-mediated changes at D8 and 64 compared to baseline and normalized to the placebo group.

**Figure 2:**
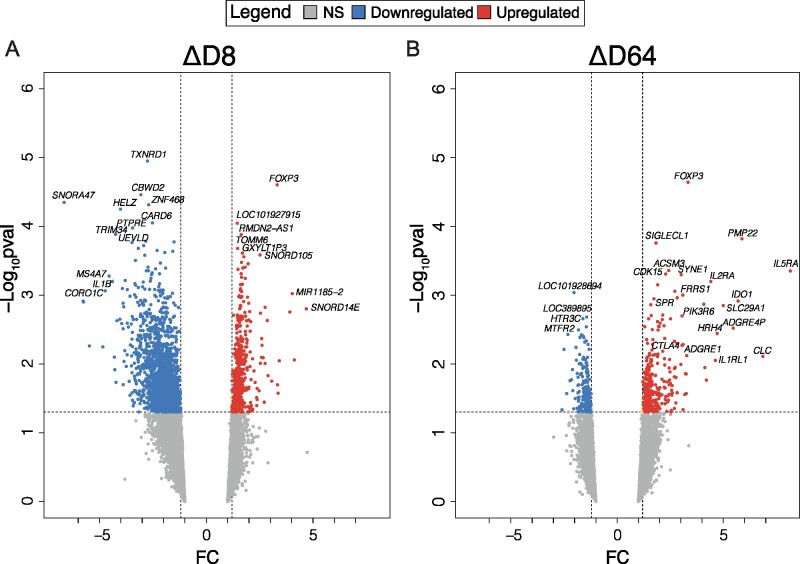
**Transcriptional changes during 2MIU-IL2 administration. Volcano plots displaying differentially expressed genes (DEGs) resulting from the comparisons ΔD8** (**A**) and ΔD64 (**B**). DEGs are plotted and colour-coded depending on their fold change (FC) and their significance levels (−Log_10_*P*-value): non-significant or transcripts that failed the FC cut-off are reported in grey, significant and with FC ≤−1.2 in blue and significant and with FC ≥1.2 in red. Three black lines are also shown: the horizontal line indicates the significance threshold (−Log_10_*P*-value = 1.3) and two vertical dotted lines mark the FC cut-off at −1.2 and 1.2, respectively. A widespread downregulation is detectable at D8, while at D64 an increased upregulation is reported amongst which some Treg makers are recognizable (Empirical Bayesian statistics was conducted using Limma to find significant DEGs).

The comparison ΔD8 identified 2635 RefSeq genes as significantly differentially expressed (1953 decreased, 682 increased), while only 525 RefSeq genes (198 decreased, 327 increased) resulted from ΔD64 (Fig. 2A and B). A widespread decrease in gene expression was reported after the first treatment cycle, while this effect seemed to be no longer present at later time points. In contrast, at D64 a more pronounced upregulation of gene expression was documented. Interestingly, in both comparisons, one of the most significant DEG is *FOXP3* which suggests an activation of Tregs starting from the first cycle and continuing throughout the administration period.

To understand the biological functions exerted by the identified DEGs, upregulated and downregulated lists of transcripts from the two comparisons were imported separately into Enrichr and GO analysis was conducted. The ΔD8 comparison revealed 448 downregulated GO BPs while ΔD64 showed only 35 downregulated significantly enriched terms. In contrast, analysis of upregulated DEGs identified 35 significantly enriched GO BPs in ΔD64 comparison while, only 10 processes were observed in ΔD8.

**Figure 3:**
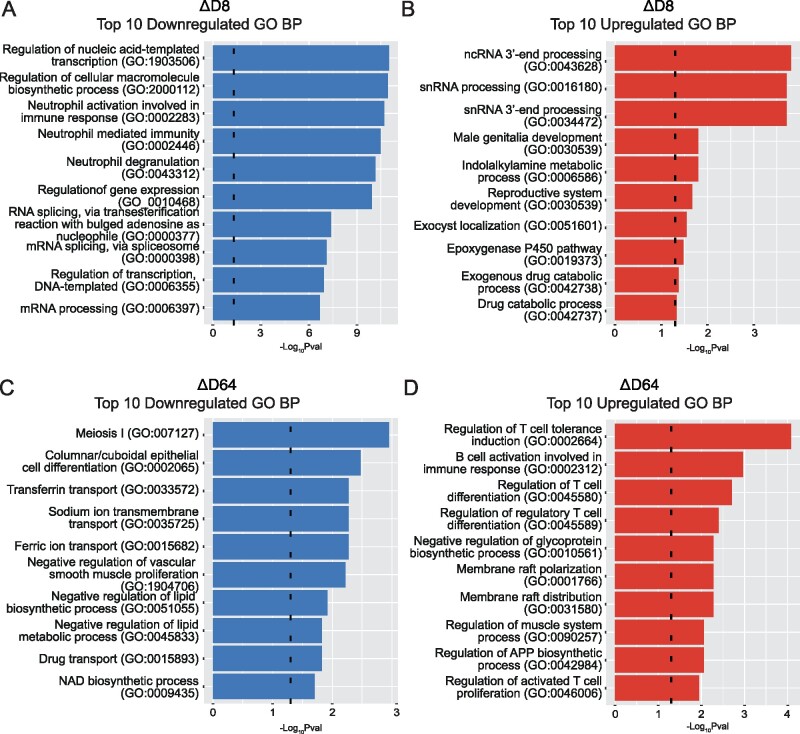
**Altered biological processes during 2MIU-IL2 administration. Bar plots resulting from GO Biological Processes (GO BP) enrichment analysis. In particular, the top 10 significant downregulated** (**A**) and upregulated (**B**) GO BPs from **Δ**D8 and top 10 significant downregulated (**C**) and upregulated (**D**) GO BPs from **Δ**D64 are shown. Significance threshold lines are reported in black (−Log_10_*P*-value = 1.3). A significant downregulation of pro-inflammatory processes involving neutrophils and an alteration in the RNA metabolism are observed at D8 while, later during the course of the trial, a significant upregulation of Treg processes is documented (Fisher's exact statistical test was performed using Enrichr to cluster transcripts into GO BP terms).

The most significant ΔD8 GO BPs revealed alterations (both increase and decrease) in different processes involved in RNA metabolism at D8. These included variations in both non-coding RNA and mRNA processing, splicing and gene expression regulation (Fig. 3A and B). Moreover, evidence of inflammatory suppression was also documented, with neutrophil activation and degranulation being significantly decreased. In contrast, the top 10 significant GO BPs from ΔD64 did not show a clear inflammatory process downregulation, while inhibition of other mechanisms including iron transport and lipid processing were observed. However, several immune-regulatory processes, especially those involved in Treg-activation and differentiation were upregulated at D64. Interestingly, enrichment in muscle regulatory process was also documented (Fig. 3C and D).

### Pathway alteration during 2MIU-IL-2 treatment

The lists of significant DEGs (*P*-value < 0.05 and 1.2 ≤ FC ≤ –1.2) outputted from ΔD8 and ΔD64 were imported into IPA to identify pathways altered longitudinally through the trial.

**Figure 4:**
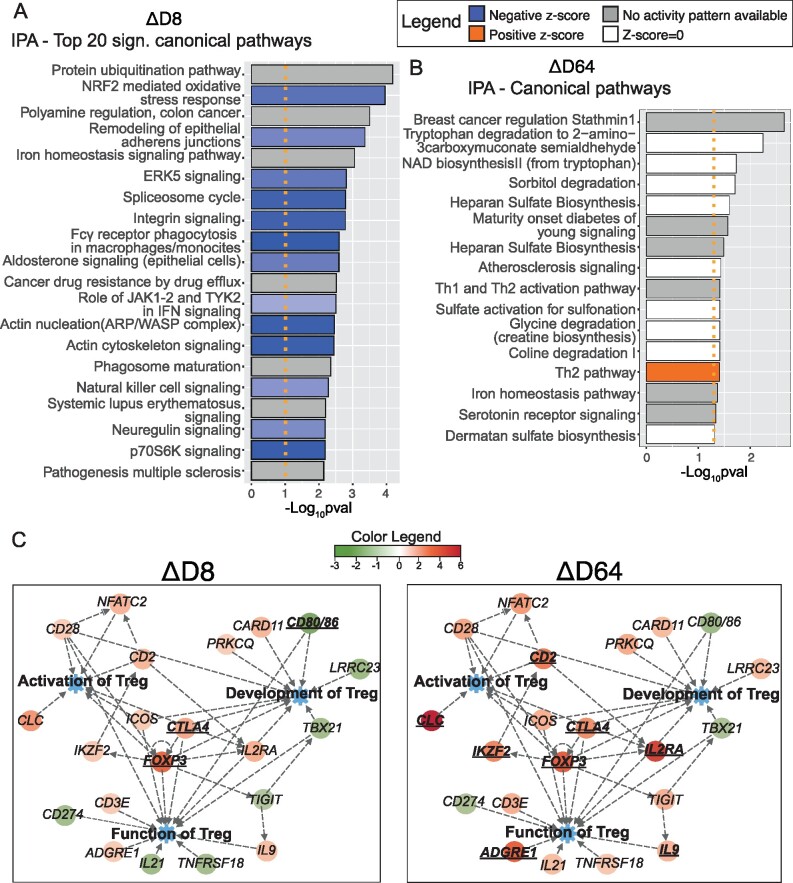
**Ingenuity Pathway Analysis (IPA). Bar plots displaying top 20 significant IPA canonical pathways. Activated (*z*-score > 0, in orange) or inhibited pathways (*z*-score < 0, in blue) resulting from the analysis ΔD8** (**A**) and ΔD64 (**B**) are shown. Significant pathways but with *z*-score equal to 0 (in white) or with no activation prediction available in the software (in grey) are also reported. A significance threshold line is displayed in orange (−Log_10_*P*-value = 1.3). A widespread downregulation of inflammatory pathways is detectable at D8 while fewer pathways are altered at D64. However, activation of Th2 is reported (A right-tailed Fisher’s Exact test was conducted to calculate significantly altered pathways and the *z*-score was computed to predict the activation state of each mechanism). (**C**) Customized pathways created with IPA displaying key regulators of activation, development and functions of Tregs. In particular, two pathway maps displaying fold changes (FCs) from ΔD8 and ΔD64 are juxtaposed for comparison. Significant (*P*-value < 0.05) differentially expressed genes are shown in bold and their gene symbol is underlined. A more prominent upregulation of Treg genes is reported at D64. Data were analysed through the use of IPA (QIAGEN Inc., https://www.qiagenbioinformatics.com/products/ingenuitypathway-analysis).

Seventy-seven pathways were altered in ΔD8, the top twenty significant processes are displayed in Fig. 4A while the complete list is available in Supplementary Table 1. An inhibition of inflammatory mechanisms was identified, with functions of both innate (phagocytic cells, neutrophils, eosinophils, macrophages/monocytes and natural killers) and adaptive immune cells (cytotoxic T lymphocytes and B cells) being decreased. Moreover, inhibition of NF-kB and signalling related to several cytokines were documented. Interestingly, negative regulation of pathways subserving autoimmune diseases, such as multiple sclerosis and systemic lupus erythematosus, were also found. Taken together, these results suggest a broader suppression of inflammatory processes, which are known to be activated in ALS, and also involved in other autoimmune pathologies. Additionally, the NRF2-mediated oxidative stress response pathway was decreased at D8 suggesting a reduction in oxidative stress. Moreover, two processes involved in CNS homeostasis, neuregulin and glioma signalling pathways, were inhibited.

Only 16 pathways were altered in ΔD64 (Fig. 4B and Supplementary Table 2). The majority of these were involved in the metabolism of several molecules, including amino acids and sulphate-containing compounds. However, two mechanisms implicated in T-cell subset modulation were altered. In particular, activation of the Th2 pathway was observed. Importantly, Th2 cells share some anti-inflammatory properties with Tregs mediated by IL-4 secretion.^18^^,^^19^ These findings suggest an activation of immune-modulatory processes at the later trial stages. This is further confirmed by our data showing an increased expression of key mediators of Treg development, activation and functions following the last injection cycle compared to the first (Fig. 4C).

IPA diseases and functions' analysis revealed an almost opposite pathway regulation occurring at D8 and D64. In fact, while the vast majority of the pathways showed negative *z*-scores, which indicates inhibition, after the first cycle, a trend towards inversion was visible later on with a positive *z*-score-skewed phenotype (Supplementary Figs. 2 and 3). This analysis reinforces previous observations indicating a rapid inhibition of inflammatory mechanisms after the first cycle. In fact, we have shown a repression of pathways involved in diapedesis of leukocytes—including phagocytes, monocytes, granulocytes and neutrophils—as well as developmental and functional inhibition of these cells. Cell death mechanisms were also found to be activated in leukocytes (Supplementary Fig. 2 and Supplementary Table 3). This may indicate a reorganization within immune cells happening at this time point. Concomitantly, downregulation of cell death processes in neurons and brain cells (‘cell death of brain’, ‘cell death of brain cells’, ‘apoptosis of cortical neurons’) was observed, together with a mild activation of the pro-survival ‘protection of cortical neurons’ which is particularly interesting in the context of ALS (Supplementary Fig. 2 and Supplementary Table 3). Furthermore, processes involved in metabolism, synthesis and production of reactive oxygen species (ROS) were inhibited at this time point.

In contrast, ΔD64 analysis revealed an extensive activation of regulatory processes (‘regulation of cells’, ‘suppression of lymphocytes’, ‘differentiation of induced Tregs’, ‘regulation of mononuclear leukocytes’ and ‘activation of Tregs’) and concomitant activation of leukocyte apoptosis (Supplementary Fig. 3). This suggested a more evident expansion of protective Tregs following the third cycle. However, unlike D8, processes involved in immune cell movement and recruitment seemed to increase at this time point. The reported activation of the ‘inflammatory response’ process may be perceived as counter intuitive. However, when investigating the transcripts involved in this process by the software, it was clear that several anti-inflammatory agents (such as *FOXP3*, *IDO1*, *IL2RA*) were also included and thus ‘inflammatory response’ included both pro and anti-inflammatory modulators (Supplementary Table 4).

### Gene expression changes after 2MIU-IL-2 treatment

**Figure 5:**
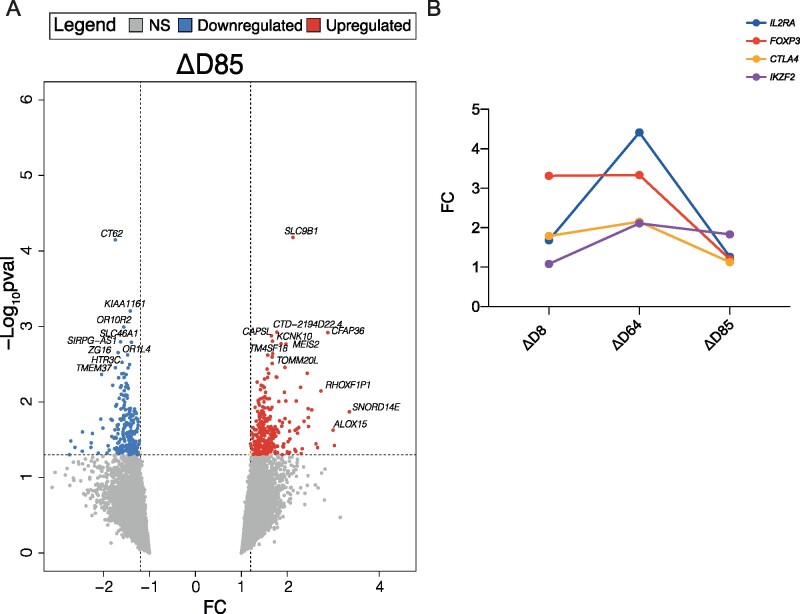
**Transcriptional changes during the follow-up period.** (**A**) Volcano plot showing differentially expressed genes (DEGs) resulting from the comparisons **Δ**D85. DEGs are plotted and colour-coded depending on their fold change (FC) and their significance levels (−Log_10_*P*-value): non-significant transcripts or transcripts that failed the FC cut-off are reported in grey, significant and with FC ≤−1.2 in blue and significant and with FC ≥1.2 in red. Three black lines are also shown: the horizontal line indicates the significance threshold (−Log_10_*P*-value = 1.3 or *P*-value = 0.05) and the two vertical dotted lines mark the FC cut-offs at −1.2 and 1.2, respectively (Empirical Bayesian statistics was conducted using Limma to find significant DEGs). (**B**) Plot displaying the variation in the expression of four key Treg activation markers—*FOXP3*, *IL2RA*, *CTLA4* and *IKZF2*—throughout and after the administration period. Their expression increases during the 2MIU-IL-2 treatment and peaks at D64 but the levels of expression decrease at D85. On the *x*-axis the different Limma comparisons are shown while on the *y*-axis the FC for each transcript is reported.

Subsequently, we analysed the transcriptional changes which occurred in IMODALS patients during the follow-up period to investigate whether the ld-IL-2 effect was sustained once treatment ceased. Limma was used to perform ΔD85 comparison. We found 508 RefSeq transcripts to be significantly differentially expressed (281 upregulated, 227 downregulated) (Fig. 5A). Interestingly, four main Treg activation markers—*FOXP3*, *IL2RA*, *CTLA4*, *IKZF2*—were no longer significantly differentially expressed. The comparison of their FC from ΔD85 with the results retrieved from the previous analyses during drug administration -ΔD8 and ΔD64- showed that the expression of Treg markers progressively increased during the administration cycles, but it dramatically decreased once treatment ceased (Fig. 5B). This suggested that 2MIU-IL-2-promoted Treg activation was no longer preserved at D85. Furthermore, to investigate the biological functions of the upregulated and downregulated DEGs, a GO analysis was carried out. Twenty-five upregulated and 23 downregulated significantly enriched GO BPs were found and none of these were related to immunological processes (Supplementary Fig. 4A and B). Interestingly though, an upregulation in mechanisms involved in CNS development, including neural tube development and neuronal differentiation, were observed, while downregulation of neurotransmitter transport and axonogenesis was documented.

### Microarray validation and variability in patient response

**Figure 6:**
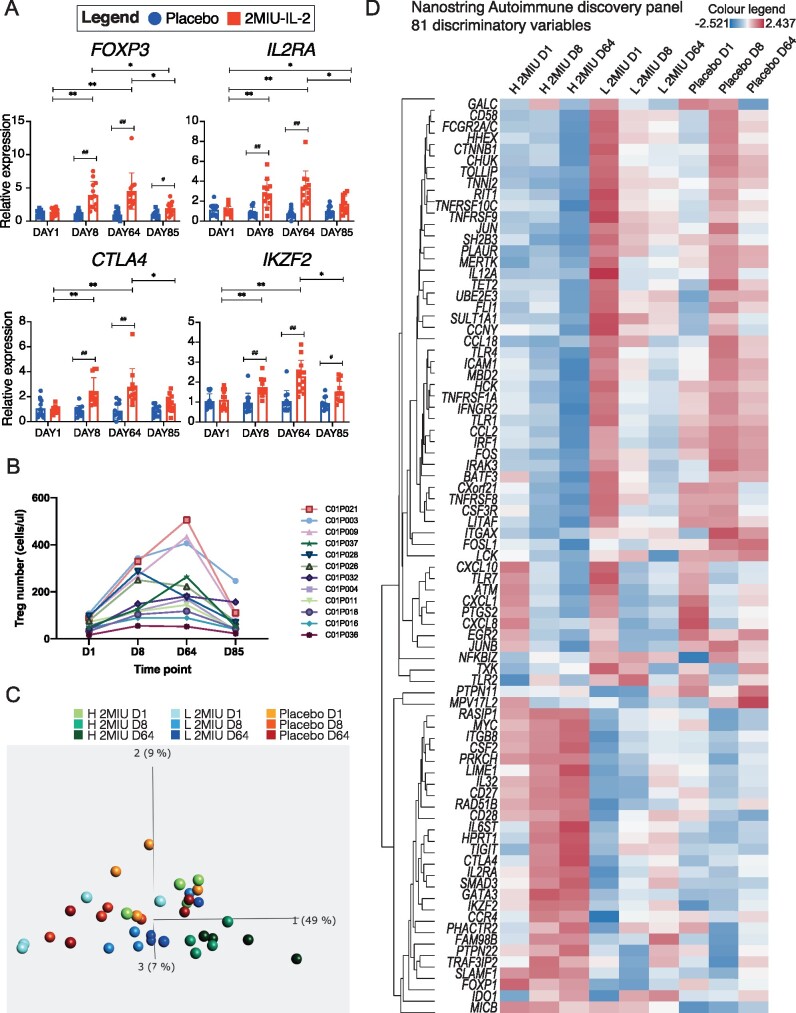
**Microarray validation and patient variability.** (**A**) Graphs showing expression of *FOXP3*, *IL2RA*, *CTLA4* and *IKZF2* in 2MIU-IL-2 treated (in red) and placebo (in blue) patients at the four different time points (D1, D8, D64 and D85). Data were generated through qRT-PCR. A time-dependent activation of these markers is reported in the ld-IL-2 group. Box plots display mean ± SD (technical replicates = 3). A two-way ANOVA with either Sidak (for comparisons between different treatment regimens, significant differences indicated with *) or Tukey (for comparisons between time points within the same treatment type, significant differences indicated with #) correction for multiple comparisons was conducted. * or #: Adjusted *P*-value <0.05, ** or ##: Adjusted *P*-value <0.01. (**B**) Graph displaying the number of Tregs per μl of blood of each 2MIU IL-2-treated participant at each time point (Flow-cytometry data). Patients are shown with different colours and their IDs are reported in the legend (**C**) PCA plot summarizing expression differences between samples depending on treatment regimen and time point (colour code legend is reported. H 2MIU D1, D8, D64 = high-Treg-responders at D1, D8 and D64; L 2MIU D1, D8, D64 = low-Treg-responders at D1, D8 and D64 and Placebo at D1, D8 and D64). High-Treg-responders from D8 and D64 are the most different samples. (Qlucore multi group comparison statistical test, *P*-value <0.05) (**D**) Hierarchically clustered heatmap displaying differences in the expression of 81 transcripts identified as discriminating variables from the PCA analysis in Fig. B. Gene expression variations across sample groups (H 2MIU D1, D8, D64 = high-Treg-responders at D1, D8 and D64; L 2MIU D1, D8, D64= low-Treg-responders at D1, D8 and D64 and Placebo at D1, D8 and D64) are displayed as *z*-scores (positive *z*-scores in red, negative in blue). An opposite expression between high and low-Treg-responders is detectable, especially at D1.

Microarray data validation was performed through qRT-PCR. Four key Treg markers (*FOXP3*, *IL2RA*, *CTLA4*, *IKZF2*) were chosen and samples from all 2MIU-IL-2-treated and placebo patients across all time points were screened. In line with microarray data, qRT-PCRs showed a time-dependent increase in the mRNA levels of these markers during the administration period which peaked at D64 (Fig. 6A). At D85 this upregulation was no longer sustained and transcripts showed levels more comparable to baseline. In contrast, no significant alterations were reported longitudinally within the placebo group. A considerable variability in the expression of Treg markers was registered amongst 2MIU-IL-2-treated patients. This suggested the existence of inter-individual variations in the response of ALS patients to the drug. This finding is in line with flow-cytometry data (Fig. 6B and previously published data^32^) showing a wide range of Treg count increases during the administration period (standard deviation of Treg cells measured at D8 = 101.7 and at D64 = 144.8). Given these results, we classified our 2MIU-IL-2-treated patients as low, moderate and high-Treg-responders according to their Treg levels registered at D64 (high = Treg level at D64 > 250 cell/µl; moderate = Treg level between 250 and 150 cell/µl; low = Treg level at D64 < 150 cell/µl; see Table 1). Importantly, no significant differences in age or in disease decline per month were reported amongst the three subgroups at baseline (One-way ANOVA with Tukey's correction).

To further investigate these inter-individual dissimilarities and to evaluate the existence of key transcriptional patterns underlying the different magnitude of Treg expansion in low and high-Treg-responders, nanoString analyses were performed. In particular, we aimed to find immunological transcript dissimilarities in these participants across the administration period (D1, D8 and D64) and, for this reason, the nanoString autoimmune discovery panel was used. We performed a principal component analysis (PCA) analysis to visualize transcriptional-driven group separation (Fig. 6C). High-Treg-responder samples from D8 and D64 appeared to be spatially distanced, whereas, placebo and low-Treg-responders were more dispersed and partially overlapped. Interestingly, low at D64 and high-Treg-responders at D1 were overlapping (Fig. 6C). We then identified a cluster of 81 discriminatory transcripts that were responsible for this PCA spatial separation (Fig. 6D). This included genes encoding both pro- and anti-inflammatory agents. At baseline, an almost opposite trend was reported between high and low-Treg-responders in this selected panel of transcripts.

**Figure 7:**
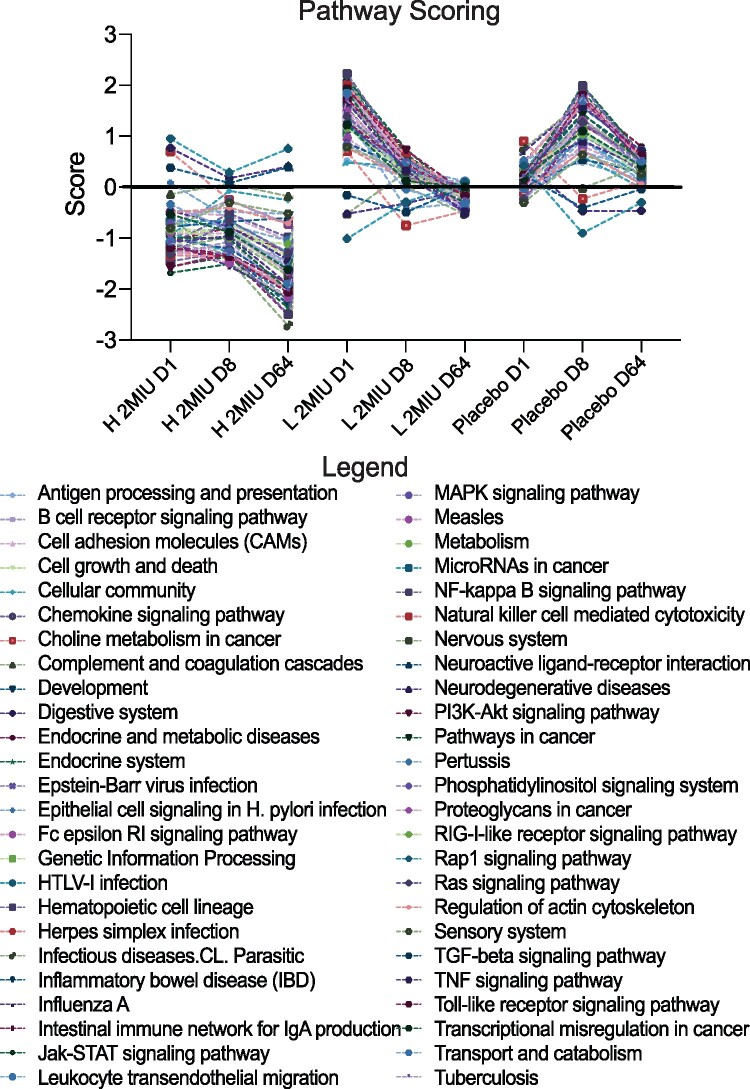
**Pathway scoring analysis.** Graph showing results from the pathway scoring analysis performed using Advanced Analysis nSolver software. Pathway activation scores are plotted as a function of the different treatment type and time points (H 2MIU D1, D8, D64 = high-Treg-responders at D1, D8 and D64; L 2MIU D1, D8, D64 = low-Treg-responders at D1, D8 and D64 and Placebo at D1, D8 and D64). An evident downregulation of several inflammatory pathways is demonstrated in both high and low-Treg-responders although baseline differences were observed between the two treated subgroups.

Lastly, pathway scoring analysis was performed. A sharp downregulation of pro-inflammatory pathways was reported in both high and low 2MIU-IL-2 Treg-responders (Fig. 7). However, baseline scores were considerably different between these two groups. Collectively, these results suggested that an almost opposite immunological phenotype characterized high and low-Treg-responders prior to drug administration. Therefore, this might have significantly influenced the participant reaction to 2MIU-IL-2.

### Predictive biomarker identification

Given the inter-individual differences observed, we aimed to identify transcripts whose expression at D1 could predict 2MIU-IL-2-mediated Treg-response. In particular, we aimed to identify biomarkers of gene expression that could predict target engagement at the end of the trial (expressed as Tregs count at D64). To this end, we conducted a preliminary screening by correlating the expression of all the transcripts included in the autoimmune nanoString panel at D1 with Treg levels registered at D64 or with the expression of *IL2RA* at D64. Thirty-five genes showed expression levels at D1 which significantly correlated with both the variables and six biomarker candidates (*TLR9*, *SBNO2*, *CD27*, *BLNK*, *TRAF2* and *BTLA*) were selected which had the best correlation coefficients and statistical significance (Supplementary Table 5). However, given that only high and low-Treg-responders were screened through nanoString, the expression of these transcripts in all patients was investigated through qRT-PCR.

**Figure 8:**
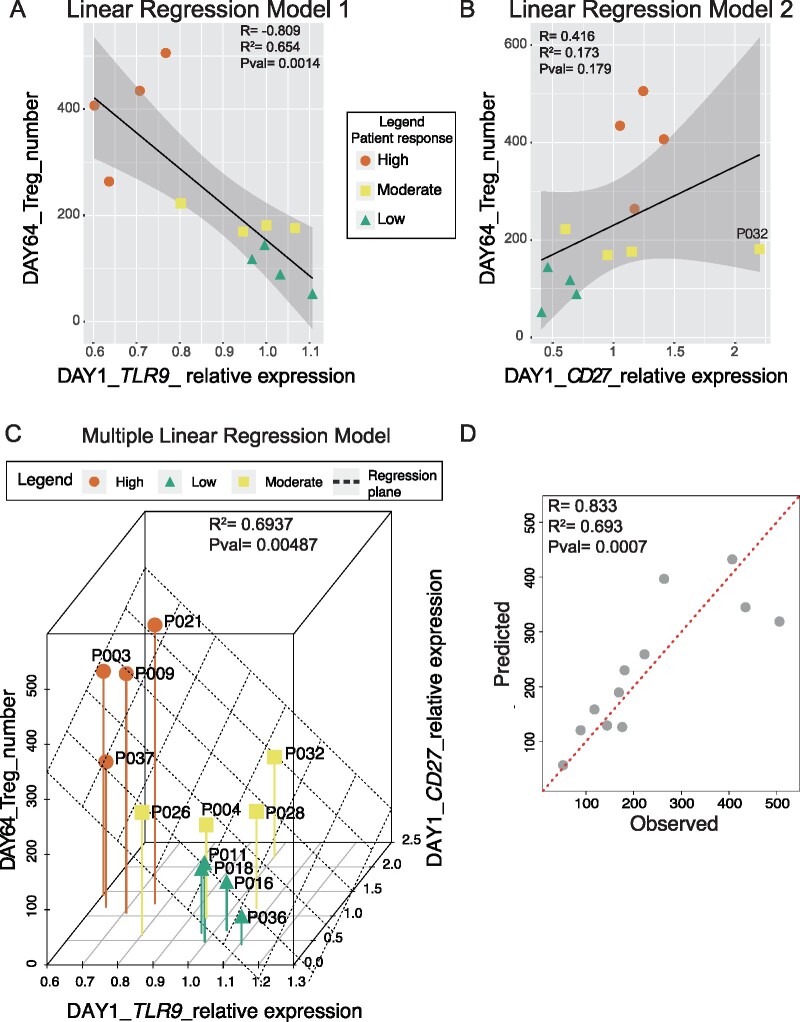
**Biomarker identification analysis.** Linear regression models describing the correlation between the expression of *TLR9* (**A**) and *CD27* (**B**) at the baseline (D1) and the Treg number at D64 for each 2MIU-IL-2 treated patient. Each dot represents a trial participant (average expression values computed from 3 qRT-PCR experiments, *N* = 3) and they are colour-coded depending on their Treg-response type: high (orange dots), moderate (yellow squares) and low (green triangles). A regression line (black) and its regression confidence bands (grey) are also shown. Linear regression model for *TLR9* (**A**: *R* = −0.809, *R*^2^ = 0.654, *P*-value = 0.0014) was stronger than the model for *CD27* (**B**: *R* = 0.416, *R*^2^= 0.173, *P*-value = 0.179) (**C**) Multiple linear regression analysis indicating the relationship between 2 predictors (expression of *TLR9* and *CD27* at D1) and the response variable (number of Tregs at D64). A good prediction model was obtained (*R*^2^ = 0.6937and *P*-value = 0.00487). Each dot represents a patient and they are colour-coded depending on their Treg-response type: high (orange dots), moderate (yellow squares) and low (green triangles). The regression plane is also displayed in black. (**D**) Plot showing the correlation between flow-cytometry-measured Treg counts (observed, *X*-axis) and Treg numbers predicted by our multiple linear model (predicted, *Y*-axis). Correlation metrics (*R* = 0.833, *R*^2^ = 0.693, *P*-value= 0.0007) suggest an acceptable predictive model. Each dot represents a sample and a red dotted regression line is also shown.

*TLR9* and *CD27* were selected because their expression at D1 showed the best correlation with Treg count at D64. The rest of the proposed biomarkers instead did not significantly correlate and for this reason were excluded from further analyses (Supplementary Table 6). A strong negative correlation was observed for *TLR9* (*R* = −0.809, *R*^2^ = 0.654, *P*-value = 0.0014) (Fig. 8A). A milder positive correlation was reported for *CD27* (*R* = 0.416, *R*^2^ = 0.173, *P*-value = 0.179) (Fig. 8B).

Expression data from *TLR9* and *CD27* at D1 were then combined and used as variables to create a more robust multiple linear regression model (Fig. 8C) which performed better than single linear models and with metrics which appeared encouraging (*R*^2^ = 0.694, Adjusted_*R*^2^ = 0.626, *P*-value = 0.005). In particular, coefficient of determination (*R*^2^) and fitting root mean squared error (RMSE) obtained with this model were better than the values from *TLR9* alone ($R_{\mathrm{multiple}}^{2}=0.694$, RMSE_multiple_ = 76.7, $R_{\mathrm{TLR}9}^{2}=0.654$, RMSE_TLR9_ = 81.4).

Therefore, the computed multiple model formula (Formula 1) could be used to predict ALS patient Treg count after three 2MIU-IL-2 administration cycles once the baseline level of *CD27* and *TLR9* expression is known.

$$\mathrm{Formula}\;1:\;\mathrm{Treg}\;\mathrm{number}\;D64=724.57+\left(59.49*CD27\right)+(-625.00*\mathrm{TLR}9)$$

The model performance was assessed through LOOCV and the emerging metric was computed (RMSE = 84.28). Although this might be perceived as a high score, it needs to be stressed that RMSE is a scale-dependent measurement which means that it needs to be interpreted in the context of the range of values assumed by the dependent variable (Treg count at D64). As mentioned before, the standard deviation for the Treg count was quite high (SD_Treg_D64_ = 144.8) and thus, if compared with the LOOCV RMSE, we could conclude that the model was sufficiently robust when predicting new sets of data. Moreover, the predictive ability was also inspected correlating experimentally measured, or observed Treg counts at D64, with the predicted values (Fig. 8D). As expected, a strong positive correlation was observed (*R* = 0.833, *P*-value = 0.0007).

## Discussion

Ld-IL-2 has been proposed as an immune-modulatory strategy for ALS to promote Treg expansion, dampen neuroinflammation and increase patient survival.^39^ A pilot phase-II clinical trial was carried out to test the safety and activity of 1MIU or 2MIU-IL-2.^32^ In the present report, transcriptomic profiling of patient leukocytes was performed to evaluate the effects of ld-IL-2 throughout the IMODALS trial.

Firstly, we assessed whether the two ld-IL-2 doses induced different reactions in IMODALS participants. To this end, microarray expression data at D64 from all treatment arms were compared. DEGs and GO enrichment analyses revealed an immune-regulation, with T-cell subset and the signalling related to several cytokines being altered in both treatment arms. This suggested that both doses efficiently promoted Treg expansion. However, 2MIU-IL-2 induced a more pronounced immune-regulation and a dose-dependent expression was identified for the majority (∼70%) of the common DEGs. This is in line with results reported by Camu et al. which showed a dose-dependent increase in the percentage of Tregs. Interestingly, ganglioside, ceramide and lipid metabolism were commonly altered at the end of both 1MIU and 2MIU-IL-2 treatment regimens. These mechanisms, especially ganglioside biosynthesis, have been implicated in the pathogenesis of ALS.^40^ Thus, modulation of these metabolic processes could potentially contribute to the beneficial effects of ld-IL-2 on the pathophysiology of ALS. Given the dose-dependency, further analyses were conducted comparing only 2MIU-IL-2 with placebo participants.

We then analysed our microarray data longitudinally. A broad differential expression was observed in the comparison ΔD8. This can be interpreted as a rapid response to the newly administered drug after the first treatment cycle. Over the trial course though, an adaptation probably occurred and less DEGs were reported in ΔD64.

GO gene enrichment and IPA^®^ analyses were carried out to identify pathways longitudinally altered throughout the trial. An inhibition of inflammatory processes was registered after the first administration cycle. In particular, transcripts associated with the function of both innate (neutrophils, eosinophils, macrophages and natural killers) and adaptive immune cells (cytotoxic T-cells and B-cells) were downregulated at D8. Of importance, evidence of dysregulation in these immune system cells is reported in the ALS literature. Neutrophil activation correlates with disease severity and predicts patient survival.^41–43^ Peripheral blood natural killer alterations and their infiltration into the spinal cord and motor cortex of ALS patients has been reported.^44^^,^^45^ Although the role of monocytes/macrophages in ALS is still to be elucidated, recently, total CD14+ monocyte levels appeared significantly higher, with increased M1 activation and pro-inflammatory features.^17^^,^^46^^,^^47^ Moreover, adaptive immune system cells such as cytotoxic T-cells are known to infiltrate the CNS and to contribute to the loss of motor neurons.^48^^,^^49^

NF-kB, cytokine signalling pathways and pathways promoting diapedesis of leukocytes were also down-regulated after the first cycle and these may contribute to the ld-IL-2-induced immune-suppression observed early after treatment initiation. Leukocyte cell death mechanisms were reported to be activated at this time point. However, this process specificity towards leukocytes should be investigated to exclude possible toxic effects on other cell types. Concomitantly, pathway analysis showed evidence of downregulation of cell death mechanisms in brain and cortical neurons which may indicate a positive pro-survival effect. Taken together, these data suggest that the initial suppression of the immune system may have beneficial effects by dampening the widespread inflammation characteristic of ALS.

This seems to be a short-term effect given that the downregulation of the majority of the inflammatory pathways was no longer present at D64. However, a robust activation of immune-regulatory processes was observed at this time point, with evidence of time-dependent activation of both Tregs and Th2 being observed. Of importance, Th2 share anti-inflammatory and neuroprotective properties with Tregs and both cell types are reduced in ALS patients.^18^^,^^19^ Lymphocyte suppression and apoptosis induction was recorded at D64 which might be due to the inhibitory action of Treg/Th2 cells on inflammatory mediators.

Alongside immunological pathway alterations, ld-IL-2 appeared to affect other biological mechanisms. Metabolism, synthesis and production of ROS as well as the NFR2-mediated oxidative stress response pathways were inhibited at D8. Excessive ROS generation, oxidative stress and consequently oxidative damage are key mechanisms in the pathophysiology of ALS.^50^ NRF2 is a transcription factor which promotes a cytoprotective response including induction of anti-oxidant and anti-inflammatory gene expression.^51^ Dysregulation in NRF2 signalling pathways has been reported in ALS and this is now considered a promising therapeutic target.^52^^,^^53^ The observed NRF2 pathway inhibition may represent a detrimental short-term effect of ld-IL-2 given that this suppression was not detected at D64. However, considering the concurrent downregulation of mechanisms involved in ROS production, we can speculate that ld-IL-2 can instead have an anti-oxidant effect by dampening the production of ROS and therefore reducing the need for the NRF2-associated stress response. However, further investigation is necessary to verify this hypothesis.

Subsequently, we investigated the transcriptional profiles of IMODALS participants during the post-treatment period. We found that the ld-IL-2 immune-regulatory effect was no longer preserved at D85 and the expression of Treg markers was returning towards baseline levels. These findings are consistent with those reported by Camu et al. At the D85, GO enrichment analysis revealed no significant alteration in immunological processes. However, some CNS developmental mechanisms were upregulated (neural tube formation, development and closure and positive regulation of neuron differentiation) whereas processes involved in axonogenesis and neurotransmitter transport were downregulated.

Microarray validation was also carried out through qRT-PCR screening of four Treg markers. In line with the microarray data, a time-dependent increase in the expression of all of these transcripts was observed within the 2MIU-treated group whereas no significant changes were observed in placebo participants. In particular, the expression of Treg-specific markers peaked at D64, suggesting the existence of a cumulative reaction to successive doses of 1d-IL-2. In line with microarray data, at D85 these effects of 2MIU-IL-2 effect were no longer sustained. This analysis also revealed inter-individual differences in terms of the patient response to ld-IL-2. This is consistent with data presented by Camu et al and the significant variability in terms of Treg expansion previously reported in clinical trials evaluating ld-IL-2 in several autoimmune disorders.^26^^,^^28^^,^^31^^,^^54^

Thus, the aim was to investigate the existence of differences in participants' gene expression that could reflect the observed variation in Treg expansion. For this reason, patients were classified patients into high, moderate and low-Treg-responders depending on their Treg levels measured at D64. We then performed nanoString experiments to characterize immunological transcriptional differences between high and low-Treg-responding participants. We successfully identified an 81 transcript cluster which was able to discriminate between the two different groups. This analysis revealed an almost opposite immune phenotype at baseline between high and low-Treg-responders. Furthermore, pathway scoring analysis revealed that a considerable amount of inflammatory pathways were progressively downregulated in both types of ld-IL-2 Treg-responders. However, a more inflammatory-prone phenotype seemed to characterize low-Treg-responders at baseline. This might have exerted a significant influence on their ability to mount a strong Treg-response to ld-IL-2.

Finally, we aimed to identify smaller sets of transcripts capable of predicting the patient Treg-response to ld-IL-2. To this end, the expression of all the genes included in the nanoString panel at D1 was correlated with Treg levels^32^ at D64. Two transcripts were selected: *TLR9* which showed a strong negative correlation and *CD27* which correlated positively with the D64 Treg count. Toll-like receptors (TLR) are a class of pattern recognition receptors implicated in pathogen identification and immune-response initiation. Dysregulation of TLR9 signalling has been associated with several autoimmune and neurodegenerative diseases.^55^^,^^56^ Interestingly, increased *TLR9* expression was reported in the spinal cord of SOD1^G93A^ transgenic mice.^57^ CD27 is a member of the tumour necrosis factor receptor family. The binding of its ligand, CD70, leads to immune cell activation and a pro-inflammatory response. However, CD27-CD70 has key role in Treg generation in the thymus and genetic ablation of either protein leads to a reduced number of thymic Tregs.^58^ Moreover, CD27-deficiency caused reduction in Treg cell numbers.^59^^,^^60^ Interestingly, Zhao et al reported *CD27* as one of the downregulated transcripts solely differentially expressed in the monocytes of rapidly progressive ALS patients.^61^

*TLR9* and *CD27* expression data were then combined and a robust multiple linear regression model with good predictive capacity (*R*^2^ = 0.694, Adjusted *R*^2^ = 0.626, *P*-value = 0.005) was generated. Therefore, the model formula should be able to forecast the magnitude of Treg expansion in ALS patients after measuring the baseline evels of *TLR9* and *CD27* expression. These can be considered as biomarkers of target engagement as their expression at recruitment is proposed to be predictive of cellular target stimulation and therefore Treg expansion promoted by three cycles of ld-IL-2. Importantly, if this pharmacological treatment provides evidence of clinical efficacy in ALS patients in future phase II/III trials, our model may be valuable for future precision medicine approaches, to allow patient stratification and identification of the best therapeutic strategy for each individual.

A limitation of our study is the small participant group size, which particularly affects our analyses. In particular, we recognize age and sex as crucial variables possibly affecting ALS phenotype. However, in such a limited sized cohort, we believe that stratifying for these factors would have had a considerable impact in reducing the statistical power of our analysis. Moreover, this limited participant size also substantially affected our proposed predictive biomarker research. However, preliminary evaluations are necessary to examine the safety and tolerability of new proposed drugs. Further investigation is needed to verify the immune-modulatory transcriptomic effect of ld-IL-2 and to validate our predictive model. To this end, a randomized, placebo-controlled, double-blind phase-II clinical trial, MIROCALS (NCT03039673), is currently active and aims to evaluate the effect of 2MIU-IL-2 in a larger ALS cohort of 220 participants. This will allow more complex examination of gene expression variations throughout the trial and further investigation of the variability in Treg-response as well as the validation of the robustness of our proposed predictive model.

In conclusion, transcriptional profiling of leukocytes from participants in the IMODALS trial revealed evidence of immune modulation following ld-IL-2 administration. Inter-individual differences in the treatment responses to 2MIU-IL-2 were observed and a two-biomarker-based model able to predict drug-induced Treg-response was identified. This could be particularly relevant in future precision medicine approaches for ALS.

## Supplementary material

Supplementary material is available at *Brain Communications* online.

## Supplementary Material

fcab141_Supplementary_Data

## Abbreviations

ALS =: amyotrophic lateral sclerosis
D =: day
DEG =: differentially expressed gene
FC =: fold change
GO BP =: gene ontology biological process
GO =: gene ontology
IL =: interleukin
IMODALS =: immune-modulation in amyotrophic lateral sclerosis
IPA =: ingenuity pathway analysis
Ld-IL-2 =: low-dose IL-2
LOOCV =: leave-one-out cross-validation
MIU =: million international units
PCA =: principal component analysis
QC =: quality control
qRT-PCR =: quantitative real-time polymerase chain reaction
*R*^2^ =: coefficient of determination
REVIGO =: reduce and visualize gene ontology
RMSE =: root mean squared error
ROS =: reactive oxygen species
Th =: T helper
Treg =: regulatory T-cell
