# Supplementary material for: Amyotrophic lateral sclerosis transcriptomics reveals immunological effects of low-dose interleukin-2
Source: Brain Commun. 2021 Jun 29;3(3):fcab141. doi: 10.1093/braincomms/fcab141 (PMC8364666; doi:10.1093/braincomms/fcab141)
Supplement: fcab141_Supplementary_Data [file fcab141_supplementary_data.pdf]

Supplementary figure 1: Treemaps showing REVIGO clustered Gene Ontology biological processes (GO BPs) obtained from three differentially expressed gene lists generated with TAC: unique lists of differentially expressed genes characteristic of either 1MIU_vs_Placebo (A) or 2MIU_vs_Placebo (B) and list of commonly differentially expressed in both comparisons (C). GO BP terms belonging to the same REVIGO cluster are grouped and displayed in the same rectangle and cluster representatives are reported with grey tags. GO BP terms are sized and colour-coded depending on their significance levels ($-Log_{10}$pvalue).

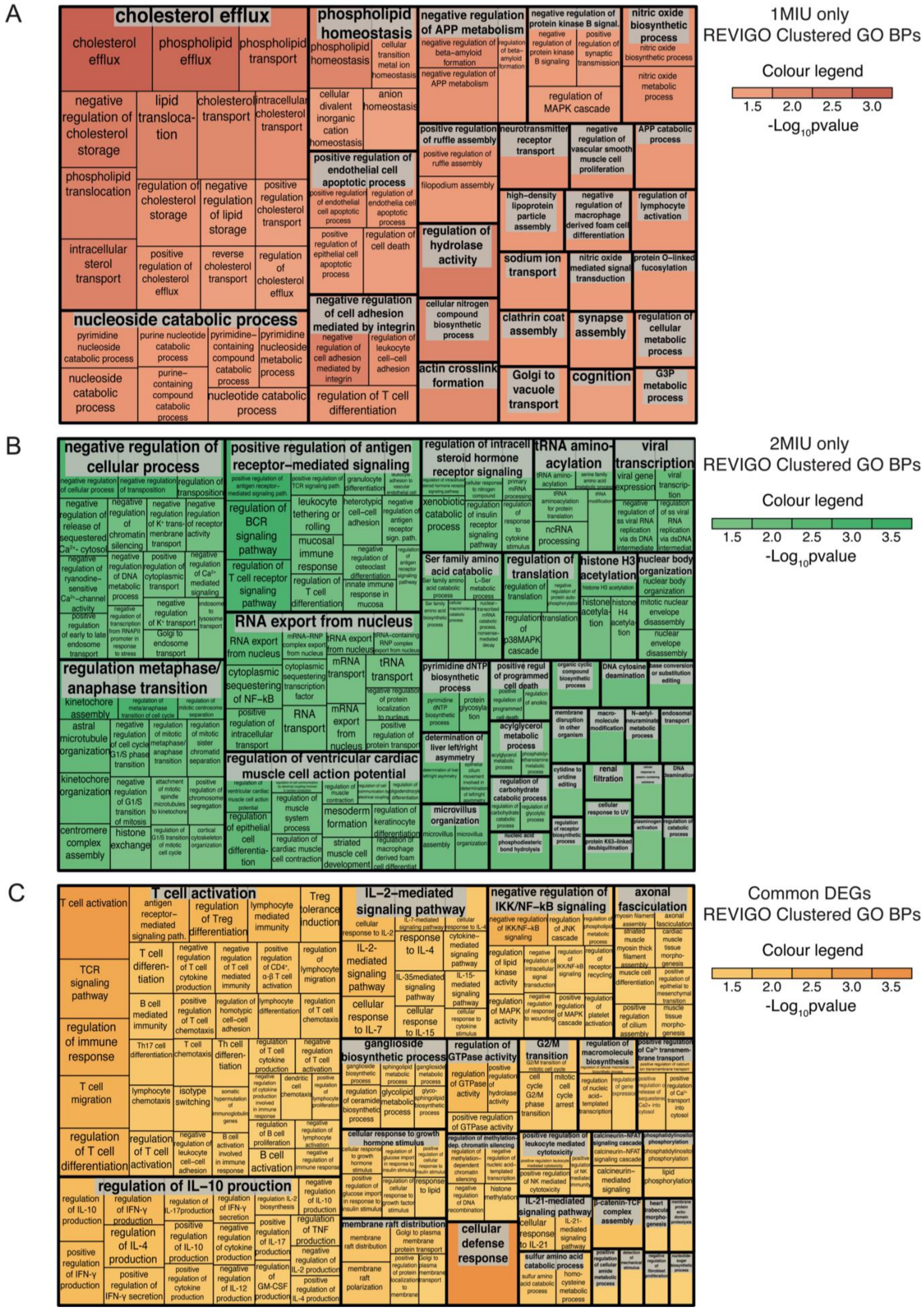

Supplementary figure 2: Treemap summarising altered IPA diseases and biological functions resulting from ΔD8 comparison. Each box represents a biological function or a disease and they are sized and coloured by their z-score (z-score > 0 in the orange colour scale or < 0 in the blue scale). Diseases and functions are clustered by IPA into master categories for which the name is displayed by a grey label. Data were analyzed through the use of IPA (QIAGEN Inc., https://www.qiagenbioinformatics.com/products/ingenuitypathway-analysis).

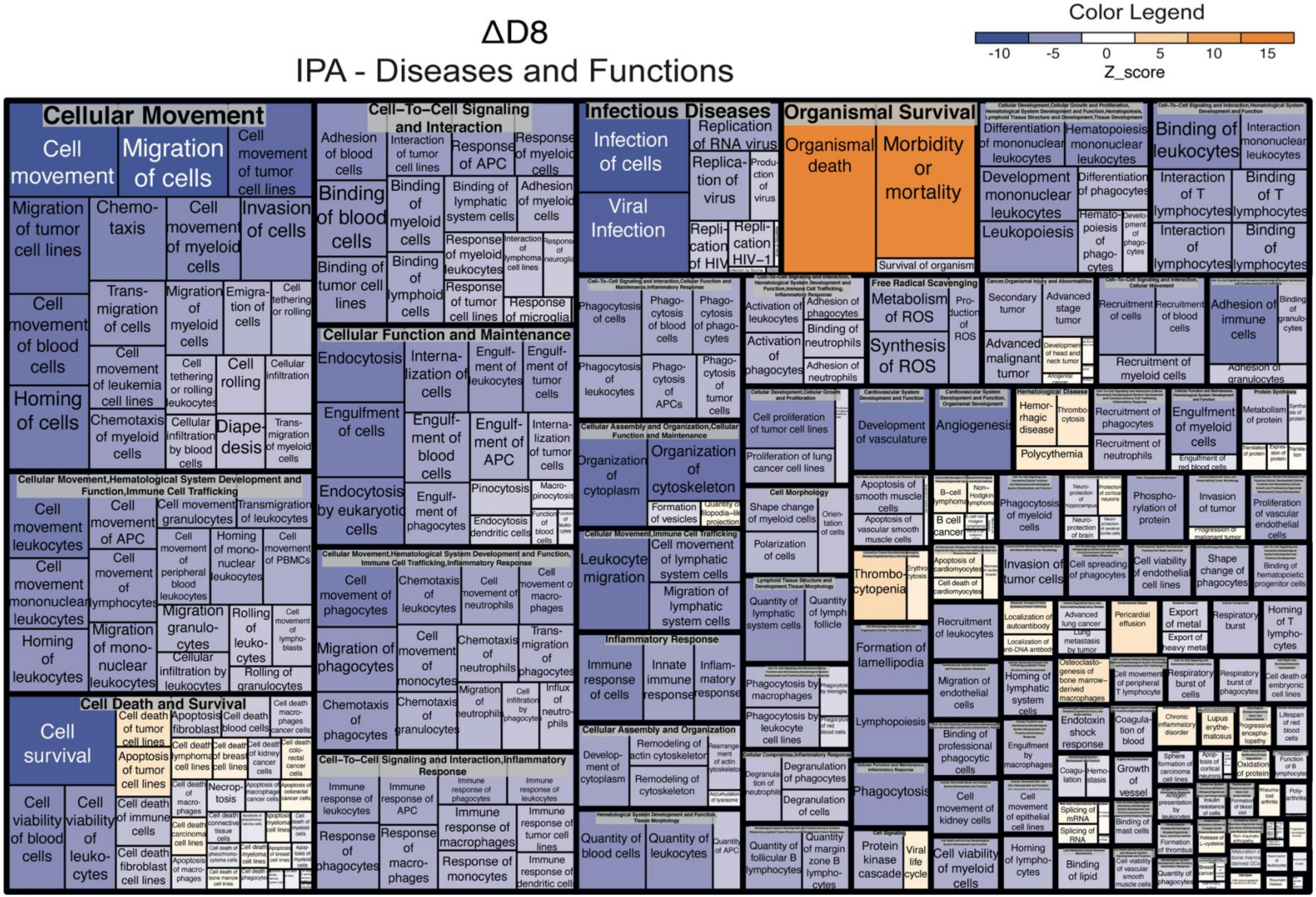

Supplementary figure 3: Treemap summarising altered IPA diseases and biological functions resulting from ΔD64 comparison. Each box represents a biological function or a disease and they are sized and coloured by their z-score (z-score > 0 in the orange colour scale or < 0 in the blue scale). Diseases and functions are clustered by IPA into master categories for which the name is displayed by a grey label. Data were analyzed through the use of IPA (QIAGEN Inc., https://www.qiagenbioinformatics.com/products/ingenuitypathway-analysis).

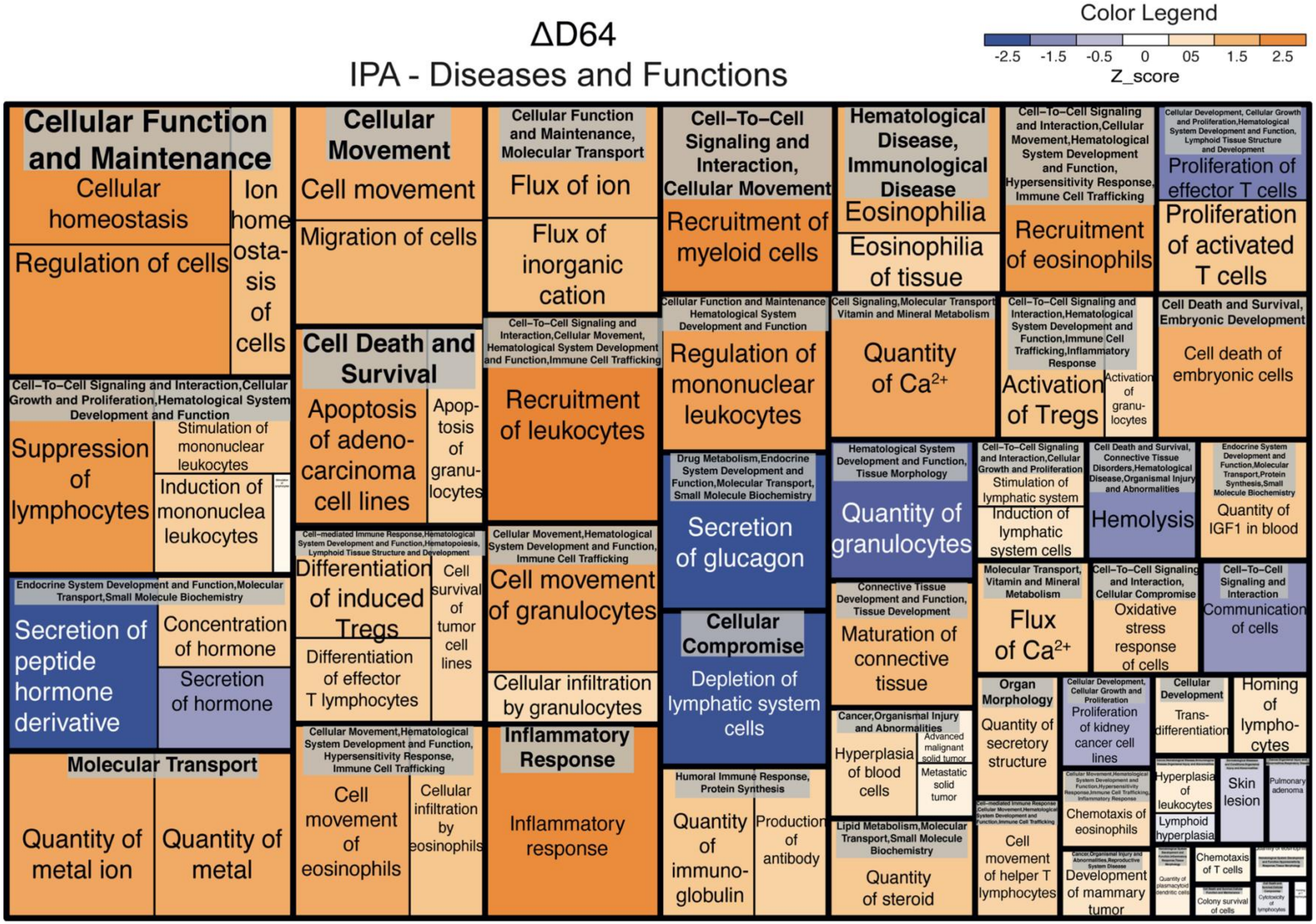

Supplementary figure 4: Bar plots showing downregulated (**A**) and upregulated (**B**) enriched Gene Ontology biological processes. Significance threshold lines are reported in black (-Log10 p-value=1.3). (Fisher exact statistical test was performed using Enrichr and enrichment -Log10 p-value is reported in the X-axis).

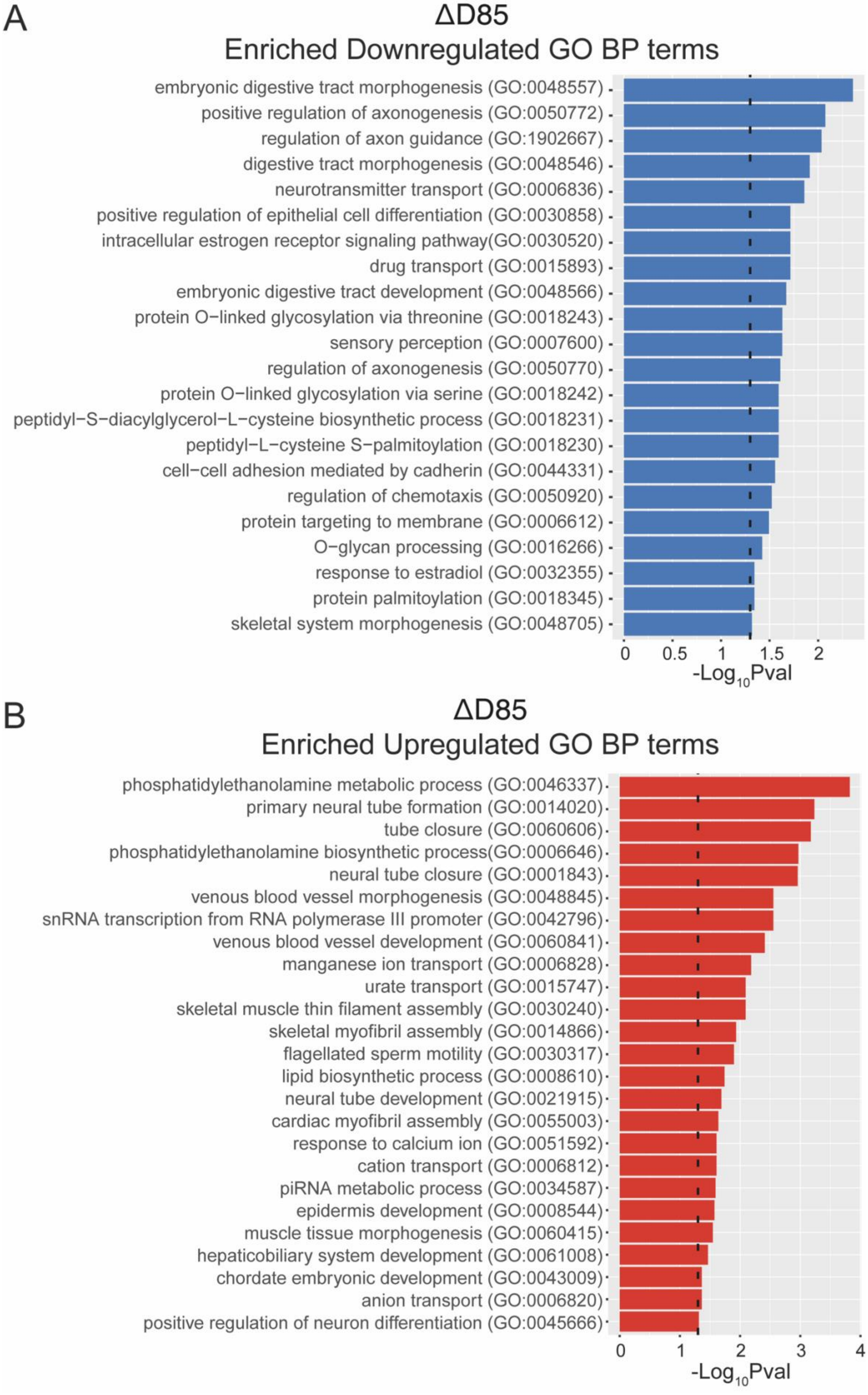

Supplementary table 1: This table shows significant (p<0.05) enriched canonical pathways from ΔD8 list of differentially expressed genes. This analysis was performed using IPA®. For each term, its Z-score, p-value are reported together with the ratio (number of genes in the input list over the total number included in the pathway) and gene names. Data were analyzed through the use of IPA (QIAGEN Inc., https://www.qiagenbioinformatics.com/products/ingenuitypathway-analysis)

Canonical pathway	Z-score	p val	Ratio	Genes
Protein ubiquitination pathway	NaN	6.55E-05	51/267	B2M, BRCA1, CDC23, DNAJB4, DNAJB5, DNAJB6, DNAJB11, DNAJB12, DNAJB14, DNAJC2, DNAJC7, DNAJC14, DNAJC17, HLA-A, HLA-C, HLA-E, HSP90AA1, HSP90AB1, HSP90B1, HSPA5, HSPA13, HSPA1A/HSPA1B, HSPB7, HSPD1, MDM2, PSMA4, PSMA8, PSMC1, PSMC2, PSMD1, PSMD2, PSMD4, PSMD7, PSMD10, PSMD11, PSMD12, SKP2, UBE2B, UBE2E2, UBE2E3, UBE2F, UBE2J2, UBE2L3, UBE2V1, UBE3B, UBE4B, UBR2, USP4, USP15, USP19, USP32
NRF2 mediated oxidative stress response	-2.982	1.10E-04	38/184	ABCC2, ACTG1, BACH1, DNAJA2, DNAJA4, DNAJB4, DNAJB5, DNAJB6, DNAJB11, DNAJB14, DNAJC7, DNAJC14, DNAJC17, ERAS, FKBP5, FTH1, FTL, GCLC, GSTA1, HACD3, HERPUD1, HMOX1, KRAS, MAFF, MAP3K1, NFE2L2, PRKCD, PRKCG, RAF1,, RALB, RAP1A, RASD1, SOD2, STIP1, TXNRD1, UBE2E3
Polyamine regulation in colon cancer	NaN	3.12E-04	9/22	APC, KRAS, MAX, MXD1, OAZ1, OAZ2, PSME3, SAT1, TCF4
Remodeling of epithelial adherens junctions	-2.449	4.34E-04	17/64	ACTG1, ACTN1, ACTR2, APC, ARPC2, ARPC5, CLIP1, CTNNA1, CTNND1, DNM3, MAPRE1, RAB5A, TUBA1A, TUBA1B, TUBA1C, TUBB2A
Iron homeostasis signaling pathway	NaN	8.79E-04	27/130	ACO1, ACO2, ARNT, ATP6V1A, ATP6V1B2, ATP6V1G1, EPO, FTH1, FTL, HAMP, HBA1/HBA2, HBB, HFE, HMOX1, IREB2, ISCU, JAK1, NFS1, NUBP1, PDGFRA, SKP2, SLC11A1, SLC25A37, SMAD1, STAT3, TFRC
ERK5 signaling	-2.84	1.54E-03	17/71	CREB1, ERAS, FOX3, GAB1, KRAS, MAP3K2, MEF2C, NTRK1, RALB, RAP1A, RSP6KA5, SGK1, WNK1, YWHAB, YWHAE, YWHAZ
Spliceosome cycle	-3.606	1.64E-03	13/48	BUD13, CASC3, CDC5L, CWC, 15, DDX23, DHX8, DHX38, MAGOHB, SF3A3, SF3B1, SF3B6, SLU7, U2AF1/U2AF1L
Integrin signaling	-3.651	1.65E-03	37/204	ACTG1, ACTN1, ACTR2, ARF1, ARF4, ARHGAP26, ARP2, ARPC5, CAPN5, CAPN11, ERAS, GRB2, ITGA4, ITGAX, ITGB8, KRAS,LIMS1, MYLK2, MYLK, NEDD9, PAK2, PLCG2, PP1CB, PP1R12B, PTEN, RAC2, RAF1, RALB, RAP1A, RASD1, RHOA, RHOB, SOS2, TSPAN1, TSPAN3, WIPF1
Fcγreceptor-mediated phagocytosis in macrophages and monocyte	-4.025	2.53E-03	20/93	ACTG1, ACTR2, ARPC2, ARPC5, EZR, FCGR2A, FYB1, GAB2, HCK, LCP2, LYN, MYO5A, PIP5K1A, PRKCD, PRKCG, PTEN, RAC2, SYK
Aldosterone signaling in epithelial cells	-2.714	2.55E-03	29/153	DNAJB4, DNAJB5, DNAJB6, DNAJB11, DNAJB14, DNAJC2, DNAJC7, DNAJC14, DNAJC17, HSP90AA1, HSP90AB1, HSP90B1, HSPA5, HSPA13, HSPA1A/HSPA1B, HSPB7, HSPD1, KRAS, PDIA3, PIP5K1A, PLCB3, PLCG2, PLCL1, PRKCD, PRKCG, RAF1, SGK1,SOS2
Cancer drug resistance by drug efflux	NaN	3.02E-03	14/57	ABCC2, ERAS, FOXO3, KRAS, MDM2, mir-133, mir-154, PTEN, PTGS2, RAF1, RALB, RAP1A, RASD1, YBX1
Role of JAK1, JAK2 and TYK2 in interferone signaling	-1.633	3.16E-03	8/24	CGA, IFNAR1, IFNGR1, INFGR2, JAK1, PTPN6, RAF1, STAT3

Actin nucleation by ARP-WASP complex	-2.714	3.47E-03	16/70	ACTR2, ARPC2, ARPC5, ERAS, GRB2, ITGA4, KRAS, PPP1R12B, RAC2, RALB, RAP1A, RASD1, RHOA, RHOB, SOS2, WIPF1
Actin cytoskeleton signaling	-3.722	3.55E-03	37/213	ACTG1, ACTN1, ACTR2, APC2, APC, ARPC2, ARPC5, CD14, CRKL, ERAS, EZR, F2R, GNG12,GRB2, ITGA4, KRAS, LIMK2, MSN, MYH14, MYLK2, MYLK, NCKAP1L, PAK2, PIP5K1A, PPP1CB, PPP1R12B, RAC2, RAF1, RALB, RAP1A, RASD1, RHOA, SOS2, SSH1, SSH3, TRIO, WASF2
Phagosome maturation	NaN	4.49E-03	26/138	ATP6V1A, ATP6V1B2, ATP6V1G1, ATP6V1H, B2M, CTSB, CTSC, CTSZ, CYBB, DYNC1LI1, DYNLL1, HLA-A, HLA-C, HLA-E, M6PR, NAPB, NCF2, RAB5A, TSG101, TUBA1A, TUBA1B
Natural killer cell signaling	-2.121	5.30E-03	32/182	B2M, COL1A2, ERAS, FCGR2A, GRB2, HLA-A, HLA-C, HLA-E, HLA-G, HSA1A/HSPA1B, KRAS, LCP2, LIMK2, MAP3K1, MAP3K2, MICB, MTOR, PAK2, PLCG2, PTPN6, RAC2, RAF1, RALB, RAP1A, RASD1, SOS2, SYK, TNFSF10, TYROBP, WIPF1
Systemic lupus erythematosus signaling	NaN	6.40E-03	32/206	CD86, ERAS, FCGR2A, FCGR2C, GRB2, HLA-A, HLA-C, HLA-E, HLA-G, HNRNPA2B1, IL1B, IL1RN, KRAS, LSM3, LSM6, LSM12, LSM14A, LYN, MTOR, NFACT4, PLCG2, PRPF4, PRPF6, PRPF38A, PTPN6, RALB, RAP1A, RASD1, RNU12, RNU5F-1, SNRPB, SNRPD3, SNRPF, SOS2, TLR7
Neuregulin signaling	-2.309	6.50E-03	19/94	ADAM17, CRKL, ERA, GRB2, HSP90AA1, HSP90AB1, HSP90B1, ITGA4, KRAS, MTOR, PLCG2, PRKCD, PRKCG, PTEN, RAF1 RALB, RAP1A, RASD1, SOS2
p70S6K signaling	-3.9	6.55E-03	24/128	BTK, EEF2K, ERAS, F2R, GRB2, JAK1, KRAS, LYN, MTOR, PDIA3, PLCB3, PLCG2, PLC1, PRKCD, PRKCG, RAF1, RALB, RAP1A, RASD1, SOS2, SYK, YWHAB, YWHAE, YWHAZ
Pathogenesis of multiple sclerosis	NaN	7.20E-03	4/8	CCL5, CCR1, CXCL9, CXCCR3
Glioma signaling	-2.357	7.72E-03	21/109	CAMK1D, CAMK1G, CAMK2A, ERAS, GRB2, IGF1, IGF1R, IGF2R, KRAS, MDM2, MTOR, PDGFRA, PLCG2, PRKCD, PRKCG, PTEN, RAF1, RALB, RAP1A, RASD1, SOS2
Chemokine signaling	-1.604	8.03E-03	16/76	CAMK1D, CAMK1G, CAMK2A, CCL5, ERAS, KRAS, LIMK2, PLCB3, PLPCG2, PPP1CB, PPP1R12B, RAF1, RALB, RAP1A, RASD1, RHOA
HGF signaling	-3.357	8.57E-03	21/110	CRKL, ELF1, ELF3, ELK3, ERAS, GAB1, GRB2, ITGA4, KRAS, MAP3K1, MAP3K2, PLCG2, PRKCD, PRKCG, PTGS2, RAF1, RALB, RAP1A, RASD1, SOS2, STAT3
14-3-3 mediated signaling	-3.5	9.62E-03	23/125	ERAS, GRB2, KRAS, PDIA3, PLCB3, PLCG2, PLCL1, PRKCD, PRKCG, RAF1,RALB, RAP1A, RASD1, SRPK2, TNFRSF1A, TUBA1A, TUBA1B, TUBA1C, TUBB2A, VIM, YWHAB, YWHAE, YWHAZ
Hypoxia signaling in the cardiovascular system	-1	1.11E-02	15/75	ARNT, CREB1, EPO, HSP90AA1, HSP90AB1, HSP90B1, MDM2, PTEN, UBE2B, UBE2E2, UBE2E3, UBE2F, UBE2J2, UBE2L3, UBE2V1
Rapoport-Luebering glycolytic shunt	NaN	1.13E-02	3/5	BPGM, PGAM2, TIGAR
Tumoricidal function of hepatic natrual killer cells	-1	1.26E-02	7/24	BID, CASP8, DFFA, ICAM1, LYVE1, M6PR, SRGN
PPAR signaling	3.638	1.40E-02	19/101	ERAS, GRB2, HSP90AA1, HSP90AB1, HSP90B1, IL1B, IL1R2, IL1RN, KRAS, MAP4K4, NCOA1, PDGFRA, PTGS2, RAF1, RALB, RAP1A, RASD1, SOS2, TNFRSF1A
fMLP signaling in neutrophils	-2.673	1.41E-02	21/115	ACTR2, ARPC2, ARPC5, CYBB, ERAS, FPR1, FPR2, GNB4, GNG7, GNG10, GNG12, KRAS, NCF2, NFATC4, PLCB3, PRKCD, PRKCG, RAF1, RALB, RAP1A, RASD1
PI3K/AKT signaling	-3.3	1.46E-02	29/173	CXCR2, ERAS, FOXO3, GAB1, GAB2, GRB2, HSP90AA1, HSP90AB1, HSP90B1, IFNAR1, IL1R2, IL22RA2, ITGA4, JAK1, KRAS, LIMMS1, MCL1, MDM2, MTOR, PTEN, PTGS2, RAF1, RALB, RAP1A,RASD1, SOS2, YWHAB, YWHAZ

Systemic lupus erythematosus in B cell signaling pathway	-2.03	1.46E-02	41/263	BCL2L11, BTK, ERAS, FCGR2A, FCR2C, FOX0O3, GAB1, GRB2, HCK, IFNAR1, IFNGR1, IFNGR2, IL1B,JAK1, KRAS, LILRA6, LILRB3, LYN, MAP4K4, MCL1, MTOR, NFACT4, PLCG2, PRKCD, PRKCG, PTPN6, RAC2, RAF1, RALB, RAP1A, RASD1, SHC3, SOS2, STAT3, SYK, TBK1, TLR7, TNFSF4, TNFSF10, TNFSF14, TRAF3
Prolactin signaling	-1.941	1.47E-02	16/81	ERAS, GRB2, KRAS, NR3C1, PLCG2, PRKCD, PRKCG, PRL, RAF1, RALB, RAP1A, RASD1, SOC4, SOS2, SP1, STAT3
Phospholipase C signaling	-4.017	1.80E-02	37/236	ARHGEF9, BTK, CREB1, ERAS, FCGR2A, FCGR2C, GNB4, GNB7, GNG10, GNG12, GRB2, HDAC7, HDAC9, HMOX1, ITGA4, KRAS, LCP2, LYN, MEF2C, NFACT4, PLA2G12A, PLA2G4C, PLCB3, PLCG2, PPP1CB, PPP1R12B, PRKCD, RAC2, RAF1, RALB, RAP1A, RASD1, RHOA, RHOB, SOS2, SYK
CCR3 signaling in eosinophils	-2.53	1.85E-02	21/118	ERAS, GNB4, GNG7, GNG10, GNG12, KRAS, LIMK2, MYLK, PAK2, PLA2G12A, PLA2G4C, PLCB3, PPP1CB, PPP1R12B, PRKCD, PRKCG, RAF1, RALB, RAP1A, RASD1, RHOA
IGF-1 signaling	-2.887	1.88E-02	19/104	ERAS, FOXO3, GRB2, IGF1, IGF1R, IGFBP4, JAK1, KRAS, PRKAR1A, RAF1, RALB, RAP1A, RASD1, SOCS4, SOS2, STAT3, YWHAB, YWHAE, YWHAZ
Oxidative ethanol degradation III	-2.236	1.90E-02	5/15	ACSL1, ACSS3, ALDH3A1, ALDH3A2, ALDH9A1
TREM1 signaling	-3.742	1.95E-02	14/70	CD86, GRB2, ICAM1, IL1B, ITGAX, LAT2, PLCG2, STAT3, TLR2, TLR4, TLR5, TLR7,, TREM1, TYROBP
Role of JAK2 in hormone-like cytokine signaling	NaN	2.04E-02	8/32	EPO, JAK1, PRL, PTPN6, SH2B3, SIRPA, SOCS4, STAT3
Phagosome formation	NaN	2.04E-02	20/112	CLEC7A, FCAMR, FCGR2A, FCGR2C, ITGA4, ITGAX, PDIA3, PLCB3, PLCG2, PLCL1, PRKCD, PRKCG, RAC2, RHOA, RHOB, SYK, TLR2, TLR4, TLR, TLR7
Ephrin receptor signaling	-4.796	2.09E-02	29/178	ACTR2, ADAM10, ARPC2, ARPC5, CREB1, CRKL, EPHA8, EPHB1, ERAS, GNB4, GNG7, GNG10, GNG12, GRB2, ITGA4, KRAS, LIMK2, MAPK4, PAK2, RAC2, RAF1, RALB, RAP1A,RASD1, RHOA, SDCBP, SOS2, STAT3, WIPF1
NF-kB signaling	-4.2	2.16E-02	28/171	CASP8, ERAS, IGF1R, IGF2R, IL1B, IL1R2, IL1RN, KRAS, LTBR, MAP3K1, MAP4K4, NTRK1, PDGFRA, PLCG2, RAF1, RALB, RAP1A, RASD1, TBK1, TDP2, TLR2, TLR4, TLR5, TLR7, TNFRSF1A, TNIP1, TRAF3, UBE2V1
Epithelial adherens junction signaling	NaN	2.19E-02	25/149	ACTG1, ACTN1, ACTR2, APC, ARPC2, ARPC5, CLPI1, CTNNA1, CTNND1, ERAS, FARP2, KRAS, MYH14, NOTCH2, PTEN, RALB, RAP1A, RASD1, RHOA, SSX21P, TCF4, TUBA1A, TUBA1B, TUBB2A
Estrogen receptor signaling	-4.824	2.45E-02	47/319	CACNA1E, CREB1, DDX5, ERAS, FOXO3, GNG7, GRB2, HSP90AA1, HSP90AB1, HSP90B1, IGF1, IG1FR, IG2R, JAK1, KRAS, LIMK2, MED18, MED23, MED30, MED31, MMP14, MTOR, NCOA1, NR3C1, PDIA3, PLCB3, PLCG2, PLCL1, PPP1CB, PPP1R12B, PRKAA1, PRKAR1A, PRKCD, PRKCG, PTNE, RAF1, RALB, RAP1A, RASD1, RHOA, SDHD, SHC3, SOD2, SOS2, SP1, THRAP3, UQCRC2
ERK/MAPK signaling	-3.53	2.48E-02	30/188	CREB1, CRKL, ELF1, ELF3, ELK3, ERAS, GRB2, H3-3A/H3-3B, HSPB7, ITGA4, KRAS, MKNK1, PAK2, PLA2G12A, PLA2G4C, PLCG2, PPP1CB, PRKAR1A, PRKCD, PRKCG, RAC2, RAF1, RALB, RAP1A, RASD1, RSP6KA5, SOS2, STAT3, YWHAB, YWHAZ
PDGF signaling	-3.207	2.51E-02	16/86	CAV3, CRKL, ERAS, GRB2, JAK1, KRAS, MAP3K1, PDGFRA, PLCG2, RAF1, RALB, RASD1, SOS2, SPHK2, STAT3
Fatty acid alpha-oxidation	-2.236	2.52E-02	5/16	ALDH3A1, ALDH3A2, ALDH9A1, PTGS2, TMLHE
Alpha-adrenergic signaling	-2.53	2.76E-02	17/94	ERAS, GNB4, GNG7, GNG10, GNG12, KRAS, PHKB, PLCG2, PRKAR1A, PRKCD, PRKCG, RAF1, RAP1A, RASD1, SLC8A1, SLC8A3
FAK signaling	NaN	2.76E-02	17/94	ACTG1, ARHGAP26, CAPN5, CAPN11, ERAS, GIT2, GRB2, ITGA4, KRAS, PAK2, PLCG2, PTEN, RAF1, RALB, RAP1A, RASD1, SOS2

Mechanism of viral exit from host cells	NaN	2.77E-02	9/40	ACTG1, CHMP3, CHMP6, CHMP2A, CHMP4B, PRKCD, PRKCG, TSG101, VSP25
Autophagy	NaN	2.81E-02	11/53	ATG3, ATG7, BECN1, CTSB, CTSC, CTSZ, MAP1LC3A, MAP1LC3A, MTOR, VSP41, WDFY3
Cytotoxic T lymphocyte-mediated apoptosis of target cells	NaN	2.92E-02	7/28	B2M, BID, CASP8, DFFA, HLA-A, HLA-C, HLA-E
Endometrial cancer signaling	-1.414	2.93E-02	12/60	APC2, CTNNA1, ERAS, FOXO3, GRB2, KRAS, PTEN, RAF1, RALB, RAP1A, RASD1, SOS2
Sirtuin signaling pathway	-2.335	3.17E-02	41/277	ADAM10, APP, ARNTL, ATG3, ATG7, ATG13, ATG14, ATG2B, ATP5PF, BCL2L11, BECN1, BPGM, CPT1A, EPO, FOXO3, H1-6, H3-3A/H3-3B, MAP1LC3A, MTOR, NAMPT, NDUFA7, NDUFA8, NDUFA8, NDUFS1, NDUFS3, NFE2L2, PCK1, PGAM2, POLR1A, PPIF, PRKAA1, SDHD, SOD2, SP1, STAT3, TUBA1A, TUBA1B, TUBA1C, UQCRC2, VDAC1, VDAC2, XRCC5
SPINK1 general cancer pathway	-0.632	3.29E-02	12/61	ERAS, JAK1, KRAS, MT1A, MT1B, MT1F, MT1X, RAF1, RALB, RAP1A, RASD1, STAT3
L-carnitine biosynthesis	NaN	3.39E-02	2/3	ALDH9A1, TMLHE
PPARα/RXRα activation	1.528	3.45E-02	28/178	ACOX1, ADIPOR1, APOA1, APOA2, ERAS, GK, GPD1, GRB2, HSP90AA1, HSP90AB1, HSP90B1, IL1B, IL1R2, KRAS, MAP4J4, MED23, PDAI3, PLCB3, PLCG2, PLCL1, PRKAA1, PRKAR1A, RAF1, RALB, RAP1A, RASD1, SOS2
HIFα signaling	-2.335	3.47E-02	31/201	ADM, ARNT, CAMK1D, CAMK1G, CAMK2A, CYBB, EPO, ERAS, FOXP3, HMOX1, HSP90AA1, HSPA5, HSP1A/HSPA1B, IGF1, KRAS, MDM2, MKNK1, MMP14, MTOR, NCF2, NCOA1, PLCG2, PRKCD, PRKCG, RAF1, RALB, RAP1A, SAT1, STAT3, VIM
Cleavage and polyadenilation of pre-mRNA	NaN	3.55E-02	4/12	CPSF4, CSTF1, NUDT21, WDR33
Telomerase signaling	-3.464	3.56E-02	18/104	ELF1, ELF3, ERAS, GRB2, HDAC7, HDAC9, HSP90AA1, HSP90AB1, HSP90B1, KRAS, RAF1, RALB, RAP1A, RASD1, SOS2, SP1, TERF2IP
BAG2 signaling pathway	-1.667	3.71E-02	9/42	ANXA2, ATXN3, CTS8, HSP90AA1, HSPA5, HSPA1A/HSPA1B, MDM2, PSME3, SP1
IL-10 signaling	NaN	3.73E-02	13/69	BLVRA, CCR1, CD14, FCGR2A, FCGR2C, HMOX1, IL1B, IL1R2, IL1RN, JAK1, MAP4K4, SP1, STAT3
Role of NFAT in cardiac hypertrophy	-3.266	3.74E-02	32/210	CACNA1E, CAM1KD, CAMK1G, CAMK2A, ERAS, GNB4, GNG7, GNG10, GNG12, GRB2, HDAC7, HDAC9, IGF1, IGF1R, KRAS, MAP3K1, NFATC4, PDAI3, PLCB3, PLCG2, PLCL1M PRKAR1A, PRKCD, RAF1, RALB, RAP1A, RASD1, SLC8A1, SLC8A3, SOS2
Macropinocytosis	-2.212	3.74E-02	14/76	ANKFY1, CD14, CSF1R, ERAS, ITGB8, KRAS, PLCG2, PRKCD, PRKCG, RAB5A, RALB, RAP1A, RASD1, RHOA,
Cell cycle: G2/M DNA checkpoint regulation	-0.333	3.92E-02	10/49	BRCA1, CDC25C, CKS2, MDM2, PPM1D, SKP2, TRIP12, YWHAB, YWHAE, YWHAZ
CNTF signaling	-3	4.05E-02	11/56	ERAS, GRB2, JAK1, KRAS, MTOR, RAF1, RALB, RAP1A, RASD1, RSP6KA5, STAT3
Thrombopoietin signaling	-2.53	4.11E-02	12/63	ERAS, GAB2, GRB2, KRAS, PLCG2, PRKCD, PRKCG, RAF1, RALB, RAP1A, RASD1, STAT3

Virus entry via endocytic pathways	NaN	4.19E-02	18/106	ACTG1, AP1G1, B2M, ERAS, HLA-A, HLA-C, HLA-C, HLA-E, ITGA4, ITGB8, KRAS, PLCG2, PRKCD, PRKCG, RAC2, RALB, RAP1A, RASD1, TFRC
Sperm motility	-3.051	4.19E-02	32/212	ABHD3, BTK, CLK2, CLK3, CSF1R, DYRK4, DYRK1A, EPHA8, EPHB1,HCK, IGF1R, JAK1, LMTK2, LYN, MERTK, NTRK1, PDE4B, PDGFRA, PDIA3, PEAK1, PLA2G12A, PLA2G4C, PLB1, PLCB3, PLCG2, PLCL1, PRKAR1A, PRKCD, PRKCG, PRKG1, SYK, ZAN
TCA cycle II (eukaryotic)	-2.449	4.23E-02	6/24	ACO1, ACO2, DLD, IDH3A, SDHD, SUCLG1
Oncostatin M signaling	-2.646	4.25E-02	9/43	ERAS, GRB2, JAK1, KRAS, RAF1, RALB, RAP1A, RASD1, STAT3
Fc epsilon RI signaling	-3.638	4.41E-02	19/114	BTK, ERAS, GAB1, GRB2, KRAS, LCP2, LYN, PLA2G12A, PLA2G4C, PRKCD, PRKCG, RAC2, RAF1,, RALB, RAP1A, RASD1, SOS2, SYK
Myc mediated apoptosis	-1.265	4.43E-02	10/50	BCL2L11, BID, CASP8, CFLAR, CRADD, MAX, MCL1, MDM2, PRKAR1A, TNFRSF1A
Cdc42 signaling	-1.807	4.61E-02	20/122	ACTR2, APC2, APC, ARPC2, B2M, CLIP1, FNBP1L, HLA-A, HLA-C, HLA-E, HLA-G, ITGA4, LIMK2, MYLK, PAK2, PPP1CB, RAF1, WIPF1
Histamine degradation	-2	4.69E-02	4/13	ALDH3A1, ALDH3A2, ALDH9A1, HNMT
Salvage pathways of pyrimidine deocyribonucleotides	NaN	4.92E-02	3/8	APOBEC3A, APOBEC3B, CDA
IL-3 signaling	-2.309	4.97E-02	14/79	CRKL, ERAS, GAB2, GRB2, JAK1, KRAS, PRKCD, PRKCG, PTPN6, RAF1, RALB, RAP1A, RASD1, STAT3

Supplementary table 2: This table shows significant (p<0.05) enriched canonical pathways from ΔD64 list of differentially expressed genes. This analysis was performed using IPA®. For each term, its Z-score, p-value are reported together with the ratio (number of genes in the input list over the total number included in the pathway) and gene names. Data were analyzed through the use of IPA (QIAGEN Inc., https://www.qiagenbioinformatics.com/products/ingenuitypathway-analysis)

Canonical pathway	Z score	Pvalue	Ratio	Genes
Breast Cancer Regulation by Stathmin1	NaN	2.25E-03	22/573	ADGRE1, ADGRL3, CCR3, CYSLTR2, FFAR4, GHRHR, GNG4, GPER1, GPR34, GPR82, GPR146, GPR183, HCAR1, LGR5, mir-31, NPY4R/NPY4R2, P2RY10, P2RY14, PIK3R6, PTGDR2, RXFP2
Tryptophan degradation to 2-amino-3carboxymuconate semialdhehyde	NaN	5.74E-03	2/6	HAAO/IDO1
NAD biosynthesis II (from tryptophan)	NaN	1.88E-03	2/11	HAAO/IDO1
Sorbitol degradation	NaN	1.96E-02	1/1	SORD
Heparan Sulfate Biosynthesis (Late stages)	0	2.55E-02	4/57	CHST13, HS3ST5, HS3ST6, PNPLA7
Maturity onset diabetes of young (MODY) signaling	NaN	2.70E-02	4/58	APOB, APOC1, CACNA1E, PKLR
Atherosclerosis signaling	NaN	3.22E-02	6/121	ALOX15, APOB, APOC1, CCR3, CSF1, PLAAT5
Heparan Sulfate Biosynthesis	0	3.70E-02	4/64	CHST13, HS3ST5, HS3ST6, PNPLA7
Th1 and Th2 activation pathway	NaN	3.88E-02	7/160	CCR3, IL9, IL1RL1, IL2RA, KLRC1, PIK3R6, PTGDR2
Choline degradation I	NaN	3.89E-02	1/2	ALDH7A1

Sulfate activation for sulfonation	NaN	3.89E-02	1/2	PAPSS1
Glycine degradation (Creatine Biosynthesis)	NaN	3.89E-02	1/3	GATM
Th2 pathway	2.236	3.94E-02	6/127	CCR3, IL9, IL1RL1, IL2RA, KLRC1, PIK3R6, PTGDR2
Iron homeostasis pathway	NaN	4.33E-02	6/130	ATP6V0A2, ATP6VOD2, ATP6V1A, HFE, SLC46A1, SMAD5
Serotonin receptor signaling	Nan	4.62E-02	3/41	HTR3A, HTR3C, SPR
Dermatan sulfate biosynthesis (late stages)	NaN	4.91E-02	3/42	CHST13, HS3ST5, HS3ST6

Supplementary table 3: Table illustrates significantly (p-value<0.05) enriched diseases and functions from ΔD8 differentially expressed gene list analysis using IPA®. For each disease and function term the cluster category is reported together with p-value, z-score, predicted activation state (z-score>2= increased activation, z-score<-2=decreased activation), number of transcripts and their IDs. Data were analyzed through the use of IPA (QIAGEN Inc., https://www.qiagenbioinformatics.com/products/ingenuitypathway-analysis)

Categories	Diseases or Functions Annotation	p-value	Predicted Activation State	Activation z-score	# Genes	Genes
Cellular Compromise, Inflammatory Response	Degranulation of phagocytes	1.90E-10	Decreased	-2.36	124	ACP3, ACTR2, ADAM10, ADGRE2, ADGRG3, ANXA2, ANXA3, APP, ARMC8, ARPC5, ASAH1, ATG7, ATP6AP2, B2M, B4GALT1, BRI3, BTK, CAP1, CCT2, CD14, CD84, CD93, CDA, CEACAM3, CLEC4D, CPPED1, CTLA4, CTSB, CTSC, CTSZ, CXCL1, CXCR2, CYBB, CYSTM1, DDX3X, DEFB103A/DEFB103B, DHCR7, DOCK2, DYNC1LI1, DYNLL1, FCGR2A, FGL2, FPR1, FPR2, FTH1, FTL, GAB2, GDI2, GLA, GLIPR1, GMFG, GYG1, HBB, HCK, HLA-C, HMOX1, HSP90AA1, HSP90AB1, HSPA1A/HSPA1B, HVCN1, IGF2R, IL1B, IRAG2, ITGAX, KCNAB2, LAT2, LCP2, LILRB3, LYN, LYZ, MAGT1, MCEMP1, METTL7A, MLEC, MVP, MYO1F, NCKAP1L, OSTF1, P2RX1, PAK2, PDAP1, PECAM1, PF4, PIGA, PLCG2, PLEKHO2, PRKCD, PSAP, PSMC2, PSMD1, PSMD11, PSMD12, PSMD2, PSMD7, PTEN, PTPN6, RAB27A, RAB31, RAC2, RAP1A, RHOA, RNASET2, S100A9, SDCBP, SERPINB3, SH3BP2, SIGLEC9, SIRPA, SIRPB1, SLC11A1, SNAP23, SNAP29, SPHK2, SURF4, SWAP70, SYK, TLR2, TREM1, TREML2, TYROBP, WIPF1, XRCC5, YPEL5, ZEB2
Cellular Function and Maintenance	Endocytosis	3.33E-10	Decreased	-5.945	151	ACTG1, ACTR2, AMPH, ANKFY1, ANXA5, APC, APLP2, APOA1, APOA2, APOB, APOL1, APP, APPL2, ARF1, ARHGAP27, ARPC2, ATG2B, ATG7, ATP6V1A, ATP6V1B2, ATP6V1H, B2M, BECN1, BTK, CAP1, CARMIL1, CAV3, CCL5, CD14, CD93, CDC5L, CEACAM3, CLCN3, CLEC4M, CLEC6A, CLEC9A, CLIC4, CLIP1, CORO1C, CSF1R, CTNND1, CYBB, DAB2, DDX3X, DEF6, DET1, DNM3, DOCK2, DPYSL2, EEF2K, ENTPD1, EZR, FCAMR, FCGR2A, FGD4, FNBP1L, FPR1, FPR2, FRS2, GAB2, GRB2, HBA1/HBA2, HBB, HCK, HFE, HMOX1, HSP90AA1, HSP90B1, HSPA5, ICAM1, IFNAR1, IGF1R, IGF2R, IL1B, IRF8, JAK1, KAT6A, KCTD5, KRAS, let-7, LRP2, LRP8, LRPAP1, LYN, M6PR, MAPKAPK3, MERTK, MEX3B, mir-24, MS4A4A, MYLK, MYO5A, NCKAP1L, NCL, NLGN3, NR3C1, NTRK1, PACSIN2, PARK7, PDZD8, PF4, PIP5K1A, PLEK, PRKCD, PSMD4, PTEN, PTPN6, RAB11A, RAB21, RAB22A, RAB31, RAB4A, RAB5A, RAC2, RALB, RALBP1, RGCC, RHOA, RHOB, RIT1, RUFY1, S100A9, SCARB2, SCRIB, SH3BP2, SIRPA, SIRPB1, SLAMF7, SNAP23, SNAP91, SORL1, SRSF3, STK4, SWAP70, SYK, TFRC, TLR2, TLR4, TM2D2, TNFRSF1A, TNFSF10, TREM1, TREML2, TYROBP, UBE2L3, VIM, WASF2, WIPF1, WNK1, ZDHHC17, ZNF217
Cellular Compromise, Inflammatory Response	Degranulation of neutrophils	4.17E-10	Decreased	-2.433	102	ACP3, ACTR2, ADAM10, ADGRG3, ANXA2, ANXA3, ARMC8, ARPC5, ASAH1, ATG7, ATP6AP2, B2M, B4GALT1, BRI3, CAP1, CCT2, CD14, CD93, CDA, CEACAM3, CLEC4D, CPPED1, CTSB, CTSC, CTSZ, CXCL1, CXCR2, CYBB, CYSTM1, DDX3X, DOCK2, DYNC1LI1, DYNLL1, FCGR2A, FGL2, FPR1, FPR2, FTH1, FTL, GDI2, GLA, GLIPR1, GMFG, GYG1, HBB, HCK, HLA-C, HSP90AA1, HSP90AB1, HSPA1A/HSPA1B, HVCN1, IGF2R, IRAG2, ITGAX, KCNAB2, LILRB3, LYZ, MAGT1, MCEMP1, METTL7A, MLEC, MVP, MYO1F, NCKAP1L, OSTF1, P2RX1, PDAP1, PECAM1, PF4, PLCG2, PLEKHO2, PRKCD, PSAP, PSMC2, PSMD1, PSMD11, PSMD12, PSMD2, PSMD7, PTPN6, RAB27A, RAB31, RAP1A, RHOA, RNASET2, S100A9, SDCBP, SERPINB3, SIGLEC9, SIRPA, SIRPB1, SLC11A1, SNAP23, SNAP29, SURF4, SYK, TLR2, TREM1, TREML2, TYROBP, XRCC5, YPEL5
Cellular Compromise, Inflammatory Response	Degranulation of cells	1.99E-09	Decreased	-2.032	146	ACP3, ACTN1, ACTR2, ADAM10, ADGRE2, ADGRG3, ANXA2, ANXA3, ANXA5, APLP2, APOA1, APP, ARMC8, ARPC5, ASAH1, ATG7, ATP6AP2, B2M, B4GALT1, BRI3, BTK, CAP1, CCR1, CCT2, CD14, CD84, CD93, CDA, CEACAM3, CLEC4D, CPPED1, CTLA4, CTSB, CTSC, CTSZ, CX3CR1, CXCL1, CXCR2, CYBB, CYSTM1, DDX3X, DEFB103A/DEFB103B, DHCR7, DOCK2, DUSP5, DYNC1LI1, DYNLL1, ENTPD1, F13A1, F8, FCGR2A, FGL2, FPR1, FPR2, FTH1, FTL, GAB2, GDI2, GLA, GLIPR1, GMFG, GYG1, HBB, HCK, HLA-C, HMOX1, HSP90AA1, HSP90AB1, HSPA1A/HSPA1B, HVCN1, IGF1, IGF2R, IL1B, IRAG2, ITGAX, KCNAB2, LAT2, LCP2, LHFPL2, LILRB3, LYN, LYZ, MAGT1, MCEMP1, METTL7A, MLEC, MMRN1, MVP, MYO1F, NCKAP1L, OSTF1, P2RX1, PAK2, PDAP1, PECAM1, PF4, PIGA, PLCG2, PLEK, PLEKHO2, POTEKP, PRKCD, PSAP, PSMC2, PSMD1, PSMD11, PSMD12, PSMD2, PSMD7, PTEN, PTPN6, RAB27A, RAB31, RAC2, RALB, RAP1A, RHOA, RICTOR, RNASET2, S100A9, SCG3, SDCBP, SERPINB3, SGK1, SH3BP2, SIGLEC9, SIRPA, SIRPB1, SLC11A1, SNAP23, SNAP29, SPHK2, SRGN, SURF4, SWAP70, SYK, TAGLN2, TLR2, TREM1, TREML2, TYROBP, VTI1B, WIPF1, XRCC5, YPEL5, ZEB2
Cellular Movement	Cell movement	1.00E-08	Decreased	-9.182	493	ACO2, ACTG1, ACTN1, ACTR2, ADAM10, ADAM15, ADAM17, ADGRE2, ADGRG3, ADIPOR1, ADM, AGO2, AIF1, AKAP12, ALKBH1, ALOX5AP, ANGPTL4, ANO5, ANXA2, ANXA3, APBA1, APBB1IP, APC, APLP2, APOA1, APOB, APP, APPL2, AQP9, ARF1, ARF4, ARHGAP19, ARHGAP25, ARHGDIB, ARNT, ARPC2, ARRDC3, ATAT1, ATG3, ATG7, ATOX1, ATRN, B4GALT1, BARHL1, BARX2, BCAS3, BECN1, BGN, BID, BRCA1, BTG2, BTK, CACNA1E, CALML3, CALU, CAMK1D, CAP1, CARMIL1, CASP8, CATSPERD, CAV3, CAVIN2, CCDC40, CCDC88A, CCL23, CCL5, CCNYL1, CCR1, CCR10, CD14, CD84, CD86, CD93, CDKL5, CEACAM3, CELSR3, CGA, CGB3 (includes others), CHD4, CHST1, CLASP1, CLCA2, CLCN3, CLEC1B, CLEC4M, CLEC7A, CLIC4, CLIP1, CNP, CNR1, COL1A2, COL7A1, CORO1C, CPEB1, CREB1, CRKL, CSF1R, CSF3R, CTLA4, CTNNA1, CTNND1, CTNND2, CTSB, CTSC, CTSZ, CUX1, CX3CR1, CXCL1, CXCL16, CXCL6, CXCL9, CXCR2, CXCR3, CYBB, CYP26B1, CYP2J2, DAB2, DDX3X, DEF6, DEFB103A/DEFB103B, DEFB114, DKK3, DNAJB4, DNAJB6,

						DOCK2, DOCK8, DPP10-AS1, DPYSL2, DSE, DUSP3, DUSP5, EFS, EIF3A, ELF3, ELK3, ELN, EMC10, ENTPD1, EPB41L5, EPHA8, EPHB1, EPO, EYA3, EZR, F10, F11R, F13A1, F2R, FAIM2, FBLN2, FCAMR, FCGR2A, FFAR4, FGD4, FGL2, FNBP1L, FOXO3, FOXP3, FPR1, FPR2, FRS2, FTH1, FTX, FUT7, FYB1, FZD3, GAB1, GAB2, GAL3ST1, GALNT1, GAPDH, GC, GCNT2, GIT2, GLCE, GLIPR2, GLUL, GMFG, GNG12, GRB2, GSE1, GUCA2A, H1-6, HAMP, HCK, HCLS1, HDAC9, HEBP1, HLA-A, HLA-G, HMOX1, HNRNPA2B1, HOTAIR, HOXA4, HOXA7, HSBP1, HSP90AA1, HSP90AB1, HSP90B1, HSPA1A/HSPA1B, HSPA5, HSPD1, HTATIP2, HVCN1, ICAM1, IER2, IFI16, IFNAR1, IFNGR1, IFT88, IGF1, IGF1R, IGF2BP3, IGF2R, IGFBP4, IL1B, IL1RN, ILF3, IP6K2, IRF8, ITGA4, ITGAX, ITGB8, JAK1, JAML, JPX, KCNE3, KDM5A, KIAA0319L, KIDINS220, KIF13A, KIF1C, KIF26B, KLF6, KLHL20, KMT5B, KRAS, LAMA5, LASP1, LCP1, LCP2, LEFTY1, let-7, LGALS8, LGMN, LILRB3, LIMK2, LIMS1, LINC00887, LITAF, LRP2, LRP8, LRPAP1, LSP1, LTBR, LUCAT1, LYN, LYVE1, LYZ, MACIR, MAK, MAP3K1, MAP3K2, MAP4, MAP4K4, MAPKAP1, MAPRE1, MAPRE3, MAX, MBP, MCL1, MCM3, MCM7, MDM2, MEF2C, MERTK, MGAT5, mir-122, mir-133, mir-137, mir-138, mir-154, mir-24, mir-26, mir-28, mir-299, mir-515, MMP14, MPP1, MS4A4A, MSN, MT1F, MTCH2, MTDH, MTOR, MUC1, MUC13, MXD1, MYH14, MYLK, MYO1F, MYO5A, MYO5B, MYOF, NAMPT, NARS1, NCF2, NCKAP1L, NCL, NDE1, NDEL1, NEDD9, NFATC4, NFE2L2, NFKBIZ, NINJ1, NKD2, NOTCH2, NR3C1, NRDC, NREP, NUAK2, NUMB, OPA1, P2RX1, PACSIN2, PAK2, PAQR3, PARK7, PDCD4, PDCL, PDE4B, PDGFRA, PDIA3, PEAK1, PECAM1, PF4, PHACTR1, PHLPP1, PILRA, PIP5K1A, PITX2, PLCB3, PLCG2, PLCL1, PLP1, PPIF, PPM1D, PRKAA1, PRKAR1A, PRKCD, PRKCG, PRKG1, PRL, PROK2, PRSS55, PSG1, PSMB8, PSMD10, PTEN, PTGS2, PTMA, PTPN6, RAB21, RAB27A, RAB5A, RABEP1, RAC2, RAF1, RALB, RALBP1, RAMP2, RAP1A, RAPGEF2, RCC2, RFFL, RGCC, RHOA, RHOB, RICTOR, RIN2, RIOK3, RIPK2, RIPK3, RNF11, RNF20, ROPN1L, RPL13A, RTN4, RUFY3, S100A14, S100A9, SCN9A, SCRIB, SDCBP, SEMA4A, SERPINB3, SFRP4, SGK1, SH2B3, SH3RF1, SHC3, SIGLEC9, SIRPA, SKP2, SLC11A1, SLC4A2, SLC7A7, SLC8A1, SMAD1, SNAP23, SNX27, SOCS4, SOD2, SOS2, SP1, SP100, SPAG9, SPATA13, SPHK2, SPOCK1, SRGN, SRSF1, SSH1, ST3GAL6, ST6GALNAC2, ST8SIA4, STAT3, STK24, STK35, STK4, STX3, SWAP70, SYK, TAFA4, TAGLN2, TAZ, TBX5, TBXAS1, TCAF1, TCF4, TDGF1, TDP2, TEKT4, TET2, THBS2, TJP1, TLR2, TLR4, TLR5, TLR7, TMOD3, TNFRSF10D, TNFRSF1A, TNFSF10, TNFSF14, TNFSF4, TNIP1, TPD52L1, TPM3, TREM1, TREML2, TRIM46, TRIM55, TRIO, TRIP10, TSPAN3, TUBA1A, TUBA1C, TXNRD1, TYROBP, UNC5C, USP17L2 (includes others), USP4, VCAN, VDAC1, VDR, VIM, VNN2, VTCN1, WARS1, WASF2, WASF3, WIPF1, WNK1, WWTR1, YBX1, YWHAE, YWHAZ, ZEB2, ZFYVE21, ZNF217, ZNF24
Cellular Function and Maintenance, Inflammatory Response	Phagocytosis	1.28E-08	Decreased	-4.656	102	ACTR2, AMPH, ANXA3, ANXA5, APOA1, APOA2, APP, APPL2, ARPC2, ATG2B, ATG3, ATG7, BECN1, BTK, CAMK1D, CD14, CD93, CEACAM3, CLCN3, CLEC4M, CLEC6A, CLEC7A, CLIC4, CLIP1, CORO1C, CSF1R, CSF3R, CYBB, DDX3X, DEF6, DET1, DNTTIP1, DOCK2, F10, FCGR2A, FPR1, FPR2, GAB2, GRB2, HCK, HMOX1, ICAM1, IFNAR1, IL1B, IRF8, JAK1, KAT6A, KCTD5, let-7, LYN, MAP1LC3A, MERTK, MEX3B, mir-24, MSN, MYO5A, NCKAP1L, NR3C1, PECAM1, PF4, PIP4P2, PIP5K1A, PLEK, PRKCD, PRKCG, PRLH, PSMD4, PTEN, PTPN6, RAB11A, RAB27A, RAB31, RAB5A, RAC2, RALB, RGCC, RHOA, RIT1, S100A9, SCARB2, SH3BP2, SIRPA, SIRPB1, SLAMF7, SLC11A1, SNAP23, SWAP70, SYK, TAFA4, TAZ, TBK1, TLR2, TLR4, TM2D2, TM9SF4, TREM1, TREML2, TYROBP, UBE2L3, VIM, WASF2, ZNF217
Cellular Function and Maintenance	Engulfment of cells	1.39E-08	Decreased	-5.778	129	ACTR2, AMPH, ANKFY1, ANXA3, ANXA5, APC, APLP2, APOA1, APOA2, APP, APPL2, ARF1, ARPC2, ATG3, ATG7, ATP6V1A, ATP6V1B2, BECN1, BTK, CAMK1D, CCL5, CD14, CD93, CDC5L, CEACAM3, CLCN3, CLEC4M, CLEC6A, CLEC7A, CLIC4, CLIP1, CORO1C, CSF1R, CSF3R, CTNND1, DAB2, DDX3X, DEF6, DET1, DNTTIP1, DOCK2, DPYSL2, EEF2K, EZR, F10, FCGR2A, FGD4, FPR1, FPR2, FRS2, GAB2, GRB2, HCK, HMOX1, HSP90AA1, HSPA5, ICAM1, IFNAR1, IL1B, IRF8, JAK1, KAT6A, KCTD5, KRAS, let-7, LRP2, LRPAP1, LYN, M6PR, MERTK, MEX3B, mir-24, MS4A4A, NCKAP1L, NCL, NR3C1, NTRK1, PARK7, PDZD8, PECAM1, PF4, PIP4P2, PIP5K1A, PLEK, PRKCD, PRKCG, PRLH, PSMD4, PTEN, PTPN6, RAB11A, RAB27A, RAB31, RAB4A, RAC2, RALB, RGCC, RHOA, RHOB, RIT1, RUFY1, S100A9, SCARB2, SH3BP2, SIRPA, SIRPB1, SLAMF7, SLC11A1, SNAP23, SNAP91, SRSF3, STK4, SWAP70, SYK, TAFA4, TLR2, TLR4, TM2D2, TM9SF4, TNFSF10, TREM1, TREML2, TYROBP, UBE2L3, VIM, WASF2, WIPF1, WNK1, ZNF217
Cellular Function and Maintenance	Internalization of cells	4.86E-08	Decreased	-4.781	79	ACTR2, APC, APOA1, APOA2, APP, APPL2, ARPC2, ATG7, BECN1, BTK, CD14, CD93, CEACAM3, CLCN3, CLEC4M, CLEC6A, CLEC7A, CLIC4, CLIP1, CSF1R, CSF3R, DDX3X, DEF6, DET1, DOCK2, EZR, FCGR2A, FGD4, FPR1, GAB2, GRB2, HCK, HMOX1, ICAM1, IFNAR1, IL1B, IRF8, JAK1, KAT6A, KCTD5, KRAS, let-7, LYN, MERTK, MEX3B, mir-24, NCKAP1L, NCL, PF4, PIP5K1A, PLEK, PRKCD, PSMD4, PTEN, PTPN6, RAB11A, RAB31, RAC2, RALB, RGCC, RHOA, RIT1, S100A9, SH3BP2, SIRPA, SIRPB1, SLAMF7, SNAP23, SNAP29, SYK, TLR2, TLR4, TM2D2, TREM1, TYROBP, UBE2L3, VIM, WASF2, ZNF217
Cellular Function and Maintenance	Endocytosis by eukaryotic cells	5.90E-08	Decreased	-5.392	98	ACTR2, ANXA5, APLP2, APOA1, APOA2, APP, APPL2, ARPC2, ATG7, ATP6V1A, ATP6V1B2, BECN1, BTK, CCL5, CD14, CD93, CDC5L, CEACAM3, CLCN3, CLEC4M, CLEC6A, CLIC4, CLIP1, CSF1R, CTNND1, DAB2, DDX3X, DEF6, DET1, DOCK2, DPYSL2, FCGR2A, FPR1, FRS2, GAB2, GRB2, HCK, HMOX1, HSP90AA1, HSPA5, ICAM1, IFNAR1, IL1B, IRF8, JAK1, KAT6A, KCTD5, let-7, LRPAP1, M6PR, MERTK, MEX3B, mir-24, MS4A4A, NCKAP1L, NR3C1, NTRK1, PARK7, PDZD8, PF4, PIP5K1A, PLEK, PRKCD, PSMD4, PTEN, PTPN6, RAB11A, RAB31, RALB, RGCC, RHOA, RHOB, RIT1, RUFY1, S100A9, SCARB2, SH3BP2, SIRPA, SIRPB1, SLAMF7, SNAP23, SNAP91, SRSF3, STK4, SWAP70, SYK, TLR2, TLR4, TM2D2, TNFSF10, TREM1, TREML2, TYROBP, UBE2L3, VIM, WASF2, WNK1, ZNF217
Cell-To-Cell Signaling and Interaction, Cellular Function and	Phagocytosis of cells	9.66E-08	Decreased	-4.496	91	ACTR2, ANXA3, ANXA5, APOA1, APOA2, APP, APPL2, ARPC2, ATG3, ATG7, BECN1, BTK, CAMK1D, CD14, CD93, CEACAM3, CLCN3, CLEC4M, CLEC6A, CLEC7A, CLIC4, CLIP1, CORO1C, CSF1R, CSF3R, DDX3X, DEF6, DET1, DNTTIP1, DOCK2, F10, FCGR2A, FPR1, FPR2, GAB2, GRB2, HCK, HMOX1, ICAM1, IFNAR1, IL1B, IRF8, JAK1, KAT6A, KCTD5, let-7, LYN, MERTK, MEX3B, mir-24, NCKAP1L, NR3C1, PECAM1, PF4, PIP4P2, PIP5K1A, PLEK, PRKCD, PRKCG, PRLH, PSMD4, PTEN, PTPN6, RAB11A, RAB27A, RAB31, RAC2, RALB, RGCC,

Maintenance, Inflammatory Response						RHOA, RIT1, S100A9, SH3BP2, SIRPA, SIRPB1, SLAMF7, SLC11A1, SNAP23, SWAP70, SYK, TAFA4, TLR2, TLR4, TM2D2, TM9SF4, TREML2, TYROBP, UBE2L3, VIM, WASF2, ZNF217
Infectious Diseases	Viral Infection	1.27E-07	Decreased	-7.999	338	ABCC2, ACP3, ACSL1, ACTN1, ACTR2, ADAM10, AGO2, ALG14, ALKBH3, ALKBH8, AMPH, ANXA2, ANXA5, APBB1IP, APC2, APOA1, APOB, APOBEC3B, APOL1, APP, ARF1, ARHGDIB, ARNTL, ARPC5, ARRDC3, ASMTL, ATF5, ATG7, ATOX1, ATP6AP2, ATP6V1A, ATP6V1B2, ATP6V1G1, B2M, BCL2L11, BECN1, BGN, BMP2K, BNIP2, BRCA1, BRINP2, BTG2, CALCOCO1, CAMK1D, CARD16, CCL5, CCNK, CCR1, CCT2, CD14, CD86, CD93, CFLAR, CHMP2A, CHMP3, CHMP4B, CHMP6, CHORDC1, CHST1, CHST6, CLEC4M, CLIC4, CLIP1, CNP, COG2, COG5, CPSF4, CREB1, CSF3R, CTLA4, CTSB, CTSZ, CXCL1, CXCL9, CXCR2, CXCR3, CYB5B, CYBB, CYP51A1, CYSTM1, DAZAP2, DCP1A, DDIT3, DDX17, DDX23, DDX3X, DDX5, DEFB103A/DEFB103B, DLGAP4, DNAJA2, DTX2, DUSP3, DYRK1A, EDEM3, EIF3A, EIF3G, EIF3I, ELOA, EPO, ERCC5, ETV3, F10, F11R, F13A1, F2R, F8, FAM228B, FAS-AS1, FCGR2A, FCGR2C, FGD6, FOXO3, FOXP3, FPR1, FRS2, FTL, G3BP2, GAB1, GAB2, GALC, GAPDH, GATAD2A, GCLC, GLUL, GLYR1, GRB2, GYG1, H2AC18/H2AC19, H2BC12, H2BC21, H3-3A/H3-3B, HBA1/HBA2, HCK, HERPUD1, HLA-A, HLA-C, HLA-E, HMCN2, HMOX1, HNRNPA1, HNRNPH1, HSP90AA1, HSP90AB1, HSP90B1, HSPA5, HSPD1, ICAM1, IFNAR1, IFNGR1, IGF1, IGF1R, IGF2R, IL1B, IL1RN, ILF3, IMPA2, INTS6, IRF8, ITGA4, JAK1, KAT6A, KDM7A, KMT5B, KRAS, LCP2, LEFTY1, LILRA2, LIMK2, LRPAP1, LSM3, LSP1, MAGT1, MAP1LC3A, MAP3K7CL, MAP4, MAP4K4, MAPKAPK3, MAPRE1, MAPRE3, MDM2, MED30, MED31, MERTK, MGAT5, MICB, mir-103, mir-122, mir-24, mir-515, MKNK1, MS4A1, MS4A4A, MT1X, MTOR, MVP, MX2, MXD1, MYO1F, MYO5B, MYOF, NACA2, NCL, NDE1, NFKBIZ, NLGN3, NMT1, NPC1L1, NPSR1-AS1, NR3C1, NUP160, NUP50, NUP58, NUP62, NUP93, OR5M1, OTUD3, P2RX1, PACSIN2, PAK2, PCK1, PDE4B, PDE8A, PDGFRA, PDIA3, PDZD8, PF4, PHF12, PIP5K1A, PLCG2, PPM1D, PRKAA1, PRKCD, PRL, PRPF38A, PRPF6, PSMC2, PSMD12, PSMD2, PSMD4, PTGS2, PTPN6, PURA, RAB11A, RAB31, RAB33B, RAB5A, RAB8A, RAB9A, RABEP1, RAF1, RALB, RBM25, RBM5, RBPJ, RFFL, RHOA, RHOB, RIPK2, RPL13A, RPL18, RPL38, RPL5, RTN3, S100A9, SART3, SBF2, SCARB2, SDCBP, SEC13, SEC14L1, SENP5, SERPINB3, SESTD1, SF3B1, SF3B6, SGCA, SGK1, SH2B3, SLC31A1, SLU7, SMARCA2, SNAP23, SNAPIN, SNRPD3, SNRPF, SP100, SP110, SPAST, SRPK1, SRPK2, SRSF1, SSR1, STAT3, STAU1, STIP1, TAGLN2, TALDO1, TBK1, TCF4, TFRC, TKFC, TLR2, TLR4, TLR7, TNFRSF1A, TNFSF10, TNFSF14, TNPO3, TRAF3, TRAF3IP1, TREM1, TRIM5, TRIM55, TRMT5, TRPT1, TSG101, TUBA1A, TUBA1C, TUBB2A, TYROBP, UBE2B, UBE2E2, UBE2L3, USP15, UTP11, VDR, VNN2, VPS4B, WASF2, WIPF1, WNK1, YBX1, ZEB2, ZMPSTE24, ZNF148, ZNF175, ZNF417/ZNF587, ZNF720
Cellular Function and Maintenance	Engulfment of blood cells	2.16E-07	Decreased	-3.931	67	ACTR2, ANXA5, APOA1, APOA2, APP, ARPC2, ATG7, BECN1, BTK, CCL5, CD14, CD93, CEACAM3, CLCN3, CLEC4M, CLEC6A, CLIC4, CSF1R, CTNND1, DEF6, DOCK2, FCGR2A, FPR1, GAB2, HCK, HMOX1, ICAM1, IFNAR1, IL1B, IRF8, JAK1, KAT6A, KCTD5, let-7, LYN, M6PR, MERTK, MEX3B, mir-24, NCKAP1L, PF4, PLEK, PRKCD, PTEN, PTPN6, RAB11A, RAC2, RGCC, RHOA, RIT1, S100A9, SH3BP2, SIRPA, SIRPB1, SLAMF7, STK4, SWAP70, SYK, TLR2, TLR4, TREM1, TREML2, TYROBP, UBE2L3, WASF2, WNK1, ZNF217
Cell-To-Cell Signaling and Interaction	Response of antigen presenting cells	2.73E-07	Decreased	-4.049	58	APOA1, APOA2, APP, ATG7, BECN1, BTK, CCL5, CD14, CD86, CD93, CLEC4M, CLEC6A, CLEC7A, CLEC9A, CLIC4, CSF1R, DEF6, DOCK2, F2R, FCAMR, FCGR2A, GAB2, HCK, HMOX1, HSP90AA1, IFNAR1, IL1B, IRF8, let-7, LYN, MERTK, MEX3B, mir-24, MUC1, NCKAP1L, NR3C1, PARK7, PLCB3, PRKAA1, PSMB8, PTEN, RAB11A, RGCC, S100A9, SEMA4A, SH3BP2, SIRPA, SIRPB1, SLAMF7, STAT3, SWAP70, SYK, TLR2, TLR4, TLR7, TNFSF4, TREM1, TYROBP
Cellular Movement	Migration of cells	2.92E-07	Decreased	-9.058	437	ACTG1, ACTN1, ADAM10, ADAM15, ADAM17, ADGRE2, ADGRG3, ADIPOR1, ADM, AGO2, AIF1, AKAP12, ALKBH1, ALOX5AP, ANGPTL4, ANXA2, ANXA3, APBA1, APBB1IP, APC, APLP2, APOA1, APOB, APP, APPL2, AQP9, ARF1, ARF4, ARHGAP25, ARHGDIB, ARNT, ARPC2, ARRDC3, ATAT1, ATG3, ATG7, ATOX1, ATRN, B4GALT1, BARHL1, BARX2, BCAS3, BECN1, BGN, BID, BRCA1, BTG2, BTK, CALML3, CALU, CAMK1D, CAP1, CARMIL1, CASP8, CAVIN2, CCDC88A, CCL23, CCL5, CCR1, CCR10, CD14, CD84, CD86, CD93, CDKL5, CEACAM3, CELSR3, CGA, CGB3 (includes others), CHST1, CLASP1, CLCA2, CLCN3, CLEC1B, CLEC4M, CLEC7A, CLIC4, CNP, CNR1, COL1A2, COL7A1, CORO1C, CPEB1, CRKL, CSF1R, CSF3R, CTLA4, CTNNA1, CTNND1, CTSB, CTSC, CTSZ, CUX1, CX3CR1, CXCL1, CXCL16, CXCL6, CXCL9, CXCR2, CXCR3, CYBB, CYP26B1, CYP2J2, DAB2, DDX3X, DEF6, DEFB103A/DEFB103B, DNAJB6, DOCK2, DOCK8, DPP10-AS1, DPYSL2, DSE, DUSP3, DUSP5, EFS, ELK3, ELN, EMC10, EPB41L5, EPHA8, EPHB1, EPO, EYA3, EZR, F10, F11R, F13A1, F2R, FAIM2, FBLN2, FCAMR, FCGR2A, FGD4, FGL2, FNBP1L, FOXO3, FOXP3, FPR1, FPR2, FRS2, FTH1, FTX, FUT7, FYB1, FZD3, GAB1, GAB2, GAL3ST1, GALNT1, GC, GCNT2, GIT2, GLIPR2, GLUL, GMFG, GNG12, GRB2, GSE1, GUCA2A, HAMP, HCK, HCLS1, HEBP1, HLA-A, HLA-G, HMOX1, HNRNPA2B1, HOTAIR, HOXA4, HOXA7, HSBP1, HSP90AA1, HSP90AB1, HSP90B1, HSPA1A/HSPA1B, HSPA5, HSPD1, HTATIP2, HVCN1, ICAM1, IFI16, IFNAR1, IFNGR1, IGF1, IGF1R, IGF2BP3, IGF2R, IGFBP4, IL1B, IL1RN, ILF3, IP6K2, ITGA4, ITGAX, ITGB8, JAK1, JAML, JPX, KCNE3, KDM5A, KIAA0319L, KIDINS220, KIF13A, KIF26B, KLF6, KLHL20, KMT5B, KRAS, LAMA5, LASP1, LCP1, LCP2, LEFTY1, let-7, LGMN, LILRB3, LIMK2, LINC00887, LITAF, LRP8, LRPAP1, LSP1, LTBR, LUCAT1, LYN, LYVE1, LYZ, MACIR, MAP3K1, MAP3K2, MAP4, MAP4K4, MAPKAP1, MAPRE1, MAX, MCL1, MCM3, MCM7, MDM2, MEF2C, MERTK, MGAT5, mir-122, mir-133, mir-137, mir-138, mir-154, mir-24, mir-26, mir-28, mir-299, mir-515, MMP14, MPP1, MS4A4A, MSN, MT1F, MTDH, MTOR, MUC1, MUC13, MXD1, MYLK, MYO1F, MYOF, NAMPT, NARS1, NCF2, NCKAP1L, NCL, NDE1, NDEL1, NEDD9, NFATC4, NFE2L2, NFKBIZ, NINJ1, NKD2, NOTCH2, NR3C1, NRDC, NREP, NUMB, OPA1, P2RX1, PACSIN2, PAK2, PAQR3, PARK7, PDCD4, PDCL, PDE4B, PDGFRA, PEAK1, PECAM1, PF4, PHACTR1, PILRA, PIP5K1A, PITX2, PLCB3, PLCG2, PLCL1, PLP1, PPIF, PPM1D, PRKAA1, PRKAR1A, PRKCD, PRKCG, PRKG1, PRL, PROK2, PRSS55, PSG1, PSMB8, PSMD10, PTEN, PTGS2, PTMA, PTPN6, RAB21,

						RAB27A, RAB5A, RABEP1, RAC2, RAF1, RALB, RALBP1, RAMP2, RAP1A, RAPGEF2, RCC2, RFFL, RGCC, RHOA, RHOB, RICTOR, RIN2, RIOK3, RIPK2, RNF11, RNF20, RPL13A, RTN4, RUFY3, S100A14, S100A9, SCN9A, SCRIB, SDCBP, SEMA4A, SERPINB3, SFRP4, SGK1, SH2B3, SH3RF1, SIGLEC9, SIRPA, SKP2, SLC4A2, SLC7A7, SLC8A1, SMAD1, SNAP23, SOCS4, SOD2, SOS2, SP1, SP100, SPAG9, SPATA13, SPHK2, SPOCK1, SRGN, SSH1, ST3GAL6, ST8SIA4, STAT3, STK24, STK35, STK4, SWAP70, SYK, TAFA4, TAZ, TBX5, TCAF1, TCF4, TDGF1, TDP2, THBS2, TJP1, TLR2, TLR4, TLR5, TLR7, TMOD3, TNFRSF10D, TNFRSF1A, TNFSF10, TNFSF14, TNFSF4, TNIP1, TPD52L1, TPM3, TREM1, TREML2, TRIM46, TRIM55, TRIO, TRIP10, TSPAN3, TUBA1A, TXNRD1, TYROBP, UNC5C, USP17L2 (includes others), USP4, VCAN, VDAC1, VDR, VIM, VNN2, VTCN1, WARS1, WASF2, WASF3, WIPF1, WWTR1, YBX1, YWHAE, YWHAZ, ZEB2, ZFYVE21, ZNF24
Cellular Movement, Hematological System Development and Function, Immune Cell Trafficking	Cell movement of leukocytes	3.00E-07	Decreased	-6.103	187	ADAM10, ADAM15, ADAM17, ADGRE2, ADM, AIF1, ALOX5AP, ANXA2, APBB1IP, APC, APOA1, APP, AQP9, ARHGAP25, ATG7, ATRN, B4GALT1, BECN1, BGN, BID, BTK, CAMK1D, CASP8, CCDC88A, CCL23, CCL5, CCR1, CCR10, CD14, CD86, CD93, CHST1, CLEC1B, CLEC4M, CNP, CNR1, CRKL, CSF1R, CSF3R, CTLA4, CTSB, CTSC, CTSZ, CUX1, CX3CR1, CXCL1, CXCL16, CXCL6, CXCL9, CXCR2, CXCR3, CYBB, CYP26B1, CYP2J2, DEF6, DEFB103A/DEFB103B, DOCK2, DOCK8, DPYSL2, EFS, ELN, EPO, EZR, F10, F11R, F2R, FCGR2A, FGL2, FOXP3, FPR1, FPR2, FUT7, FYB1, GAL3ST1, GALNT1, GIT2, HAMP, HCK, HCLS1, HEBP1, HLA-A, HLA-G, HMOX1, HSPA5, HSPD1, ICAM1, IFNAR1, IFNGR1, IL1B, IL1RN, ITGA4, ITGAX, JAK1, JAML, KCNE3, KLF6, KRAS, LAMA5, LCP1, LCP2, LGMN, LILRB3, LITAF, LSP1, LTBR, LYN, MAP3K2, MAPKAP1, MGAT5, mir-133, mir-154, MMP14, MPP1, MS4A4A, MSN, MTOR, MYLK, MYO1F, NARS1, NCKAP1L, NEDD9, NFE2L2, NFKBIZ, NINJ1, NR3C1, OPA1, PDE4B, PECAM1, PF4, PILRA, PLCB3, PLCG2, PLP1, PPM1D, PRKAA1, PRKCD, PRKG1, PROK2, PSMB8, PTEN, PTGS2, PTMA, PTPN6, RAB27A, RAC2, RAP1A, RGCC, RHOA, RHOB, RICTOR, RIPK2, RPL13A, RTN4, S100A14, S100A9, SCN9A, SCRIB, SEMA4A, SERPINB3, SGK1, SH2B3, SIRPA, SOS2, SPHK2, STAT3, STK4, SWAP70, SYK, TAFA4, THBS2, TLR2, TLR4, TLR7, TNFRSF1A, TNFSF10, TNFSF14, TNFSF4, TNIP1, TREM1, TREML2, TRIO, TYROBP, VCAN, VDR, VTCN1, WIPF1, YBX1
Inflammatory Response	Immune response of cells	3.17E-07	Decreased	-5.184	145	ACTR2, ALS2, ANXA3, ANXA5, APOA1, APOA2, APP, APPL2, ARPC2, ATG3, ATG7, ATXN3, BCL2L11, BECN1, BTK, CAMK1D, CASP8, CD14, CD86, CD93, CEACAM3, CLCN3, CLEC4M, CLEC6A, CLEC7A, CLEC9A, CLIC4, CLIP1, CORO1C, CSF1R, CSF3R, CTLA4, CTSB, CXCL1, DDX3X, DEF6, DET1, DNTTIP1, DOCK2, DOCK8, ELF3, F10, F2R, F8, FCAMR, FCGR2A, FFAR4, FPR1, FPR2, GAB2, GAPDH, GRB2, HCK, HMOX1, HSDL1, HSP90AA1, HSP90B1, ICAM1, IFNAR1, IFNGR1, IL1B, IRF8, ITGA4, ITGAX, JAK1, KAT6A, KCTD5, LCP2, let-7, LILRA2, LILRB3, LITAF, LYN, MCL1, MERTK, MEX3B, mir-24, mir-515, MS4A1, MTOR, MUC1, NAMPT, NCKAP1L, NR3C1, PECAM1, PF4, PIP4P2, PIP5K1A, PLEK, PLP1, PRKAA1, PRKCD, PRKCG, PRLH, PSMB8, PSMD4, PTEN, PTPN6, RAB11A, RAB27A, RAB31, RAC2, RALB, RALBP1, RGCC, RHOA, RICTOR, RIT1, S100A9, SEMA4A, SGK1, SH3BP2, SIAH1, SIRPA, SIRPB1, SLAMF7, SLC11A1, SNAP23, STAT3, STK4, SWAP70, SYK, TAFA4, TLR2, TLR4, TLR5, TLR7, TM2D2, TM9SF4, TNFRSF1A, TNFSF10, TNFSF4, TRAF3, TREM1, TREML2, TRIM23, TRIM5, TRIM55, TRIM65, TYROBP, UBE2L3, VIM, VTCN1, WASF2, ZNF217
Cell-To-Cell Signaling and Interaction, Inflammatory Response	Response of phagocytes	4.76E-07	Decreased	-3.823	62	ADGRE2, ANXA5, APOA1, APOA2, APP, ATG7, BECN1, BTK, CCL5, CCR1, CD14, CD93, CEACAM3, CLCN3, CLEC4M, CLEC6A, CLEC7A, CLIC4, CSF1R, CXCL1, DEF6, DOCK2, F2R, FCGR2A, FPR1, GAB2, HCK, HMOX1, ICAM1, IFNAR1, IL1B, IRF8, ITGA4, ITGAX, let-7, LILRB3, LYN, MERTK, MEX3B, mir-24, MUC1, NCKAP1L, NR3C1, PF4, PLCB3, PRKAA1, PTEN, RAB11A, RGCC, S100A9, SH3BP2, SIRPA, SIRPB1, SLAMF7, STAT3, SYK, TLR2, TLR4, TLR7, TREM1, TYROBP, ZEB2
Cellular Function and Maintenance, Hematological System Development and Function	Engulfment of myeloid cells	5.73E-07	Decreased	-4.124	49	APOA1, APOA2, APP, ATG7, BECN1, BTK, CD14, CD93, CEACAM3, CLCN3, CLEC4M, CLEC6A, CLIC4, CSF1R, CTNND1, DEF6, DOCK2, FCGR2A, FPR1, GAB2, HCK, HMOX1, ICAM1, IFNAR1, IL1B, IRF8, let-7, M6PR, MERTK, MEX3B, mir-24, NCKAP1L, PF4, PTEN, PTPN6, RAB11A, RGCC, RHOA, S100A9, SH3BP2, SIRPA, SIRPB1, SLAMF7, SYK, TLR2, TLR4, TREM1, TYROBP, WNK1
Cardiovascular Disease, Organismal Injury and Abnormalities	Peripheral arterial disease	8.22E-07			59	AMPH, APOA1, ARHGDIB, B2M, BTG2, CASP8, CCR1, CHORDC1, COL1A2, CTSB, CTSC, DAB2, DNAJB4, DNAJB5, EIF1B, F10, F2R, FCGR2A, FPR1, FTH1, FYB1, HBA1/HBA2, HBB, HCK, HCLS1, HSPA1A/HSPA1B, HTATIP2, IRF8, ITGA4, LCP1, LYN, MAP4K4, NEDD9, NGRN, NPC1L1, NR3C1, OTUD3, PACSIN2, PDLIM5, PLEKHO2, PPP1CB, PTGS2, RAB33B, RAB4A, RUFY1, RUSC1, SAT1, SGK1, SLC25A32, SSX2IP, STK24, SYK, TGOLN2, TLR7, TREM1, TUBA1A, TUBA1C, TUBB2A, USP15
Cellular Function and Maintenance	Engulfment of phagocytes	8.54E-07	Decreased	-3.613	50	ANXA5, APOA1, APOA2, APP, ATG7, BECN1, BTK, CD14, CD93, CEACAM3, CLCN3, CLEC4M, CLEC6A, CLIC4, CSF1R, CTNND1, DEF6, DOCK2, FCGR2A, GAB2, HCK, HMOX1, ICAM1, IFNAR1, IL1B, let-7, M6PR, MERTK, MEX3B, mir-24, NCKAP1L, PF4, PTEN, PTPN6, RAB11A, RGCC, RHOA, S100A9, SH3BP2, SIRPA, SIRPB1, SLAMF7, STK4, SWAP70, SYK, TLR2, TLR4, TREML2, TYROBP, WNK1
Cell-To-Cell Signaling and Interaction, Inflammatory Response	Immune response of antigen presenting cells	8.69E-07	Decreased	-3.666	53	APOA1, APOA2, APP, ATG7, BECN1, BTK, CD14, CD86, CD93, CLEC4M, CLEC6A, CLEC9A, CLIC4, CSF1R, DEF6, DOCK2, F2R, FCAMR, FCGR2A, GAB2, HCK, HMOX1, HSP90AA1, IFNAR1, IL1B, IRF8, let-7, LYN, MERTK, MEX3B, mir-24, MUC1, NCKAP1L, NR3C1, PRKAA1, PSMB8, PTEN, RAB11A, RGCC, S100A9, SEMA4A, SH3BP2, SIRPA, SIRPB1, SLAMF7, SWAP70, SYK, TLR2, TLR4, TLR7, TNFSF4, TREM1, TYROBP

Cellular Function and Maintenance	Engulfment of leukocytes	8.69E-07	Decreased	-4.064	53	ANXA5, APOA1, APOA2, APP, ATG7, BECN1, BTK, CCL5, CD14, CD93, CEACAM3, CLCN3, CLEC4M, CLEC6A, CLIC4, CSF1R, CTNND1, DEF6, DOCK2, FCGR2A, FPR1, GAB2, HCK, HMOX1, ICAM1, IFNAR1, IL1B, let-7, M6PR, MERTK, MEX3B, mir-24, NCKAP1L, PF4, PTEN, PTPN6, RAB11A, RGCC, RHOA, S100A9, SH3BP2, SIRPA, SIRPB1, SLAMF7, STK4, SWAP70, SYK, TLR2, TLR4, TREM1, TREML2, TYROBP, WNK1
Cellular Movement	Cell movement of blood cells	1.18E-06	Decreased	-6.463	216	ADAM10, ADAM15, ADAM17, ADGRE2, ADGRG3, ADM, AIF1, ALOX5AP, ANXA2, APBB1IP, APC, APOA1, APOB, APP, AQP9, ARHGAP25, ATG7, ATRN, B4GALT1, BECN1, BGN, BID, BTK, CAMK1D, CASP8, CCDC88A, CCL23, CCL5, CCR1, CCR10, CD14, CD84, CD86, CD93, CEACAM3, CHST1, CLEC1B, CLEC4M, CLEC7A, CNP, CNR1, COL1A2, CRKL, CSF1R, CSF3R, CTLA4, CTSB, CTSC, CTSZ, CUX1, CX3CR1, CXCL1, CXCL16, CXCL6, CXCL9, CXCR2, CXCR3, CYBB, CYP26B1, CYP2J2, DDX3X, DEF6, DEFB103A/DEFB103B, DOCK2, DOCK8, DPYSL2, EFS, ELN, EPO, EZR, F10, F11R, F13A1, F2R, FCAMR, FCGR2A, FGL2, FOXP3, FPR1, FPR2, FRS2, FUT7, FYB1, GAB2, GAL3ST1, GALNT1, GC, GIT2, GRB2, HAMP, HCK, HCLS1, HEBP1, HLA-A, HLA-G, HMOX1, HOXA7, HSPA1A/HSPA1B, HSPA5, HSPD1, ICAM1, IFNAR1, IFNGR1, IGF1, IL1B, IL1RN, ITGA4, ITGAX, JAK1, JAML, KCNE3, KLF6, KRAS, LAMA5, LCP1, LCP2, LGMN, LILRB3, LITAF, LSP1, LTBR, LYN, LYZ, MAP3K2, MAPKAP1, MERTK, MGAT5, mir-133, mir-154, MMP14, MPP1, MS4A4A, MSN, MTOR, MYLK, MYO1F, NARS1, NCKAP1L, NEDD9, NFE2L2, NFKBIZ, NINJ1, NR3C1, OPA1, P2RX1, PDE4B, PECAM1, PF4, PILRA, PLCB3, PLCG2, PLP1, PPM1D, PRKAA1, PRKCD, PRKG1, PROK2, PSG1, PSMB8, PTEN, PTGS2, PTMA, PTPN6, RAB27A, RAC2, RAP1A, RGCC, RHOA, RHOB, RICTOR, RIPK2, RIPK3, RPL13A, RTN4, S100A14, S100A9, SCN9A, SCRIB, SEMA4A, SERPINB3, SGK1, SH2B3, SIGLEC9, SIRPA, SLC7A7, SOD2, SOS2, SPHK2, ST3GAL6, STAT3, STK4, SWAP70, SYK, TAFA4, TET2, THBS2, TLR2, TLR4, TLR5, TLR7, TNFRSF10D, TNFRSF1A, TNFSF10, TNFSF14, TNFSF4, TNIP1, TREM1, TREML2, TRIO, TYROBP, VCAN, VDR, VNN2, VTCN1, WIPF1, YBX1
Cellular Movement, Immune Cell Trafficking	Leukocyte migration	1.22E-06	Decreased	-6.465	214	ADAM10, ADAM15, ADAM17, ADGRE2, ADGRG3, ADM, AIF1, ALOX5AP, ANXA2, APBB1IP, APC, APOA1, APOB, APP, AQP9, ARHGAP25, ATG7, ATRN, B4GALT1, BECN1, BGN, BID, BTK, CAMK1D, CASP8, CCDC88A, CCL23, CCL5, CCR1, CCR10, CD14, CD84, CD86, CD93, CEACAM3, CHST1, CLEC1B, CLEC4M, CLEC7A, CNP, CNR1, COL1A2, CRKL, CSF1R, CSF3R, CTLA4, CTSB, CTSC, CTSZ, CUX1, CX3CR1, CXCL1, CXCL16, CXCL6, CXCL9, CXCR2, CXCR3, CYBB, CYP26B1, CYP2J2, DDX3X, DEF6, DEFB103A/DEFB103B, DOCK2, DOCK8, DPYSL2, EFS, ELN, EPO, EZR, F10, F11R, F13A1, F2R, FCAMR, FCGR2A, FGL2, FOXP3, FPR1, FPR2, FRS2, FUT7, FYB1, GAB2, GAL3ST1, GALNT1, GC, GIT2, GRB2, HAMP, HCK, HCLS1, HEBP1, HLA-A, HLA-G, HMOX1, HOXA7, HSPA1A/HSPA1B, HSPA5, HSPD1, ICAM1, IFNAR1, IFNGR1, IGF1, IL1B, IL1RN, ITGA4, ITGAX, JAK1, JAML, KCNE3, KLF6, KRAS, LAMA5, LCP1, LCP2, LGMN, LILRB3, LITAF, LSP1, LTBR, LYN, LYZ, MAP3K2, MAPKAP1, MERTK, MGAT5, mir-133, mir-154, MMP14, MPP1, MS4A4A, MSN, MTOR, MYLK, MYO1F, NARS1, NCKAP1L, NEDD9, NFE2L2, NFKBIZ, NINJ1, NR3C1, OPA1, P2RX1, PDE4B, PECAM1, PF4, PILRA, PLCB3, PLCG2, PLP1, PPM1D, PRKAA1, PRKCD, PRKG1, PROK2, PSG1, PSMB8, PTEN, PTGS2, PTMA, PTPN6, RAB27A, RAC2, RAP1A, RGCC, RHOA, RHOB, RICTOR, RIPK2, RPL13A, RTN4, S100A14, S100A9, SCN9A, SCRIB, SEMA4A, SERPINB3, SGK1, SH2B3, SIGLEC9, SIRPA, SLC7A7, SOD2, SOS2, SPHK2, ST3GAL6, STAT3, STK4, SWAP70, SYK, TAFA4, THBS2, TLR2, TLR4, TLR5, TLR7, TNFRSF10D, TNFRSF1A, TNFSF10, TNFSF14, TNFSF4, TNIP1, TREM1, TREML2, TRIO, TYROBP, VCAN, VDR, VNN2, VTCN1, WIPF1, YBX1
Cellular Movement, Hematological System Development and Function, Immune Cell Trafficking, Inflammatory Response	Cell movement of phagocytes	1.32E-06	Decreased	-5.681	138	ADAM10, ADAM15, ADAM17, ADM, AIF1, ALOX5AP, ANXA2, APOA1, APP, AQP9, ARHGAP25, ATRN, B4GALT1, BECN1, BID, BTK, CAMK1D, CASP8, CCDC88A, CCL23, CCL5, CCR1, CD14, CD86, CLEC1B, CLEC4M, CNP, CNR1, CRKL, CSF1R, CSF3R, CTSB, CTSC, CTSZ, CX3CR1, CXCL1, CXCL6, CXCL9, CXCR2, CXCR3, CYBB, CYP2J2, DEFB103A/DEFB103B, DOCK2, DOCK8, ELN, EPO, F10, F11R, F2R, FCGR2A, FPR1, FPR2, FUT7, GAL3ST1, GIT2, HAMP, HCK, HCLS1, HEBP1, HMOX1, HSPA5, ICAM1, IFNGR1, IL1B, IL1RN, ITGA4, ITGAX, JAK1, JAML, KLF6, LGMN, LILRB3, LITAF, LSP1, LYN, MGAT5, mir-133, MMP14, MPP1, MS4A4A, MYLK, MYO1F, NARS1, NCKAP1L, NFE2L2, NFKBIZ, NINJ1, OPA1, PDE4B, PECAM1, PF4, PILRA, PLCB3, PLCG2, PLP1, PPM1D, PRKCD, PRKG1, PROK2, PSMB8, PTEN, PTGS2, PTMA, PTPN6, RAB27A, RAC2, RHOA, RHOB, RICTOR, RPL13A, RTN4, S100A14, S100A9, SCN9A, SEMA4A, SGK1, SH2B3, SIRPA, SPHK2, STAT3, STK4, SWAP70, SYK, TAFA4, THBS2, TLR2, TLR4, TLR7, TNFRSF1A, TNFSF4, TNIP1, TREM1, TREML2, TYROBP, VCAN, VTCN1, YBX1
Cell-To-Cell Signaling and Interaction	Response of myeloid cells	1.41E-06	Decreased	-3.969	60	ADGRE2, APOA1, APOA2, APP, ATG7, BECN1, BTK, CCR1, CD14, CD93, CEACAM3, CLCN3, CLEC4M, CLEC6A, CLEC7A, CLIC4, CSF1R, CXCL1, DEF6, DOCK2, F2R, FCGR2A, FPR1, GAB2, HCK, HMOX1, ICAM1, IFNAR1, IL1B, IRF8, ITGA4, ITGAX, let-7, LILRB3, LYN, MERTK, MEX3B, mir-24, NCKAP1L, NR3C1, PARK7, PF4, PLCB3, PRKAA1, PTEN, RAB11A, RGCC, S100A9, SH3BP2, SIRPA, SIRPB1, SLAMF7, STAT3, SYK, TLR2, TLR4, TLR7, TREM1, TYROBP, ZEB2
Cellular Movement	Cell movement of tumor cell lines	1.43E-06	Decreased	-7.086	233	ACTN1, ADAM10, ADAM15, ADAM17, AGO2, AIF1, AKAP12, ANGPTL4, ANXA2, APC, APP, ARF1, ARHGDIB, ARPC2, ARRDC3, ATOX1, BRCA1, CALML3, CALU, CAP1, CASP8, CCDC88A, CCL23, CCL5, CGB3 (includes others), CLCN3, CLEC1B, CLIC4, CLIP1, COL7A1, CRKL, CSF1R, CTNND1, CTSB, CTSZ, CXCL1, CXCL9, CXCR2, CXCR3, CYP2J2, DAB2, DEF6, DEFB103A/DEFB103B, DKK3, DNAJB4, DNAJB6, DOCK2, DOCK8, DPP10-AS1, DPYSL2, DSE, DUSP5, EIF3A, EPHB1, EPO, EYA3, EZR, F11R, F2R, FAIM2, FBLN2, FFAR4, FGD4, FNBP1L, FOXO3, FOXP3, FPR1, FPR2, FTX, FUT7, FYB1, GAB1, GAB2, GC, GIT2, GMFG, GRB2, GSE1, HCK, HDAC9, HMOX1, HNRNPA2B1, HOTAIR, HOXA4, HSBP1, HSP90AA1, HSP90B1, HSPA1A/HSPA1B, HTATIP2, HVCN1, IFNAR1, IGF1, IGF1R, IGF2BP3, IGFBP4, IL1B, ILF3, IP6K2, ITGA4, JAK1, JPX, KDM5A, KIDINS220, KLF6, KRAS, LAMA5, LASP1, LCP1, LCP2, let-7, LGALS8, LIMK2, LINC00887, LRPAP1, LUCAT1, LYN, LYVE1, MAP3K1, MAP4, MAP4K4, MAPRE3, MDM2, MERTK, MGAT5, mir-122, mir-154, mir-24, mir-26, mir-28, mir-299, mir-515, MMP14, MSN, MTCH2, MTDH, MTOR, MUC1, MUC13, MYOF, NAMPT, NCL, NEDD9, NFATC4, NFE2L2, NINJ1, NKD2, NOTCH2, NREP, NUMB, P2RX1, PACSIN2, PAK2, PDCD4, PDGFRA, PEAK1, PECAM1, PHACTR1, PHLPP1, PITX2, PLCL1, PPIF,

						PRKAA1, PRKCD, PRKCG, PRKG1, PRL, PSMD10, PTEN, PTGS2, PTPN6, RAB21, RAB27A, RAB5A, RAC2, RAF1, RALB, RALBP1, RAP1A, RFFL, RHOA, RHOB, RICTOR, RIOK3, RNF11, RNF20, S100A14, S100A9, SCRIB, SDCBP, SEMA4A, SERPINB3, SFRP4, SH2B3, SHC3, SIRPA, SKP2, SMAD1, SNX27, SOCS4, SOD2, SP1, SPHK2, SRGN, SSH1, ST6GALNAC2, STAT3, STK24, SYK, TAGLN2, TAZ, TBXAS1, TCAF1, TCF4, TDGF1, THBS2, TLR2, TLR4, TNFSF10, TPD52L1, TPM3, TRIO, TRIP10, TUBA1C, USP4, VCAN, VDAC1, VIM, WARS1, WASF2, WWTR1, YBX1, ZEB2, ZFYVE21
Cancer, Gastrointestinal Disease, Organismal Injury and Abnormalities	Duodenal cancer	1.51E-06			21	APC, B2M, BCL7A, FCRLA, GSE1, HSP90AA1, HSP90AB1, HSP90B1, HVCN1, IRF8, KBTBD12, KRAS, MCL1, MSH6, PRKCD, PRKCG, PTEN, RHOA, TET2, TRAF3, U2AF1/U2AF1L5
Cell-To-Cell Signaling and Interaction, Inflammatory Response	Immune response of phagocytes	1.61E-06	Decreased	-3.115	56	ANXA5, APOA1, APOA2, APP, ATG7, BECN1, BTK, CD14, CD93, CEACAM3, CLCN3, CLEC4M, CLEC6A, CLIC4, CSF1R, CXCL1, DEF6, DOCK2, F2R, FCGR2A, FPR1, GAB2, HCK, HMOX1, ICAM1, IFNAR1, IL1B, IRF8, ITGA4, ITGAX, let-7, LILRB3, LYN, MERTK, MEX3B, mir-24, MUC1, NCKAP1L, NR3C1, PF4, PRKAA1, PTEN, RAB11A, RGCC, S100A9, SH3BP2, SIRPA, SIRPB1, SLAMF7, STAT3, SYK, TLR2, TLR4, TLR7, TREM1, TYROBP
Hematological Disease, Immunological Disease	Abnormal function of neutrophils	1.89E-06			25	B4GALT1, CCR1, CD14, CLCN3, CLEC6A, CLEC7A, CXCL6, CXCR2, CYBB, ENTPD1, FCGR2A, FUT7, GIT2, HCK, LCP1, LYN, MPP1, NFE2L2, PILRA, PLCB3, PRKCD, RAC2, RAP1A, SIGLEC9, SYK
Hematological Disease	Hemorrhagic disease	2.19E-06	Increased	2.222	69	APC, APP, ARID4B, ARNT, ASXL1, ATG7, BCL2L11, C1GALT1C1, CCL5, CD93, CDA, CLEC1B, CLEC4M, CSF3R, CTSB, CXCL1, ENTPD1, ETV6, F10, F13A1, F2R, F8, FCGR2A, FCGR2C, FOXP3, FYB1, HSP90B1, HSPA5, IFNAR1, IFNGR1, IFNGR2, IL1RN, IREB2, let-7, LYN, MAPKAP1, MDM2, mir-154, MIR4270, MS4A1, MTOR, MX2, NFE2L2, NR3C1, P2RX1, PAK2, PLEK, PSMD1, PSMD2, PTEN, PTGS2, RAP1A, RPL18, RPL5, S100A9, SIRPA, SLAMF7, SLC11A1, SP1, SP3, SPHK2, ST3GAL6, SYK, THBS2, TLR7, TNFSF10, TNNC1, VDR, WIPF1
Cell-To-Cell Signaling and Interaction, Inflammatory Response	Immune response of tumor cell lines	2.31E-06	Decreased	-2.435	54	ACTR2, APPL2, ARPC2, ATG7, ATXN3, BECN1, BTK, CD93, CLIP1, CTSB, DEF6, DET1, DOCK2, FCGR2A, GAPDH, GRB2, HCK, HMOX1, HSP90B1, ICAM1, IL1B, JAK1, KAT6A, KCTD5, MCL1, MERTK, mir-515, MS4A1, MTOR, NCKAP1L, NR3C1, PIP5K1A, PLEK, PRKCD, PSMD4, PTEN, PTPN6, RAB11A, RAB31, RALB, RHOA, RIT1, SIAH1, SIRPA, SLAMF7, STAT3, TLR4, TM2D2, TRAF3, TYROBP, UBE2L3, VIM, WASF2, ZNF217
Cell-To-Cell Signaling and Interaction, Cellular Function and Maintenance, Inflammatory Response	Phagocytosis of blood cells	2.57E-06	Decreased	-3.481	58	ACTR2, ANXA5, APOA1, APOA2, APP, ARPC2, ATG7, BECN1, BTK, CD14, CD93, CEACAM3, CLCN3, CLEC4M, CLEC6A, CLIC4, CSF1R, DEF6, DOCK2, FCGR2A, FPR1, GAB2, HCK, HMOX1, ICAM1, IFNAR1, IL1B, IRF8, JAK1, KAT6A, KCTD5, let-7, LYN, MERTK, MEX3B, mir-24, NCKAP1L, PF4, PLEK, PRKCD, PTEN, RAB11A, RAC2, RGCC, RIT1, S100A9, SH3BP2, SIRPA, SIRPB1, SLAMF7, SYK, TLR2, TLR4, TREML2, TYROBP, UBE2L3, WASF2, ZNF217
Cell-To-Cell Signaling and Interaction, Cellular Function and Maintenance, Hematological System Development and Function	Phagocytosis of myeloid cells	2.81E-06	Decreased	-3.885	44	APOA1, APOA2, APP, ATG7, BECN1, BTK, CD14, CD93, CEACAM3, CLCN3, CLEC4M, CLEC6A, CLIC4, CSF1R, DEF6, DOCK2, FCGR2A, FPR1, GAB2, HCK, HMOX1, ICAM1, IFNAR1, IL1B, IRF8, let-7, MERTK, MEX3B, mir-24, NCKAP1L, PF4, PTEN, RAB11A, RGCC, S100A9, SH3BP2, SIRPA, SIRPB1, SLAMF7, SYK, TLR2, TLR4, TREM1, TYROBP
Cellular Movement	Diapedesis	2.87E-06	Decreased	-2.425	9	ADAM17, CXCL1, F11R, IL1B, ITGA4, LSP1, PECAM1, RHOA, TRIM55
Cardiovascular Disease, Organismal Injury and Abnormalities	Intermediate disease stage peripheral arterial disease	3.06E-06			40	AMPH, ARHGDIB, BTG2, CASP8, CCR1, CTSB, CTSC, DAB2, DNAJB4, DNAJB5, EIF1B, FCGR2A, FPR1, FYB1, HBA1/HBA2, HBB, HCK, HCLS1, IRF8, ITGA4, LCP1, LYN, MAP4K4, NGRN, PACSIN2, PDLIM5, PLEKHO2, PPP1CB, RAB33B, RAB4A, RUFY1, SAT1, SGK1, SSX2IP, STK24, SYK, TGOLN2, TLR7, TREM1, USP15
Cell-To-Cell Signaling and	Immune response of leukocytes	3.29E-06	Decreased	-3.946	79	ANXA5, APOA1, APOA2, APP, ATG7, BCL2L11, BECN1, BTK, CASP8, CD14, CD86, CD93, CEACAM3, CLCN3, CLEC4M, CLEC6A, CLEC9A, CLIC4, CSF1R, CTLA4, CXCL1, DEF6, DOCK2, DOCK8, F2R, F8, FCAMR, FCGR2A, FFAR4, FPR1, GAB2, HCK, HMOX1, HSDL1, HSP90AA1,

Interaction, Inflammatory Response						ICAM1, IFNAR1, IFNGR1, IL1B, IRF8, ITGA4, ITGAX, LCP2, let-7, LILRB3, LYN, MERTK, MEX3B, mir-24, MTOR, MUC1, NAMPT, NCKAP1L, NR3C1, PF4, PLP1, PRKAA1, PSMB8, PTEN, RAB11A, RGCC, S100A9, SEMA4A, SH3BP2, SIRPA, SIRPB1, SLAMF7, STAT3, SWAP70, SYK, TLR2, TLR4, TLR7, TNFRSF1A, TNFSF4, TREM1, TREML2, TYROBP, VTCN1
Cell-To-Cell Signaling and Interaction	Binding of blood cells	3.76E-06	Decreased	-4.749	100	ADAM10, ADAM17, ADGRE2, ANXA5, APBB1IP, APOA1, APP, ATRN, B4GALT1, BTK, C1GALT1C1, CCL5, CCR1, CD14, CD84, CD86, CLEC1B, CLEC4M, CNR1, CSF3R, CTLA4, CTSZ, CX3CR1, CXCL1, CXCL9, CXCR2, CXCR3, CYBB, DOCK2, DOCK8, ENTPD1, EZR, F10, F11R, F2R, F8, FCGR2A, FPR1, FPR2, FUT7, FYB1, GAB2, GALNT1, HCK, ICAM1, IFNGR1, IL1B, IRF8, ITGA4, ITGAX, JAK1, LAMA5, LCP1, LCP2, LGALS8, LILRB3, LRP8, LRPAP1, LSP1, LTBR, LYN, MAP3K2, MGAT5, MSN, NEDD9, NFE2L2, NINJ1, NOTCH2, NR3C1, PAK2, PECAM1, PF4, PILRA, PLCB3, PLCG2, PRL, PTGS2, PTPN6, RAC2, RAP1A, RHOA, RHOB, RICTOR, S100A9, SIRPA, ST6GALNAC2, STK4, SWAP70, SYK, TFRC, THBS2, TLR2, TLR4, TLR5, TLR7, TNFRSF1A, TNFSF14, TNIP1, TYROBP, WIPF1
Cell-To-Cell Signaling and Interaction	Response of tumor cell lines	3.90E-06	Decreased	-2.286	63	ACTR2, APP, APPL2, ARPC2, ATG7, ATXN3, BECN1, BTK, CD93, CLIP1, CTSB, DDIT3, DEF6, DET1, DOCK2, ERO1A, FCGR2A, GAPDH, GRB2, HCK, HFE, HMOX1, HNRNPA1, HSP90B1, HSPA5, ICAM1, IGF1, IL1B, JAK1, KAT6A, KCTD5, MCL1, MERTK, mir-515, MS4A1, MTOR, NCKAP1L, NR3C1, PIP5K1A, PLEK, PRKCD, PSMD4, PTEN, PTPN6, RAB11A, RAB31, RALB, RHOA, RIT1, RTN1, SIAH1, SIRPA, SLAMF7, STAT3, TLR4, TM2D2, TMBIM6, TRAF3, TYROBP, UBE2L3, VIM, WASF2, ZNF217
RNA Post-Transcriptional Modification	Processing of mRNA	4.55E-06		0.421	65	AGO2, AKAP8L, APP, BUD13, CASC3, CDC5L, CELF1, CPEB1, CPSF4, CPSF7, CSTF1, CWC15, DDX17, DDX23, DDX39A, DDX5, DHX38, DHX8, DYRK1A, FUS, GTF2H3, HBB, HNRNPA1, HNRNPA2B1, HNRNPH1, HNRNPH2, IK, LSM3, LSM6, MAGOHB, NCBP1, NONO, NSRP1, NUDT21, PABPC1, PNN, PQBP1, PRPF38A, PRPF4, PRPF6, RBM22, RBM25, RBM4, RBM5, SART3, SF3A3, SF3B1, SF3B6, SLU7, SNRPB, SNRPD3, SNRPF, SRPK1, SRPK2, SRSF1, SRSF3, SRSF4, SRSF5, THRAP3, U2AF1/U2AF1L5, WBP11, WDR33, YBX1, ZMAT2, ZRSR2
Cellular Movement, Hematological System Development and Function, Immune Cell Trafficking, Inflammatory Response	Cell movement of neutrophils	4.72E-06	Decreased	-3.831	79	ADAM10, ADAM15, ADAM17, ADM, ALOX5AP, ANXA2, APOA1, APP, AQP9, ARHGAP25, B4GALT1, BTK, CAMK1D, CCL23, CCL5, CCR1, CD14, CSF3R, CTSB, CTSC, CXCL1, CXCL6, CXCL9, CXCR2, CYBB, CYP2J2, DEFB103A/DEFB103B, DOCK2, F10, FCGR2A, FPR1, FPR2, FUT7, GIT2, HCK, HMOX1, ICAM1, IFNGR1, IL1B, IL1RN, ITGA4, JAML, LILRB3, LSP1, LYN, MGAT5, mir-133, MPP1, MYLK, MYO1F, NCKAP1L, NFKBIZ, PDE4B, PECAM1, PF4, PLCB3, PLCG2, PLP1, PPM1D, PRKG1, PTEN, PTGS2, PTPN6, RAB27A, RAC2, RTN4, S100A9, SGK1, STAT3, SYK, TLR2, TLR4, TLR7, TNFRSF1A, TNIP1, TREM1, TREML2, VTCN1, YBX1
Cardiovascular Disease, Organismal Injury and Abnormalities	Advanced stage peripheral arterial disease	4.99E-06			38	AMPH, ARHGDIB, CASP8, CCR1, CHORDC1, COL1A2, CTSB, DAB2, DNAJB4, DNAJB5, F10, FTH1, FYB1, HBA1/HBA2, HBB, HCLS1, HSPA1A/HSPA1B, HTATIP2, IRF8, ITGA4, LCP1, LYN, MAP4K4, NEDD9, NGRN, OTUD3, PDLIM5, PLEKHO2, PPP1CB, RAB33B, RUFY1, RUSC1, SLC25A32, SSX2IP, STK24, SYK, TLR7, USP15
RNA Post-Transcriptional Modification	Splicing of RNA	5.20E-06		0.806	62	ATXN3, BUD13, CASC3, CDC5L, CELF1, CLK2, CLK3, CPSF4, CPSF7, CSTF1, CWC15, DDX17, DDX23, DDX39A, DDX5, DHX38, DHX8, DYRK1A, FUS, HNRNPA1, HNRNPA2B1, HNRNPH1, HNRNPH2, IK, LSM3, LSM6, MAGOHB, NCBP1, NONO, NSRP1, NUDT21, PNN, PQBP1, PRPF38A, PRPF4, PRPF6, RBM22, RBM25, RBM4, RBM5, SART3, SF3A3, SF3B1, SF3B6, SLU7, SNRPB, SNRPD3, SNRPF, SRPK1, SRPK2, SRSF1, SRSF3, SRSF4, SRSF5, THRAP3, U2AF1/U2AF1L5, WBP11, WDR33, YBX1, ZMAT2, ZNF326, ZRSR2
Cellular Function and Maintenance	Engulfment of antigen presenting cells	6.75E-06	Decreased	-3.36	43	APOA1, APOA2, APP, ATG7, BECN1, BTK, CD14, CD93, CLEC4M, CLEC6A, CLIC4, CSF1R, CTNND1, DEF6, DOCK2, FCGR2A, GAB2, HCK, HMOX1, IL1B, let-7, M6PR, MERTK, MEX3B, mir-24, NCKAP1L, PTEN, PTPN6, RAB11A, RGCC, RHOA, S100A9, SH3BP2, SIRPA, SIRPB1, SLAMF7, STK4, SWAP70, TLR2, TLR4, TREML2, TYROBP, WNK1
RNA Post-Transcriptional Modification	Splicing of mRNA	7.06E-06		0.883	55	BUD13, CASC3, CDC5L, CPSF4, CPSF7, CSTF1, CWC15, DDX17, DDX23, DDX39A, DDX5, DHX38, DHX8, DYRK1A, FUS, HNRNPA1, HNRNPA2B1, HNRNPH1, HNRNPH2, IK, LSM3, LSM6, MAGOHB, NCBP1, NSRP1, NUDT21, PNN, PQBP1, PRPF38A, PRPF4, PRPF6, RBM22, RBM25, RBM4, RBM5, SART3, SF3A3, SF3B1, SF3B6, SLU7, SNRPB, SNRPD3, SNRPF, SRPK2, SRSF1, SRSF3, SRSF4, SRSF5, THRAP3, U2AF1/U2AF1L5, WBP11, WDR33, YBX1, ZMAT2, ZRSR2
Cellular Movement	Cell movement of myeloid cells	7.17E-06	Decreased	-5.317	133	ADAM10, ADAM15, ADAM17, ADM, AIF1, ALOX5AP, ANXA2, APOA1, APP, AQP9, ARHGAP25, ATRN, B4GALT1, BECN1, BID, BTK, CAMK1D, CASP8, CCDC88A, CCL23, CCL5, CCR1, CD14, CNP, CNR1, CRKL, CSF1R, CSF3R, CTSB, CTSC, CX3CR1, CXCL1, CXCL6, CXCL9, CXCR2, CXCR3, CYBB, CYP2J2, DEFB103A/DEFB103B, DOCK2, ELN, EPO, F10, F11R, F2R, FCGR2A, FPR1, FPR2, FUT7, FYB1, GAL3ST1, GIT2, HAMP, HCK, HEBP1, HMOX1, HSPA5, ICAM1, IFNGR1, IL1B, IL1RN, ITGA4, ITGAX, JAK1, JAML, KLF6, LAMA5, LGMN, LILRB3, LITAF, LSP1, LYN, MGAT5, mir-133, MMP14, MPP1, MS4A4A, MTOR, MYLK, MYO1F, NCKAP1L, NFE2L2, NFKBIZ, NINJ1, OPA1, PDE4B, PECAM1, PF4, PILRA, PLCB3, PLCG2, PLP1, PPM1D, PRKCD, PRKG1, PROK2, PSMB8, PTEN, PTGS2, PTMA, PTPN6, RAB27A, RAC2, RHOA, RHOB, RICTOR, RPL13A, RTN4, S100A14, S100A9, SEMA4A, SGK1, SH2B3, SIRPA, SPHK2, STAT3, SWAP70, SYK, TAFA4, TET2, THBS2, TLR2, TLR4, TLR7, TNFRSF1A, TNFSF4, TNIP1, TREM1, TREML2, TYROBP, VCAN, VTCN1, YBX1
Cellular Movement, Hematological System	Migration of phagocytes	7.31E-06	Decreased	-4.588	71	ADAM10, ADAM15, ADAM17, ANXA2, APOA1, APP, BTK, CCL5, CCR1, CD86, CLEC1B, CLEC4M, CNR1, CSF1R, CTSZ, CX3CR1, CXCL1, CXCL6, CXCL9, CXCR2, CXCR3, CYBB, DEFB103A/DEFB103B, DOCK8, F10, F11R, FPR1, FPR2, HCK, HCLS1, ICAM1, IL1B, IL1RN, ITGA4, ITGAX, JAK1, KLF6, LSP1, MGAT5, mir-133, MMP14, MS4A4A, MYLK, MYO1F, NARS1, NINJ1, PDE4B, PECAM1, PILRA, PPM1D,

Development and Function, Immune Cell Trafficking, Inflammatory Response						PROK2, PTEN, PTGS2, PTPN6, RAC2, RHOA, RHOB, RTN4, S100A9, SCN9A, SEMA4A, SH2B3, SIRPA, STK4, SWAP70, TLR2, TLR4, TLR7, TNFRSF1A, TYROBP, VCAN
Cancer, Gastrointestinal Disease, Organismal Injury and Abnormalities	Duodenal neoplasm	7.61E-06			22	APC, B2M, BCL7A, FCRLA, GSE1, HSP90AA1, HSP90AB1, HSP90B1, HVCN1, IRF8, KBTBD12, KRAS, MCL1, MSH6, PRKCD, PRKCG, PTEN, PTGS2, RHOA, TET2, TRAF3, U2AF1/U2AF1L5
Cancer, Organismal Injury and Abnormalities	Malignant genitourinary solid tumor	8.87E-06		0.344	1292	A1CF, ABCB5, ABCC2, ABHD3, ABHD8, ACBD3, ACO1, ACOX1, ACP3, ACSL1, ACSS3, ACTG1, ACTN1, ACTR2, ACYP1, ADAD1, ADAM10, ADAM15, ADAM17, ADGRA1, ADM, ADNP2, AGO2, AIF1, AIG1, AIPL1, AK9, AKAP12, ALDH3A1, ALDH5A1, ALDH9A1, ALG11, ALKBH1, ALKBH3, ALKBH8, ALOX5AP, ALS2, AMPH, ANAPC13, ANGPTL5, ANKFY1, ANKLE2, ANKRD13A, ANKRD13B, ANKRD42, ANKRD44, ANO5, ANO9, ANTXR2, ANXA2, ANXA3, ANXA5, AOPEP, AP1G1, AP5M1, APBA1, APBB1IP, APC, APOA1, APOA2, APOB, APOBEC3A, APOBEC3B, APOLD1, APP, AQP9, ARF4, ARHGAP19, ARHGAP25, ARHGAP27, ARHGAP29, ARHGDIB, ARHGEF25, ARID4B, ARIH2, ARL6IP1, ARMC3, ARMCX5-GPRASP2/GPRASP2, ARNT, ARNTL, ARRDC3, ARSD, ARVCF, ASB10, ASB7, ASMTL, ASXL1, ATF7IP, ATG13, ATG2B, ATG7, ATL3, ATN1, ATP6AP2, ATP6V1A, ATP6V1B2, ATP6V1H, ATP7B, ATRN, ATXN3, AURKB, B2M, B3GNTL1, BABAM2, BACH1, BARX2, BASP1, BAZ2B, BBS7, BCAS3, BCL2L11, BECN1, BGN, BLVRA, BLZF1, BMP2K, BMS1, BNIP2, BNIP3L, BNIPL, BOD1L1, BPGM, BPIFB1, BRCA1, BRD8, BRIP1, BRWD3, BTBD3, BTD, BTG2, BTK, BTN2A1, BTNL8, BUD13, C10orf71, C12orf60, C16orf70, C17orf80, C18orf25, C1GALT1C1, C1orf87, C1RL, C22orf23, C7orf25, C9orf153, C9orf64, C9orf78, CACNA1E, CALCOCO2, CALU, CAMK2A, CAMSAP2, CAP1, CAPN11, CAPZA1, CAPZB, CARD16, CARD6, CARD8, CARMIL1, CARNS1, CASC2, CASC3, CASP8, CATSPERD, CAVIN2, CBX1, CBY1, CCDC174, CCDC40, CCDC47, CCDC88A, CCL5, CCNK, CCP110, CCR1, CCT2, CD14, CD1E, CD300E, CD86, CDA, CDC25C, CDC5L, CDCP2, CDH12, CDKL1, CDKL5, CELF1, CELF2, CELSR3, CEP128, CEP63, CEP72, CETN1, CFAP161, CFAP206, CFAP58, CFAP92, CFLAR, CGB3 (includes others), CHCHD5, CHD4, CHMP2A, CHMP3, CHPF, CHST11, CKMT2, CLASP1, CLEC1B, CLEC4F, CLEC4M, CLEC9A, CLIC2, CLIC4, CLIP1, CLK2, CMSS1, CNMD, CNP, CNPY3, CNR1, COG2, COG5, COL1A2, COL7A1, COMMD2, CORO1C, CPEB1, CPN1, CPQ, CPSF4, CPSF7, CPT1A, CRB1, CREB1, CRKL, CRNN, CRY2, CRYBG3, CSDE1, CSF1R, CSF3R, CST8, CSTF1, CT45A10/CT45A5, CTAG2, CTBS, CTLA4, CTNNA1, CTNND1, CTNND2, CTSB, CTSC, CTSZ, CUX1, CXCL1, CXCL16, CXCL6, CXCL9, CXCR2, CXCR3, CYBB, CYP24A1, CYP2A6 (includes others), CYP2W1, CYP4F2, CYP4F3, CYP51A1, CYTH4, DAB2, DCLRE1C, DDC, DDIT3, DDX17, DDX21, DDX23, DDX27, DDX39A, DDX3X, DDX5, DEF6, DENND3, DENND5A, DET1, DGAT2, DGLUCY, DHCR7, DHDDS, DHRS7, DHX30, DHX38, DHX8, DIP2B, DKK3, DLG3, DLGAP4, DLX6-AS1, DNAJA2, DNAJB11, DNAJB12, DNAJB5, DNAJB6, DNAJC14, DNAJC17, DNAJC2, DNAJC7, DNM3, DOCK2, DOCK8, DOK5, DPF2, DPF3, DPH2, DPRX, DPYD, DSE, DUSP5, DYNC1LI1, DYRK1A, EBLN2, ECE1, ECPAS, EDEM3, EDRF1, EEF2K, EFCAB2, EFS, EHD3, EIF1AX, EIF1B, EIF3A, EIF3I, EIF4G3, ELAC1, ELF1, ELF3, ELK3, ELL2, ELN, ELOA, EMC10, EMSY, ENPP5, ENTPD1, ENTPD4, EOGT, EPB41L3, EPHB1, EPM2AIP1, ERCC5, ERO1A, ESS2, ETFDH, ETV3, ETV6, EVC2, EVI5L, EWSR1, EXD2, EXOC3L4, EXT1, EZR, F10, F11R, F13A1, F2R, F8, FAH, FAM126B, FAM136A, FAM13B, FAM209A, FAM214B, FAM217B, FAM72A, FASTKD2, FBLN2, FBXO33, FBXO38, FCAMR, FEZ1, FFAR4, FGD4, FGD6, FGGY, FGL2, FKBP5, FNBP1L, FOXL2, FOXO3, FOXP3, FPR1, FPR2, FRMD4B, FRS2, FTH1, FTX, FUBP3, FUS, FUT7, FYB1, FZD1, FZD3, G3BP2, GAB2, GADL1, GAL3ST1, GALC, GALNT1, GAPDH, GAS7, GASK1B, GATA5, GBE1, GC, GFOD2, GFRAL, GHITM, GIT2, GK, GLCE, GLE1, GLIPR1, GLT8D2, GLUL, GLYCTK, GLYR1, GNB4, GOLGB1, GPATCH1, GPATCH4, GPR21, GPR50, GPR75, GPRIN1, GRB2, GSTA1, GTDC1, GTF2E1, GTF2H3, GTF3C3, GTPBP1, GUCA2B, GYG1, H2AC18/H2AC19, H2BC21, H3-3A/H3-3B, H3-5, HAL, HAMP, HAUS7, HBA1/HBA2, HBB, HBP1, HCCS, HCK, HCLS1, HDAC7, HDAC9, HDLBP, HECA, HELZ, HERC3, HGD, HIC2, HLA-A, HLA-C, HLA-E, HLA-G, HMCN2, HMOX1, HNRNPA1, HNRNPA2B1, HNRNPH1, HNRNPH2, HOTAIR, HOXA10, HOXA3, HOXA4, HOXA6, HS3ST4, HSD17B12, HSP90AA1, HSP90AB1, HSP90B1, HSPA13, HSPA1A/HSPA1B, HSPA5, HSPB7, HSPD1, HTATIP2, HTR1F, HVCN1, ICAM1, IDH3A, IER2, IFI16, IFNAR1, IFNGR1, IFNGR2, IGF1, IGF1R, IGF2BP3, IGF2R, IGFBP4, IGSF6, IK, IL1B, IL1R2, IL1RN, ILF3, IMPG2, IP6K2, IQCD, IQSEC3, IRAG2, IREB2, IRX4, ITGA4, ITGAX, ITGB8, JADE1, JAK1, JAML, JMJD1C, JMJD4, JPH4, JPT1, JPX, KAT6A, KAT6B, KCNJ2, KCNJ4, KCTD20, KDM1B, KDM5A, KDM7A, KIAA0319L, KIAA0556, KIDINS220, KIF13A, KIF1A, KIF1C, KIF26B, KLF6, KLHL15, KLHL20, KMT5B, KRAS, KRT23, KRT34, KYNU, L3MBTL3, LAMA5, LAMTOR5, LARP4, LARP6, LAS1L, LASP1, LAT2, LCOR, LCP1, LCT, LEFTY1, let-7, LETM2, LGALS8, LGMN, LGR5, LHCGR, LHFPL2, LIAS, LILRA1, LILRA2, LILRB3, LIMK2, LINC00511, LINC00887, LINC01564, LIPM, LITAF, LMTK2, LONRF3, LRP2, LRP8, LRPAP1, LRRFIP1, LSM12, LSM14A, LSP1, LUCAT1, LUZP2, LY6K, LY86, LY9, LYPLA2, LYVE1, LYZ, M6PR, MAD2L1BP, MAFF, MAGT1, MAK, MAN2A2, MANBAL, MAP3K1, MAP3K2, MAP3K7CL, MAP4, MAP4K4, MAPKAP1, MAPRE1, MAPRE3, MARF1, MAX, MBP, MCCC2, MCL1, MCM7, MDM1, MDM2, MED23, MED30, MEF2C, MERTK, METTL21A, MEX3B, MGAT5, MICB, mir-101, mir-103, mir-122, mir-133, mir-138, mir-154, mir-202, mir-24, mir-26, mir-28, mir-515, MKNK1, MLX, MMP14, MOB3A, MORN5, MPHOSPH10, MPND, MPP1, MPZL3, MRPL15, MS4A1, MS4A14, MS4A4A, MS4A7, MSH6, MSMB, MSN, MT1A, MT1F, MT1X, MTCH2, MTDH, MTERF2, MTF1, MTFR2, MTHFD2, MTOR, MTRR, MTTP, MTURN,

						MUC1, MUC13, MUC15, MVP, MX2, MXD1, MYBBP1A, MYH14, MYH15, MYLK, MYLK2, MYO15A, MYO1F, MYO5A, MYO5B, MYOF, MYOG, NABP1, NACA2, NAMPT, NAP1L5, NAPB, NASP, NAT2, NCBP1, NCCRP1, NCF2, NCKAP1L, NCL, NCOA1, NCOA4, NDE1, NDEL1, NDRG4, NECAB2, NEDD9, NETO2, NFATC4, NFE2L2, NFKBIZ, NHSL1, NIM1K, NIN, NINJ1, NIPSNAP1, NLGN3, NLGN4X, NOM1, NONO, NOTCH2, NOTCH2NLA/NOTCH2NLB, NOXRED1, NPC1L1, NPL, NPTN, NR3C1, NRBF2, NRDC, NSFL1C, NSRP1, NTRK1, NUAK2, NUBP1, NUDT7, NUMB, NUP160, NUP50, NUP62, NUP93, NXPE4, NXPH4, OAT, OAZ1, OBP2A, OGFOD2, OPA1, OPN3, OR10R2, OR2A14, OR4D10, OR4K5, OR51A2, OR52L1, OR5AC2, OR5M1, OSBPL11, OTUD3, OXA1L, P2RY13, PABPC1, PACSIN2, PAK2, PAQR3, PARP8, PCLO, PCOLCE, PCYT1A, PDAP1, PDCD4, PDE4B, PDGFRA, PDIA3, PDIA5, PDK3, PDLIM5, PDS5B, PDZD8, PEAK1, PECAM1, PER2, PEX19, PF4, PGAM2, PHF12, PHLPP1, PID1, PIGO, PILRA, PIN4, PIP4P2, PIP5K1A, PITPNA, PITPNM1, PITX2, PIWIL1, PJA2, PKN3, PLAGL2, PLB1, PLCB3, PLCG2, PLCL1, PLEK, PLEKHA5, PLEKHA7, PLEKHM3, PLP1, PLXDC2, PLXNA4, PNN, PODNL1, POLD3, POLR1A, POTEH (includes others), POU4F2, PPHLN1, PPIF, PPM1D, PPP1CB, PPP1R12B, PPP1R17, PPP1R3B, PPP4R2, PPP6R3, PQBP1, PRC1, PRKAA1, PRKAR1A, PRKCD, PRKCG, PRKG1, PRL, PRPF38A, PRPF6, PRR12, PRRC2C, PRSS55, PSAP, PSMC1, PSMC2, PSMD1, PSMD12, PSMD2, PSMD4, PSMD7, PSME3, PTCD3, PTCHD4, PTEN, PTF1A, PTGFRN, PTGS2, PTPRE, PUDP, PURA, PUS3, PWWP3A, RAB27A, RAB31, RAB3GAP2, RAB9A, RABEP1, RABGAP1L, RAC2, RAD51C, RAD51D, RAF1, RALB, RALBP1, RALGPS1, RALGPS2, RAMP2, RAP1A, RAPGEF2, RASEF, RASSF2, RBM12B, RBM25, RBM4, RBM47, RBM5, RBMS1, RBMXL3, RCBTB2, RCC2, RCOR3, REEP5, RFPL2, RFX3, RGCC, RGS2, RHBG, RHOA, RHOB, RICTOR, RIIAD1, RIN2, RIOK1, RIOK2, RIOK3, RIOX2, RIPK3, RNF103, RNF103-CHMP3, RNF121, RNF130, RNF149, RNF169, RNF20, RNF40, RPF2, RPGRIP1, RPL28, RPL39, RPL4, RPL5, RPS15, RPS6KA5, RRNAD1, RSPH3, RTCB, RTF1, RTN3, RTN4, RTTN, RUSC1, S100A14, S100A9, S100Z, SAAL1, SAMD4B, SART3, SAT1, SAV1, SBF2, SCARB2, SCFD2, SCG3, SCN9A, SCP2D1, SCRIB, SCRN1, SCRT2, SDCBP, SEC13, SEC14L1, SEC24D, SEC61A2, SEL1L, SEMA3G, SENP2, SENP5, SERHL2, SERPINB3, SERPINB4, SERPINB8, SESTD1, SETDB1, SF3A3, SF3B1, SF3B6, SFRP4, SGK1, SH2B3, SH3BP2, SHISAL2B, SHROOM3, SIAH1, SIPA1L2, SIRPA, SIRPB1, SKP2, SLC16A11, SLC22A15, SLC22A18, SLC22A4, SLC22A5, SLC24A4, SLC25A2, SLC25A3, SLC25A32, SLC26A11, SLC31A1, SLC35F4, SLC36A1, SLC43A3, SLC49A4, SLC4A1AP, SLC4A2, SLC6A6, SLC7A7, SLC8A1, SLC8A3, SLC9A9, SLITRK6, SLU7, SMARCA2, SMARCC2, SMS, SMTN, SNAP91, SNRPB, SNRPF, SNX13, SNX27, SOCS4, SOD2, SORL1, SOS2, SP1, SP100, SP110, SP3, SPACA5/SPACA5B, SPAG9, SPAST, SPATA31A6 (includes others), SPATA5, SPEF2, SPHK2, SPINK5, SPOP, SPOUT1, SPRY3, SPTBN4, SPTSSB, SQOR, SRD5A2, SRPK1, SRPK2, SRSF3, SRSF4, SRSF5, SSBP2, SSH1, SSH3, SSR1, ST6GALNAC2, ST8SIA4, STARD8, STAT3, STAU1, STEAP4, STK24, STK4, STX3, STXBP6, SUFU, SUSD6, SVIL, SWAP70, SWT1, SYK, SYNE4, SYT17, SZT2, TACC1, TAF1, TAF7, TAGLN2, TALDO1, TASP1, TBC1D12, TBC1D14, TBC1D8, TBC1D9, TBL1X, TBX5, TCAIM, TCF4, TCP1, TDGF1, TDP2, TDRD1, TEKT4, TERF2IP, TET2, TFRC, TGOLN2, THBS2, THEG, THEMIS2, THOC5, THRAP3, TIFA, TIGAR, TIGD1, TIMMDC1, TJP1, TKFC, TLK2, TLR2, TLR4, TLR5, TLR7, TM2D2, TM6SF2, TM7SF3, TMEM140, TMEM185B, TMEM43, TMEM70, TMEM86A, TMPRSS7, TMTC2, TNFRSF10D, TNFRSF1A, TNFSF10, TNIP1, TNNC1, TNNI3K, TOPORS, TOR1B, TOX4, TPM3, TRAF3, TRAF3IP1, TRANK1, TREML2, TRIM34, TRIM41, TRIM43/TRIM43B, TRIM46, TRIM5, TRIM55, TRIM64C, TRIM65, TRIO, TRIOBP, TRIP10, TRIP12, TRIP4, TRMT1, TRMT9B, TRNT1, TRPM6, TSG101, TSHZ3, TSPAN1, TTC13, TTC17, TTC26, TTF1, TTI2, TUBA1A, TUBA1B, TUBA1C, TUBB2A, TUBGCP3, TUT7, U2AF1/U2AF1L5, UBAP2L, UBE2E3, UBE2F, UBE2J2, UBE3B, UBE4B, UBR2, UCK2, UEVLD, UGT2B11, UGT3A1, UNC5C, UQCRC2, USP15, USP19, USP32, USP4, UTP14A, UTP23, UTP4, VCAN, VCPIP1, VDAC2, VDR, VIM, VKORC1L1, VMP1, VNN2, VPS26C, VRK3, VTCN1, VTI1B, WASF2, WASF3, WDFY3, WDR19, WDR33, WIPF1, WNK1, WNK3, WSB1, WWTR1, XAF1, XPNPEP3, XRCC5, YBX1, YBX3, YPEL3, YPEL5, YWHAE, YWHAZ, ZAN, ZBTB21, ZEB2, ZFPM2, ZFYVE21, ZMAT2, ZMPSTE24, ZMYM3, ZNF10, ZNF134, ZNF143, ZNF148, ZNF165, ZNF17, ZNF180, ZNF189, ZNF195, ZNF200, ZNF212, ZNF217, ZNF224, ZNF229, ZNF235, ZNF24, ZNF257, ZNF267, ZNF281, ZNF283, ZNF287, ZNF3, ZNF320, ZNF333, ZNF33A, ZNF33B, ZNF34, ZNF347, ZNF350, ZNF398, ZNF41, ZNF429, ZNF431, ZNF443, ZNF45, ZNF461, ZNF469, ZNF493, ZNF516, ZNF518A, ZNF525, ZNF528, ZNF534, ZNF548, ZNF555, ZNF558, ZNF565, ZNF567, ZNF568, ZNF570, ZNF585B, ZNF606, ZNF610, ZNF613, ZNF615, ZNF616, ZNF649, ZNF667, ZNF677, ZNF684, ZNF700, ZNF711, ZNF714, ZNF717, ZNF721, ZNF738, ZNF746, ZNF761, ZNF776, ZNF781, ZNF799, ZNF81, ZNF829, ZNF836, ZNF880, ZNF93, ZNFX1, ZRANB1, ZRSR2, ZSCAN2, ZXDC
Cellular Movement	Cellular infiltration	9.54E-06	Decreased	-2.447	112	ACO2, ADAM17, ADM, ALOX5AP, ANXA2, ANXA3, APC, APOA1, APP, ARHGAP25, ATG7, B4GALT1, BECN1, BGN, BID, CASP8, CAV3, CCDC88A, CCL5, CCR1, CD14, CD86, CD93, CHD4, CNP, CNR1, CSF1R, CTLA4, CTSB, CTSC, CUX1, CX3CR1, CXCL16, CXCL9, CXCR2, CXCR3, CYBB, CYP2J2, DEF6, DOCK2, EFS, EPO, EZR, F2R, FGL2, FOXP3, FPR1, FPR2, FUT7, GAL3ST1, GAPDH, HAMP, HCK, HLA-A, HMOX1, HSPA5, HSPD1, ICAM1, IFNGR1, IL1B, IL1RN, IRF8, ITGA4, KCNE3, KRAS, LCP2, LILRB3, LTBR, LYN, MBP, MGAT5, MMP14, MYLK, NFE2L2, NFKBIZ, NINJ1, NR3C1, OPA1, PDIA3, PF4, PLCB3, PLP1, PPM1D, PRKAA1, PRKCD, PRKG1, PSMB8, PTEN, PTGS2, PTMA, PTPN6, RAB27A, RAC2, RGCC, RIPK2, RPL13A, S100A9, SGK1, STAT3, TET2, THBS2, TLR2, TLR4, TLR7, TNFRSF1A, TNFSF10, TNFSF4, TNIP1, TREM1, VDR, VTCN1, YBX1
Cell-To-Cell Signaling and Interaction, Cellular Function and Maintenance,	Phagocytosis of leukocytes	9.65E-06	Decreased	-3.603	44	ANXA5, APOA1, APOA2, APP, ATG7, BECN1, BTK, CD14, CD93, CEACAM3, CLCN3, CLEC4M, CLEC6A, CLIC4, CSF1R, DEF6, DOCK2, FCGR2A, FPR1, GAB2, HCK, HMOX1, ICAM1, IFNAR1, IL1B, let-7, MERTK, MEX3B, mir-24, NCKAP1L, PF4, PTEN, RAB11A, RGCC, S100A9, SH3BP2, SIRPA, SIRPB1, SLAMF7, SYK, TLR2, TLR4, TREML2, TYROBP

Inflammatory Response						
Cellular Function and Maintenance	Engulfment of tumor cell lines	1.02E-05	Decreased	-3.991	56	ACTR2, APC, APLP2, APPL2, ARPC2, ATP6V1A, ATP6V1B2, BECN1, BTK, CCL5, CD93, CDC5L, CLIP1, DAB2, DEF6, DET1, DOCK2, EZR, FCGR2A, FRS2, GRB2, HCK, HMOX1, HSP90AA1, ICAM1, JAK1, KAT6A, KCTD5, KRAS, LRPAP1, MERTK, NCKAP1L, NCL, NTRK1, PDZD8, PIP5K1A, PLEK, PRKCD, PSMD4, PTPN6, RAB11A, RAB31, RALB, RHOA, RHOB, RIT1, SCARB2, SLAMF7, SRSF3, TM2D2, TNFSF10, TYROBP, UBE2L3, VIM, WASF2, ZNF217
Nervous System Development and Function	Neuroprotection of cerebral cortex cells	1.11E-05		-0.575	11	APP, CNR1, DDIT3, EPO, IGF1, NFATC4, PHLPP1, PTGS2, STAT3, STIP1, WDFY3
Cellular Movement	Homing of cells	1.12E-05	Decreased	-6.075	129	ACTN1, ACTR2, ADAM10, ADAM17, ADGRE2, AIF1, AKAP12, ANXA2, APOA1, APP, AQP9, ARHGAP25, ARPC2, B4GALT1, CAMK1D, CCDC88A, CCL23, CCL5, CCR1, CCR10, CLCN3, CNR1, CRKL, CSF1R, CSF3R, CUX1, CX3CR1, CXCL1, CXCL16, CXCL6, CXCL9, CXCR2, CXCR3, CYBB, DEFB103A/DEFB103B, DOCK2, ELN, EPHB1, F10, F2R, FCGR2A, FOXP3, FPR1, FPR2, FRS2, FUT7, FYB1, GAB1, GIT2, GRB2, HCK, HCLS1, HEBP1, HLA-G, HSPD1, ICAM1, IGF1, IGF1R, IGF2R, IL1B, ITGA4, JAK1, JAML, LCP1, LGMN, LILRB3, LITAF, LRP2, LSP1, LTBR, LYN, MAP3K1, MAPKAP1, MEF2C, mir-154, mir-24, MMP14, MPP1, MTOR, MUC1, MYLK, MYO1F, MYO5B, NCKAP1L, NEDD9, NINJ1, NR3C1, PDE4B, PDGFRA, PF4, PIP5K1A, PLCG2, PRKCD, PRKCG, PRKG1, PROK2, PTEN, PTGS2, PTPN6, RAC2, RALBP1, RHOA, RHOB, RICTOR, RPL13A, RTN4, S100A14, S100A9, SCN9A, SCRIB, SIRPA, SPHK2, STAT3, STK4, STX3, SWAP70, SYK, TAFA4, THBS2, TLR2, TLR4, TNFRSF1A, TNFSF14, TREM1, TREML2, TRIP10, WARS1, WIPF1, WNK1
Cancer, Organismal Injury and Abnormalities	Genitourinary tumor	1.33E-05		0.167	1309	A1CF, ABCB5, ABCC2, ABHD3, ABHD8, ACBD3, ACO1, ACOX1, ACP3, ACSL1, ACSS3, ACTG1, ACTN1, ACTR2, ACYP1, ADAD1, ADAM10, ADAM15, ADAM17, ADGRA1, ADM, ADNP2, AGO2, AIF1, AIG1, AIPL1, AK9, AKAP12, AKAP8L, ALDH3A1, ALDH5A1, ALDH9A1, ALG11, ALKBH1, ALKBH3, ALKBH8, ALOX5AP, ALS2, AMPH, ANAPC13, ANGPTL5, ANKFY1, ANKLE2, ANKRD13A, ANKRD13B, ANKRD42, ANKRD44, ANO5, ANO9, ANTXR2, ANXA2, ANXA3, ANXA5, AOPEP, AP1G1, AP5M1, APBA1, APBB1IP, APC, APOA1, APOA2, APOB, APOBEC3A, APOBEC3B, APOLD1, APP, AQP9, ARF4, ARHGAP19, ARHGAP25, ARHGAP26, ARHGAP27, ARHGAP29, ARHGDIB, ARHGEF25, ARID4B, ARIH2, ARL6IP1, ARMC3, ARMCX5-GPRASP2/GPRASP2, ARNT, ARNTL, ARRDC3, ARSD, ARVCF, ASB10, ASB7, ASMTL, ASXL1, ATF7IP, ATG13, ATG2B, ATG7, ATL3, ATN1, ATP6AP2, ATP6V1A, ATP6V1B2, ATP6V1H, ATP7B, ATRN, ATXN3, AURKB, B2M, B3GNTL1, BABAM2, BACH1, BARX2, BASP1, BAZ2B, BBS7, BCAS3, BCL2L11, BECN1, BGN, BLVRA, BLZF1, BMP2K, BMS1, BNIP2, BNIP3L, BNIPL, BOD1L1, BPGM, BPIFB1, BRCA1, BRD8, BRIP1, BRWD3, BTBD3, BTD, BTG2, BTK, BTN2A1, BTNL8, BUD13, C10orf71, C12orf60, C16orf70, C17orf80, C18orf25, C1GALT1C1, C1orf87, C1RL, C22orf23, C7orf25, C9orf153, C9orf64, C9orf78, CACNA1E, CALCOCO2, CALU, CAMK2A, CAMSAP2, CAP1, CAPN11, CAPZA1, CAPZB, CARD16, CARD6, CARD8, CARMIL1, CARNS1, CASC2, CASC3, CASP8, CATSPERD, CAVIN2, CBX1, CBY1, CCDC174, CCDC40, CCDC47, CCDC88A, CCL5, CCNK, CCP110, CCR1, CCT2, CD14, CD1E, CD300E, CD86, CDA, CDC25C, CDC5L, CDCP2, CDH12, CDKL1, CDKL5, CELF1, CELF2, CELSR3, CEP128, CEP63, CEP72, CETN1, CFAP161, CFAP206, CFAP58, CFAP92, CFLAR, CGA, CGB3 (includes others), CHCHD5, CHD4, CHM, CHMP2A, CHMP3, CHPF, CHST11, CKMT2, CLASP1, CLEC1B, CLEC4F, CLEC4M, CLEC9A, CLIC2, CLIC4, CLIP1, CLK2, CMSS1, CNMD, CNP, CNPY3, CNR1, COG2, COG5, COL1A2, COL7A1, COMMD2, CORO1C, CPEB1, CPN1, CPQ, CPSF4, CPSF7, CPT1A, CRB1, CREB1, CRKL, CRNN, CRY2, CRYBG3, CSDE1, CSF1R, CSF3R, CST8, CSTF1, CT45A10/CT45A5, CTAG2, CTBS, CTLA4, CTNNA1, CTNND1, CTNND2, CTSB, CTSC, CTSZ, CUX1, CXCL1, CXCL16, CXCL6, CXCL9, CXCR2, CXCR3, CYBB, CYP24A1, CYP2A6 (includes others), CYP2W1, CYP4F2, CYP4F3, CYP51A1, CYTH4, DAB2, DCLRE1C, DDC, DDIT3, DDX17, DDX21, DDX23, DDX27, DDX39A, DDX3X, DDX5, DEF6, DENND3, DENND5A, DET1, DGAT2, DGLUCY, DHCR7, DHDDS, DHRS7, DHX30, DHX38, DHX8, DIP2B, DKK3, DLG3, DLGAP4, DLX6-AS1, DMTF1, DNAJA2, DNAJB11, DNAJB12, DNAJB5, DNAJB6, DNAJC14, DNAJC17, DNAJC2, DNAJC7, DNM3, DOCK2, DOCK8, DOK5, DPF2, DPF3, DPH2, DPRX, DPYD, DSE, DUSP5, DYNC1LI1, DYRK1A, EBLN2, ECE1, ECPAS, EDEM3, EDRF1, EEF2K, EFCAB2, EFS, EHD3, EIF1AX, EIF1B, EIF3A, EIF3I, EIF4G3, ELAC1, ELF1, ELF3, ELK3, ELL2, ELN, ELOA, EMC10, EMSY, ENPP5, ENTPD1, ENTPD4, EOGT, EPB41L3, EPHB1, EPM2AIP1, EPO, ERCC5, ERO1A, ESS2, ETFDH, ETV3, ETV6, EVC2, EVI5L, EWSR1, EXD2, EXOC3L4, EXT1, EZR, F10, F11R, F13A1, F2R, F8, FAH, FAM126B, FAM136A, FAM13B, FAM209A, FAM214B, FAM217B, FAM72A, FASTKD2, FBLN2, FBXO33, FBXO38, FCAMR, FEZ1, FFAR4, FGD4, FGD6, FGGY, FGL2, FKBP5, FNBP1L, FOXL2, FOXO3, FOXP3, FPR1, FPR2, FRMD4B, FRS2, FTH1, FTX, FUBP3, FUS, FUT7, FYB1, FZD1, FZD3, G3BP2, GAB2, GADL1, GAL3ST1, GALC, GALNT1, GAPDH, GAS7, GASK1B, GATA5, GBE1, GC, GFOD2, GFRAL, GHITM, GIT2, GK, GLCE, GLE1, GLIPR1, GLT8D2, GLUL, GLYCTK, GLYR1, GNB4, GOLGB1, GPATCH1, GPATCH4, GPR21, GPR50, GPR75, GPRIN1, GRB2, GSTA1, GTDC1, GTF2E1, GTF2H3, GTF3C3, GTPBP1, GUCA2B, GYG1, H2AC18/H2AC19, H2BC21, H3-3A/H3-3B, H3-5, HAL, HAMP, HAUS7, HBA1/HBA2, HBB, HBP1, HCCS, HCK, HCLS1, HDAC7, HDAC9, HDLBP, HECA, HELZ, HERC3, HGD, HIC2, HLA-A, HLA-C, HLA-E, HLA-G, HMCN2, HMOX1, HNMT, HNRNPA1, HNRNPA2B1, HNRNPH1, HNRNPH2, HOTAIR, HOXA10, HOXA3, HOXA4, HOXA6, HS3ST4, HSD17B12, HSP90AA1, HSP90AB1, HSP90B1, HSPA13, HSPA1A/HSPA1B, HSPA5, HSPB7, HSPD1, HTATIP2, HTR1F, HVCN1, ICAM1, IDH3A, IER2, IFI16, IFNAR1, IFNGR1, IFNGR2, IGF1, IGF1R, IGF2BP3, IGF2R, IGFBP4, IGSF6, IK, IL1B, IL1R2, IL1RN, ILF3, IMPG2, IP6K2, IQCD, IQSEC3, IRAG2, IREB2, IRX4, ITGA4, ITGAX, ITGB8, JADE1, JAK1, JAML, JMJD1C, JMJD4, JPH4, JPT1, JPX, KAT6A, KAT6B, KCNJ2, KCNJ4, KCTD20, KDM1B, KDM5A, KDM7A, KIAA0319L, KIAA0556, KIDINS220, KIF13A, KIF1A, KIF1C, KIF26B, KLF6, KLHL15, KLHL20, KMT5B, KRAS, KRT23, KRT34, KYNU,

						L3MBTL3, LAMA5, LAMTOR5, LARP4, LARP6, LAS1L, LASP1, LAT2, LCOR, LCP1, LCT, LEFTY1, let-7, LETM2, LGALS8, LGMN, LGR5, LHCGR, LHFPL2, LIAS, LILRA1, LILRA2, LILRB3, LIMK2, LINC00511, LINC00887, LINC01564, LIPM, LITAF, LMTK2, LONRF3, LRP2, LRP8, LRPAP1, LRRFIP1, LSM12, LSM14A, LSP1, LUCAT1, LUZP2, LY6K, LY86, LY9, LYN, LYPLA2, LYVE1, LYZ, M6PR, MAD2L1BP, MAFF, MAGT1, MAK, MAN2A2, MANBAL, MAP3K1, MAP3K2, MAP3K7CL, MAP4, MAP4K4, MAPKAP1, MAPRE1, MAPRE3, MARF1, MAX, MBP, MCCC2, MCL1, MCM7, MDM1, MDM2, MED23, MED30, MEF2C, MERTK, METTL21A, MEX3B, MGAT5, MICB, mir-101, mir-103, mir-122, mir-1260a, mir-133, mir-138, mir-154, mir-202, mir-24, mir-26, mir-28, mir-515, mir-551, MKNK1, MLX, MMP14, MOB3A, MORN5, MPHOSPH10, MPND, MPP1, MPZL3, MRPL15, MS4A1, MS4A14, MS4A4A, MS4A7, MSH6, MSMB, MSN, MT1A, MT1F, MT1X, MTCH2, MTDH, MTERF2, MTF1, MTFR2, MTHFD2, MTOR, MTRR, MTTP, MTURN, MUC1, MUC13, MUC15, MVP, MX2, MXD1, MYBBP1A, MYH14, MYH15, MYLK, MYLK2, MYO15A, MYO1F, MYO5A, MYO5B, MYOF, MYOG, NABP1, NACA2, NAMPT, NAP1L5, NAPB, NASP, NAT2, NCBP1, NCCRP1, NCF2, NCKAP1L, NCL, NCOA1, NCOA4, NDE1, NDEL1, NDRG4, NECAB2, NEDD9, NETO2, NFATC4, NFE2L2, NFKBIZ, NHSL1, NIM1K, NIN, NINJ1, NIPSNAP1, NLGN3, NLGN4X, NOM1, NONO, NOTCH2, NOTCH2NLA/NOTCH2NLB, NOXRED1, NPC1L1, NPL, NPTN, NR3C1, NRBF2, NRDC, NSFL1C, NSRP1, NTRK1, NUAK2, NUBP1, NUDT7, NUMB, NUP160, NUP50, NUP62, NUP93, NXPE4, NXPH4, OAT, OAZ1, OBP2A, OGFOD2, OPA1, OPN3, OR10R2, OR2A14, OR4D10, OR4K5, OR51A2, OR52L1, OR5AC2, OR5M1, OSBPL11, OTUD3, OXA1L, P2RY13, PABPC1, PACSIN2, PAK2, PAQR3, PARP8, PCLO, PCOLCE, PCYT1A, PDAP1, PDCD4, PDE4B, PDGFRA, PDIA3, PDIA5, PDK3, PDLIM5, PDS5B, PDZD8, PEAK1, PECAM1, PER2, PEX19, PF4, PGAM2, PHF12, PHLPP1, PID1, PIGO, PILRA, PIN4, PIP4P2, PIP5K1A, PITPNA, PITPNM1, PITX2, PIWIL1, PJA2, PKN3, PLAGL2, PLB1, PLCB3, PLCG2, PLCL1, PLEK, PLEKHA5, PLEKHA7, PLEKHM3, PLP1, PLXDC2, PLXNA4, PNN, PODNL1, POLD3, POLR1A, POTEH (includes others), POU4F2, PPHLN1, PPIF, PPM1D, PPP1CB, PPP1R12B, PPP1R17, PPP1R3B, PPP4R2, PPP6R3, PQBP1, PRC1, PRKAA1, PRKAR1A, PRKCD, PRKCG, PRKG1, PRL, PRPF38A, PRPF6, PRR12, PRRC2C, PRSS55, PSAP, PSMC1, PSMC2, PSMD1, PSMD10, PSMD12, PSMD2, PSMD4, PSMD7, PSME3, PTCD3, PTCHD4, PTEN, PTF1A, PTGFRN, PTGS2, PTPRE, PUDP, PURA, PUS3, PWWP3A, RAB27A, RAB31, RAB3GAP2, RAB9A, RABEP1, RABGAP1L, RAC2, RAD51C, RAD51D, RAF1, RALB, RALBP1, RALGPS1, RALGPS2, RAMP2, RAP1A, RAPGEF2, RASEF, RASSF2, RBM12B, RBM25, RBM4, RBM47, RBM5, RBMS1, RBMXL3, RCBTB2, RCC2, RCOR3, REEP5, RFPL2, RFX3, RGCC, RGS2, RHBG, RHOA, RHOB, RICTOR, RIIAD1, RIN2, RIOK1, RIOK2, RIOK3, RIOX2, RIPK3, RNF103, RNF103-CHMP3, RNF121, RNF130, RNF149, RNF169, RNF20, RNF40, RPF2, RPGRIP1, RPL28, RPL39, RPL4, RPL5, RPS15, RPS6KA5, RRNAD1, RSPH3, RTCB, RTF1, RTN3, RTN4, RTTN, RUSC1, RYBP, S100A14, S100A9, S100Z, SAAL1, SAMD4B, SART3, SAT1, SAV1, SBF2, SCARB2, SCFD2, SCG3, SCN9A, SCP2D1, SCRIB, SCRN1, SCRT2, SDCBP, SEC13, SEC14L1, SEC24D, SEC61A2, SEL1L, SEMA3G, SENP2, SENP5, SERHL2, SERPINB3, SERPINB4, SERPINB8, SESTD1, SETDB1, SF3A3, SF3B1, SF3B6, SFRP4, SGK1, SH2B3, SH3BP2, SHISAL2B, SHROOM3, SIAH1, SIPA1L2, SIRPA, SIRPB1, SKP2, SLC16A11, SLC22A15, SLC22A18, SLC22A4, SLC22A5, SLC24A4, SLC25A2, SLC25A3, SLC25A32, SLC26A11, SLC31A1, SLC35F4, SLC36A1, SLC43A3, SLC49A4, SLC4A1AP, SLC4A2, SLC6A6, SLC7A7, SLC8A1, SLC8A3, SLC9A9, SLITRK6, SLU7, SMARCA2, SMARCC2, SMS, SMTN, SNAP91, SNRPB, SNRPF, SNX13, SNX27, SOCS4, SOD2, SORL1, SOS2, SP1, SP100, SP110, SP3, SPACA5/SPACA5B, SPAG9, SPAST, SPATA31A6 (includes others), SPATA5, SPEF2, SPHK2, SPINK5, SPOP, SPOUT1, SPRY3, SPTBN4, SPTSSB, SQOR, SRD5A2, SRPK1, SRPK2, SRSF3, SRSF4, SRSF5, SSBP2, SSH1, SSH3, SSR1, ST6GALNAC2, ST8SIA4, STAMBP, STARD8, STAT3, STAU1, STEAP4, STIP1, STK24, STK4, STX3, STXBP6, SUFU, SUSD6, SVIL, SWAP70, SWT1, SYK, SYNE4, SYT17, SZT2, TACC1, TAF1, TAF7, TAGLN2, TALDO1, TASP1, TBC1D12, TBC1D14, TBC1D8, TBC1D9, TBL1X, TBX5, TCAIM, TCF4, TCP1, TDGF1, TDP2, TDRD1, TEKT4, TERF2IP, TET2, TFRC, TGOLN2, THBS2, THEG, THEMIS2, THOC5, THRAP3, TIFA, TIGAR, TIGD1, TIMMDC1, TJP1, TKFC, TLK2, TLR2, TLR4, TLR5, TLR7, TM2D2, TM6SF2, TM7SF3, TMEM140, TMEM185B, TMEM43, TMEM70, TMEM86A, TMPRSS7, TMTC2, TNFRSF10D, TNFRSF1A, TNFSF10, TNIP1, TNNC1, TNNI3K, TNPO3, TOPORS, TOR1B, TOX4, TPM3, TRAF3, TRAF3IP1, TRANK1, TREML2, TRIM34, TRIM41, TRIM43/TRIM43B, TRIM46, TRIM5, TRIM55, TRIM64C, TRIM65, TRIO, TRIOBP, TRIP10, TRIP12, TRIP4, TRMT1, TRMT9B, TRNT1, TRPM6, TSG101, TSHZ3, TSPAN1, TTC13, TTC17, TTC26, TTF1, TTI2, TUBA1A, TUBA1B, TUBA1C, TUBB2A, TUBGCP3, TUT7, U2AF1/U2AF1L5, UBAP2L, UBE2B, UBE2E3, UBE2F, UBE2J2, UBE3B, UBE4B, UBR2, UCK2, UEVLD, UGT2B11, UGT3A1, UNC5C, UQCRC2, USP15, USP19, USP32, USP4, UTP14A, UTP23, UTP4, VCAN, VCPIP1, VDAC2, VDR, VIM, VKORC1L1, VMP1, VNN2, VPS26C, VRK3, VTCN1, VTI1B, WASF2, WASF3, WDFY3, WDR19, WDR33, WIPF1, WNK1, WNK3, WSB1, WWTR1, XAF1, XPNPEP3, XRCC5, YBX1, YBX3, YPEL3, YPEL5, YWHAE, YWHAZ, ZAN, ZBTB21, ZDHHC17, ZEB2, ZFPM2, ZFYVE21, ZMAT2, ZMPSTE24, ZMYM3, ZNF10, ZNF134, ZNF143, ZNF148, ZNF165, ZNF17, ZNF180, ZNF189, ZNF195, ZNF200, ZNF212, ZNF217, ZNF224, ZNF229, ZNF235, ZNF24, ZNF257, ZNF267, ZNF281, ZNF283, ZNF287, ZNF3, ZNF320, ZNF333, ZNF33A, ZNF33B, ZNF34, ZNF347, ZNF350, ZNF398, ZNF41, ZNF429, ZNF431, ZNF443, ZNF45, ZNF461, ZNF469, ZNF493, ZNF516, ZNF518A, ZNF525, ZNF528, ZNF534, ZNF548, ZNF555, ZNF558, ZNF565, ZNF567, ZNF568, ZNF570, ZNF585B, ZNF606, ZNF610, ZNF613, ZNF615, ZNF616, ZNF649, ZNF667, ZNF677, ZNF684, ZNF700, ZNF711, ZNF714, ZNF717, ZNF721, ZNF738, ZNF746, ZNF761, ZNF776, ZNF781, ZNF799, ZNF81, ZNF829, ZNF836, ZNF880, ZNF93, ZNFX1, ZRANB1, ZRSR2, ZSCAN2, ZXDC
Cellular Movement	Cell tethering or rolling of leukocytes	1.60E-05	Decreased	-2.57	24	ADAM17, ARHGAP25, ARNTL, BTK, CD14, CHST1, CXCL1, CXCR2, FCGR2A, FPR2, FUT7, FYB1, GALNT1, HCK, ICAM1, IL1B, ITGA4, LCP2, LYN, MGAT5, RAC2, ST3GAL6, SWAP70, TLR4

Inflammatory Response	Inflammatory response	1.71E-05	Decreased	-3.781	174	ADAM10, ADAM17, ADM, AIF1, ALOX5AP, ALS2, ANGPTL4, ANXA2, APOA1, APP, AQP9, AREL1, ARHGAP25, ARIH2, ATG7, B4GALT1, BCL2L11, BID, CAMK1D, CASP8, CCDC88A, CCL23, CCL5, CCR1, CCR10, CD14, CD84, CEACAM3, CLEC4M, CLEC7A, CSF1R, CSF3R, CTSB, CUX1, CX3CR1, CXCL1, CXCL16, CXCL6, CXCL9, CXCR2, CXCR3, CYBB, CYP26B1, CYP4F3, DDIT3, DEFB103A/DEFB103B, DEFB114, DOCK2, EFS, ELF3, ELN, EPO, F11R, F2R, FCGR2A, FFAR4, FOXO3, FOXP3, FPR1, FPR2, FUT7, GC, GIT2, GPRC5B, HCK, HDAC7, HDAC9, HEBP1, HLA-A, HLA-G, HMOX1, HSPD1, ICAM1, IFNAR1, IGF1, IL1B, IL1RN, IL22RA2, ITGA4, JAML, KCNE3, LCP1, let-7, LGMN, LIAS, LILRA2, LILRB3, LITAF, LSP1, LYN, LYZ, MACIR, MAPKAP1, MCL1, MEFV, mir-138, mir-657, MMP14, MPP1, MUC1, MYLK, MYO1F, NCKAP1L, NCL, NEDD9, NFATC4, NFE2L2, NFKBIZ, NINJ1, NPW, NR1D2, NR3C1, OTULIN, PARK7, PDCD4, PDE4B, PECAM1, PF4, PLCG2, PLP1, PPM1D, PRKCD, PRKCG, PRKG1, PTEN, PTGS2, PTPN6, RAB27A, RAC2, RALB, RGMA, RHOA, RHOB, RICTOR, RIOX2, RIPK2, RIPK3, RPL13A, S100A14, S100A9, SCN9A, SIGLEC9, SIRPA, SLC11A1, SMAD1, SPHK2, STAT3, STK4, SWAP70, SYK, TAFA4, TBK1, TBXAS1, THBS2, TLR2, TLR4, TLR5, TLR7, TNFRSF1A, TNFSF14, TNFSF4, TNIP1, TRAF3, TREM1, TREML2, TRNT1, TUBA1A, TUBA1C, TUBB2A, TYROBP, VPS35, VTCN1, WFDC1, WIPF1
Cell Death and Survival	Cell death of colorectal cancer cell lines	1.77E-05		1.186	75	ADAM17, ADIPOR1, APC, ATG7, BCL2L11, BECN1, BID, BTK, CASP8, CD14, CFLAR, CNR1, DDIT3, DFFA, EIF1AX, EZR, FOXO3, GLIPR1, GSTA1, GUCA2A, GUCA2B, HMOX1, HOTAIR, HSPD1, IGF1, IGF1R, IGF2R, IL1B, IP6K2, IRF8, KRAS, LGALS8, LGR5, LIMS1, LTBR, LUCAT1, MCL1, mir-154, mir-515, MLKL, MT1F, MTOR, MUC1, NFE2L2, PARK7, PDE4B, PECAM1, PHLPP1, PRKCD, PRKG1, PTEN, PTGS2, RAF1, RASSF3, RHOA, RICTOR, SGK1, SOD2, SPHK2, SRPK1, STAU1, TCF4, TLR4, TM9SF4, TNFRSF10C, TNFRSF1A, TNFSF10, TNFSF14, TRAF3, TXNRD1, VDAC1, VDR, VPS35, XRCC5, YWHAE
Cell-To-Cell Signaling and Interaction, Cellular Function and Maintenance, Inflammatory Response	Phagocytosis of phagocytes	1.80E-05	Decreased	-3.405	42	ANXA5, APOA1, APOA2, APP, ATG7, BECN1, BTK, CD14, CD93, CEACAM3, CLCN3, CLEC4M, CLEC6A, CLIC4, CSF1R, DEF6, DOCK2, FCGR2A, GAB2, HCK, HMOX1, ICAM1, IFNAR1, IL1B, let-7, MERTK, MEX3B, mir-24, NCKAP1L, PF4, PTEN, RAB11A, RGCC, S100A9, SH3BP2, SIRPA, SIRPB1, SLAMF7, SYK, TLR2, TLR4, TYROBP
Free Radical Scavenging	Synthesis of reactive oxygen species	2.39E-05	Decreased	-3.846	112	ACOX1, ADGRE2, ALDH3A2, ALS2, ANXA2, AOPEP, APOA1, APP, ARHGDIB, ARNT, ATG7, ATP6AP2, BECN1, BID, BNIP3L, BRCA1, CASP8, CCL5, CD14, CLCN3, CLEC7A, CTLA4, CXCL9, CYBB, CYP2A6 (includes others), DDIT3, DOCK2, ENTPD1, EPO, ERO1A, F2R, FCGR2A, FOXO3, FPR1, FPR2, FTH1, FTL, GAB2, HBA1/HBA2, HBB, HCK, HMOX1, HSP90AB1, HVCN1, ICAM1, IGF1, IL1B, ITGAX, ITM2B, JAK1, KRAS, LCP2, let-7, LYN, MLKL, MMP14, MS4A1, MTOR, MUC1, MYLK, NAMPT, NCF2, NDUFS1, NFE2L2, NTRK1, PARK7, PCK1, PECAM1, PGAM2, PLAGL2, PLCB3, PLCG2, PRKAA1, PRKCD, PSMB8, PTEN, PTGS2, PTPN6, RAC2, RAP1A, RBPJ, RHOA, RIPK3, RTN4, SAT1, SIGLEC9, SLC8A1, SLU7, SNAP23, SOD2, SPAG9, STAT3, SYK, TAFA4, TAZ, TFRC, TIGAR, TLR2, TLR4, TLR5, TLR7, TMBIM6, TNFRSF1A, TNFSF14, TRAF3, TREML2, TXNRD1, TYROBP, UQCRC2, VDAC1, VDR, YWHAZ
Cellular Movement	Chemotaxis	2.71E-05	Decreased	-5.536	121	ACTN1, ADAM10, ADAM17, ADGRE2, AIF1, AKAP12, ANXA2, APOA1, APP, AQP9, ARHGAP25, B4GALT1, CAMK1D, CCDC88A, CCL23, CCL5, CCR1, CCR10, CLCN3, CNR1, CRKL, CSF1R, CSF3R, CUX1, CX3CR1, CXCL1, CXCL16, CXCL6, CXCL9, CXCR2, CXCR3, CYBB, DEFB103A/DEFB103B, DOCK2, ELN, EPHB1, F10, F2R, FCGR2A, FPR1, FPR2, FRS2, GAB1, GIT2, GRB2, HCK, HCLS1, HEBP1, HLA-G, HSPD1, ICAM1, IGF1, IGF1R, IGF2R, IL1B, ITGA4, JAML, LCP1, LGMN, LILRB3, LITAF, LRP2, LSP1, LYN, MAP3K1, MAPKAP1, mir-154, mir-24, MMP14, MPP1, MTOR, MUC1, MYLK, MYO1F, MYO5B, NCKAP1L, NEDD9, NINJ1, NR3C1, PDE4B, PDGFRA, PF4, PIP5K1A, PLCG2, PRKCD, PRKCG, PRKG1, PROK2, PTEN, PTGS2, PTPN6, RAC2, RALBP1, RHOA, RHOB, RICTOR, RPL13A, RTN4, S100A14, S100A9, SCN9A, SCRIB, SIRPA, SPHK2, STAT3, STK4, STX3, SWAP70, SYK, TAFA4, THBS2, TLR2, TLR4, TNFRSF1A, TNFSF14, TREM1, TREML2, TRIP10, WARS1, WIPF1, WNK1
Cellular Movement, Hematological System Development and Function, Immune Cell Trafficking	Homing of leukocytes	3.27E-05	Decreased	-4.568	86	ADAM10, ADAM17, AIF1, APOA1, APP, AQP9, ARHGAP25, B4GALT1, CAMK1D, CCDC88A, CCL23, CCL5, CCR1, CSF1R, CSF3R, CUX1, CX3CR1, CXCL1, CXCL16, CXCL6, CXCL9, CXCR2, CXCR3, CYBB, DEFB103A/DEFB103B, DOCK2, ELN, F2R, FCGR2A, FOXP3, FPR1, FPR2, FUT7, FYB1, GIT2, HCK, HEBP1, HLA-G, HSPD1, ICAM1, IL1B, ITGA4, JAK1, JAML, LCP1, LGMN, LILRB3, LITAF, LSP1, LTBR, LYN, MAPKAP1, MPP1, MYLK, MYO1F, NCKAP1L, NEDD9, NINJ1, NR3C1, PDE4B, PF4, PLCG2, PRKG1, PTEN, PTPN6, RAC2, RHOA, RHOB, RICTOR, RPL13A, S100A14, S100A9, SPHK2, STAT3, STK4, SWAP70, SYK, TAFA4, THBS2, TLR2, TLR4, TNFRSF1A, TNFSF14, TREM1, TREML2, WIPF1
Cell-To-Cell Signaling and Interaction	Adhesion of blood cells	3.27E-05	Decreased	-4.957	86	ADAM10, ADAM17, ADGRE2, ANXA5, APBB1IP, APOA1, APP, ATRN, B4GALT1, BTK, CCL5, CCR1, CD14, CD86, CLEC4M, CNR1, CSF3R, CTSZ, CX3CR1, CXCL1, CXCL9, CXCR2, CXCR3, CYBB, DOCK2, ENTPD1, EZR, F10, F11R, F2R, FCGR2A, FPR1, FPR2, FUT7, FYB1, GAB2, GALNT1, HCK, ICAM1, IL1B, ITGA4, ITGAX, JAK1, LAMA5, LCP1, LCP2, LGALS8, LILRB3, LRP8, LRPAP1, LSP1, LTBR, LYN, MAP3K2, MGAT5, MSN, NEDD9, NINJ1, NR3C1, PAK2, PECAM1, PF4, PILRA, PLCB3, PLCG2, PTGS2, PTPN6, RAC2, RAP1A, RHOA, RHOB, RICTOR, S100A9, SIRPA, ST6GALNAC2, STK4, SWAP70, SYK, THBS2, TLR2, TLR4, TLR5, TLR7, TNFRSF1A, TNIP1, TYROBP
Cellular Movement, Hematological System Development and	Cell movement of granulocytes	3.29E-05	Decreased	-3.864	89	ADAM10, ADAM15, ADAM17, ADM, ALOX5AP, ANXA2, APOA1, APP, AQP9, ARHGAP25, B4GALT1, BECN1, BTK, CAMK1D, CASP8, CCL23, CCL5, CCR1, CD14, CSF3R, CTSB, CTSC, CXCL1, CXCL6, CXCL9, CXCR2, CXCR3, CYBB, CYP2J2, DEFB103A/DEFB103B, DOCK2, EPO, F10, F2R, FCGR2A, FPR1, FPR2, FUT7, GIT2, HCK, HMOX1, ICAM1, IFNGR1, IL1B, IL1RN, ITGA4, JAML, LAMA5, LILRB3, LSP1, LYN, MGAT5, mir-133, MPP1, MYLK, MYO1F, NCKAP1L, NFE2L2, NFKBIZ, PDE4B, PECAM1, PF4, PLCB3, PLCG2, PLP1, PPM1D,

Function, Immune Cell Trafficking						PRKG1, PTEN, PTGS2, PTPN6, RAB27A, RAC2, RTN4, S100A14, S100A9, SGK1, SIRPA, STAT3, SWAP70, SYK, TLR2, TLR4, TLR7, TNFRSF1A, TNIP1, TREM1, TREML2, VTCN1, YBX1
Cellular Movement, Hematological System Development and Function, Immune Cell Trafficking	Cell rolling of leukocytes	3.75E-05	Decreased	-2.258	22	ADAM17, ARHGAP25, BTK, CD14, CHST1, CXCL1, CXCR2, FCGR2A, FPR2, FUT7, FYB1, GALNT1, HCK, ICAM1, IL1B, ITGA4, LCP2, LYN, MGAT5, RAC2, SWAP70, TLR4
Cellular Function and Maintenance	Internalization of tumor cell lines	3.79E-05	Decreased	-2.779	40	ACTR2, APC, APPL2, ARPC2, BTK, CD93, CLIP1, DEF6, DET1, DOCK2, EZR, FCGR2A, GRB2, HCK, HMOX1, ICAM1, JAK1, KAT6A, KCTD5, KRAS, MERTK, NCKAP1L, NCL, PIP5K1A, PLEK, PRKCD, PSMD4, PTPN6, RAB11A, RAB31, RALB, RHOA, RIT1, SLAMF7, TM2D2, TYROBP, UBE2L3, VIM, WASF2, ZNF217
Cancer, Gastrointestinal Disease, Organismal Injury and Abnormalities	Small intestine cancer	3.80E-05			25	APC, B2M, BCL7A, CTLA4, FCRLA, GSE1, HSP90AA1, HSP90AB1, HSP90B1, HVCN1, IRF8, KBTBD12, KRAS, MCL1, MSH6, NOTCH2, NTRK1, PRKCD, PRKCG, PTEN, RHOA, SSBP2, TET2, TRAF3, U2AF1/U2AF1L5
Cell Death and Survival	Cell death of fibroblast cell lines	3.83E-05	Decreased	-2.156	94	ADIPOR1, APP, ARNT, ASAH1, ATG3, ATG7, ATN1, ATP6AP2, BCL2L11, BECN1, BID, BNIP3L, BRCA1, CA4, CASP8, CFLAR, CLIC4, CRADD, CTSB, CWC15, DDIT3, DDX3X, DFFA, DMTF1, EIF3I, FOXL2, FOXO3, FTH1, GRB2, GSTA1, HMOX1, HNRNPA1, HOXA3, HSPA5, HSPD1, IFI16, IFNAR1, IGF1, INSM2, KLF6, KRAS, L3MBTL2, MAP3K1, MAPKAP1, MCL1, MDM2, MLKL, MTCH2, MTF1, MTOR, MUC1, MXD1, NFATC4, NFE2L2, NTRK1, NUDT13, OAZ1, OPA1, PAK2, PARK7, PDIA3, PLAGL2, PPM1D, PRKAR1A, PRKCD, PRKCG, PTEN, PURA, RALB, RALBP1, RHOA, RHOB, RIPK3, RPS6KA5, RTN4, SENP2, SERPINB3, SERPINB4, SF3B6, SKP2, SOD2, SPHK2, STK4, SYK, TACC1, TBK1, TMEM107, TNFRSF1A, TNFSF10, TNIP1, UNC5C, VDAC1, VIM, YWHAZ
Cellular Development, Cellular Growth and Proliferation, Hematological System Development and Function, Hematopoiesis, Lymphoid Tissue Structure and Development, Tissue Development	Leukopoiesis	3.85E-05	Decreased	-4.652	173	ADAM10, ADAM17, ADGRG3, ADM, APC, APP, ARID4B, ARNTL, ASXL1, B2M, BCL2L11, BRCA1, BTK, CASP8, CCL23, CCL5, CD14, CD86, CDA, CFLAR, CHD4, CLEC4M, CLEC6A, CREB1, CSF1R, CSF3R, CTLA4, CXCL1, CXCR2, CXCR3, CYBB, CYP26B1, DCLRE1C, DEF6, DMTF1, DNAJA2, DOCK2, DOCK8, DUSP5, DYRK1A, ELF1, ELF3, ENTPD1, EPHB1, EPO, EZR, FCAMR, FCGR2A, FOXO3, FOXP3, FUT7, FYB1, GAB2, GIMAP4, GIT2, GMPR2, GRB2, HCLS1, HDAC7, HDAC9, HLA-A, HLA-G, HOXA10, HOXA7, HSP90AA1, HSP90B1, HSPD1, ICAM1, IFI16, IFNAR1, IFNGR1, IFNGR2, IGF1, IGF1R, IGF2R, IL1B, IL1RN, IRF8, ITGA4, ITGB8, JAK1, KRAS, L3MBTL3, LAT2, LCP1, LCP2, let-7, LGALS8, LILRA2, LILRB3, LSP1, LTBR, LY9, LYN, MAPKAP1, MBP, MCL1, MDM2, MEF2C, MERTK, mir-24, MMP14, MPZL2, MS4A1, MSN, MTOR, NCKAP1L, NFE2L2, NFKBIZ, NMT1, NOTCH2, NTRK1, PF4, PHLPP1, PLCG2, PLP1, PPM1D, PRKAA1, PRKCD, PRL, PROK2, PSAP, PSMB8, PTEN, PTGS2, PTPN6, RAC2, RAD52, RAF1, RALB, RBPJ, RFFL, RGCC, RHOA, RICTOR, RIPK2, RIPK3, S100A9, SEMA4A, SFRP4, SH2B3, SIGLEC9, SKP2, SP3, SPINK5, STAT3, SWAP70, SYK, TCF4, TDP2, TET2, THEMIS2, THOC5, TLR2, TLR4, TLR5, TLR7, TMEM178A, TNFRSF1A, TNFSF10, TNFSF4, TRAF3, TREM1, TYROBP, USP15, USP4, VDR, VTCN1, WIPF1, XRCC5, ZBTB46, ZEB2, ZRSR2
Connective Tissue Disorders, Immunological Disease, Inflammatory Disease, Inflammatory Response, Organismal Injury and Abnormalities, Skeletal and Muscular Disorders	Rheumatoid arthritis	3.89E-05		0.851	159	ACO1, ACSL1, ADAM10, ADAM15, ADAM17, ADGRA1, ADIPOR1, ADM, AIF1, APLP2, APOA1, AQP9, ARF1, ARHGDIB, ATAT1, B2M, BGN, C9orf78, CARD8, CASC3, CCL23, CCL5, CCR1, CD86, CDA, CELF2, CLEC1B, CLEC4D, CLIC2, CSF3R, CTLA4, CTSB, CTSC, CX3CR1, CXCL1, CXCL16, CXCL6, CXCL9, CXCR2, CYP4F3, DEF6, DNAJA4, DYNLL1, ECHDC1, EEF1E1, EIF1B, EPO, F10, F11R, FCGR2A, FGL2, FKBP5, FOXO3, FOXP3, FPR2, FTH1, GALNT1, GLIPR2, GLUL, H3-3A/H3-3B, HAMP, HCK, HCLS1, HDAC7, HLA-A, HLA-C, HLA-G, HMOX1, HNMT, HNRNPA1, HSP90B1, HSPA1A/HSPA1B, HSPD1, ICAM1, IFNAR1, IGF1, IGFBP4, IL1B, IL1R2, IL1RN, JAK1, JMJD1C, KCTD20, KRAS, LCP1, LINC00922, LYZ, MACIR, MAP3K2, MAP4K4, MAPRE1, MCL1, MDM2, MEFV, MMP14, MRFAP1, MS4A1, MS4A7, MTOR, NAMPT, NOM1, NONO, NR3C1, NTRK1, NUMB, P2RY13, PDIA3, PECAM1, PHTF1, PLAC4, PSMB8, PTGS2, PTMA, PTPRE, RALB, RAMP2, RFX3, RGCC, RNF149, RNF169, RPL18A, RTF2, S100A9, SEC14L3, SEL1L, SF3B6, SLC22A4, SORL1, SPOCK1, STAT3, STEAP4, STK19, SWT1, SYK, TALDO1, TCF4, TFRC, TJP1, TLR2, TLR4, TLR7, TNFRSF10C, TNFRSF10D, TNFRSF1A, TNFSF10, TNFSF4, TNNC1, TRIO, TUT7, UQCRC2, USP15, VDR, VIM, VTCN1, WNK1, ZNF143, ZNF281, ZNF326, ZNF331
Cancer, Organismal Injury and Abnormalities, Tumor Morphology	Invasion of tumor	3.90E-05	Decreased	-2.853	56	ANXA2, APC, CD14, COL7A1, CTNND1, CTNND2, CTSB, CTSZ, CXCL1, CXCL6, EZR, F2R, FOXO3, G3BP2, GAB1, GAB2, HDLBP, HMOX1, HNRNPA1, HSPA5, ICAM1, IGF1, KRAS, let-7, LHCGR, LIMK2, MDM2, mir-103, mir-133, MMP14, NEDD9, NFE2L2, NOTCH2, NUAK2, PARK7, PDCD4, PDGFRA, PSMD10, PTEN, PTGS2, RALBP1, RHOA, RHOB, S100A9, SCRIB, SETDB1, SSX2IP, STAT3, SYK, TDGF1, TLR4, TRAF3, VCAN, VIM, WASF3, ZFYVE21

Cell-To-Cell Signaling and Interaction	Binding of lymphatic system cells	3.96E-05	Decreased	-3.523	45	ADGRG3, ANXA2, APBB1IP, APOA1, BGN, BTK, CCL5, CCR1, CD86, CTLA4, CXCL9, CXCR3, DOCK2, DOCK8, EZR, F2R, FUT7, FYB1, ICAM1, IFNGR1, IL1B, ITGA4, JAK1, LCP2, LTBR, LYN, MAP3K2, MSN, MTOR, NEDD9, NR3C1, PECAM1, PRL, PTPN6, RAC2, RAP1A, RHOA, RICTOR, STK4, SWAP70, SYK, TFRC, THBS2, TLR4, TNFSF14
Cell-To-Cell Signaling and Interaction, Hematological System Development and Function	Binding of leukocytes	3.98E-05	Decreased	-5.297	87	ADAM10, ADAM17, ADGRE2, APBB1IP, APOA1, APP, ATRN, B4GALT1, BTK, CCL5, CCR1, CD14, CD86, CLEC4M, CNR1, CSF3R, CTLA4, CTSZ, CX3CR1, CXCL1, CXCL9, CXCR2, CXCR3, CYBB, DOCK2, DOCK8, ENTPD1, EZR, F10, F11R, F2R, FCGR2A, FPR1, FPR2, FUT7, FYB1, GAB2, GALNT1, HCK, ICAM1, IFNGR1, IL1B, ITGA4, ITGAX, JAK1, LCP1, LCP2, LGALS8, LILRB3, LSP1, LTBR, LYN, MAP3K2, MGAT5, MSN, NEDD9, NINJ1, NOTCH2, NR3C1, PAK2, PECAM1, PF4, PILRA, PLCB3, PRL, PTGS2, PTPN6, RAC2, RAP1A, RHOA, RHOB, RICTOR, S100A9, SIRPA, STK4, SWAP70, SYK, TFRC, THBS2, TLR2, TLR4, TLR5, TLR7, TNFRSF1A, TNFSF14, TNIP1, TYROBP
Infectious Diseases	Production of virus	4.08E-05		-1.859	31	AGO2, APOB, ASMTL, BECN1, CD14, CNP, DDX17, DDX5, EDEM3, EIF3G, FAS-AS1, HCK, HNRNPA1, HSP90AB1, IFNAR1, LCP2, MAP1LC3A, mir-122, PSMC2, PTPN6, SART3, SDCBP, SNAPIN, SNRPF, STAU1, TKFC, TLR2, TNFRSF1A, TSG101, YBX1, ZNF175
Cellular Function and Maintenance	Pinocytosis	4.35E-05		-1.85	17	ANKFY1, APC, CARMIL1, DAB2, DOCK2, EZR, FRS2, KRAS, MAPKAPK3, NCL, NTRK1, RAB5A, RHOA, RHOB, TLR4, TNFRSF1A, WNK1
Free Radical Scavenging	Production of reactive oxygen species	4.60E-05	Decreased	-3.16	87	ACOX1, ADGRE2, ALDH3A2, ALS2, ANXA2, AOPEP, APP, ARHGDIB, ARNT, ATG7, BID, BRCA1, CASP8, CCL5, CD14, CLEC7A, CTLA4, CXCL9, CYBB, DDIT3, DOCK2, EPO, F2R, FCGR2A, FOXO3, FPR1, FPR2, FTH1, FTL, HBA1/HBA2, HBB, HCK, HMOX1, HSP90AB1, HVCN1, ICAM1, IGF1, IL1B, ITGAX, JAK1, LCP2, LYN, MLKL, MS4A1, MTOR, MUC1, MYLK, NCF2, NDUFS1, NFE2L2, NTRK1, PARK7, PCK1, PECAM1, PLAGL2, PLCG2, PRKAA1, PRKCD, PSMB8, PTEN, PTGS2, PTPN6, RAC2, RBPJ, RHOA, RIPK3, RTN4, SIGLEC9, SLC8A1, SLU7, SNAP23, SOD2, SPAG9, SYK, TAZ, TIGAR, TLR2, TLR4, TLR5, TMBIM6, TNFRSF1A, TRAF3, TREML2, TXNRD1, TYROBP, VDAC1, YWHAZ
Cancer, Gastrointestinal Disease, Organismal Injury and Abnormalities	Carcinoma of ampulla of Vater	4.63E-05			11	APC, FCRLA, GSE1, HSP90AA1, HSP90AB1, HSP90B1, KBTBD12, KRAS, PRKCD, PRKCG, U2AF1/U2AF1L5
Cellular Movement	Cellular infiltration by blood cells	4.95E-05	Decreased	-2.456	99	ADAM17, ADM, ALOX5AP, ANXA2, APC, APOA1, APP, ARHGAP25, ATG7, B4GALT1, BECN1, BGN, BID, CASP8, CCL5, CCR1, CD14, CD86, CD93, CNP, CNR1, CSF1R, CTLA4, CTSB, CTSC, CUX1, CX3CR1, CXCL16, CXCL9, CXCR2, CXCR3, CYBB, CYP2J2, DEF6, DOCK2, EFS, EPO, EZR, F2R, FGL2, FOXP3, FPR1, FPR2, FUT7, GAL3ST1, HAMP, HCK, HLA-A, HMOX1, HSPA5, HSPD1, ICAM1, IFNGR1, IL1B, IL1RN, ITGA4, KCNE3, KRAS, LILRB3, LTBR, LYN, MMP14, MYLK, NFE2L2, NFKBIZ, NINJ1, OPA1, PF4, PLCB3, PLP1, PPM1D, PRKAA1, PRKCD, PRKG1, PSMB8, PTEN, PTGS2, PTMA, PTPN6, RAB27A, RAC2, RGCC, RIPK2, RPL13A, S100A9, SGK1, STAT3, TET2, TLR2, TLR4, TLR7, TNFRSF1A, TNFSF10, TNFSF4, TNIP1, TREM1, VDR, VTCN1, YBX1
Connective Tissue Disorders, Inflammatory Disease, Organismal Injury and Abnormalities, Skeletal and Muscular Disorders	Rheumatic Disease	5.24E-05		0.407	259	ABCC2, ACO1, ACSL1, ADAM10, ADAM15, ADAM17, ADGRA1, ADIPOR1, ADM, AIF1, ALOX5AP, APLP2, APOA1, APOBEC3A, AQP9, ARF1, ARHGDIB, ARPC5, ATAT1, B2M, BCL2L11, BGN, BID, C9orf78, CARD8, CASC3, CASP8, CCL23, CCL5, CCR1, CD86, CDA, CELF2, CFLAR, CLEC1B, CLEC4D, CLIC2, CNR1, COL1A2, COL9A2, CPT1A, CRB1, CREB1, CRY2, CSF1R, CSF3R, CTLA4, CTSB, CTSC, CX3CR1, CXCL1, CXCL16, CXCL6, CXCL9, CXCR2, CXCR3, CYP4F3, DDIT3, DEF6, DGAT2, DKK3, DNAJA4, DUSP5, DYNLL1, ECHDC1, EEF1E1, EIF1B, ELF3, EPO, ERCC5, F10, F11R, F13A1, FCGR2A, FCGR2C, FGL2, FKBP5, FOXO3, FOXP3, FPR1, FPR2, FTH1, FTL, GAB1, GALNT1, GLIPR2, GLUL, H3-3A/H3-3B, HAMP, HBA1/HBA2, HBB, HCK, HCLS1, HDAC7, HLA-A, HLA-C, HLA-E, HLA-G, HMOX1, HNMT, HNRNPA1, HOXA7, HSP90B1, HSPA1A/HSPA1B, HSPA5, HSPD1, ICAM1, IFI16, IFNAR1, IFNGR1, IGF1, IGF1R, IGFBP4, IL1B, IL1R2, IL1RN, IRF8, ITGAX, JAK1, JMJD1C, KCTD20, KRAS, LCP1, let-7, LINC-PINT, LINC00922, LTBR, LY9, LYN, LYZ, MACIR, MAP3K2, MAP4K4, MAPRE1, MCL1, MDM2, MEFV, MERTK, mir-154, mir-24, mir-299, MMP14, MRFAP1, MS4A1, MS4A7, MTOR, NAMPT, NCF2, NFE2L2, NOM1, NONO, NR3C1, NTRK1, NUMB, NUP62, OPA1, OXT, P2RY13, PDE4B, PDGFRA, PDIA3, PECAM1, PF4, PHTF1, PILRA, PLA2G4C, PLAC4, PLCG2, PRL, PROK2, PSMB8, PSME3, PTEN, PTGS2, PTMA, PTPN6, PTPRE, PURA, RAB27A, RAB31, RAB5A, RABGAP1L, RALB, RAMP2, RBPJ, RFPL2, RFX3, RGCC, RHOA, RIPK2, RIPK3, RNF149, RNF169, RPL18A, RTF2, S100A9, SCN9A, SEC14L3, SEL1L, SF3B6, SGK1, SKP2, SLAMF7, SLC11A1, SLC22A4, SLU7, SOD2, SORL1, SOS2, SP1, SPHK2, SPOCK1, SRSF1, SRSF3, STAT3, STEAP4, STK19, STXBP6, SWT1, SYK, TALDO1, TBK1, TCF4, TFRC, TJP1, TLR2, TLR4, TLR5, TLR7, TMEM178A, TMEM39A, TNFRSF10C, TNFRSF10D, TNFRSF1A, TNFSF10, TNFSF4, TNIP1, TNNC1, TNPO3, TRIO, TUBA1A, TUBA1C, TUBB2A, TUT7, TYROBP, UBE2L3, UQCRC2, USP15, VDR, VIM, VTCN1, WARS1, WIPF1, WNK1, ZNF143, ZNF148, ZNF281, ZNF326, ZNF331
Cellular Movement, Hematological System Development and Function, Immune Cell Trafficking	Cellular infiltration by leukocytes	5.45E-05	Decreased	-2.583	98	ADAM17, ADM, ALOX5AP, ANXA2, APC, APOA1, APP, ARHGAP25, ATG7, B4GALT1, BECN1, BGN, BID, CASP8, CCL5, CCR1, CD14, CD86, CD93, CNP, CNR1, CSF1R, CTLA4, CTSB, CTSC, CUX1, CX3CR1, CXCL16, CXCL9, CXCR2, CXCR3, CYBB, CYP2J2, DEF6, DOCK2, EFS, EPO, EZR, F2R, FGL2, FOXP3, FPR1, FPR2, FUT7, GAL3ST1, HAMP, HCK, HLA-A, HMOX1, HSPA5, HSPD1, ICAM1, IFNGR1, IL1B, IL1RN, ITGA4, KCNE3, KRAS, LILRB3, LTBR, LYN, MMP14, MYLK, NFE2L2, NFKBIZ, NINJ1, OPA1, PF4, PLCB3, PLP1, PPM1D, PRKAA1, PRKCD, PRKG1, PSMB8, PTEN, PTGS2, PTMA, PTPN6, RAB27A, RAC2, RGCC, RIPK2, RPL13A, S100A9, SGK1, STAT3, TLR2, TLR4, TLR7, TNFRSF1A, TNFSF10, TNFSF4, TNIP1, TREM1, VDR, VTCN1, YBX1

Cell Death and Survival	Cell death of pheochromocytoma cell lines	5.63E-05		-0.712	30	APP, ATG7, ATN1, ATXN3, BCL2L11, BNIP3L, BTG2, DYNLL1, FFAR4, GAB1, GAPDH, HERPUD1, IGF1, IL1B, IL1RN, MAP3K1, NTRK1, PRKCD, PSAP, PTGS2, PTPN6, RIT1, SIAH1, SIRPA, SOD2, SP1, SPHK2, STAT3, TNFRSF1A, VCAN
Cell Death and Survival	Cell death of myeloid cells	5.77E-05		-0.619	57	ADAM17, APP, ASAH1, ATG7, BID, BNIP3L, BTK, CASP8, CCL5, CD14, CFLAR, CTSB, CXCL1, CYBB, DDIT3, DFFA, ENTPD1, EPO, FOXO3, HMOX1, IFNAR1, IGF1, IL1B, IL1RN, IRF8, LYN, LYZ, MCL1, MDM2, MEFV, mir-154, MLKL, MTOR, NAMPT, NFE2L2, NR3C1, PELI2, PF4, PRKCD, PTEN, PTPN6, RAF1, RALBP1, RIPK3, SH3BP2, SIGLEC9, SIRPA, SOD2, STAT3, STK4, SYK, TLR2, TLR4, TMOD3, TNFRSF1A, TNFSF10, TREM1
Hematological System Development and Function, Humoral Immune Response, Lymphoid Tissue Structure and Development, Tissue Morphology	Quantity of follicular B lymphocytes	5.80E-05	Decreased	-3.434	34	ADAM10, ADGRG3, APBB1IP, ARHGDIB, ARNTL, BCL2L11, BTK, CASP8, CD84, DKK3, DOCK2, DOCK8, GALNT1, HVCN1, IFNAR1, IFNGR1, IRF8, KIDINS220, KRAS, LYN, MTOR, NEDD9, NOTCH2, PLCG2, PRKCD, PTEN, SH3BP2, STAT3, STK4, TET2, TLR2, TLR4, TYROBP, WIPF1
Cell-To-Cell Signaling and Interaction, Inflammatory Response	Response of macrophages	5.99E-05	Decreased	-3.348	44	APOA1, APOA2, APP, ATG7, BECN1, BTK, CD14, CD93, CLEC4M, CLEC6A, CLEC7A, CLIC4, CSF1R, DEF6, DOCK2, F2R, FCGR2A, GAB2, HCK, HMOX1, IL1B, IRF8, let-7, LYN, MERTK, MEX3B, mir-24, NCKAP1L, NR3C1, PLCB3, PRKAA1, PTEN, RAB11A, RGCC, S100A9, SH3BP2, SIRPA, SIRPB1, SLAMF7, STAT3, TLR2, TLR4, TREM1, TYROBP
Cell Death and Survival	Cell death of connective tissue cells	6.19E-05		-0.896	128	ADIPOR1, ADM, APP, ARNT, ASAH1, ATF5, ATG3, ATG7, ATN1, ATP6AP2, BABAM2, BCL2L11, BECN1, BGN, BID, BNIP3L, BRCA1, CA4, CASP8, CCDC47, CFLAR, CLIC4, CNR1, CRADD, CREB1, CRKL, CTSB, CWC15, DDIT3, DDX3X, DFFA, DMTF1, EIF3I, ELOA, FOXL2, FOXO3, FTH1, GAB1, GRB2, GSTA1, HMOX1, HNRNPA1, HOXA3, HSPA5, HSPD1, IFI16, IFNAR1, IGF1, IGF1R, IGF2R, IL1B, INSM2, KLF6, KRAS, L3MBTL2, LGALS8, LRPAP1, M6PR, MAP3K1, MAP4, MAPKAP1, MCL1, MDM2, mir-103, MLKL, MMP14, MTCH2, MTF1, MTOR, MUC1, MXD1, NFATC4, NFE2L2, NTRK1, NUDT13, OAZ1, OPA1, PAK2, PARK7, PDIA3, PITPNA, PLAGL2, PPM1D, PRKAA1, PRKAR1A, PRKCD, PRKCG, PRL, PTEN, PURA, RAD51D, RAF1, RALB, RALBP1, RHOA, RHOB, RIPK3, RPS6KA5, RTN4, SENP2, SERPINB3, SERPINB4, SETDB1, SF3B6, SFRP4, SGK1, SKP2, SOD2, SPHK2, STAT3, STK4, SYK, TACC1, TBK1, TCF4, TMEM107, TNFRSF10D, TNFRSF1A, TNFSF10, TNFSF14, TNIP1, TRAF3, TSG101, UNC5C, VDAC1, VDR, VIM, YWHAZ
RNA Post-Transcriptional Modification	Processing of RNA	6.26E-05		-0.141	86	AGO2, AKAP8L, APP, ATXN3, BMS1, BUD13, CASC3, CDC5L, CELF1, CELF2, CLK2, CLK3, CPEB1, CPSF4, CPSF7, CSTF1, CWC15, DDX17, DDX23, DDX27, DDX39A, DDX5, DHX38, DHX8, DYRK1A, FUS, GTF2H3, HBB, HNRNPA1, HNRNPA2B1, HNRNPH1, HNRNPH2, IK, INTS4, INTS6, LAS1L, LSM3, LSM6, MAGOHB, MPHOSPH10, NCBP1, NONO, NSRP1, NUDT21, PABPC1, PIN4, PNN, POP5, PQBP1, PRPF38A, PRPF4, PRPF6, RBM22, RBM25, RBM4, RBM5, RBMS1, RPL5, RPS15, SART3, SF3A3, SF3B1, SF3B6, SLU7, SNRPB, SNRPD3, SNRPF, SRPK1, SRPK2, SRSF1, SRSF3, SRSF4, SRSF5, THRAP3, TRNT1, U2AF1/U2AF1L5, UTP11, UTP14A, UTP3, UTP4, WBP11, WDR33, YBX1, ZMAT2, ZNF326, ZRSR2
Cellular Movement	Cell tethering or rolling	6.56E-05	Decreased	-2.745	25	ADAM17, ARHGAP25, ARNTL, BTK, CD14, CHST1, CXCL1, CXCR2, FCGR2A, FPR2, FUT7, FYB1, GALNT1, HCK, ICAM1, IL1B, ITGA4, LCP2, LYN, MGAT5, RAC2, ST3GAL6, ST6GALNAC2, SWAP70, TLR4
Cell Death and Survival	Apoptosis of myeloma cell lines	6.56E-05		0.659	25	ARNT, B2M, BCL2L11, BTK, CASP8, CXCR3, DDIT3, FOXO3, IGF1, IGF1R, IRF8, KRAS, MCL1, MDM2, mir-154, MTOR, NR3C1, PRKCD, PTEN, SP1, STAT3, STK4, TNFSF10, VDAC1, YBX1
Cell-To-Cell Signaling and Interaction, Inflammatory Response	Immune response of macrophages	6.56E-05	Decreased	-3.089	41	APOA1, APOA2, APP, ATG7, BECN1, BTK, CD14, CD93, CLEC4M, CLEC6A, CLIC4, CSF1R, DEF6, DOCK2, F2R, FCGR2A, GAB2, HCK, HMOX1, IL1B, IRF8, let-7, LYN, MERTK, MEX3B, mir-24, NCKAP1L, NR3C1, PRKAA1, PTEN, RAB11A, RGCC, S100A9, SH3BP2, SIRPA, SIRPB1, SLAMF7, TLR2, TLR4, TREM1, TYROBP
Cell-To-Cell Signaling and Interaction, Hematological System Development and Function, Immune Cell Trafficking	Adhesion of immune cells	6.73E-05	Decreased	-5.381	80	ADAM10, ADAM17, ADGRE2, APBB1IP, APOA1, APP, ATRN, B4GALT1, BTK, CCL5, CCR1, CD14, CD86, CLEC4M, CNR1, CSF3R, CTSZ, CX3CR1, CXCL1, CXCL9, CXCR2, CXCR3, CYBB, DOCK2, ENTPD1, EZR, F10, F11R, F2R, FCGR2A, FPR1, FPR2, FUT7, FYB1, GAB2, GALNT1, HCK, ICAM1, IL1B, ITGA4, ITGAX, JAK1, LCP1, LCP2, LGALS8, LILRB3, LSP1, LTBR, LYN, MAP3K2, MGAT5, MSN, NEDD9, NINJ1, NR3C1, PAK2, PECAM1, PF4, PILRA, PLCB3, PTGS2, PTPN6, RAC2, RAP1A, RHOA, RHOB, RICTOR, S100A9, SIRPA, STK4, SWAP70, SYK, THBS2, TLR2, TLR4, TLR5, TLR7, TNFRSF1A, TNIP1, TYROBP

Cell Morphology, Cellular Assembly and Organization, Cellular Function and Maintenance	Formation of lamellipodia	6.89E-05	Decreased	-4.743	42	ACTR2, ARPC2, BECN1, BTK, CAP1, CAPZB, CARMIL1, CCDC88A, CRKL, CTLA4, CTNND2, CXCR3, DKK3, EZR, FGD4, FNBP1L, GAB1, HSP90AA1, IGF1, ITGA4, ITGB8, LASP1, LCP1, LCP2, MAP3K1, MTOR, NCF2, PIP5K1A, PLCG2, RAB5A, RAC2, RHOA, RHOB, SIRPB1, SPATA13, STAT3, SWAP70, SYK, TRIP10, VIM, WASF2, WASF3
Immunological Disease	Systemic autoimmune syndrome	6.90E-05		-0.056	266	ACO1, ACSL1, ADAD1, ADAM10, ADAM15, ADAM17, ADGRA1, ADIPOR1, ADM, AIF1, ALOX5AP, ANXA3, APBB1IP, APLP2, APOA1, APOBEC3A, AQP9, ARF1, ARHGDIB, ARIH2, ATAT1, ATP6V1B2, B2M, BCL2L11, BGN, BTN2A1, C9orf78, CAPSL, CARD8, CASC3, CASP8, CCL23, CCL5, CCR1, CD14, CD84, CD86, CDA, CELF2, CFLAR, CLEC1B, CLEC4D, CLEC6A, CLEC9A, CLIC2, COL1A2, COL7A1, CPT1A, CRB1, CREB1, CSF3R, CTLA4, CTSB, CTSC, CX3CR1, CXCL1, CXCL16, CXCL6, CXCL9, CXCR2, CXCR3, CYBB, CYP4F3, DDC, DEF6, DGAT2, DKK3, DNAJA4, DUSP5, DYNLL1, ECHDC1, EEF1E1, EIF1B, EPO, ERCC5, F10, F11R, FCGR2A, FCGR2C, FGL2, FKBP5, FOXO3, FOXP3, FPR2, FTH1, GALNT1, GIMAP4, GLIPR2, GLUL, H2AC18/H2AC19, H3-3A/H3-3B, HAAO, HAMP, HBA1/HBA2, HCK, HCLS1, HDAC7, HLA-A, HLA-C, HLA-G, HMOX1, HNMT, HNRNPA1, HOXA7, HSP90B1, HSPA1A/HSPA1B, HSPD1, ICAM1, IER2, IFI16, IFNAR1, IFNGR1, IFNGR2, IGF1, IGF1R, IGFBP4, IL1B, IL1R2, IL1RN, IRF8, ITGA4, ITGAX, JAK1, JAML, JMJD1C, KCTD20, KRAS, LCP1, LILRB3, LINC-PINT, LINC00922, LRP8, LY86, LY9, LYN, LYZ, MACIR, MAP3K2, MAP4K4, MAPRE1, MCL1, MDM2, MEFV, MERTK, MICB, mir-122, mir-154, mir-24, mir-299, MMP14, MPEG1, MRFAP1, MS4A1, MS4A7, MTOR, MUC1, MYO1F, NAMPT, NCF2, NOM1, NONO, NR3C1, NTRK1, NUMB, NUP62, OPA1, P2RY13, PABPC1, PDCD4, PDE4B, PDIA3, PECAM1, PHTF1, PILRB, PLA2G4C, PLAC4, PLEK, PRKCD, PROK2, PSMB8, PTGS2, PTMA, PTPN6, PTPRE, RAB27A, RAB31, RAB5A, RABGAP1L, RALB, RAMP2, RFPL2, RFX3, RGCC, RGS2, RIPK3, RNF149, RNF169, RPL18, RPL18A, RTF2, RTL6, S100A9, SEC14L3, SEL1L, SF3B6, SGK1, SH2B3, SIRPB1, SKP2, SLAMF7, SLC22A4, SLU7, SNX13, SOD2, SORL1, SOS2, SP1, SPOCK1, SRSF1, STAT3, STEAP4, STK19, STXBP6, SULT1A2, SWT1, SYK, TALDO1, TBK1, TCF4, TFRC, TJP1, TLR2, TLR4, TLR5, TLR7, TMEM140, TMEM39A, TNFRSF10C, TNFRSF10D, TNFRSF1A, TNFSF10, TNFSF4, TNIP1, TNNC1, TNP2, TNPO3, TRIO, TUT7, TYROBP, UBE2L3, UQCRC2, USP15, VDR, VIM, VTCN1, WARS1, WNK1, XAF1, ZNF143, ZNF148, ZNF165, ZNF281, ZNF326, ZNF331, ZNF468, ZSCAN12
Cellular Movement, Hematological System Development and Function, Immune Cell Trafficking, Inflammatory Response	Chemotaxis of neutrophils	7.01E-05	Decreased	-3.02	40	APOA1, APP, AQP9, ARHGAP25, CAMK1D, CCL23, CCL5, CSF3R, CXCL1, CXCL6, CXCR2, DEFB103A/DEFB103B, DOCK2, FCGR2A, FPR1, FPR2, GIT2, HCK, ICAM1, IL1B, ITGA4, JAML, LILRB3, LSP1, LYN, MPP1, MYO1F, NCKAP1L, PDE4B, PF4, PRKG1, PTEN, PTPN6, RAC2, S100A9, SYK, TLR4, TNFRSF1A, TREM1, TREML2
Cell-To-Cell Signaling and Interaction, Cellular Function and Maintenance, Hematological System Development and Function, Inflammatory Response	Phagocytosis of leukocyte cell lines	7.03E-05	Decreased	-2.776	11	APOA1, APOA2, APP, BECN1, CD14, DDX3X, FCGR2A, PTPN6, SNAP23, TLR2, TLR4
Hematological Disease	Polycythemia	7.24E-05		1.759	51	APC, APOA1, APP, ARNTL, ASXL1, BCL2L11, BNIP3L, BPGM, CFLAR, CSF1R, CSF3R, CUX1, CXCL1, CXCR2, DEF6, EPO, FUT7, HAMP, HBA1/HBA2, HBB, IFNAR1, IL1B, IL1RN, ITGA4, JAK1, KRAS, let-7, LILRB3, MGAT5, mir-26, NFE2L2, NR3C1, PDE4B, PDE8A, PDGFRA, PF4, PTGS2, PTPN6, RAC2, RHOA, S100A9, SF3B1, SH2B3, STAT3, SYK, TET2, THBS2, TLR2, TNFSF10, TRNT1, U2AF1/U2AF1L5
Cardiovascular System Development and Function, Organismal Development	Angiogenesis	7.51E-05	Decreased	-5.991	213	ACTG1, ADAM15, ADAM17, ADM, ADM2, AGO2, AIF1, ALOX5AP, ANGPTL4, ANTXR2, ANXA2, ANXA3, APC, APOA1, APOB, APP, ARID4B, ARNT, ARNTL, ATG7, B4GALT1, BCAS3, BECN1, BRCA1, C1GALT1C1, CAMK2A, CARD6, CASP8, CAVIN2, CCDC88A, CCL5, CHM, CLEC1B, CLIC4, CNMD, CNR1, COL1A2, CREB1, CRKL, CSF1R, CTSB, CX3CR1, CXCL1, CXCL6, CXCL9, CXCR2, CXCR3, CYBB, CYP4F2, CYP51A1, DAB2, DCTN5, DDIT3, DHCR7, DUSP3, ECE1, EHD3, ELK3, ELN, EMC10, EPHB1, EPO, ERO1A, ETV6, F11R, F2R, FBLN2, FFAR4, FOXO3, FPR2, FRS2, GAB1, GAB2, GATA5, GATAD2A, GLUL, HCK, HDAC7, HDAC9, HLA-G, HMOX1, HOXA3, HOXA7, HSP90AA1, HSPA5, HSPD1, HTATIP2, ICAM1, IDH3A, IFI16, IFNAR1, IFT88, IGF1, IGF1R, IGF2R, IGFBP4, IL1B, IL1RN, ITGA4, ITGAX, ITGB8, KAT6A, KCNJ2, KLF6, KLF7, KLHL20, LAMA5, LEFTY1, let-7, LGALS8, LRP2, LRP8, LRPAP1, LRRFIP1, LTBR, LYVE1, MDM2, MEF2C, MERTK, MGAT5, mir-103, mir-122, mir-133, mir-137, mir-154, mir-24, mir-26, MMP14, MTDH, MTOR, MYOF, NCF2, NCL, NCOA1, NFATC4, NFE2L2, NOTCH2, NR3C1, NTRK1, OTULIN, OXT, PAQR3, PDGFRA, PEAK1, PECAM1, PF4, PITX2, PLXNA4, PRKAA1,

						PRKCD, PRKCG, PRKG1, PRL, PRLH, PROK2, PSAP, PTEN, PTGS2, PTPN6, RAB9A, RAC2, RAF1, RAMP2, RAP1A, RAPGEF2, RBPJ, RGCC, RGS2, RHOA, RHOB, RICTOR, RIPK3, RTN4, S100A9, SAT1, SEMA4A, SIRPA, SKP2, SLC8A1, SOS2, SP1, SP100, SPHK2, SPINK5, SRGN, SRPK1, SRPK2, STAT3, STK4, STX7, SUFU, SYK, TAZ, TCF4, TDGF1, THAP1, THBS2, THRAP3, TJP1, TLR2, TLR4, TNFRSF1A, TNFSF10, TUBA1C, VIM, WARS1, WASF2, WNK1, WWTR1, YWHAZ, ZBTB46, ZFPM2, ZNF24
Cell Death and Survival	Cell death of blood cells	7.95E-05		-1.824	145	ADAM17, ADGRE2, ADGRG3, ANTXR2, AOPEP, APC, APOB, APP, ARNT, ASAH1, ATG3, ATG7, AURKB, BCL2L11, BECN1, BID, BNIP3L, BRCA1, BTK, CASP8, CCL5, CD14, CD86, CFLAR, CNR1, CREB1, CRKL, CSF1R, CTLA4, CTSB, CX3CR1, CXCL1, CYBB, DDIT3, DEF6, DFFA, DKK3, DOCK8, DUSP5, ELF1, ENTPD1, EPO, EZR, F2R, FGL2, FOXO3, FTH1, FUS, GAB2, GAPDH, GIMAP4, HCK, HCLS1, HLA-G, HMOX1, HOXA3, HSP90AB1, HSPA5, ICAM1, IFNAR1, IFNGR1, IGF1, IL1B, IL1RN, IP6K2, IRF8, ITGA4, JAK1, KIF1C, KRAS, LAT2, LGALS8, LY9, LYN, LYZ, MAP3K2, MCL1, MDM2, MEF2C, MEFV, MERTK, MGAT5, mir-154, mir-24, MLKL, MS4A1, MTOR, MTTP, MVP, MXD1, NAMPT, NCF2, NFE2L2, NR3C1, NUMB, PDIA3, PECAM1, PELI2, PF4, PLCG2, PPM1D, PRKAA1, PRKCD, PRL, PTEN, PTPN6, RAC2, RAF1, RALBP1, RBM5, RBPJ, RHOA, RICTOR, RIPK3, SF3B1, SH3BP2, SIGLEC9, SIRPA, SLC6A6, SOD2, SP1, STAT3, STK4, SWAP70, SYK, TBC1D15, THAP1, THBS2, TLR2, TLR4, TLR7, TMOD3, TNFRSF1A, TNFSF10, TNFSF14, TRAF3, TREM1, TYROBP, USP17L2 (includes others), VDR, WIPF1, XRCC5, YWHAZ, ZEB2, ZMPSTE24
Cell Death and Survival	Cell death of immune cells	8.10E-05	Decreased	-2.325	137	ADAM17, ADGRE2, ADGRG3, ANTXR2, AOPEP, APOB, APP, ATG3, AURKB, BCL2L11, BECN1, BID, BRCA1, BTK, CASP8, CCL5, CD14, CD86, CFLAR, CNR1, CREB1, CRKL, CSF1R, CTLA4, CTSB, CX3CR1, CXCL1, CYBB, DDIT3, DEF6, DFFA, DKK3, DOCK8, DUSP5, ELF1, ENTPD1, EPO, EZR, F2R, FGL2, FOXO3, FTH1, FUS, GAB2, GAPDH, GIMAP4, HCK, HCLS1, HLA-G, HMOX1, HOXA3, HSP90AB1, HSPA5, ICAM1, IFNAR1, IFNGR1, IGF1, IL1B, IL1RN, IP6K2, IRF8, ITGA4, JAK1, KIF1C, KRAS, LAT2, LGALS8, LY9, LYN, LYZ, MAP3K2, MCL1, MDM2, MEF2C, MEFV, MERTK, MGAT5, mir-154, mir-24, MLKL, MS4A1, MTOR, MTTP, MVP, NAMPT, NCF2, NFE2L2, NR3C1, NUMB, PDIA3, PECAM1, PELI2, PF4, PLCG2, PPM1D, PRKAA1, PRKCD, PRL, PTEN, PTPN6, RAC2, RAF1, RALBP1, RBM5, RBPJ, RHOA, RICTOR, RIPK3, SH3BP2, SIGLEC9, SIRPA, SLC6A6, SOD2, SP1, STAT3, STK4, SWAP70, SYK, TBC1D15, THAP1, THBS2, TLR2, TLR4, TLR7, TNFRSF1A, TNFSF10, TNFSF14, TRAF3, TREM1, TYROBP, USP17L2 (includes others), VDR, WIPF1, XRCC5, YWHAZ, ZEB2, ZMPSTE24
Connective Tissue Disorders, Hematological Disease, Organismal Injury and Abnormalities	Thrombocytopeni a	8.25E-05	Increased	3.548	54	APC, APP, ARID4B, ARNT, ASXL1, BCL2L11, C1GALT1C1, CDA, CLEC1B, CSF3R, ENTPD1, ETV6, F10, F2R, FCGR2A, FCGR2C, FOXP3, FYB1, HSP90B1, IFNAR1, IFNGR1, IFNGR2, IREB2, let-7, LYN, MAPKAP1, MDM2, mir-154, MIR4270, MS4A1, MTOR, MX2, NFE2L2, NR3C1, PAK2, PLEK, PSMD1, PSMD2, PTEN, PTGS2, RAP1A, SIRPA, SLAMF7, SLC11A1, SP1, SP3, SPHK2, ST3GAL6, SYK, TLR7, TNFSF10, TNNC1, VDR, WIPF1
Cellular Movement, Hematological System Development and Function, Immune Cell Trafficking, Inflammatory Response	Chemotaxis of leukocytes	9.65E-05	Decreased	-4.253	80	ADAM10, ADAM17, AIF1, APOA1, APP, AQP9, ARHGAP25, B4GALT1, CAMK1D, CCDC88A, CCL23, CCL5, CCR1, CSF1R, CSF3R, CUX1, CX3CR1, CXCL1, CXCL16, CXCL6, CXCL9, CXCR2, CXCR3, CYBB, DEFB103A/DEFB103B, DOCK2, ELN, F2R, FCGR2A, FPR1, FPR2, GIT2, HCK, HEBP1, HLA-G, HSPD1, ICAM1, IL1B, ITGA4, JAML, LCP1, LGMN, LILRB3, LITAF, LSP1, LYN, MAPKAP1, MPP1, MYLK, MYO1F, NCKAP1L, NEDD9, NINJ1, NR3C1, PDE4B, PF4, PLCG2, PRKG1, PTEN, PTPN6, RAC2, RHOA, RHOB, RICTOR, RPL13A, S100A14, S100A9, SPHK2, STK4, SWAP70, SYK, TAFA4, THBS2, TLR2, TLR4, TNFRSF1A, TNFSF14, TREM1, TREML2, WIPF1
Cancer, Organismal Injury and Abnormalities	Anogenital cancer	9.94E-05		0.443	1185	A1CF, ABCB5, ABCC2, ABHD3, ABHD8, ACBD3, ACO1, ACOX1, ACP3, ACSL1, ACTG1, ACTN1, ACTR2, ACYP1, ADAD1, ADAM10, ADAM15, ADAM17, ADGRA1, ADM, ADNP2, AGO2, AIF1, AIG1, AIPL1, AK9, AKAP12, ALDH3A1, ALDH5A1, ALDH9A1, ALG11, ALKBH1, ALKBH3, ALOX5AP, ALS2, AMPH, ANAPC13, ANGPTL5, ANKFY1, ANKRD13A, ANKRD13B, ANKRD42, ANKRD44, ANO5, ANO9, ANTXR2, ANXA2, ANXA3, ANXA5, AOPEP, AP1G1, AP5M1, APBA1, APBB1IP, APC, APOA1, APOA2, APOB, APOBEC3A, APOBEC3B, APOLD1, APP, AQP9, ARF4, ARHGAP19, ARHGAP25, ARHGAP29, ARHGDIB, ARHGEF25, ARID4B, ARIH2, ARL6IP1, ARMC3, ARMCX5-GPRASP2/GPRASP2, ARNT, ARNTL, ARRDC3, ARSD, ARVCF, ASB10, ASB7, ASMTL, ASXL1, ATF7IP, ATG13, ATG2B, ATG7, ATL3, ATN1, ATP6AP2, ATP6V1A, ATP6V1B2, ATP6V1H, ATP7B, ATRN, AURKB, B2M, B3GNTL1, BABAM2, BACH1, BAZ2B, BBS7, BCAS3, BCL2L11, BECN1, BGN, BLVRA, BMP2K, BMS1, BNIP2, BNIP3L, BNIPL, BOD1L1, BPIFB1, BRCA1, BRIP1, BRWD3, BTBD3, BTD, BTG2, BTK, BTN2A1, BTNL8, C10orf71, C12orf60, C16orf70, C17orf80, C18orf25, C1GALT1C1, C1orf87, C1RL, C22orf23, C7orf25, C9orf153, C9orf78, CACNA1E, CALCOCO2, CALU, CAMK2A, CAMSAP2, CAPN11, CAPZA1, CAPZB, CARD16, CARD8, CARMIL1, CARNS1, CASC2, CASC3, CASP8, CATSPERD, CAVIN2, CBX1, CBY1, CCDC174, CCDC40, CCDC47, CCDC88A, CCL5, CCNK, CCP110, CCT2, CD14, CD1E, CD300E, CD86, CDA, CDC25C, CDC5L, CDH12, CDKL1, CDKL5, CELF1, CELF2, CELSR3, CEP128, CEP72, CETN1, CFAP161, CFAP206, CFAP58, CFAP92, CFLAR, CHCHD5, CHD4, CHMP2A, CHMP3, CHPF, CHST11, CKMT2, CLASP1, CLEC4F, CLEC4M, CLEC9A, CLIC2, CLIC4, CLIP1, CLK2, CMSS1, CNP, CNPY3, CNR1, COG2, COG5, COL1A2, COL7A1, CORO1C, CPEB1, CPQ, CPSF7, CPT1A, CRB1, CREB1, CRKL, CRY2, CRYBG3, CSDE1, CSF1R, CSF3R, CST8, CT45A10/CT45A5, CTAG2, CTBS, CTLA4, CTNNA1, CTNND1, CTNND2, CTSB, CTSC, CTSZ, CUX1, CXCL1, CXCL16, CXCL6, CXCL9, CXCR2, CXCR3, CYBB, CYP24A1, CYP2A6 (includes others), CYP2W1, CYP4F2, CYP4F3, CYP51A1, CYTH4, DAB2, DCLRE1C, DDIT3, DDX17, DDX23, DDX27, DDX39A, DDX3X, DDX5, DEF6,

						DENND3, DENND5A, DET1, DGLUCY, DHCR7, DHRS7, DHX30, DHX38, DHX8, DIP2B, DKK3, DLG3, DLGAP4, DNAJA2, DNAJB11, DNAJB12, DNAJB5, DNAJB6, DNAJC17, DNAJC2, DNAJC7, DNM3, DOCK2, DOCK8, DOK5, DPF2, DPF3, DPH2, DPRX, DPYD, DSE, DUSP5, DYNC1LI1, DYRK1A, EBLN2, ECE1, ECPAS, EDEM3, EDRF1, EEF2K, EFCAB2, EFS, EHD3, EIF1AX, EIF1B, EIF3A, EIF3I, EIF4G3, ELAC1, ELF1, ELF3, ELL2, ELN, ELOA, EMC10, EMSY, ENTPD1, EOGT, EPB41L3, EPHB1, EPM2AIP1, ERCC5, ERO1A, ESS2, ETFDH, ETV3, ETV6, EVC2, EVI5L, EWSR1, EXD2, EXOC3L4, EXT1, EZR, F10, F11R, F13A1, F2R, F8, FAM126B, FAM136A, FAM13B, FAM209A, FAM214B, FAM217B, FAM72A, FASTKD2, FBLN2, FBXO33, FBXO38, FCAMR, FEZ1, FGD4, FGD6, FGGY, FGL2, FKBP5, FNBP1L, FOXL2, FOXO3, FOXP3, FPR1, FPR2, FRMD4B, FRS2, FTH1, FUBP3, FUS, FUT7, FYB1, FZD1, FZD3, G3BP2, GAB2, GADL1, GAL3ST1, GALC, GALNT1, GAPDH, GAS7, GASK1B, GATA5, GBE1, GC, GFOD2, GFRAL, GHITM, GIT2, GK, GLCE, GLE1, GLIPR1, GLT8D2, GLUL, GLYCTK, GLYR1, GNB4, GOLGB1, GPATCH1, GPATCH4, GPR21, GPR75, GRB2, GSTA1, GTDC1, GTF2H3, GTF3C3, GTPBP1, GUCA2B, GYG1, H2AC18/H2AC19, H2BC21, H3-3A/H3-3B, H3-5, HAL, HAMP, HAUS7, HBA1/HBA2, HBB, HBP1, HCCS, HCK, HCLS1, HDAC7, HDAC9, HDLBP, HECA, HELZ, HERC3, HGD, HIC2, HLA-A, HLA-C, HLA-E, HLA-G, HMCN2, HMOX1, HNRNPA1, HNRNPA2B1, HNRNPH1, HNRNPH2, HOTAIR, HOXA3, HOXA4, HOXA6, HS3ST4, HSD17B12, HSP90AA1, HSP90AB1, HSP90B1, HSPA13, HSPA5, HSPB7, HSPD1, HTATIP2, HTR1F, HVCN1, IDH3A, IER2, IFI16, IFNGR1, IFNGR2, IGF1, IGF1R, IGF2BP3, IGF2R, IGFBP4, IGSF6, IK, IL1B, IL1R2, ILF3, IMPG2, IP6K2, IQCD, IQSEC3, IRAG2, IREB2, IRX4, ITGA4, ITGAX, ITGB8, JADE1, JAK1, JAML, JMJD1C, JMJD4, JPH4, JPT1, JPX, KAT6A, KAT6B, KCNJ2, KCNJ4, KCTD20, KDM1B, KDM5A, KDM7A, KIAA0556, KIDINS220, KIF13A, KIF1A, KIF1C, KIF26B, KLF6, KLHL15, KLHL20, KRAS, KRT23, KRT34, L3MBTL3, LAMA5, LAMTOR5, LARP4, LARP6, LAS1L, LASP1, LAT2, LCOR, LCP1, LCT, LEFTY1, let-7, LETM2, LGALS8, LGMN, LGR5, LHCGR, LIAS, LILRA1, LIMK2, LINC00511, LINC01564, LIPM, LITAF, LMTK2, LONRF3, LRP2, LRP8, LRPAP1, LRRFIP1, LSM12, LSP1, LUZP2, LY6K, LY86, LY9, LYPLA2, LYVE1, LYZ, MAD2L1BP, MAFF, MAGT1, MAK, MAN2A2, MAP3K1, MAP3K2, MAP3K7CL, MAP4, MAP4K4, MAPKAP1, MAPRE1, MAPRE3, MARF1, MAX, MBP, MCCC2, MCL1, MCM7, MDM1, MDM2, MED23, MED30, MEF2C, MERTK, METTL21A, MEX3B, MGAT5, MICB, mir-101, mir-103, mir-122, mir-133, mir-154, mir-202, mir-24, mir-26, mir-28, mir-515, MKNK1, MLX, MMP14, MOB3A, MORN5, MPHOSPH10, MPND, MRPL15, MS4A1, MS4A14, MS4A4A, MS4A7, MSH6, MSMB, MSN, MT1A, MT1F, MT1X, MTCH2, MTDH, MTERF2, MTF1, MTFR2, MTHFD2, MTOR, MTRR, MTTP, MTURN, MUC1, MUC15, MYBBP1A, MYH14, MYH15, MYLK, MYLK2, MYO15A, MYO1F, MYO5A, MYO5B, MYOF, MYOG, NABP1, NACA2, NAMPT, NAP1L5, NAPB, NASP, NAT2, NCBP1, NCCRP1, NCF2, NCKAP1L, NCL, NCOA1, NCOA4, NDE1, NECAB2, NEDD9, NETO2, NFATC4, NFE2L2, NHSL1, NIM1K, NIN, NIPSNAP1, NLGN3, NLGN4X, NONO, NOTCH2, NOTCH2NLA/NOTCH2NLB, NOXRED1, NPC1L1, NPTN, NR3C1, NRBF2, NRDC, NSFL1C, NSRP1, NTRK1, NUAK2, NUBP1, NUDT7, NUMB, NUP160, NUP50, NUP62, NUP93, NXPE4, OAT, OAZ1, OBP2A, OPA1, OPN3, OR10R2, OR2A14, OR4D10, OR51A2, OR52L1, OR5AC2, OR5M1, OSBPL11, OTUD3, OXA1L, P2RY13, PABPC1, PACSIN2, PAK2, PARP8, PCLO, PCOLCE, PCYT1A, PDAP1, PDCD4, PDE4B, PDGFRA, PDIA3, PDIA5, PDK3, PDLIM5, PDS5B, PDZD8, PEAK1, PECAM1, PER2, PEX19, PF4, PHF12, PID1, PILRA, PIN4, PIP4P2, PIP5K1A, PITPNA, PITPNM1, PITX2, PIWIL1, PJA2, PKN3, PLAGL2, PLB1, PLCB3, PLCG2, PLCL1, PLEK, PLEKHA5, PLEKHA7, PLEKHM3, PLP1, PLXDC2, PLXNA4, PNN, PODNL1, POLD3, POLR1A, POTEH (includes others), POU4F2, PPHLN1, PPIF, PPM1D, PPP1CB, PPP1R12B, PPP1R17, PPP1R3B, PPP4R2, PPP6R3, PQBP1, PRC1, PRKAA1, PRKAR1A, PRKCD, PRKCG, PRKG1, PRL, PRPF38A, PRPF6, PRR12, PRRC2C, PRSS55, PSAP, PSMC1, PSMC2, PSMD1, PSMD12, PSMD2, PSMD4, PSMD7, PSME3, PTCHD4, PTEN, PTGFRN, PTGS2, PTPRE, PUDP, PURA, PUS3, PWWP3A, RAB31, RAB3GAP2, RAB9A, RABEP1, RABGAP1L, RAC2, RAD51C, RAD51D, RAF1, RALBP1, RALGPS1, RALGPS2, RAMP2, RAP1A, RAPGEF2, RASEF, RASSF2, RBM12B, RBM25, RBM47, RBM5, RBMS1, RBMXL3, RCBTB2, RCC2, RCOR3, REEP5, RFPL2, RFX3, RGCC, RGS2, RHBG, RHOA, RHOB, RICTOR, RIIAD1, RIN2, RIOK1, RIOK2, RIOK3, RIOX2, RIPK3, RNF103, RNF103-CHMP3, RNF121, RNF130, RNF149, RNF169, RNF20, RNF40, RPF2, RPGRIP1, RPL28, RPL39, RPL4, RPL5, RPS15, RPS6KA5, RRNAD1, RSPH3, RTCB, RTF1, RTN3, RTN4, RTTN, RUSC1, S100A14, S100A9, S100Z, SAAL1, SAMD4B, SART3, SAT1, SBF2, SCARB2, SCFD2, SCN9A, SCP2D1, SCRIB, SCRN1, SCRT2, SEC14L1, SEC24D, SEC61A2, SEL1L, SEMA3G, SENP2, SENP5, SERHL2, SERPINB3, SERPINB4, SERPINB8, SETDB1, SF3A3, SF3B1, SF3B6, SFRP4, SGK1, SH2B3, SH3BP2, SHISAL2B, SHROOM3, SIPA1L2, SIRPA, SIRPB1, SKP2, SLC16A11, SLC22A15, SLC22A18, SLC22A4, SLC22A5, SLC24A4, SLC25A2, SLC25A3, SLC25A32, SLC26A11, SLC31A1, SLC35F4, SLC36A1, SLC43A3, SLC4A1AP, SLC4A2, SLC6A6, SLC7A7, SLC8A1, SLC8A3, SLC9A9, SLITRK6, SMARCA2, SMARCC2, SMS, SMTN, SNAP91, SNRPB, SNRPF, SNX27, SOCS4, SOD2, SORL1, SOS2, SP1, SP100, SP110, SP3, SPACA5/SPACA5B, SPAG9, SPAST, SPATA31A6 (includes others), SPATA5, SPEF2, SPHK2, SPINK5, SPOP, SPOUT1, SPTBN4, SPTSSB, SQOR, SRD5A2, SRPK1, SRPK2, SRSF4, SRSF5, SSBP2, SSH1, SSH3, SSR1, ST6GALNAC2, ST8SIA4, STARD8, STAT3, STAU1, STEAP4, STK24, STK4, STX3, STXBP6, SUFU, SUSD6, SVIL, SWAP70, SWT1, SYK, SYNE4, SYT17, SZT2, TACC1, TAF1, TAF7, TAGLN2, TALDO1, TASP1, TBC1D12, TBC1D14, TBC1D8, TBC1D9, TBL1X, TBX5, TCAIM, TCF4, TCP1, TDGF1, TDRD1, TEKT4, TERF2IP, TET2, TFRC, TGOLN2, THBS2, THEG, THEMIS2, THOC5, THRAP3, TIFA, TIGAR, TIGD1, TIMMDC1, TJP1, TKFC, TLK2, TLR2, TLR4, TLR5, TLR7, TM2D2, TM6SF2, TM7SF3, TMEM140, TMEM43, TMEM70, TMEM86A, TMPRSS7, TMTC2, TNFRSF10D, TNFRSF1A, TNFSF10, TNIP1, TNNC1, TNNI3K, TOPORS, TOR1B, TPM3, TRAF3, TRANK1, TREML2, TRIM34, TRIM41, TRIM43/TRIM43B, TRIM46, TRIM5, TRIM55, TRIM64C, TRIM65, TRIO, TRIOBP, TRIP10, TRIP12, TRIP4, TRMT1, TRMT9B, TRNT1, TRPM6, TSG101, TSHZ3, TTC17, TTC26, TTF1, TTI2, TUBA1A, TUBA1B, TUBA1C, TUBB2A, TUBGCP3, U2AF1/U2AF1L5, UBAP2L, UBE2E3, UBE2F, UBE2J2, UBE3B, UBE4B, UBR2, UCK2, UEVLD, UGT2B11, UGT3A1, UNC5C, UQCRC2, USP15, USP19, USP32, USP4, UTP23, UTP4, VCAN, VCPIP1, VDAC2, VDR, VIM, VKORC1L1, VMP1, VNN2, VPS26C, VRK3, VTCN1,

						VTI1B, WASF2, WASF3, WDFY3, WDR19, WIPF1, WNK1, WNK3, WSB1, WWTR1, XAF1, XPNPEP3, XRCC5, YBX1, YPEL3, YPEL5, YWHAE, YWHAZ, ZAN, ZBTB21, ZEB2, ZFPM2, ZFYVE21, ZMPSTE24, ZMYM3, ZNF10, ZNF134, ZNF143, ZNF148, ZNF165, ZNF17, ZNF180, ZNF189, ZNF195, ZNF200, ZNF212, ZNF217, ZNF224, ZNF229, ZNF235, ZNF24, ZNF257, ZNF267, ZNF281, ZNF283, ZNF287, ZNF3, ZNF320, ZNF333, ZNF33A, ZNF33B, ZNF34, ZNF347, ZNF350, ZNF398, ZNF41, ZNF429, ZNF431, ZNF443, ZNF45, ZNF461, ZNF469, ZNF493, ZNF516, ZNF518A, ZNF525, ZNF528, ZNF534, ZNF548, ZNF555, ZNF558, ZNF565, ZNF567, ZNF568, ZNF570, ZNF585B, ZNF606, ZNF610, ZNF615, ZNF616, ZNF649, ZNF667, ZNF677, ZNF684, ZNF711, ZNF717, ZNF721, ZNF738, ZNF746, ZNF761, ZNF776, ZNF781, ZNF799, ZNF81, ZNF829, ZNF836, ZNF880, ZNF93, ZNFX1, ZRANB1, ZSCAN2, ZXDC
Cell-To-Cell Signaling and Interaction, Inflammatory Response	Response of monocytes	1.04E-04	Decreased	-2.772	11	CCR1, CD14, CD93, CLEC7A, FCGR2A, FPR1, PF4, SYK, TLR2, TLR4, TREM1
Cellular Movement	Migration of tumor cell lines	1.05E-04	Decreased	-6.859	189	ACTN1, ADAM10, ADAM15, ADAM17, AGO2, AIF1, AKAP12, ANGPTL4, ANXA2, APC, APP, ARF1, ARHGDIB, ARPC2, ARRDC3, ATOX1, BRCA1, CALML3, CALU, CASP8, CCDC88A, CCL5, CGB3 (includes others), COL7A1, CRKL, CSF1R, CTNND1, CTSB, CTSZ, CXCL9, CYP2J2, DAB2, DEF6, DNAJB6, DOCK8, DPP10-AS1, DPYSL2, DSE, DUSP5, EPHB1, EPO, EYA3, EZR, F2R, FAIM2, FBLN2, FGD4, FNBP1L, FOXO3, FOXP3, FTX, GAB1, GAB2, GMFG, GSE1, HMOX1, HNRNPA2B1, HOTAIR, HOXA4, HSBP1, HSP90AA1, HSP90B1, HSPA1A/HSPA1B, HTATIP2, HVCN1, IFNAR1, IGF1, IGF1R, IGF2BP3, IGFBP4, IL1B, ILF3, IP6K2, ITGA4, JAK1, JPX, KDM5A, KIDINS220, KLF6, KRAS, LAMA5, LASP1, let-7, LIMK2, LINC00887, LUCAT1, LYN, LYVE1, MAP3K1, MAP4, MAP4K4, MDM2, MERTK, MGAT5, mir-122, mir-154, mir-24, mir-26, mir-28, mir-299, mir-515, MMP14, MSN, MTDH, MTOR, MUC1, MUC13, MYOF, NCL, NEDD9, NFATC4, NFE2L2, NINJ1, NKD2, NOTCH2, NREP, NUMB, P2RX1, PACSIN2, PAK2, PDCD4, PDGFRA, PEAK1, PECAM1, PHACTR1, PITX2, PLCL1, PPIF, PRKAA1, PRKCD, PRKG1, PRL, PSMD10, PTEN, PTGS2, PTPN6, RAB21, RAC2, RAF1, RALB, RALBP1, RAP1A, RFFL, RHOA, RHOB, RICTOR, RIOK3, RNF11, RNF20, S100A9, SCRIB, SDCBP, SEMA4A, SERPINB3, SFRP4, SH2B3, SIRPA, SKP2, SMAD1, SOCS4, SOD2, SP1, SPHK2, SRGN, SSH1, STAT3, STK24, SYK, TAZ, TCAF1, TCF4, TDGF1, THBS2, TLR2, TLR4, TNFSF10, TPD52L1, TPM3, TRIO, TRIP10, USP4, VCAN, VDAC1, VIM, WASF2, WWTR1, YBX1, ZEB2, ZFYVE21
Cellular Development, Cellular Growth and Proliferation, Hematological System Development and Function, Hematopoiesis, Lymphoid Tissue Structure and Development, Tissue Development	Development of mononuclear leukocytes	1.06E-04	Decreased	-4.709	149	ADAM10, ADAM17, ADGRG3, ADM, APC, APP, ARNTL, ASXL1, B2M, BCL2L11, BRCA1, BTK, CASP8, CCL23, CCL5, CD14, CD86, CDA, CFLAR, CHD4, CLEC4M, CLEC6A, CREB1, CSF1R, CSF3R, CTLA4, CXCL1, CXCR2, CXCR3, CYP26B1, DCLRE1C, DEF6, DNAJA2, DOCK2, DOCK8, DUSP5, ELF1, ELF3, ENTPD1, EPHB1, EPO, EZR, FCAMR, FCGR2A, FOXO3, FOXP3, FUT7, FYB1, GIMAP4, GMPR2, GRB2, HDAC7, HDAC9, HLA-A, HLA-G, HOXA7, HSP90AA1, HSP90B1, HSPD1, ICAM1, IFI16, IFNAR1, IFNGR1, IFNGR2, IGF1, IGF1R, IGF2R, IL1B, IL1RN, IRF8, ITGA4, ITGB8, JAK1, KRAS, LAT2, LCP1, LCP2, let-7, LGALS8, LILRA2, LSP1, LTBR, LY9, LYN, MAPKAP1, MBP, MCL1, MDM2, MEF2C, MERTK, mir-24, MMP14, MPZL2, MS4A1, MSN, MTOR, NCKAP1L, NFKBIZ, NMT1, NOTCH2, NTRK1, PF4, PHLPP1, PLCG2, PLP1, PRKAA1, PRKCD, PRL, PSAP, PSMB8, PTEN, PTGS2, PTPN6, RAD52, RAF1, RBPJ, RGCC, RHOA, RICTOR, RIPK2, RIPK3, SEMA4A, SH2B3, SKP2, SP3, SPINK5, STAT3, SWAP70, SYK, TCF4, TDP2, THEMIS2, THOC5, TLR2, TLR4, TLR5, TLR7, TNFRSF1A, TNFSF10, TNFSF4, TRAF3, TREM1, TYROBP, USP15, USP4, WIPF1, XRCC5, ZBTB46, ZEB2
Cell Death and Survival	Apoptosis of fibroblast cell lines	1.07E-04		-1.876	72	APP, ASAH1, ATG3, ATN1, ATP6AP2, BCL2L11, BECN1, BID, BNIP3L, BRCA1, CA4, CASP8, CFLAR, CLIC4, CRADD, CWC15, DDIT3, DDX3X, EIF3I, FOXO3, FTH1, HNRNPA1, HSPA5, HSPD1, IFI16, IFNAR1, IGF1, INSM2, KRAS, L3MBTL2, MAP3K1, MAPKAP1, MCL1, MDM2, MLKL, MTCH2, MTF1, MTOR, MUC1, MXD1, NFATC4, NFE2L2, NTRK1, NUDT13, PDIA3, PLAGL2, PPM1D, PRKAR1A, PRKCD, PRKCG, PTEN, RALB, RALBP1, RHOA, RHOB, RIPK3, RPS6KA5, RTN4, SERPINB3, SERPINB4, SF3B6, SKP2, SPHK2, STK4, SYK, TMEM107, TNFRSF1A, TNFSF10, TNIP1, UNC5C, VDAC1, VIM
Cancer, Cellular Movement, Organismal Injury and Abnormalities, Tumor Morphology	Invasion of tumor cells	1.12E-04	Decreased	-3.141	43	APC, CD14, COL7A1, CTNND2, CTSB, CTSZ, CXCL1, CXCL6, EZR, F2R, FOXO3, GAB1, GAB2, HDLBP, HMOX1, IGF1, KRAS, let-7, LIMK2, mir-103, mir-133, MMP14, NEDD9, NFE2L2, NOTCH2, NUAK2, PARK7, PDCD4, PSMD10, PTEN, PTGS2, RALBP1, RHOA, RHOB, S100A9, SCRIB, SSX2IP, STAT3, SYK, TRAF3, VCAN, WASF3, ZFYVE21
Protein Synthesis	Expression of protein	1.13E-04		0.531	89	ACO1, ADM, AGO2, ALDH3A1, ALKBH1, APP, ATF5, BTG2, BTK, CASC3, CAV3, CNBP, CPEB1, CREB1, DDX3X, EEF2K, EIF1AX, EIF3A, EIF3G, EIF3I, EIF4G3, EIF4H, FOXO3, FUS, GAB2, GAPDH, HCK, HELZ, HSPA1A/HSPA1B, HSPA5, IFNAR1, IGF1, IGF2BP3, IL1B, ILF3, IREB2, KRAS, LARP4B, let-7, LYN, MARS1, MKNK1, MMP14, MRPL15, MRPL18, MRPL28, MRPL55, MRPS10, MRPS18A, MRRF, MTOR, MTRF1L, MYBBP1A, NCBP1, NCL, NR3C1, OXA1L, PABPC1, PDCD4, PHLPP1, PIWIL1, PPM1G, PRKAA1, PTCD3, RBM4, RGS2, RNASET2, RPL13A, RPL18, RPL18A, RPL28, RPL38, RPL39, RPL4, RPL5, RPS15, S100A9, SOD2, SRSF3, STAT3, STAU1, SWAP70, SYK, TNFSF10, TNIP1, VDR, WARS1, YBX1, ZFPM2

Cellular Development, Cellular Growth and Proliferation, Hematological System Development and Function, Hematopoiesis, Lymphoid Tissue Structure and Development, Tissue Development	Differentiation of mononuclear leukocytes	1.16E-04	Decreased	-4.909	149	ADAM10, ADAM17, ADGRG3, APC, APP, ARNTL, ASXL1, B2M, BCL2L11, BRCA1, BTK, CASP8, CCL23, CCL5, CD14, CD86, CDA, CFLAR, CHD4, CLEC4M, CLEC6A, CREB1, CSF1R, CSF3R, CTLA4, CXCL1, CXCR2, CXCR3, CYP26B1, DCLRE1C, DEF6, DNAJA2, DOCK2, DOCK8, DUSP5, ELF1, ELF3, ENTPD1, EPHB1, EPO, EZR, FCAMR, FCGR2A, FOXO3, FOXP3, FUT7, FYB1, GAB2, GIMAP4, GMPR2, GRB2, HDAC7, HDAC9, HLA-A, HLA-G, HOXA7, HSP90AA1, HSP90B1, HSPD1, ICAM1, IFI16, IFNAR1, IFNGR1, IFNGR2, IGF1, IGF1R, IGF2R, IL1B, IL1RN, IRF8, ITGA4, ITGB8, JAK1, KRAS, LAT2, LCP1, LCP2, let-7, LGALS8, LILRA2, LSP1, LTBR, LY9, LYN, MAPKAP1, MBP, MCL1, MDM2, MEF2C, MERTK, mir-24, MMP14, MPZL2, MS4A1, MSN, MTOR, NCKAP1L, NFKBIZ, NMT1, NOTCH2, NTRK1, PF4, PHLPP1, PLCG2, PLP1, PRKAA1, PRKCD, PRL, PSAP, PSMB8, PTEN, PTGS2, PTPN6, RAD52, RAF1, RBPJ, RGCC, RHOA, RICTOR, RIPK2, RIPK3, SEMA4A, SH2B3, SKP2, SP3, SPINK5, STAT3, SWAP70, SYK, TCF4, TDP2, THEMIS2, THOC5, TLR2, TLR4, TLR5, TLR7, TNFRSF1A, TNFSF10, TNFSF4, TRAF3, TREM1, TYROBP, USP15, USP4, WIPF1, XRCC5, ZBTB46, ZEB2
Cellular Function and Maintenance, Hematological System Development and Function, Inflammatory Response	Engulfment by macrophages	1.22E-04	Decreased	-2.772	35	APP, ATG7, BECN1, BTK, CD14, CD93, CLEC4M, CLEC6A, CLIC4, CSF1R, DEF6, DOCK2, FCGR2A, GAB2, HCK, HMOX1, IL1B, let-7, MERTK, MEX3B, mir-24, NCKAP1L, PTEN, RAB11A, RGCC, S100A9, SH3BP2, SIRPA, SIRPB1, SLAMF7, TLR2, TLR4, TREML2, TYROBP, WNK1
Cancer, Gastrointestinal Disease, Organismal Injury and Abnormalities	Duodenal carcinoma	1.22E-04			12	APC, FCRLA, GSE1, HSP90AA1, HSP90AB1, HSP90B1, KBTBD12, KRAS, MSH6, PRKCD, PRKCG, U2AF1/U2AF1L5
Connective Tissue Disorders, Immunological Disease, Inflammatory Disease, Inflammatory Response, Organismal Injury and Abnormalities, Skeletal and Muscular Disorders	Juvenile rheumatoid arthritis	1.23E-04			47	ADM, APOA1, C9orf78, CCL5, CCR1, CD86, CDA, CLIC2, DNAJA4, DYNLL1, F11R, FGL2, FOXO3, FPR2, GALNT1, HDAC7, HLA-G, HNMT, HNRNPA1, HSPA1A/HSPA1B, IL1B, IL1RN, JAK1, JMJD1C, KRAS, MAP3K2, MCL1, NOM1, NR3C1, NUMB, P2RY13, PECAM1, PTGS2, PTMA, PTPRE, RALB, S100A9, SF3B6, SORL1, SWT1, TALDO1, TNFRSF10C, TRIO, VTCN1, WNK1, ZNF281, ZNF326
Molecular Transport	Export of heavy metal	1.24E-04		-1.071	8	ACO1, APP, ATOX1, ATP7B, FTH1, HAMP, HMOX1, SLC11A1
Cell Death and Survival	Cell death of breast cell lines	1.29E-04		1.257	26	ATG7, BCL2L11, BID, BRCA1, BTK, CASP8, CFLAR, CLCA2, DAB2, DYNLL1, FOXO3, IGF1, IGF1R, KRAS, LIMS1, MERTK, MTOR, PPM1D, PRL, SERPINB3, SGK1, TAZ, TNFSF10, UBE2B, WWTR1, YWHAZ
Free Radical Scavenging	Metabolism of reactive oxygen species	1.32E-04	Decreased	-3.941	114	ACOX1, ADGRE2, ALDH3A2, ALS2, ANXA2, AOPEP, APOA1, APP, ARHGDIB, ARNT, ATG7, ATP6AP2, BECN1, BID, BNIP3L, BRCA1, CASP8, CCL5, CD14, CLCN3, CLEC7A, CTLA4, CXCL9, CYBB, CYP2A6 (includes others), DDIT3, DOCK2, ENTPD1, EPO, ERO1A, F2R, FCGR2A, FOXL2, FOXO3, FPR1, FPR2, FTH1, FTL, GAB2, HBA1/HBA2, HBB, HCK, HMOX1, HSP90AB1, HVCN1, ICAM1, IGF1, IL1B, ITGAX, ITM2B, JAK1, KRAS, LCP2, let-7, LYN, MLKL, MMP14, MS4A1, MTOR, MUC1, MYLK, NAMPT, NCF2, NDUFS1, NDUFS3, NFE2L2, NTRK1, PARK7, PCK1, PECAM1, PGAM2, PLAGL2, PLCB3, PLCG2, PRKAA1, PRKCD, PSMB8, PTEN, PTGS2, PTPN6, RAC2, RAP1A, RBPJ, RHOA, RIPK3, RTN4, SAT1, SIGLEC9, SLC8A1, SLU7, SNAP23, SOD2, SPAG9, STAT3, SYK, TAFA4, TAZ, TFRC, TIGAR, TLR2, TLR4, TLR5, TLR7, TMBIM6, TNFRSF1A, TNFSF14, TRAF3, TREML2, TXNRD1, TYROBP, UQCRC2, VDAC1, VDR, YWHAZ
Cellular Development, Cellular Growth and Proliferation,	Hematopoiesis of mononuclear leukocytes	1.33E-04	Decreased	-4.819	148	ADAM10, ADAM17, ADGRG3, APC, APP, ARNTL, ASXL1, B2M, BCL2L11, BRCA1, BTK, CASP8, CCL23, CCL5, CD14, CD86, CDA, CFLAR, CHD4, CLEC4M, CLEC6A, CREB1, CSF1R, CSF3R, CTLA4, CXCL1, CXCR2, CXCR3, CYP26B1, DCLRE1C, DEF6, DNAJA2, DOCK2, DOCK8, DUSP5, ELF1, ELF3, ENTPD1, EPHB1, EPO, EZR, FCAMR, FCGR2A, FOXO3, FOXP3, FUT7, FYB1, GIMAP4, GMPR2, GRB2, HDAC7, HDAC9, HLA-A, HLA-G, HOXA7, HSP90AA1, HSP90B1, HSPD1, ICAM1, IFI16, IFNAR1, IFNGR1, IFNGR2, IGF1, IGF1R, IGF2R, IL1B,

Hematological System Development and Function, Hematopoiesis, Lymphoid Tissue Structure and Development, Tissue Development						IL1RN, IRF8, ITGA4, ITGB8, JAK1, KRAS, LAT2, LCP1, LCP2, let-7, LGALS8, LILRA2, LSP1, LTBR, LY9, LYN, MAPKAP1, MBP, MCL1, MDM2, MEF2C, MERTK, mir-24, MMP14, MPZL2, MS4A1, MSN, MTOR, NCKAP1L, NFKBIZ, NMT1, NOTCH2, NTRK1, PF4, PHLPP1, PLCG2, PLP1, PRKAA1, PRKCD, PRL, PSAP, PSMB8, PTEN, PTGS2, PTPN6, RAD52, RAF1, RBPJ, RGCC, RHOA, RICTOR, RIPK2, RIPK3, SEMA4A, SH2B3, SKP2, SP3, SPINK5, STAT3, SWAP70, SYK, TCF4, TDP2, THEMIS2, THOC5, TLR2, TLR4, TLR5, TLR7, TNFRSF1A, TNFSF10, TNFSF4, TRAF3, TREM1, TYROBP, USP15, USP4, WIPF1, XRCC5, ZBTB46, ZEB2
Cellular Movement	Emigration of cells	1.36E-04	Decreased	-3.055	18	ADAM17, BTK, CXCL1, CXCL6, F11R, FCGR2A, FPR2, IL1B, ITGA4, LAMA5, LSP1, LTBR, PECAM1, RHOA, SIRPA, STK4, TNFRSF1A, TRIM55
Cell-To-Cell Signaling and Interaction	Response of myeloid leukocytes	1.36E-04	Decreased	-2.427	30	ADGRE2, APP, BECN1, CD14, CEACAM3, CLCN3, CLEC6A, CXCL1, DOCK2, FCGR2A, FPR1, HCK, HMOX1, ICAM1, IFNAR1, IL1B, ITGA4, ITGAX, LILRB3, LYN, MERTK, PARK7, RAB11A, S100A9, STAT3, SYK, TLR2, TLR4, TREM1, TYROBP
Cell-To-Cell Signaling and Interaction, Hematological System Development and Function, Immune Cell Trafficking, Inflammatory Response	Binding of neutrophils	1.41E-04	Decreased	-2.056	29	ADAM10, ADAM17, ADGRE2, APOA1, B4GALT1, CSF3R, CXCL1, CXCR2, CYBB, FCGR2A, FUT7, HCK, ICAM1, IL1B, ITGA4, ITGAX, LCP1, LGALS8, LILRB3, LSP1, LYN, MGAT5, PF4, PLCB3, PTPN6, S100A9, TLR2, TLR4, TLR5
Infectious Diseases	Replication of HIV	1.46E-04		-1.807	34	ADAM10, ANXA5, APOBEC3B, ARHGDIB, ARNTL, ATG7, BECN1, CCL5, CCNK, CFLAR, DDX5, DYRK1A, FAS-AS1, GALC, HCK, IL1B, MED30, NUP62, P2RX1, PACSIN2, PDE8A, RAB11A, RAB9A, RAF1, S100A9, SNAPIN, SRSF1, STAT3, TLR2, TLR4, TLR7, TNFSF10, TRIM5, TSG101
Cellular Movement, Hematological System Development and Function, Immune Cell Trafficking	Cell movement of mononuclear leukocytes	1.50E-04	Decreased	-4.973	106	ADAM10, ADAM17, AIF1, ANXA2, APBB1IP, APC, APOA1, APP, ATG7, ATRN, BGN, BTK, CCL23, CCL5, CCR1, CCR10, CD86, CTLA4, CUX1, CX3CR1, CXCL1, CXCL16, CXCL6, CXCL9, CXCR2, CXCR3, CYP26B1, DEF6, DEFB103A/DEFB103B, DOCK2, DOCK8, DPYSL2, EFS, ELN, EZR, F11R, F2R, FOXP3, FPR1, FPR2, FUT7, FYB1, HCK, HCLS1, HEBP1, HLA-A, HLA-G, HMOX1, HSPD1, ICAM1, IFNAR1, IFNGR1, IL1B, ITGA4, ITGAX, JAK1, KCNE3, KRAS, LCP1, LCP2, LGMN, LTBR, MAP3K2, MAPKAP1, mir-133, MMP14, MSN, MTOR, MYLK, NEDD9, NFKBIZ, NINJ1, NR3C1, PECAM1, PF4, PILRA, PLCB3, PRKAA1, PRKCD, PROK2, PTEN, PTGS2, RAC2, RAP1A, RHOA, RICTOR, S100A14, SCRIB, SERPINB3, SIRPA, SOS2, SPHK2, STAT3, STK4, SWAP70, THBS2, TLR2, TLR4, TLR7, TNFRSF1A, TNFSF10, TNFSF14, TNFSF4, TNIP1, VTCN1, WIPF1
Cellular Assembly and Organization, Cellular Function and Maintenance	Formation of vesicles	1.62E-04		-1.262	38	ANKFY1, ANXA13, ANXA2, ANXA5, ARF1, ATG13, ATG14, ATG3, ATG7, BECN1, BRCA1, CASP8, CHMP2A, CHMP3, CHMP4B, CHMP6, FOXO3, LITAF, MTOR, MYH14, PCLO, PITPNA, PRL, RAB11A, RHOA, SNAP23, SNAP91, TAZ, TBC1D14, TBK1, TGOLN2, TLR4, TMBIM6, TSG101, VPS25, VPS4B, WASF2, WNK1
Cell Death and Survival	Apoptosis of colorectal cancer cell lines	1.62E-04		0.94	61	ADAM17, ADIPOR1, APC, ATG7, BCL2L11, BID, BTK, CASP8, CD14, CFLAR, CNR1, DDIT3, DFFA, EZR, FOXO3, GLIPR1, GSTA1, GUCA2A, GUCA2B, HOTAIR, HSPD1, IGF1, IGF2R, IL1B, IP6K2, IRF8, KRAS, LGALS8, LGR5, LIMS1, LUCAT1, MCL1, mir-154, mir-515, MT1F, MUC1, NFE2L2, PARK7, PDE4B, PECAM1, PHLPP1, PRKCD, PRKG1, PTGS2, RAF1, RASSF3, RHOA, RICTOR, SGK1, SPHK2, SRPK1, STAU1, TCF4, TLR4, TM9SF4, TNFRSF10C, TNFRSF1A, TNFSF10, VPS35, XRCC5, YWHAE
Hematological System Development and Function	Hemostasis	1.63E-04		-1.052	73	ANXA2, ANXA5, APLP2, APP, ARNTL, C1GALT1C1, C4BPB, CALU, CAPZA1, CAPZB, CARMIL1, CCL5, CD84, CLEC1B, COL1A2, CYBB, CYP4F2, DOCK8, EHD3, ENTPD1, EPO, F10, F13A1, F2R, F8, FCGR2A, FYB1, GATA5, H3-3A/H3-3B, H3C1, H3C13, HBB, HCK, ICAM1, JMJD1C, LCP2, LRP8, LRPAP1, LYN, MAFF, MERTK, P2RX1, PDGFRA, PECAM1, PF4, PLCB3, PLCG2, PLEK, PRKAR1A, PRKCD, PRKCG, PRKG1, PTEN, PTGS2, PTPN6, RAB27A, RAB5A, RAD51C, RAF1, RAP1A, RHOA, SGK1, SH2B3, ST6GALNAC2, SYK, THBS2, TLR2, TLR4, TREM1, VCAN, WIPF1, YWHAZ, ZFPM2
Cell-To-Cell Signaling and Interaction, Cellular Function and Maintenance,	Phagocytosis of tumor cell lines	1.65E-04	Decreased	-2.608	36	ACTR2, APPL2, ARPC2, BTK, CD93, CLIP1, DEF6, DET1, DOCK2, FCGR2A, GRB2, HCK, HMOX1, ICAM1, JAK1, KAT6A, KCTD5, MERTK, NCKAP1L, PIP5K1A, PLEK, PRKCD, PSMD4, PTPN6, RAB11A, RAB31, RALB, RHOA, RIT1, SLAMF7, TM2D2, TYROBP, UBE2L3, VIM, WASF2, ZNF217

Inflammatory Response						
Cell-To-Cell Signaling and Interaction, Cellular Function and Maintenance, Inflammatory Response	Phagocytosis of antigen presenting cells	1.76E-04	Decreased	-3.119	35	APOA1, APOA2, APP, ATG7, BECN1, BTK, CD14, CD93, CLEC4M, CLEC6A, CLIC4, CSF1R, DEF6, DOCK2, FCGR2A, GAB2, HCK, HMOX1, IL1B, let-7, MERTK, MEX3B, mir-24, NCKAP1L, PTEN, RAB11A, RGCC, S100A9, SH3BP2, SIRPA, SIRPB1, SLAMF7, TLR2, TLR4, TYROBP
Cellular Movement	Cell rolling	1.79E-04	Decreased	-2.453	23	ADAM17, ARHGAP25, BTK, CD14, CHST1, CXCL1, CXCR2, FCGR2A, FPR2, FUT7, FYB1, GALNT1, HCK, ICAM1, IL1B, ITGA4, LCP2, LYN, MGAT5, RAC2, ST6GALNAC2, SWAP70, TLR4
Cellular Movement	Chemotaxis of myeloid cells	1.79E-04	Decreased	-4.095	65	AIF1, APOA1, APP, AQP9, ARHGAP25, B4GALT1, CAMK1D, CCDC88A, CCL23, CCL5, CCR1, CSF1R, CSF3R, CX3CR1, CXCL1, CXCL6, CXCL9, CXCR2, CXCR3, CYBB, DEFB103A/DEFB103B, DOCK2, ELN, F2R, FCGR2A, FPR1, FPR2, GIT2, HCK, HEBP1, ICAM1, IL1B, ITGA4, JAML, LGMN, LILRB3, LITAF, LSP1, LYN, MPP1, MYO1F, NCKAP1L, NINJ1, PDE4B, PF4, PRKG1, PTEN, PTPN6, RAC2, RHOA, RHOB, RICTOR, RPL13A, S100A14, S100A9, SPHK2, SWAP70, SYK, TAFA4, THBS2, TLR2, TLR4, TNFRSF1A, TREM1, TREML2
Cellular Movement	Cell movement of leukemia cell lines	1.95E-04	Decreased	-4.108	37	ACTN1, ADAM10, AIF1, ANXA2, APP, CCL23, CCL5, CXCL1, DEFB103A/DEFB103B, DOCK2, DOCK8, DPYSL2, F2R, FGD4, FPR1, FPR2, FUT7, FYB1, GAB1, GC, GMFG, ITGA4, KIDINS220, KRAS, LCP2, LYN, MTOR, NEDD9, P2RX1, PAK2, RHOA, RICTOR, SSH1, ST6GALNAC2, STAT3, SYK, WARS1
Cellular Movement, Hematological System Development and Function, Immune Cell Trafficking, Inflammatory Response	Migration of neutrophils	1.96E-04	Decreased	-2.647	31	ADAM10, ADAM15, BTK, CCL5, CCR1, CXCL1, CXCL6, CXCL9, CXCR2, CYBB, F10, FPR1, HCK, ICAM1, IL1B, LSP1, MGAT5, mir-133, MYLK, MYO1F, PDE4B, PECAM1, PPM1D, PTEN, PTPN6, RTN4, S100A9, TLR2, TLR4, TLR7, TNFRSF1A
Cellular Movement, Hematological System Development and Function, Immune Cell Trafficking	Transmigration of leukocytes	1.99E-04	Decreased	-3.76	35	ADAM10, ADAM15, ADAM17, APP, ARHGAP25, CCL5, CCR1, CD86, CXCL1, CXCL9, CXCR2, CXCR3, DOCK8, F11R, FPR1, GALNT1, ICAM1, IL1B, ITGA4, ITGAX, MMP14, MTOR, MYLK, MYO1F, NINJ1, PECAM1, PTPN6, RAC2, RAP1A, RHOA, RTN4, SIRPA, TLR2, TNFRSF1A, TRIO
Endocrine System Disorders, Metabolic Disease, Organismal Injury and Abnormalities	Insulin resistance of cells	2.04E-04		-1.026	9	C1QL3, CCDC88A, CYBB, DYRK1A, PTEN, SOD2, STAT3, TLR4, TNFRSF1A
Cellular Assembly and Organization, Cellular Function and Maintenance	Organization of cytoskeleton	2.09E-04	Decreased	-6.574	292	ABITRAM, ACTG1, ACTN1, ACTR2, ADAM10, ADM, AKAP12, ALKBH1, ALS2, ANGPTL4, AP1G1, APBA1, APBB1IP, APC, APC2, APLP2, APP, APPL2, ARF1, ARHGAP17, ARHGAP25, ARHGEF25, ARHGEF9, ARPC2, ARPC5, ASB7, ATAT1, ATG7, ATRN, ATXN3, AURKB, BASP1, BCAS3, BECN1, BMP2K, BRCA1, BRWD3, BTBD3, BTG2, BTK, CALML3, CALU, CAMK1D, CAMK1G, CAMK2A, CAMSAP2, CAP1, CAPZB, CARMIL1, CAV3, CBY1, CCDC88A, CCL5, CCP110, CDKL5, CELSR3, CEP72, CIBAR1, CLASP1, CLEC1B, CLIP1, CNP, CNR1, CORO1C, CREB1, CRKL, CSF1R, CTLA4, CTNND1, CTNND2, CUX1, CX3CR1, CXCL1, CXCL9, CXCR2, CXCR3, CYBB, DKK3, DLG3, DNAJB6, DNM3, DOCK2, DPYSL2, DRG1, DYNC1LI1, DYNLL1, DYRK1A, EIF4G3, EMC10, EPB41L3, EPB41L5, EPHA8, EPHB1, EPO, ERC2, EVI5L, EZR, F11R, F13A1, F2R, FARP2, FCGR2A, FEZ1, FGD4, FNBP1L, GAB1, GAPDH, GAS7, GMFG, GPRIN1, GPSM2, HBP1, HCK, HOXA4, HSP90AA1, HSP90AB1, ICAM1, IFT88, IGF1, IGF1R, IL1B, ITGA4, ITGB8, KCNJ2, KIDINS220, KIF13B, KIF1C, KLF7, KNSTRN, KRAS, KRT6C, LAMA5, LARP4, LASP1, LCP1, LCP2, LHCGR, LIMK2, LRP2, LRP8, LRPAP1, LSP1, LYN, MAP3K1, MAP4, MAPRE1, MAPRE3, MAST3, MBP, MERTK, MGAT5, mir-138, mir-26, MPP1, MSN, MTOR, MYLK, MYO1F, MYO5A, MYO5B, NCF2, NCKAP1L, NDC80, NDE1, NDEL1, NEDD1, NEDD9, NFATC4, NIN, NINJ1, NLGN3, NTRK1, NUAK2, NUMB, NUP160, NUP62, OPA1, P2RX1, PACSIN2, PAK2, PCLO, PDGFRA, PDIA3, PDZD8, PF4, PHACTR1, PIP5K1A, PITPNA, PITPNM1, PJA2, PLCG2, PLEK, PLXNA4, POU4F2, PQBP1, PRC1, PRKAA1, PRKCD, PRKCG, PRKG1, PTEN, PTF1A, PTGS2, PTPRE, RAB11A, RAB21, RAB31, RAB5A, RAB8A, RAC2, RAF1, RALB, RALBP1, RAP1A, RAPGEF2, RFX3, RGMA, RHOA, RHOB, RICTOR, RIT1, RNF6, RPL4, RTN3, RTN4, RUFY3, S100A9, SEMA3G, SEMA4A, SEMA4F, SGK1, SHROOM3, SIAH1, SIRPA, SIRPB1, SLITRK6, SMAD1, SNAP29, SNAP91, SNAPIN, SP1, SPAST, SPATA13, SPTBN4, SRGAP3, SSH1, SSH3, SSX2IP, STAT3, STIP1, STK24, STK35, STK38L, STX3, SWAP70, SYK, TACC1, TBC1D30, TBK1, TESK2, TJP1, TLR4,

						TLR7, TMEM107, TMOD3, TNFRSF1A, TNFSF10, TPM3, TRAF3IP1, TRIM46, TRIO, TRIOBP, TRIP10, TTC26, TUBGCP3, TXNRD1, TYROBP, UBAP2L, UBE4B, VAMP4, VIM, VTCN1, WASF2, WASF3, WDR19, WDR60, WIPF1, WWTR1, YBX1, ZEB2, ZMYM3, ZRANB1
Cellular Movement, Hematological System Development and Function, Immune Cell Trafficking, Inflammatory Response	Transmigration of phagocytes	2.12E-04	Decreased	-2.855	24	ADAM15, ADAM17, APP, CCL5, CXCL1, CXCR2, DOCK8, F11R, FPR1, ICAM1, IL1B, ITGA4, ITGAX, MMP14, MYLK, MYO1F, NINJ1, PECAM1, PTPN6, RHOA, RTN4, SIRPA, TLR2, TNFRSF1A
Cell-To-Cell Signaling and Interaction	Binding of myeloid cells	2.25E-04	Decreased	-3.723	50	ADAM10, ADAM17, ADGRE2, APOA1, APP, B4GALT1, BTK, CCL5, CCR1, CD14, CLEC4M, CNR1, CSF3R, CTSZ, CXCL1, CXCR2, CYBB, F10, F2R, FCGR2A, FPR1, FPR2, FUT7, HCK, ICAM1, IL1B, ITGA4, ITGAX, LCP1, LGALS8, LILRB3, LSP1, LYN, MGAT5, MSN, NOTCH2, PAK2, PECAM1, PF4, PLCB3, PTGS2, PTPN6, RAC2, RHOA, RHOB, S100A9, SWAP70, TLR2, TLR4, TLR5
Cell-To-Cell Signaling and Interaction	Binding of tumor cell lines	2.28E-04	Decreased	-4.26	89	ADAM10, ADAM15, ADAM17, AGO2, AKAP12, ANGPTL4, ANXA2, ANXA5, APP, B4GALT1, CASP8, CD14, CHST6, CLCA2, CLEC4M, CLEC7A, CTSB, CTSZ, CXCL9, CXCR2, CXCR3, CYBB, CYP2J2, DAB2, DKK3, DOCK8, DSE, ELN, EPHB1, F10, F11R, F2R, FCGR2A, FUT7, FYB1, GAL3ST1, GMFG, HCK, HLA-A, HOXA4, HSP90B1, HSPA5, ICAM1, IGF1, IGF1R, IL1B, IL1R2, IP6K2, ITGA4, ITGAX, LAMA5, LASP1, LCP2, LGALS8, LRP2, LTBR, MGAT5, mir-103, MMP14, MTOR, MUC1, MUC13, NCL, PAK2, PECAM1, PITX2, PRKAA1, PRL, PSMD4, PTPN6, RAB21, RAF1, RAMP2, RAP1A, RHOA, SERPINB3, SFRP4, SH2B3, SRGN, ST6GALNAC2, STAT3, TFRC, TJP1, TLR2, TLR4, TLR5, VCAN, WWTR1, ZEB2
Cellular Function and Maintenance	Macropinocytosis	2.38E-04		-1.115	13	APC, CARMIL1, DOCK2, EZR, FRS2, KRAS, MAPKAPK3, NCL, NTRK1, RHOA, RHOB, TLR4, TNFRSF1A
Connective Tissue Disorders, Immunological Disease, Inflammatory Disease, Inflammatory Response, Organismal Injury and Abnormalities, Skeletal and Muscular Disorders	Polyarticular juvenile rheumatoid arthritis	2.48E-04			24	ADM, CCL5, CCR1, CD86, CDA, F11R, FPR2, HDAC7, HLA-G, HNMT, HNRNPA1, HSPA1A/HSPA1B, IL1B, MCL1, NUMB, P2RY13, PECAM1, PTGS2, PTMA, PTPRE, RALB, S100A9, SORL1, TNFRSF10C
Infectious Diseases	Infection by Murine leukemia virus	2.55E-04		0.277	6	APOBEC3B, APP, MDM2, MERTK, TLR4, TRIM5
Cellular Movement, Hematological System Development and Function, Immune Cell Trafficking, Inflammatory Response	Chemotaxis of phagocytes	2.56E-04	Decreased	-4.261	66	AIF1, APOA1, APP, AQP9, ARHGAP25, B4GALT1, CAMK1D, CCDC88A, CCL23, CCL5, CCR1, CSF1R, CSF3R, CX3CR1, CXCL1, CXCL6, CXCL9, CXCR2, CXCR3, CYBB, DEFB103A/DEFB103B, DOCK2, ELN, F2R, FCGR2A, FPR1, FPR2, GIT2, HCK, HEBP1, ICAM1, IL1B, ITGA4, JAML, LGMN, LILRB3, LITAF, LSP1, LYN, MPP1, MYO1F, NCKAP1L, NINJ1, PDE4B, PF4, PLCG2, PRKG1, PTEN, PTPN6, RAC2, RHOA, RHOB, RICTOR, RPL13A, S100A14, S100A9, SPHK2, SWAP70, SYK, TAFA4, THBS2, TLR2, TLR4, TNFRSF1A, TREM1, TREML2
Cellular Movement, Hematological System Development and Function, Immune Cell Trafficking, Inflammatory Response	Chemotaxis of granulocytes	2.57E-04	Decreased	-3.088	43	APOA1, APP, AQP9, ARHGAP25, CAMK1D, CCL23, CCL5, CSF3R, CXCL1, CXCL6, CXCL9, CXCR2, CXCR3, DEFB103A/DEFB103B, DOCK2, FCGR2A, FPR1, FPR2, GIT2, HCK, ICAM1, IL1B, ITGA4, JAML, LILRB3, LSP1, LYN, MPP1, MYO1F, NCKAP1L, PDE4B, PF4, PRKG1, PTEN, PTPN6, RAC2, S100A14, S100A9, SYK, TLR4, TNFRSF1A, TREM1, TREML2

Cancer, Hematological Disease, Organismal Injury and Abnormalities	Polycythemia vera	2.58E-04			16	APC, ARNTL, ASXL1, CUX1, HBA1/HBA2, IFNAR1, JAK1, KRAS, let-7, mir-26, NR3C1, PTGS2, SF3B1, SH2B3, TET2, U2AF1/U2AF1L5
Cardiovascular System Development and Function, Cellular Development, Cellular Function and Maintenance, Cellular Growth and Proliferation, Organismal Development, Tissue Development	Cell proliferation of vascular endothelial cells	2.61E-04	Decreased	-3.666	44	ADAM15, ADAM17, ADM, ANTXR2, C1GALT1C1, CAVIN2, COL1A2, CXCL1, CYBB, DAB2, F2R, FOXO3, FPR2, FRS2, HSPA5, IGF1, IL1B, IL1RN, ITGA4, let-7, MEF2C, MERTK, mir-133, mir-154, mir-24, MTOR, MYOF, OXT, PAQR3, PECAM1, PLXNA4, PRL, PTEN, S100A9, SKP2, SLC8A1, SP1, STAT3, TAZ, THBS2, THRAP3, TNFSF10, WNK1, WWTR1
Post-Translational Modification, Protein Degradation	Oxidation of protein	2.65E-04		1.088	10	ALDH3A1, APP, CYBB, FTH1, FTL, HMOX1, IGF1, NFE2L2, PARK7, RHOA
Cell Death and Survival	Cell death of myeloma cell lines	2.77E-04		0.601	26	ARNT, B2M, BCL2L11, BTK, CASP8, CXCR3, DDIT3, FOXO3, IGF1, IGF1R, IL1B, IRF8, KRAS, MCL1, MDM2, mir-154, MTOR, NR3C1, PRKCD, PTEN, SP1, STAT3, STK4, TNFSF10, VDAC1, YBX1
Connective Tissue Disorders, Inflammatory Disease, Inflammatory Response, Organismal Injury and Abnormalities, Skeletal and Muscular Disorders	Inflammation of joint	2.79E-04		0.104	203	ABCC2, ACO1, ACSL1, ADAM10, ADAM15, ADAM17, ADGRA1, ADIPOR1, ADM, AIF1, ALOX5AP, APLP2, APOA1, AQP9, ARF1, ARHGDIB, ATAT1, B2M, BCL2L11, BGN, BID, C9orf78, CARD8, CASC3, CASP8, CCL23, CCL5, CCR1, CD86, CDA, CELF2, CLEC1B, CLEC4D, CLIC2, CNR1, CRY2, CSF1R, CSF3R, CTLA4, CTSB, CTSC, CX3CR1, CXCL1, CXCL16, CXCL6, CXCL9, CXCR2, CXCR3, CYP4F3, DDIT3, DEF6, DNAJA4, DYNLL1, ECHDC1, EEF1E1, EIF1B, ELF3, EPO, F10, F11R, F13A1, FCGR2A, FGL2, FKBP5, FOXO3, FOXP3, FPR2, FTH1, FTL, GALNT1, GLIPR2, GLUL, H3-3A/H3-3B, HAMP, HBA1/HBA2, HBB, HCK, HCLS1, HDAC7, HLA-A, HLA-C, HLA-E, HLA-G, HMOX1, HNMT, HNRNPA1, HSP90B1, HSPA1A/HSPA1B, HSPA5, HSPD1, ICAM1, IFNAR1, IFNGR1, IGF1, IGFBP4, IL1B, IL1R2, IL1RN, JAK1, JMJD1C, KCTD20, KRAS, LCP1, LINC00922, LTBR, LYN, LYZ, MACIR, MAP3K2, MAP4K4, MAPRE1, MCL1, MDM2, MEFV, MERTK, MMP14, MRFAP1, MS4A1, MS4A7, MTOR, NAMPT, NOM1, NONO, NR3C1, NTRK1, NUMB, NUP62, OXT, P2RY13, PDE4B, PDGFRA, PDIA3, PECAM1, PF4, PHTF1, PILRA, PLAC4, PLCG2, PSMB8, PTEN, PTGS2, PTMA, PTPN6, PTPRE, PURA, RALB, RAMP2, RBPJ, RFX3, RGCC, RIPK2, RNF149, RNF169, RPL18A, RTF2, S100A9, SCN9A, SEC14L3, SEL1L, SF3B6, SLC11A1, SLC22A4, SOD2, SORL1, SPHK2, SPOCK1, STAT3, STEAP4, STK19, SWT1, SYK, TALDO1, TCF4, TFRC, TJP1, TLR2, TLR4, TLR5, TLR7, TMEM178A, TNFRSF10C, TNFRSF10D, TNFRSF1A, TNFSF10, TNFSF4, TNNC1, TRIO, TUBA1A, TUBA1C, TUBB2A, TUT7, TYROBP, UQCRC2, USP15, VDR, VIM, VTCN1, WIPF1, WNK1, ZNF143, ZNF281, ZNF326, ZNF331
Infectious Diseases	Susceptibility to tuberculosis	2.84E-04			5	IFNGR1, MAPKAPK3, SLC11A1, SP110, TLR2
Cellular Movement, Hematological System Development and Function, Immune Cell Trafficking	Cell movement of PBMCs	2.84E-04	Decreased	-3.364	20	ADAM17, ANXA2, APOA1, APP, CCL5, CCR1, CXCL16, CXCL9, CXCR3, F2R, FPR1, FPR2, FYB1, ICAM1, LCP1, LCP2, NR3C1, PECAM1, PLCB3, TLR2
Inflammatory Disease	Chronic inflammatory disorder	2.86E-04		1.659	232	ACO1, ACSL1, ADAM10, ADAM15, ADAM17, ADGRA1, ADIPOR1, ADM, AIF1, APC, APLP2, APOA1, APOA2, APP, AQP9, ARF1, ARHGDIB, ATAT1, B2M, BCAS3, BCL2L11, BGN, C9orf78, CA4, CARD8, CASC3, CCDC40, CCL23, CCL5, CCR1, CCR10, CD86, CDA, CDKL2, CELF2, CLEC1B, CLEC4D, CLIC2, COL1A2, CSF3R, CSTF1, CTLA4, CTNND1, CTSB, CTSC, CX3CR1, CXCL1, CXCL16, CXCL6, CXCL9, CXCR2, CXCR3, CYBB, CYP4F3, CYP51A1, DDIT3, DDX5, DEF6, DNAJA4, DYNLL1, ECHDC1, EEF1E1, EIF1B, ENTPD1, EPO, F10, F11R, FCGR2A, FCGR2C, FGL2, FKBP5, FOXL2, FOXO3, FOXP3, FPR2, FRMD4B, FTH1, FUT7, GALNT1, GC, GLIPR2, GLUL, GTPBP1, H3-3A/H3-3B, HAMP, HBA1/HBA2, HCK, HCLS1, HDAC7, HLA-A, HLA-C, HLA-G, HMOX1, HNMT, HNRNPA1, HSP90B1, HSPA1A/HSPA1B, HSPA5, HSPD1, ICAM1, IFI16, IFNAR1, IFNGR1, IFNGR2, IGF1, IGF1R, IGFBP4, IL1B, IL1R2, IL1RN, ITGA4, ITGB8, JAK1, JMJD1C, KCTD20, KIAA0040, KRAS, LCP1, let-7, LINC00922, LRP2, LRP8, LTBR, LY86, LYZ, MACIR, MAP3K2, MAP4K4, MAPKAPK3, MAPRE1, MCL1, MDM2, MEFV, MERTK, mir-138, mir-422, mir-515, mir-550, MMP14, MPEG1, MRFAP1, MS4A1, MS4A7, MTOR, MUC1, NAMPT, NCF2, NOM1, NONO, NOTCH2, NR3C1, NTRK1, NUMB, OXT, P2RY13, PBLD, PDE4B, PDE8A, PDGFRA, PDIA3, PECAM1,

						PHTF1, PLAC4, PRKCD, PSMB8, PSMD1, PSMD2, PTGFRN, PTGS2, PTMA, PTPN6, PTPRE, RALB, RAMP2, RFX3, RGCC, RHOA, RNF149, RNF169, RPL18A, RSPH3, RTF2, S100A9, SEC14L3, SEL1L, SF3B1, SF3B6, SLC22A4, SLC22A5, SLC24A4, SORL1, SPOCK1, SRD5A2, STAT3, STEAP4, STK19, SWT1, SYK, TAFA4, TALDO1, TCF4, TET2, TFRC, TJP1, TLR2, TLR4, TLR5, TLR7, TNFRSF10C, TNFRSF10D, TNFRSF1A, TNFSF10, TNFSF4, TNNC1, TRABD2B, TREM1, TRIO, TUT7, UQCRC2, USP15, USP4, VDR, VIM, VNN2, VTCN1, WDFY3, WNK1, ZBTB46, ZNF143, ZNF281, ZNF326, ZNF331
Cell Death and Survival	Apoptosis of breast cell lines	2.91E-04		0.539	21	ATG7, BCL2L11, BID, BTK, CASP8, CFLAR, CLCA2, DAB2, DYNLL1, FOXO3, IGF1, IGF1R, KRAS, LIMS1, MTOR, PRL, SGK1, TAZ, TNFSF10, WWTR1, YWHAZ
Cellular Development, Cellular Growth and Proliferation	Proliferation of myeloma cell lines	2.91E-04		-1.346	21	DDIT3, EEF2K, EPO, FBXL15, FOXO3, HCK, IGF1, LYN, MDM2, mir-154, MTOR, PTGS2, RAB8A, RAF1, SGK1, SLC10A5, SOD2, SP1, TIFA, TNFSF10, YBX1
Cell Death and Survival	Cell death of central nervous system cells	2.97E-04		-0.163	66	APP, ATG7, ATXN3, BCL2L11, BECN1, BID, CAMK2A, CASP8, CDC25C, CFLAR, CXCL1, DDIT3, EPO, F2R, FAIM2, FOXO3, FUS, GAPDH, GCLC, HDAC9, HSP90AA1, HSPA5, HSPD1, IGF1, IGF1R, IL1B, IL1RN, KLF6, LILRB3, LRPAP1, MAP3K1, MCL1, MEF2C, mir-26, MTOR, NFATC4, NFE2L2, NTRK1, PARK7, PITX2, PLXNA4, PRKAA1, PRKCD, PRKCG, PTEN, PTGS2, RHOA, RIT1, SEL1L, SGK1, SHC3, SKP2, SP1, SP3, SRPK2, STIP1, STK4, TCP1, TLR2, TLR4, TLR7, TNFRSF1A, TNFSF10, UBE2L3, WNK3, YWHAB
Organismal Survival	Morbidity or mortality	2.97E-04	Increased	13.683	479	ACTG1, ADAM10, ADAM15, ADAM17, ADM, AGO2, AKAP12, ALDH5A1, ALKBH3, AMPH, ANGPTL4, ANTXR2, ANXA2, AP1G1, APBA1, APC, APLP2, APOA1, APOB, APP, ARF1, ARF4, ARHGDIB, ARID4B, ARIH2, ARNT, ARNT2, ARNTL, ARPP19, ASAH1, ASXL1, ATG3, ATG7, ATN1, ATOX1, ATP7B, ATXN3, AURKB, B2M, B4GALT1, BBS7, BCAS3, BCL2L11, BCL7A, BECN1, BEST1, BGN, BID, BNIP3L, BRCA1, BRIP1, C1GALT1C1, C7orf25, CA4, CAP1, CAPZB, CASP8, CBY1, CCDC47, CCNK, CCP110, CCR1, CCR10, CD14, CDA, CDK2AP1, CELF1, CELF2, CELSR3, CFLAR, CHD4, CHM, CHMP2A, CKS2, CLCN3, CLEC1B, CLEC4D, CLEC4M, CLEC6A, CLEC7A, CLIC4, CNP, CNPY3, CNR1, COL7A1, CPLX2, CPT1A, CREB1, CRKL, CSAD, CSF1R, CTLA4, CTNND1, CTSB, CUX1, CX3CR1, CXCL6, CXCL9, CXCR2, CXCR3, CYBB, CYP24A1, CYP26B1, CYP51A1, DAB2, DCLRE1C, DDIT3, DDX17, DDX3X, DDX5, DGAT2, DHCR7, DLD, DMTF1, DNAJB4, DNAJB6, DNM3, DOCK2, DPH3, DPP10-AS1, DPYD, DYRK1A, ECE1, EEF1E1, EEF2K, EHD3, ELK3, ELN, ELOA, EPB41L3, EPO, ERCC5, ERO1A, ETV6, EVC2, EWSR1, EXT1, EXTL3, F10, F13A1, F2R, F8, FAH, FOXL2, FOXO3, FOXP3, FPR1, FPR2, FRS2, FTH1, FTL, FTX, FUS, GAB1, GALNT1, GATA5, GATAD2A, GBE1, GCLC, GLCE, GLT8D2, GNG7, GRB2, GSE1, H2AC18/H2AC19, H3-3A/H3-3B, HAMP, HCK, HDAC7, HFE, HLA-A, HLA-G, HMOX1, HNRNPA1, HOTAIR, HOXA3, HOXA4, HOXA7, HSBP1, HSD17B12, HSP90AA1, HSP90AB1, HSP90B1, HSPA5, HSPB7, ICAM1, IFNAR1, IFNGR1, IGF1, IGF1R, IGF2BP3, IGF2R, IL1B, IL1RN, ILF3, IP6K2, IPMK, IREB2, IRF8, ITGA4, ITGB8, JAK1, JPH4, JPX, KAT6A, KCNAB2, KCNJ2, KDM5A, KIDINS220, KIF1A, KLF6, KLF7, KMT5B, KRAS, L3MBTL2, L3MBTL3, LAT2, LCP1, LCP2, LEFTY1, LGR5, LIAS, LIMS1, LIN7A, LMTK2, LRP2, LRP8, LRPAP1, LTBR, LUCAT1, LY6K, LYN, LYZ, M6PR, MAFF, MAN2A2, MAP3K1, MAP4, MAP4K4, MAPKAPK3, MAX, MCL1, MCM3, MDM2, MED23, MEF2C, MERTK, MEX3B, mir-122, mir-133, mir-137, mir-154, mir-202, mir-26, mir-299, MLKL, MMP14, MORF4L1, MSH6, MSN, MTDH, MTF1, MTHFD2, MTOR, MTTP, MUC1, MYBBP1A, MYCNOS, MYH14, MYOF, MYOG, NAMPT, NAPB, NASP, NCOA1, NDC80, NDEL1, NFATC4, NFE2L2, NFKBIZ, NIN, NINJ1, NLGN3, NOTCH2, NR3C1, NTRK1, NUAK2, NUBP1, NUMB, NUP62, OAT, OPA1, OXT, P2RX1, PAK2, PCK1, PCLO, PCYT1A, PDCD4, PDE4B, PDGFRA, PDIA3, PDS5B, PELI2, PER2, PHF12, PIGA, PILRA, PILRB, PIP5K1A, PITPNA, PITX2, PLAGL2, PLCB3, PLCG2, PLCL1, PLEKHA7, PLP1, PNN, PNO1, PPIF, PPM1D, PPP6C, PRKAR1A, PRKCD, PRKG1, PSAP, PSMC1, PSMC2, PSMD4, PTEN, PTF1A, PTGS2, PTPN6, PURA, RAB11A, RAB27A, RAB31, RAB5A, RAB8A, RAC2, RAD51C, RAD51D, RAD52, RAF1, RALB, RAMP2, RAP1A, RAPGEF2, RASSF2, RBMS1, RBPJ, RFX3, RGMA, RHOA, RICTOR, RIPK3, RNASET2, RPL4, RPL5, RPS6KA5, RTEL1, RTN4, RUFY3, S100A9, SAV1, SCARB2, SCN9A, SCRIB, SDHD, SEL1L, SERTAD1, SESTD1, SETDB1, SF3B1, SFRP4, SH2B3, SHC3, SIAH1, SKP2, SLC22A4, SLC22A5, SLC25A37, SLC31A1, SLC4A2, SLC8A1, SMAD1, SMARCC2, SMTN, SNAP23, SNAP91, SNAPIN, SNX13, SNX27, SOD2, SP1, SPINK5, SPOP, SPOUT1, SPRTN, SRGAP3, SRGN, SRSF1, SRSF3, SSBP2, ST8SIA4, STAMBP, STAT3, STEAP4, STIP1, STK35, STK4, SUDS3, SUFU, SUPT4H1, SUSD6, SYK, SYT5, TAF7, TASP1, TBK1, TBX5, TCF15, TCF4, TDGF1, TET2, TFRC, THAP1, THBS2, THOC5, TIFA, TLR2, TLR4, TLR5, TLR7, TMEM107, TMOD3, TNFRSF1A, TNFSF10, TNIP1, TPM3, TRAF3, TREM1, TRIM55, TRIO, TRIP12, TRPM6, TSG101, TSHZ3, TXNRD1, U2AF1/U2AF1L5, UBA3, UBE2B, UBE2L3, UBE4B, UBR2, USP17L2 (includes others), USP4, VCAN, VDAC1, VDR, VIM, VPS41, VTCN1, VTI1A, VTI1B, WASF2, WIPF1, XRCC5, YBX1, YBX3, YWHAE, ZDHHC16, ZEB2, ZFPM2, ZMPSTE24, ZNF148, ZNF24, ZNF281
Cell-To-Cell Signaling and Interaction, Cellular Movement, Hematological System Development and Function, Immune	Recruitment of phagocytes	3.01E-04	Decreased	-3.149	57	ADAM10, ADAM17, ALOX5AP, APOA1, APOB, APP, ATG7, B4GALT1, CASP8, CCL23, CCL5, CCR1, CD14, CNR1, CSF1R, CTSC, CX3CR1, CXCL1, CXCL6, CXCR2, F13A1, FCGR2A, FPR2, FUT7, GAB2, GC, HCK, HSPA1A/HSPA1B, ICAM1, IFNAR1, IL1B, IL1RN, KRAS, LSP1, LYN, LYZ, MGAT5, NFE2L2, P2RX1, PDE4B, PECAM1, PTEN, RHOA, RHOB, RIPK2, RTN4, SIGLEC9, SOD2, ST3GAL6, STAT3, THBS2, TLR2, TLR4, TLR5, TNFRSF1A, TREML2, VDR

Cell Trafficking, Inflammatory Response						
Cell-To-Cell Signaling and Interaction, Hematological System Development and Function, Immune Cell Trafficking	Binding of granulocytes	3.03E-04	Decreased	-2.409	34	ADAM10, ADAM17, ADGRE2, APOA1, B4GALT1, CCL5, CSF3R, CXCL1, CXCR2, CYBB, F10, FCGR2A, FPR2, FUT7, HCK, ICAM1, IL1B, ITGA4, ITGAX, LCP1, LGALS8, LILRB3, LSP1, LYN, MGAT5, PECAM1, PF4, PLCB3, PTPN6, S100A9, SWAP70, TLR2, TLR4, TLR5
Lymphoid Tissue Structure and Development, Tissue Morphology	Quantity of lymph follicle	3.05E-04	Decreased	-3.497	37	ADAM10, ADGRG3, APBB1IP, ARHGDIB, ARNTL, BCL2L11, BECN1, BTK, CASP8, CD84, DKK3, DOCK2, DOCK8, FCAMR, GALNT1, HVCN1, IFNAR1, IFNGR1, IRF8, KIDINS220, KRAS, LYN, MTOR, NEDD9, NOTCH2, PLCG2, PRKCD, PTEN, SH3BP2, STAT3, STK4, TET2, TLR2, TLR4, TRIP10, TYROBP, WIPF1
Cell-To-Cell Signaling and Interaction	Interaction of tumor cell lines	3.06E-04	Decreased	-4.17	91	ADAM10, ADAM15, ADAM17, AGO2, AKAP12, ANGPTL4, ANXA2, ANXA5, APP, B4GALT1, CASP8, CD14, CHST6, CLCA2, CLEC4M, CLEC7A, CTSB, CTSZ, CXCL9, CXCR2, CXCR3, CYBB, CYP2J2, DAB2, DKK3, DOCK8, DSE, ELN, EPHB1, EZR, F10, F11R, F2R, FCGR2A, FUT7, FYB1, GAL3ST1, GMFG, HCK, HLA-A, HOXA4, HSP90B1, HSPA5, ICAM1, IGF1, IGF1R, IL1B, IL1R2, IP6K2, ITGA4, ITGAX, LAMA5, LASP1, LCP2, LGALS8, LRP2, LTBR, MGAT5, mir-103, MMP14, MTOR, MUC1, MUC13, NCL, PAK2, PECAM1, PITX2, PRKAA1, PRL, PSMD4, PTPN6, RAB21, RAF1, RAMP2, RAP1A, RHOA, RICTOR, SERPINB3, SFRP4, SH2B3, SRGN, ST6GALNAC2, STAT3, TFRC, TJP1, TLR2, TLR4, TLR5, VCAN, WWTR1, ZEB2
Cardiovascular System Development and Function	Development of vasculature	3.13E-04	Decreased	-5.992	230	ACTG1, ADAM15, ADAM17, ADM, ADM2, AGO2, AIF1, ALOX5AP, ANGPTL4, ANTXR2, ANXA2, ANXA3, APC, APOA1, APOB, APP, ARID4B, ARNT, ARNTL, ATG7, B4GALT1, BACH1, BCAS3, BECN1, BRCA1, C1GALT1C1, CAMK2A, CARD6, CASP8, CAVIN2, CCDC88A, CCL5, CHM, CLEC1B, CLIC4, CNMD, CNR1, COL1A2, CREB1, CRKL, CSF1R, CTSB, CX3CR1, CXCL1, CXCL6, CXCL9, CXCR2, CXCR3, CYBB, CYP4F2, CYP51A1, DAB2, DCTN5, DDIT3, DDX3X, DHCR7, DPH3, DUSP3, ECE1, EHD3, ELK3, ELN, EMC10, EPHB1, EPO, ERO1A, ETV6, F11R, F2R, FBLN2, FFAR4, FOXO3, FPR2, FRS2, GAB1, GAB2, GATA5, GATAD2A, GLUL, HCK, HDAC7, HDAC9, HLA-G, HMOX1, HOXA3, HOXA7, HSP90AA1, HSPA5, HSPB7, HSPD1, HTATIP2, ICAM1, IDH3A, IFI16, IFNAR1, IFT88, IGF1, IGF1R, IGF2R, IGFBP4, IL1B, IL1RN, ITGA4, ITGAX, ITGB8, KAT6A, KCNJ2, KIDINS220, KLF6, KLF7, KLHL20, KRAS, LAMA5, LEFTY1, let-7, LGALS8, LRP2, LRP8, LRPAP1, LRRFIP1, LTBR, LYVE1, MDM2, MED23, MEF2C, MERTK, MGAT5, mir-103, mir-122, mir-133, mir-137, mir-154, mir-24, mir-26, MMP14, MTDH, MTOR, MYOF, NCF2, NCL, NCOA1, NFATC4, NFE2L2, NLK, NOTCH2, NR3C1, NTRK1, NUMB, OTULIN, OXT, PAQR3, PDGFRA, PEAK1, PECAM1, PER2, PF4, PITX2, PLXNA4, PRKAA1, PRKCD, PRKCG, PRKG1, PRL, PRLH, PROK2, PSAP, PTEN, PTGS2, PTPN6, RAB9A, RAC2, RAF1, RAMP2, RAP1A, RAPGEF2, RBPJ, RGCC, RGS2, RHOA, RHOB, RICTOR, RIPK3, RTN4, S100A9, SAT1, SAV1, SCARB2, SEMA4A, SIRPA, SKP2, SLC8A1, SNX13, SOS2, SP1, SP100, SPHK2, SPINK5, SRGN, SRPK1, SRPK2, STAT3, STK4, STX7, SUFU, SYK, TAZ, TCF4, TDGF1, THAP1, THBS2, THRAP3, TJP1, TLK2, TLR2, TLR4, TNFRSF1A, TNFSF10, TUBA1C, UBE4B, UBR2, VDR, VIM, WARS1, WASF2, WNK1, WWTR1, YWHAZ, ZBTB46, ZFPM2, ZNF24
Cell-To-Cell Signaling and Interaction, Inflammatory Response	Immune response of leukocyte cell lines	3.14E-04	Decreased	-3.113	13	APOA1, APOA2, APP, BECN1, CD14, DDX3X, FCGR2A, PTPN6, SNAP23, SYK, TLR2, TLR4, TLR7
Cancer, Organismal Injury and Abnormalities	Multiple cancers	3.16E-04		0.106	640	ABCC2, ACBD3, ACP3, ACTG1, ACTR2, ACYP1, ADAM10, ADAM15, ADAM17, ADGRA1, AGO2, AIF1, AIG1, AIPL1, AK9, AKAP12, ALDH3A1, ALDH5A1, ALKBH1, ALKBH3, ALS2, AMPH, ANAPC13, ANGPTL5, ANKRD42, ANXA3, ANXA5, AOPEP, AP5M1, APBA1, APC, APOA1, APOB, APOBEC3B, APP, ARF4, ARHGAP19, ARHGDIB, ARMC3, ARMCX5-GPRASP2/GPRASP2, ARNT, ARNTL, ASB10, ASXL1, ATG2B, ATG7, ATL3, ATN1, ATP6V1B2, ATRN, AURKB, B2M, BCL2L11, BECN1, BGN, BLVRA, BMP2K, BNIPL, BOD1L1, BRCA1, BRIP1, BTG2, C17orf80, C18orf25, C1GALT1C1, C1RL, C7orf25, CACNA1E, CALCOCO2, CAMK2A, CAPZB, CARD16, CARNS1, CASP8, CATSPERD, CCDC47, CCDC88A, CCL5, CCP110, CD300E, CDC5L, CDH12, CELSR3, CEP128, CEP72, CFLAR, CHCHD5, CHD4, CKMT2, CLASP1, CLIC4, CLK2, CNPY3, COG2, COG5, COL1A2, COL7A1, CPEB1, CPQ, CPT1A, CRKL, CRY2, CRYBG3, CSDE1, CSF1R, CSF3R, CT45A10/CT45A5, CTAG2, CTBS, CTLA4, CTNND1, CTNND2, CTSB, CTSC, CTSZ, CUX1, CXCL1, CXCL16, CXCL9, CXCR2, CXCR3, CYBB, CYP24A1, CYP2A6 (includes others), CYP4F3, CYTH4, DAB2, DDX17, DDX23, DDX27, DDX39A, DDX3X, DDX5, DEF6, DENND3, DGLUCY, DHCR7, DHX8, DIP2B, DLGAP4, DNAJB12, DNAJB6, DNAJC2, DNAJC7, DNM3, DOCK8, DOK5, DPF3, DPH2, DPYD, DSE, DUSP5, DYRK1A, EBLN2, ECE1, EFS, EIF1AX, EIF3A, EIF4G3, ELF3, ELOA, EOGT, EPB41L3, EPHA8, EPM2AIP1, ERO1A, ETV6, EVI5L, EXOC3L4, EXT1, EZR, F10, F11R, F13A1, F2R, F8, FAM126B, FAM209A, FAM214B, FBLN2, FBXO38, FCAMR, FEZ1, FKBP5, FNBP1L, FOXL2, FOXO3, FOXP3, FPR2, FRMD4B, FTH1, FUBP3, FUS, FUT7, FZD1, FZD3, G3BP2, GAB2, GAL3ST1, GAPDH, GAS7, GASK1B,

						GATA5, GBE1, GC, GLE1, GLUL, GLYR1, GNB4, GOLGB1, GPATCH4, GPD1, GPR75, GSTA1, GTF3C3, GYG1, H2AC18/H2AC19, H2BC21, H3-3A/H3-3B, HAMP, HBA1/HBA2, HBB, HBP1, HCCS, HCK, HCLS1, HDAC9, HLA-A, HLA-E, HLA-G, HMOX1, HNRNPA1, HNRNPA2B1, HNRNPH2, HOTAIR, HOXA3, HOXA4, HSD17B12, HSP90AA1, HSP90AB1, HSP90B1, HSPA5, HSPB7, HSPD1, HTATIP2, HTR1F, HVCN1, IDH3A, IER2, IFNAR1, IFNGR1, IFNGR2, IGF1, IGF1R, IGF2BP3, IGF2R, IGFBP4, IGSF6, IL1B, IL1RN, ILF3, IQSEC3, IRX4, ITGAX, JAK1, JAML, JMJD1C, JMJD4, JPH4, JPT1, JPX, KAT6A, KAT6B, KCNJ4, KDM1B, KDM5A, KDM7A, KIF1C, KIF26B, KLF6, KLHL15, KRAS, KRT23, L3MBTL3, LAMA5, LAMTOR5, LARP4, LARP6, LAS1L, LASP1, LAT2, LCP2, LEFTY1, let-7, LETM2, LGALS8, LGR5, LHCGR, LILRA1, LIMK2, LINC00511, LIPM, LMTK2, LONRF3, LRP2, LRP8, LRRFIP1, LSP1, LY6K, LY86, LY9, LYVE1, MAFF, MAGT1, MAN2A2, MAP3K1, MAP4, MAP4K4, MAPRE1, MAPRE3, MARF1, MAX, MCCC2, MCL1, MDM2, MED23, MEF2C, mir-101, mir-103, mir-122, mir-154, mir-202, mir-24, mir-26, mir-28, MMP14, MORN5, MRPL15, MS4A1, MS4A14, MS4A4A, MS4A7, MSH6, MT1A, MT1F, MT1X, MTCH2, MTDH, MTFR2, MTHFD2, MTOR, MTTP, MUC1, MUC15, MYBBP1A, MYH15, MYLK, MYO5B, MYOF, MYOG, NABP1, NAMPT, NASP, NCF2, NCKAP1L, NCL, NDC80, NDE1, NFATC4, NFE2L2, NHSL1, NIN, NLGN3, NLGN4X, NONO, NOTCH2, NOXRED1, NPC1L1, NR3C1, NTRK1, NUMB, NUP50, NUP93, NXPE4, OPA1, OR2A14, OR4D10, OR5AC2, OSBPL11, OTUD3, PAK2, PCLO, PCOLCE, PDAP1, PDGFRA, PDIA3, PDLIM5, PDS5B, PEAK1, PECAM1, PEX19, PF4, PHF12, PILRA, PIN4, PIP5K1A, PITX2, PIWIL1, PLAGL2, PLB1, PLEKHA7, PLXDC2, PLXNA4, PODNL1, POTEH (includes others), PPM1D, PPP1R12B, PPP1R17, PPP4R2, PQBP1, PRC1, PRKAA1, PRKCD, PRKCG, PRL, PRPF6, PRR12, PRSS55, PSMB8, PSMD1, PSMD12, PSMD2, PSMD4, PSMD7, PTEN, PTGS2, PTPRE, PUDP, PWWP3A, RAB31, RAB3GAP2, RABGAP1L, RAD51C, RAD51D, RAF1, RALBP1, RALGPS1, RAP1A, RASEF, RBMXL3, RCBTB2, RFPL2, RFX3, RGCC, RGS2, RHBG, RHOA, RHOB, RICTOR, RIN2, RIOK1, RIOK2, RNF103, RNF103-CHMP3, RNF121, RNF130, RNF149, RPF2, RPGRIP1, RPL4, RPL5, RTCB, RTN3, RTN4, RTTN, S100A14, S100A9, SBF2, SCRIB, SCRT2, SEC14L1, SEC61A2, SENP2, SETDB1, SF3B1, SH2B3, SH3BP2, SIPA1L2, SIRPB1, SKP2, SLAMF7, SLC22A18, SLC22A4, SLC24A4, SLC25A32, SLC31A1, SLC35F4, SLC43A3, SLC4A2, SLC6A6, SLC8A1, SLITRK6, SMARCA2, SMTN, SNX27, SOD2, SORL1, SP100, SP3, SPAG9, SPATA5, SPEF2, SPHK2, SPOP, SRPK1, SRPK2, SSH3, STAT3, STEAP4, STX3, SUFU, SUSD6, SWAP70, SYK, SYNE4, SYT17, SZT2, TAF1, TAF7, TAGLN2, TBC1D12, TBC1D8, TBC1D9, TBL1X, TBX5, TCAIM, TCF4, TCP1, TDGF1, TDRD1, TERF2IP, TET2, TFRC, THBS2, THEG, THRAP3, TLR4, TLR5, TM2D2, TM7SF3, TMEM140, TMEM43, TMEM70, TMTC2, TNFRSF1A, TNFSF10, TNNC1, TNNI3K, TOR1B, TPM3, TRAF3, TRIM46, TRIM5, TRIM65, TRIO, TRIP10, TRIP12, TRMT9B, TRPM6, TSG101, TSHZ3, TTI2, TUBA1A, TUBA1B, TUBA1C, TUBB2A, U2AF1/U2AF1L5, UBA3, UBAP2L, UBE2E3, UBE4B, USP15, USP19, USP32, UTP4, VCAN, VCPIP1, VDAC2, VDR, VIM, VTCN1, VTI1B, WASF2, WASF3, WDFY3, WDR19, WIPF1, WNK1, WNK3, WSB1, XPNPEP3, XRCC5, YBX1, YPEL5, YWHAZ, ZAN, ZBTB21, ZEB2, ZFPM2, ZMPSTE24, ZMYM3, ZNF10, ZNF143, ZNF165, ZNF217, ZNF229, ZNF235, ZNF24, ZNF281, ZNF3, ZNF33B, ZNF398, ZNF41, ZNF45, ZNF461, ZNF516, ZNF525, ZNF555, ZNF570, ZNF606, ZNF615, ZNF667, ZNF677, ZNF684, ZNF711, ZNF738, ZNF746, ZNFX1, ZRSR2, ZSCAN2
Cell Death and Survival, Hematological System Development and Function	Cell viability of myeloid cells	3.20E-04	Decreased	-3.329	26	ADGRE2, APP, BCL2L11, BTK, CFLAR, CSF1R, CX3CR1, EPO, FOXO3, ICAM1, IL1B, KIF1C, LAT2, LYN, MCL1, MGAT5, PF4, PTPN6, RAC2, RAF1, SOD2, STAT3, TLR4, TNFSF10, TYROBP, YWHAZ
Cell-To-Cell Signaling and Interaction, Nervous System Development and Function	Long-term potentiation of hippocampus	3.21E-04		0.202	29	APP, ARHGEF9, ATXN3, B2M, CAMK2A, CCDC88A, CNR1, CREB1, CYBB, IGF2R, IL1B, IL1RN, ITM2B, JPH4, KIDINS220, KRAS, LGMN, LILRB3, LRP8, LRPAP1, NLGN3, NPTN, PJA2, PRKAR1A, RTN4, ST8SIA4, STIP1, TCF4, TLR4
Cell-To-Cell Signaling and Interaction, Cellular Function and Maintenance, Hematological System Development and Function, Inflammatory Response	Phagocytosis by macrophages	3.24E-04	Decreased	-2.872	33	APP, ATG7, BECN1, BTK, CD14, CD93, CLEC4M, CLEC6A, CLIC4, CSF1R, DEF6, DOCK2, FCGR2A, GAB2, HCK, HMOX1, IL1B, let-7, MERTK, MEX3B, mir-24, NCKAP1L, PTEN, RAB11A, RGCC, S100A9, SH3BP2, SIRPA, SIRPB1, SLAMF7, TLR2, TLR4, TYROBP
Cellular Function and Maintenance,	Engulfment of red blood cells	3.38E-04		-1.11	24	ACTR2, ARPC2, CD93, DOCK2, FCGR2A, HCK, ICAM1, IL1B, JAK1, KAT6A, KCTD5, LYN, MERTK, NCKAP1L, PLEK, PRKCD, PTEN, RAC2, RIT1, SIRPA, SYK, UBE2L3, WASF2, ZNF217

Hematological System Development and Function						
Cellular Movement	Transmigration of myeloid cells	3.40E-04	Decreased	-2.408	20	ADAM15, ADAM17, CXCL1, CXCL9, CXCR2, FPR1, ICAM1, ITGA4, MMP14, MTOR, MYLK, MYO1F, NINJ1, PECAM1, PTPN6, RHOA, RTN4, SIRPA, TLR2, TNFRSF1A
Cell-To-Cell Signaling and Interaction	Binding of lymphoid cells	3.59E-04	Decreased	-3.577	39	APBB1IP, APOA1, BTK, CCL5, CCR1, CD86, CTLA4, CXCL9, CXCR3, DOCK2, DOCK8, EZR, FUT7, FYB1, ICAM1, IFNGR1, IL1B, ITGA4, JAK1, LCP2, LTBR, MAP3K2, MSN, NEDD9, NR3C1, PECAM1, PRL, PTPN6, RAC2, RAP1A, RHOA, RICTOR, STK4, SWAP70, SYK, TFRC, THBS2, TLR4, TNFSF14
Cell-To-Cell Signaling and Interaction	Adhesion of myeloid cells	3.59E-04	Decreased	-2.857	39	ADAM10, ADAM17, ADGRE2, APOA1, APP, BTK, CCL5, CNR1, CSF3R, CTSZ, CXCL1, CXCR2, CYBB, F10, F2R, FPR2, HCK, ICAM1, IL1B, ITGA4, ITGAX, LCP1, LGALS8, LILRB3, LYN, MGAT5, PAK2, PECAM1, PF4, PLCB3, PTGS2, PTPN6, RAC2, RHOB, S100A9, SWAP70, TLR2, TLR4, TLR5
Cellular Assembly and Organization, Cellular Function and Maintenance	Formation of multivesicular bodies	3.79E-04			10	ANXA2, CHMP2A, CHMP3, CHMP4B, CHMP6, LITAF, RAB11A, TSG101, VPS25, VPS4B
Cardiovascular Disease, Organismal Injury and Abnormalities	Formation of blood clot	3.79E-04		-0.905	18	ANXA2, APP, CXCR2, EPO, F10, F8, FCGR2A, LCP2, LRP8, P2RX1, PF4, PLCB3, RAP1A, RHOA, SGK1, SYK, TNFRSF1A, VDR
Post-Translational Modification	Phosphorylation of protein	3.88E-04	Decreased	-3.697	141	ADAM10, ADAM17, ADM, ADM2, AIF1, ANKLE2, ANXA2, APOA1, APP, ATG14, AURKB, BTBD10, BTK, CAMK1G, CAMK2A, CCDC88A, CCL5, CCNYL1, CCR1, CDK2AP1, CDKL5, CELSR3, CLEC1B, CLEC7A, CLK2, CLK3, CORO1C, CREB1, CRKL, CSF1R, CTLA4, DAB2, DYNLL1, DYRK1A, EEF2K, EIF4G3, ELF1, EPHA8, EPHB1, EPO, F2R, FCGR2A, FPR2, FZD1, GLYCTK, GMFG, GPRC5B, GRK7, HCK, HSP90AA1, HTATIP2, IGF1, IGF1R, IL1B, ILF3, JAK1, KCTD20, KRAS, LAT2, LILRB3, LIMK2, LMTK2, LYN, MAK, MAP3K1, MAP3K2, MAP4K4, MCM7, MERTK, mir-137, mir-26, MKNK1, MTOR, MYLK2, NCKAP1L, NIM1K, NLK, NPTN, NTRK1, NUAK2, OXT, PAK2, PAQR3, PARK7, PDE8A, PDGFRA, PDIA3, PEAK1, PECAM1, PELI2, PHACTR1, PHKB, PHLPP1, PID1, PKN3, PRKAA1, PRKAR1A, PRKCD, PRKCG, PRKG1, PRL, PSAP, PTEN, PTPN6, RAF1, RALB, RGMA, RICTOR, RIOK2, RIPK3, ROPN1L, RPS6KA5, SDCBP, SENP2, SGK1, SIRPA, SMAD1, SRPK1, SRPK2, STAT3, STK19, STK24, STK38L, STK4, SYK, TAF1, TBK1, TERF2IP, TESK2, TFRC, TLK2, TLR7, TNNI3K, TRAF3IP1, TYROBP, WARS1, WEE2, WNK1, WNK3, WWTR1, YWHAZ
Cellular Movement, Immune Cell Trafficking	Cell movement of lymphatic system cells	4.03E-04	Decreased	-4.302	93	ADAM10, ADAM17, ADGRG3, APBB1IP, APC, APP, ATG7, BGN, BTK, CCL23, CCL5, CCR1, CCR10, CD86, CLEC1B, CTLA4, CUX1, CX3CR1, CXCL1, CXCL16, CXCL6, CXCL9, CXCR2, CXCR3, CYP26B1, DEF6, DEFB103A/DEFB103B, DOCK2, DOCK8, DPYSL2, EFS, EPO, EZR, F11R, FOXP3, FRS2, FUT7, FYB1, HCLS1, HLA-A, HLA-G, HMOX1, HSPD1, ICAM1, IFNAR1, IFNGR1, IGF1, IL1B, ITGA4, JAK1, KCNE3, KRAS, LCP1, LCP2, LTBR, MAP3K2, MAPKAP1, MMP14, MSN, MTOR, MYLK, NEDD9, NR3C1, PECAM1, PF4, PLCB3, PLCG2, PRKAA1, PRKCD, PTEN, PTGS2, RAC2, RAP1A, RHOA, RICTOR, SCRIB, SERPINB3, SOS2, SPHK2, STAT3, STK4, SWAP70, SYK, THBS2, TLR2, TLR4, TNFRSF1A, TNFSF10, TNFSF14, TNFSF4, TNIP1, VTCN1, WIPF1
Cancer, Hematological Disease, Immunological Disease, Organismal Injury and Abnormalities	Non-Hodgkin lymphoma	4.03E-04		1.03	223	AMPH, ANKLE2, ANO5, ANXA2, APBA1, APOBEC3A, APOBEC3B, APP, ARHGAP17, ASB10, ASMTL, ASXL1, ATN1, ATP6V1B2, ATRN, ATXN3, AURKB, B2M, BASP1, BAZ2B, BBS7, BCL2L11, BCL7A, BECN1, BOD1L1, BRCA1, BRIP1, BTG2, BTK, CARMIL1, CARNS1, CASP8, CAV3, CCNDBP1, CD86, CDC23, CELF2, CFLAR, CGB1/CGB2, CHD4, CHPF, CMSS1, CNR1, COL1A2, CPSF7, CRNN, CSDE1, CSF1R, CSF3R, CXCL9, CXCR3, CYP2A6 (includes others), DCLRE1C, DDX3X, DMTF1, DNAJB14, DNM3, DOCK2, DPYD, DUSP5, DYRK1A, ETV6, EWSR1, F11R, FAM131C, FCAMR, FCGR2A, FOXP3, FUS, FYB1, FZD1, GPRIN1, GPSM2, GRB2, GSE1, HAO2, HCK, HCLS1, HDAC7, HDAC9, HECA, HLA-A, HLA-G, HMOX1, HNRNPA2B1, HOXA7, HSP90AA1, HSP90AB1, HSP90B1, HVCN1, ICAM1, IDH3A, IFNAR1, IGF1, IGFBP4, IL1B, ING3, IRF8, ITGAX, JAK1, JMJD1C, KAT6A, KLF6, KRAS, LCT, let-7, LRRFIP1, LSM3, LTBR, MAP4K4, MAX, MCL1, MDM2, MERTK, MICB, mir-101, mir-154, mir-26, mir-28, MPEG1, MS4A1, MTOR, MUC1, MYO5B, MYOF, NACA2, NDUFS1, NETO2, NINJ1, NONO, NOTCH2, NR3C1, NUAK2, NUBP1, NUDT6, NXPE4, PAK2, PCLO, PCOLCE, PDGFRA, PECAM1, PLCG2, PLEKHA7, POLR3B, POTEH (includes others), PPM1D, PPP1R12B, PPP6R3, PRKG1, PSMB8, PSMD1, PSMD2, PSME3, PTEN, PTGS2, PTPN6, PTPRE, PWWP3A, RAB38, RAB4A, RAD52, RAF1, RBM4, RBPJ, RESF1, RGCC, RHOA, RICTOR, RPS15, RTTN, SCN9A, SEC14L1, SERTAD1, SF3B1, SGK1, SHROOM3, SKP2, SMARCA2, SORL1, SP100, SP110, SRPK2, SSBP2, STAT3, STIP1, STXBP6, SWAP70, SYK, TAF1, TDRD1, TET2, THBS2, TJP1, TLR2, TLR4, TLR7, TNFRSF10C, TNFRSF10D, TNIP1, TRAF3, TRIM55, TRIP12, TRPM6, TTC21B, TUBA1A, TUBA1C, TUBB2A, U2AF1/U2AF1L5, UBE2F, UNC5C, VDAC1, WIPF1, XRCC5, YAE1, YWHAE, YWHAZ, ZMYM3, ZNF148, ZNF331, ZNF615, ZNF700, ZNF714, ZRSR2
Cell Death and Survival,	Cell death of brain cells	4.13E-04		-0.25	61	APP, ATG7, ATXN3, BCL2L11, BECN1, BID, CAMK2A, CASP8, CDC25C, CFLAR, CXCL1, DDIT3, EPO, FAIM2, FOXO3, FUS, GAPDH, GCLC, HDAC9, HSPA5, HSPD1, IGF1, IGF1R, IL1B, IL1RN, KLF6, LILRB3, LRPAP1, MAP3K1, MCL1, MEF2C, mir-26, MTOR, NFATC4, NFE2L2,

Neurological Disease, Organismal Injury and Abnormalities						NTRK1, PARK7, PITX2, PRKCD, PRKCG, PTEN, PTGS2, RHOA, RIT1, SGK1, SHC3, SKP2, SP1, SP3, SRPK2, STIP1, STK4, TCP1, TLR2, TLR4, TLR7, TNFRSF1A, TNFSF10, UBE2L3, WNK3, YWHAB
Cell Death and Survival	Apoptosis of tumor cell lines	4.34E-04	Increased	2.359	261	ACO2, ADAM17, ADIPOR1, ADM, AKAP12, ALKBH3, ALS2, ANGPTL4, ANXA2, ANXA5, APC, APP, ARNT, ATF5, ATG7, ATN1, B2M, BACH1, BCL2L11, BECN1, BID, BNIP2, BNIP3L, BRCA1, BTG2, BTK, CARD8, CASP8, CCT2, CD14, CELF1, CFLAR, CIBAR1, CKS2, CLASP1, CLCA2, CLK3, CNR1, CREB1, CSF1R, CTNND1, CTSB, CUX1, CXCR3, CYP2J2, DAB2, DDIT3, DFFA, DKK2, DKK3, DPH2, DTD2, DYNLL1, EEF2K, EPO, EWSR1, EZR, FAIM2, FASTKD2, FFAR4, FKBP5, FOXL2, FOXO3, FOXP3, FTH1, GAB1, GAPDH, GAS7, GIMAP4, GLIPR1, GSTA1, GUCA2A, GUCA2B, HCK, HCLS1, HDAC9, HFE, HLA-G, HMOX1, HNRNPA1, HNRNPH1, HOTAIR, HSP90AB1, HSPA1A/HSPA1B, HSPA5, HSPD1, HTATIP2, IFI16, IGF1, IGF1R, IGF2R, IGFBP4, IL1B, ING3, IP6K2, IRF8, ITGA4, JAK1, KIDINS220, KIF1C, KLF6, KRAS, LAMA5, let-7, LGALS8, LGR5, LIMS1, LINC00887, LSP1, LUCAT1, LYN, LYPLA2, MAP3K1, MAPKAP1, MAX, MCL1, MDM2, MEF2C, MERTK, mir-103, mir-122, mir-133, mir-138, mir-154, mir-26, mir-299, mir-515, MIR4728, MKNK1, MMP14, MOB3A, MS4A1, MSN, MT1F, MTDH, MTOR, MUC1, MVP, MXD1, MYBBP1A, NASP, NBR2, NCL, NCOA4, NDC80, NEDD9, NFE2L2, NFKBIZ, NOTCH2, NR3C1, NTRK1, NUMB, OPA1, PAK2, PARK7, PDCD4, PDE4B, PDGFRA, PECAM1, PHLPP1, PIWIL1, PLAGL2, PLCG2, PLXNA4, PPM1D, PRKAA1, PRKAR1A, PRKCD, PRKG1, PRL, PSAP, PSMD4, PSME3, PTEN, PTGS2, PTMA, PTPN6, PTPRE, PUS10, RAF1, RALB, RAPGEF2, RASD1, RASSF3, RBM5, RHOA, RHOB, RICTOR, RIPK2, RIPK3, RIT1, RTN1, RTN4, S100A9, SAT1, SENP8, SFRP4, SGK1, SH3RF1, SIAH1, SIRPA, SKP2, SLU7, SMAD1, SOD2, SP1, SPHK2, SPOCK1, SPOP, SRGN, SRPK1, SRPK2, SRSF1, STAT3, STAU1, STK4, SUDS3, SYK, TACC1, TAGLN2, TASP1, TBK1, TCF4, TCP1, TDGF1, TDP2, TERF2IP, TESK2, TFRC, THAP1, THOC5, TLR2, TLR4, TM9SF4, TMBIM6, TNFRSF10C, TNFRSF10D, TNFRSF1A, TNFSF10, TNFSF14, TRAF3, TREM1, TSG101, TTF1, TUBA1A, TXNRD1, UBE2V1, USP17L2 (includes others), VCAN, VDAC1, VDAC2, VDR, VPS35, WSB1, WWTR1, XAF1, XRCC5, YBX1, YWHAE, YWHAZ, ZNF148
Neurological Disease, Organismal Injury and Abnormalities	Progressive encephalopathy	4.39E-04		1.405	179	ADAM10, ALDH5A1, ALS2, AMPH, ANXA2, ANXA5, APLP2, APOA1, APOA2, APP, ARHGDIB, ARL6IP5, ARMC2, ARNT, ARNT2, ASAH1, BCL2L11, BECN1, BGN, BRCA1, CAMK2A, CAPZB, CASP8, CCDC88A, CCL5, CCT2, CD14, CDCP2, CELF2, CNP, CNR1, COL1A2, CPT1A, CSF1R, CSF3R, CTSB, CXCL16, CXCR2, CYP26B1, DDC, DDIT3, DHCR7, DNAJB6, DOK5, DPYSL2, DYRK1A, EEF2K, ELN, ENO3, EZR, F2R, FCGR2A, FCGR2C, FOXO3, FRMD4B, FTH1, FTL, FUS, GAB2, GALC, GAPDH, GAS7, GC, GCNT2, H3-3A/H3-3B, HBA1/HBA2, HFE, HMOX1, HNRNPA1, HNRNPA2B1, HSPA1A/HSPA1B, HSPA5, HSPD1, ICAM1, IFNGR1, IGF1, IGF1R, IL1B, IL1R2, IREB2, JPT1, KIAA0040, KIF1A, KRAS, LARP4, let-7, LGMN, LIMS1, LOC440040, LRP8, LRPAP1, MBP, MEF2C, mir-101, mir-103, mir-133, mir-154, mir-24, mir-26, mir-28, mir-3180, mir-3690, mir-422, mir-515, mir-550, mir-551, mir-657, MIR4270, MS4A4A, MS4A6E, MTHFD2, MTOR, MTRR, MYOG, NFATC4, NFE2L2, NFS1, NOM1, NR3C1, NTRK1, OPA1, PARK7, PDE4B, PDIA3, PGAM2, PIP4P2, PLA2G4C, PLCG2, PRKAR1A, PRKCD, PRL, PSAP, PSMC1, PTEN, PTGS2, PTPRE, RHOA, RHOB, RNASET2, RNF114, RNF6, RPL13A, RTN1, RTN3, RTN4, S100A9, SCARB2, SCN9A, SGK1, SHROOM3, SLC52A2, SLC6A6, SNAP91, SOD2, SORL1, SRPK2, ST8SIA4, STAT3, STIP1, TAF1, TBK1, TFRC, TLR4, TNNC1, TRIO, TSHZ3, TUBA1A, TUBA1B, TUBA1C, TUBB2A, TYROBP, VDAC1, VDR, VIM, VPS35, WDFY3, WWTR1, YWHAZ, ZNF721
Cell-To-Cell Signaling and Interaction, Nervous System Development and Function	Long-term potentiation of cerebral cortex	4.44E-04		0.2	30	APP, ARHGEF9, ATXN3, B2M, CAMK2A, CCDC88A, CNR1, CREB1, CYBB, IGF2R, IL1B, IL1RN, ITM2B, JPH4, KIDINS220, KRAS, LGMN, LILRB3, LRP8, LRPAP1, NLGN3, NPTN, PJA2, PRKAR1A, RHOB, RTN4, ST8SIA4, STIP1, TCF4, TLR4
Cancer, Gastrointestinal Disease, Organismal Injury and Abnormalities	Small intestine carcinoma	4.46E-04			15	APC, CTLA4, FCRLA, GSE1, HSP90AA1, HSP90AB1, HSP90B1, KBTBD12, KRAS, MSH6, NTRK1, PRKCD, PRKCG, SSBP2, U2AF1/U2AF1L5
Cellular Movement	Transmigration of cells	4.57E-04	Decreased	-4.248	42	ADAM10, ADAM15, ADAM17, APP, ARHGAP25, CCL5, CCR1, CD86, CX3CR1, CXCL1, CXCL9, CXCR2, CXCR3, DOCK8, ELN, F11R, F2R, FPR1, GALNT1, HSP90AA1, ICAM1, IGF1, IL1B, ITGA4, ITGAX, LSP1, MMP14, MTOR, MYLK, MYO1F, NINJ1, PECAM1, PTPN6, RAC2, RAP1A, RHOA, RTN4, SIRPA, TLR2, TNFRSF1A, TRIM55, TRIO
Cellular Movement, Hematological System Development and Function, Immune Cell Trafficking	Homing of mononuclear leukocytes	4.61E-04	Decreased	-3.46	47	ADAM10, ADAM17, AIF1, APOA1, APP, CCL23, CCL5, CCR1, CUX1, CXCL16, CXCL6, CXCL9, CXCR2, CXCR3, DEFB103A/DEFB103B, ELN, F2R, FOXP3, FPR1, FPR2, FUT7, FYB1, HEBP1, HLA-G, HSPD1, IL1B, ITGA4, JAK1, LCP1, LGMN, LTBR, MAPKAP1, MYLK, NEDD9, NR3C1, PF4, PTEN, RAC2, RHOA, S100A14, STAT3, STK4, THBS2, TLR2, TLR4, TNFSF14, WIPF1

Cellular Movement	Migration of myeloid cells	4.62E-04	Decreased	-3.683	44	ADAM10, ADAM15, ADAM17, APP, BTK, CCL5, CCR1, CXCL1, CXCL6, CXCL9, CXCR2, CYBB, F10, F11R, FPR1, HCK, HMOX1, ICAM1, IL1B, ITGA4, ITGAX, LAMA5, LSP1, MGAT5, mir-133, MMP14, MTOR, MYLK, MYO1F, NINJ1, PDE4B, PECAM1, PPM1D, PTEN, PTPN6, RHOA, RTN4, S100A9, SIRPA, SWAP70, TLR2, TLR4, TLR7, TNFRSF1A
Cancer, Organismal Injury and Abnormalities, Reproductive System Disease	Endometrial adenosquamous carcinoma	4.63E-04			9	CSF3R, HSP90AA1, HSP90AB1, HSP90B1, KRAS, PTEN, TUBA1A, TUBA1C, TUBB2A
Cancer, Hematological Disease, Organismal Injury and Abnormalities	Philadelphia chromosome negative hematological system tumor	4.72E-04			29	APC, ARNTL, ASXL1, BCL2L11, CCL5, CSF1R, CSF3R, CUX1, HBA1/HBA2, HCK, IFNAR1, JAK1, KRAS, let-7, LYN, mir-26, MS4A1, NR3C1, PDE4B, PDE8A, PTGS2, SF3B1, SH2B3, STK24, TET2, TUBA1A, TUBA1C, TUBB2A, U2AF1/U2AF1L5
Cell-To-Cell Signaling and Interaction, Hematological System Development and Function, Immune Cell Trafficking, Inflammatory Response	Activation of phagocytes	4.76E-04	Decreased	-2.786	78	ADAM10, ALS2, ANXA2, APOA1, APP, ATG7, BID, BTK, CASP8, CCL5, CD14, CD84, CD86, CD93, CEACAM3, CLEC4M, CLEC7A, CNR1, CSF1R, CX3CR1, CXCL1, CXCL6, CXCR2, CYBB, DDIT3, EPO, FCGR2A, FPR1, FPR2, GC, HCK, HLA-A, HMOX1, HSP90B1, HSPD1, ICAM1, IFNAR1, IGF1, IL1B, IL1RN, LCP2, let-7, LILRA2, LILRB3, LTBR, LYN, MERTK, PF4, PILRB, PPM1D, PRKCD, PTGS2, PTPN6, PTPRE, RAB27A, RGMA, RHOA, RIPK2, S100A9, SCN9A, SIGLEC9, SIRPA, SLC11A1, STAT3, SYK, TLR2, TLR4, TLR5, TLR7, TNFRSF1A, TNFSF10, TNFSF14, TRAF3, TREM1, TREML2, TYROBP, VTCN1, ZBTB46
Hematological Disease, Infectious Diseases, Organismal Injury and Abnormalities	Endotoxin shock response	4.78E-04	Decreased	-2.313	22	ADAM17, B2M, BID, CD14, CLIC4, CXCL6, ENTPD1, F2R, HCK, ICAM1, IFI16, IFNGR1, IL1RN, NR3C1, PTGS2, RPS6KA5, S100A9, STAT3, TERF2IP, TLR4, TNFRSF1A, TREM1
Cancer, Hematological Disease, Immunological Disease, Organismal Injury and Abnormalities	B-cell non-Hodgkin lymphoma	4.99E-04		0.314	164	AMPH, ANKLE2, ANO5, ANXA2, APBA1, APOBEC3A, APOBEC3B, APP, ARHGAP17, ASB10, ASMTL, ATN1, ATRN, ATXN3, B2M, BAZ2B, BBS7, BCL2L11, BCL7A, BECN1, BOD1L1, BTG2, BTK, CARMIL1, CARNS1, CASP8, CAV3, CCNDBP1, CDC23, CFLAR, CGB1/CGB2, CHD4, CMSS1, CNR1, CPSF7, CRNN, CSDE1, CSF1R, CSF3R, CYP2A6 (includes others), DCLRE1C, DMTF1, DNAJB14, DOCK2, DPYD, DYRK1A, ETV6, EWSR1, F11R, FAM131C, FCGR2A, FOXP3, FUS, FYB1, GPRIN1, GPSM2, GRB2, GSE1, HAO2, HCLS1, HDAC7, HDAC9, HECA, HLA-G, HMOX1, HSP90AA1, HSP90AB1, HSP90B1, HVCN1, IDH3A, IL1B, IRF8, JAK1, KAT6A, KRAS, LCT, let-7, LRRFIP1, LSM3, MAX, MCL1, MDM2, mir-154, mir-28, MPEG1, MS4A1, MTOR, MYO5B, MYOF, NACA2, NDUFS1, NETO2, NONO, NOTCH2, NR3C1, NUBP1, NUDT6, NXPE4, PCLO, PDGFRA, PECAM1, PLCG2, PLEKHA7, POLR3B, POTEH (includes others), PPM1D, PPP1R12B, PPP6R3, PSMB8, PSMD1, PSMD2, PSME3, PTEN, PTGS2, PTPRE, PWWP3A, RAB38, RAB4A, RAF1, RBM4, RESF1, RHOA, RICTOR, RPS15, RTTN, SCN9A, SEC14L1, SF3B1, SGK1, SHROOM3, SMARCA2, SORL1, SRPK2, STAT3, STIP1, STXBP6, SWAP70, TAF1, TDRD1, TET2, THBS2, TLR2, TLR7, TNFRSF10C, TNIP1, TRAF3, TRIM55, TRIP12, TRPM6, TTC21B, TUBA1A, TUBA1C, TUBB2A, UBE2F, UNC5C, VDAC1, XRCC5, YAE1, YWHAE, YWHAZ, ZMYM3, ZNF615, ZNF700, ZNF714
Cancer, Hematological Disease, Immunological Disease, Organismal Injury and Abnormalities	Aggressive NK-cell leukemia	5.09E-04			6	HSP90AA1, HSP90AB1, HSP90B1, NR3C1, STAT3, TNFSF10
Cellular Function and Maintenance	Endocytosis by dendritic cells	5.09E-04		-1.664	6	HMOX1, RHOA, STK4, SWAP70, TLR2, TLR4
Cancer, Organismal Injury and Abnormalities, Respiratory Disease	Advanced lung cancer	5.21E-04		-1.404	57	ADM, AKAP12, ANGPTL4, ANXA2, B2M, BRCA1, CALU, CASP8, CCL5, CLCN3, CPEB1, CSF1R, CSF3R, CTLA4, CTNND1, CTSB, CTSZ, FTL, G3BP2, HSP90AA1, HSP90AB1, HSP90B1, HSPA1A/HSPA1B, IGF1R, IL1B, KLF6, KRAS, let-7, LYN, MAP4, MDM2, MERTK, mir-133, mir-24, mir-26, MKNK1, MTOR, NKD2, NR3C1, NTRK1, PDGFRA, PTEN, PTGS2, RAF1, RHOA, RIOK3, RIPK3, SCRIB, SKP2, SOD2, STAT3, TMBIM6, TUBA1A, TUBA1C, TUBB2A, YWHAE, ZEB2

Molecular Transport, Protein Synthesis, Protein Trafficking	Localization of autoantibody	5.38E-04		1.673	8	ATG7, BECN1, CYBB, LYN, MERTK, PRKCD, STAT3, TNIP1
Cell-To-Cell Signaling and Interaction, Cellular Function and Maintenance, Hematological System Development and Function, Inflammatory Response	Phagocytosis of red blood cells	5.45E-04		-0.901	23	ACTR2, ARPC2, CD93, DOCK2, FCGR2A, HCK, ICAM1, IL1B, JAK1, KAT6A, KCTD5, LYN, NCKAP1L, PLEK, PRKCD, PTEN, RAC2, RIT1, SIRPA, SYK, UBE2L3, WASF2, ZNF217
Cell-To-Cell Signaling and Interaction, Hematological System Development and Function, Inflammatory Response	Binding of professional phagocytic cells	5.52E-04	Decreased	-3.302	46	ADAM10, ADAM17, ADGRE2, APOA1, APP, B4GALT1, BTK, CCL5, CCR1, CD14, CLEC4M, CNR1, CSF3R, CTSZ, CXCL1, CXCR2, CYBB, F2R, FCGR2A, FPR1, FUT7, HCK, ICAM1, IL1B, ITGA4, ITGAX, LCP1, LGALS8, LILRB3, LSP1, LYN, MGAT5, MSN, NOTCH2, PAK2, PF4, PLCB3, PTGS2, PTPN6, RAC2, RHOA, RHOB, S100A9, TLR2, TLR4, TLR5
Nervous System Development and Function	Neuroprotection of brain	5.53E-04		-0.845	12	APP, CNR1, DDIT3, EPO, IGF1, IL1RN, NFATC4, PHLPP1, PTGS2, STAT3, STIP1, WDFY3
Cell Death and Survival	Cell death of macrophages	5.54E-04		-1.154	32	APP, CASP8, CCL5, CD14, CFLAR, CTSB, CYBB, DDIT3, DFFA, ENTPD1, HMOX1, IFNAR1, IL1B, LYZ, MCL1, MEFV, MTOR, NAMPT, NFE2L2, PELI2, PTEN, PTPN6, RALBP1, RIPK3, SOD2, STAT3, STK4, TLR2, TLR4, TNFRSF1A, TNFSF10, TREM1
Neurological Disease	Progressive neurological disorder	5.55E-04		0.6	208	ACTG1, ADAM10, ADAM17, ALDH5A1, ALS2, AMPH, ANXA2, ANXA5, APLP2, APOA1, APOA2, APP, ARHGDIB, ARL6IP5, ARMC2, ARNT, ARNT2, ASAH1, BCL2L11, BECN1, BGN, BRCA1, CAMK2A, CAPZB, CASP8, CCDC88A, CCL5, CCT2, CD14, CDCP2, CELF2, CFLAR, CNP, CNR1, COL1A2, CPT1A, CSF1R, CSF3R, CTLA4, CTSB, CXCL1, CXCL16, CXCR2, CXCR3, CYP26B1, CYP51A1, DDC, DDIT3, DHCR7, DNAJB11, DNAJB6, DOCK8, DOK5, DPYSL2, DYRK1A, EEF2K, ELN, ENO3, EZR, F2R, FCGR2A, FCGR2C, FOXO3, FOXP3, FRMD4B, FTH1, FTL, FUS, GAB2, GALC, GAPDH, GAS7, GC, GCNT2, H3-3A/H3-3B, HBA1/HBA2, HFE, HLA-G, HMOX1, HNRNPA1, HNRNPA2B1, HSP90AA1, HSP90AB1, HSP90B1, HSPA1A/HSPA1B, HSPA5, HSPD1, ICAM1, IFNAR1, IFNGR1, IGF1, IGF1R, IL1B, IL1R2, IREB2, ITGA4, JPT1, KIAA0040, KIF1A, KRAS, LARP4, let-7, LGMN, LIMS1, LOC440040, LRP8, LRPAP1, M6PR, MBP, MEF2C, MERTK, mir-101, mir-103, mir-133, mir-154, mir-24, mir-26, mir-28, mir-3180, mir-3690, mir-422, mir-515, mir-550, mir-551, mir-657, MIR4270, MS4A1, MS4A4A, MS4A6E, MTHFD2, MTOR, MTRR, MYOG, NFATC4, NFE2L2, NFS1, NOM1, NR3C1, NTRK1, OAZ1, OPA1, PARK7, PDE4B, PDGFRA, PDIA3, PGAM2, PIP4P2, PLA2G4C, PLCG2, PRKAR1A, PRKCD, PRL, PSAP, PSMB8, PSMC1, PTEN, PTGS2, PTPRE, RAF1, RHOA, RHOB, RNASET2, RNF114, RNF6, RPL13A, RPL5, RTN1, RTN3, RTN4, S100A9, SCARB2, SCN9A, SGK1, SHROOM3, SLC52A2, SLC6A6, SNAP91, SOD2, SORL1, SRPK2, ST8SIA4, STAT3, STIP1, TAF1, TBK1, TFRC, THAP1, TLR2, TLR4, TNFRSF1A, TNNC1, TRIM5, TRIO, TSHZ3, TUBA1A, TUBA1B, TUBA1C, TUBB2A, TYROBP, UQCRC2, VDAC1, VDR, VIM, VPS35, WDFY3, WWTR1, YWHAZ, ZNF721
Cell-To-Cell Signaling and Interaction	Response of microglia	5.57E-04		-1.635	11	APP, BECN1, CD14, DOCK2, HMOX1, IL1B, MERTK, PARK7, S100A9, TLR2, TLR4
Cellular Development, Hematological System Development and Function, Lymphoid Tissue Structure and Development	Maturation of bone marrow-derived dendritic cells	5.57E-04		-0.756	11	BTK, CLEC7A, ELF3, HMOX1, HSPD1, IL1B, LYN, RHOA, SWAP70, TLR4, TLR7

Cancer, Organismal Injury and Abnormalities, Reproductive System Disease	Breast or ovarian cancer	5.75E-04		0.286	622	ABCC2, ACBD3, ACP3, ACTG1, ACTR2, ACYP1, ADAM10, ADAM15, ADAM17, ADGRA1, AGO2, AIF1, AIG1, AIPL1, AK9, AKAP12, ALDH3A1, ALDH5A1, ALKBH1, ALKBH3, ALS2, AMPH, ANAPC13, ANGPTL5, ANKRD42, ANXA3, AOPEP, AP5M1, APBA1, APC, APOA1, APOB, APOBEC3B, APP, ARF4, ARHGAP19, ARHGDIB, ARMC3, ARMCX5-GPRASP2/GPRASP2, ARNT, ARNTL, ASB10, ASXL1, ATG2B, ATG7, ATL3, ATN1, ATP6V1B2, ATRN, AURKB, B2M, BCL2L11, BECN1, BGN, BLVRA, BMP2K, BNIPL, BOD1L1, BRCA1, BRIP1, BTG2, C17orf80, C18orf25, C1GALT1C1, C1RL, C7orf25, CACNA1E, CALCOCO2, CAMK2A, CAPZB, CARD16, CARNS1, CASP8, CATSPERD, CCDC47, CCDC88A, CCP110, CD300E, CDC5L, CDH12, CELSR3, CEP128, CEP72, CFLAR, CHCHD5, CHD4, CKMT2, CLASP1, CLIC4, CLK2, CNPY3, COG2, COG5, COL1A2, COL7A1, CPEB1, CPQ, CPT1A, CRKL, CRY2, CRYBG3, CSDE1, CSF1R, CSF3R, CT45A10/CT45A5, CTAG2, CTBS, CTLA4, CTNND1, CTNND2, CTSB, CTSC, CTSZ, CUX1, CXCL1, CXCL16, CXCL9, CXCR2, CXCR3, CYBB, CYP24A1, CYP2A6 (includes others), CYP4F3, CYTH4, DAB2, DDX17, DDX23, DDX27, DDX39A, DDX3X, DDX5, DEF6, DENND3, DGLUCY, DHCR7, DHX8, DIP2B, DLGAP4, DNAJB12, DNAJB6, DNAJC2, DNAJC7, DNM3, DOCK8, DOK5, DPF3, DPH2, DPYD, DSE, DUSP5, DYRK1A, EBLN2, ECE1, EFS, EIF1AX, EIF3A, EIF4G3, ELF3, ELOA, EOGT, EPB41L3, EPM2AIP1, ERO1A, ETV6, EVI5L, EXOC3L4, EXT1, EZR, F10, F11R, F13A1, F2R, F8, FAM126B, FAM209A, FAM214B, FBLN2, FBXO38, FCAMR, FEZ1, FKBP5, FNBP1L, FOXL2, FOXO3, FOXP3, FPR2, FRMD4B, FTH1, FUBP3, FUS, FUT7, FZD1, FZD3, G3BP2, GAB2, GAL3ST1, GAPDH, GAS7, GASK1B, GATA5, GBE1, GC, GLE1, GLUL, GLYR1, GNB4, GOLGB1, GPATCH4, GPR75, GSTA1, GTF3C3, GYG1, H2AC18/H2AC19, H2BC21, H3-3A/H3-3B, HAMP, HBA1/HBA2, HBB, HBP1, HCCS, HCK, HCLS1, HDAC9, HLA-A, HLA-E, HLA-G, HMOX1, HNRNPA1, HNRNPA2B1, HNRNPH2, HOTAIR, HOXA3, HOXA4, HSD17B12, HSP90AA1, HSP90AB1, HSP90B1, HSPA5, HSPB7, HSPD1, HTATIP2, HTR1F, HVCN1, IDH3A, IER2, IFNGR1, IFNGR2, IGF1, IGF1R, IGF2BP3, IGF2R, IGFBP4, IGSF6, IL1B, ILF3, IQSEC3, IRX4, ITGAX, JAK1, JAML, JMJD1C, JMJD4, JPH4, JPT1, JPX, KAT6A, KAT6B, KCNJ4, KDM1B, KDM5A, KDM7A, KIF1C, KIF26B, KLF6, KLHL15, KRAS, KRT23, L3MBTL3, LAMA5, LAMTOR5, LARP4, LARP6, LAS1L, LASP1, LAT2, LEFTY1, let-7, LETM2, LGALS8, LGR5, LHCGR, LILRA1, LIMK2, LINC00511, LIPM, LMTK2, LONRF3, LRP2, LRP8, LRRFIP1, LSP1, LY6K, LY86, LY9, LYVE1, MAFF, MAGT1, MAN2A2, MAP3K1, MAP4, MAP4K4, MAPRE1, MAPRE3, MARF1, MAX, MCCC2, MCL1, MDM2, MED23, MEF2C, mir-101, mir-103, mir-122, mir-154, mir-202, mir-24, mir-26, mir-28, MMP14, MORN5, MRPL15, MS4A1, MS4A14, MS4A4A, MS4A7, MSH6, MT1A, MT1F, MT1X, MTCH2, MTDH, MTFR2, MTHFD2, MTOR, MTTP, MUC1, MUC15, MYBBP1A, MYH15, MYLK, MYO5B, MYOF, MYOG, NABP1, NAMPT, NASP, NCF2, NCKAP1L, NCL, NDE1, NFATC4, NFE2L2, NHSL1, NIN, NLGN3, NLGN4X, NONO, NOTCH2, NOXRED1, NPC1L1, NR3C1, NTRK1, NUMB, NUP50, NUP93, NXPE4, OPA1, OR2A14, OR4D10, OR5AC2, OSBPL11, OTUD3, PAK2, PCLO, PCOLCE, PDAP1, PDGFRA, PDIA3, PDLIM5, PDS5B, PEAK1, PECAM1, PEX19, PF4, PHF12, PILRA, PIN4, PIP5K1A, PITX2, PIWIL1, PLAGL2, PLB1, PLEKHA7, PLXDC2, PLXNA4, PODNL1, POTEH (includes others), PPM1D, PPP1R12B, PPP1R17, PPP4R2, PQBP1, PRC1, PRKCD, PRKCG, PRL, PRPF6, PRR12, PRSS55, PSMD12, PSMD4, PSMD7, PTEN, PTGS2, PTPRE, PUDP, PWWP3A, RAB31, RAB3GAP2, RABGAP1L, RAD51C, RAD51D, RAF1, RALBP1, RALGPS1, RAP1A, RASEF, RBMXL3, RCBTB2, RFPL2, RFX3, RGCC, RGS2, RHBG, RHOA, RHOB, RICTOR, RIN2, RIOK1, RIOK2, RNF103, RNF103-CHMP3, RNF121, RNF130, RNF149, RPF2, RPGRIP1, RPL4, RPL5, RTCB, RTN3, RTN4, RTTN, S100A14, S100A9, SBF2, SCRIB, SCRT2, SEC14L1, SEC61A2, SENP2, SETDB1, SF3B1, SH2B3, SH3BP2, SIPA1L2, SIRPB1, SKP2, SLC22A18, SLC22A4, SLC24A4, SLC25A32, SLC31A1, SLC35F4, SLC43A3, SLC4A2, SLC6A6, SLC8A1, SLITRK6, SMARCA2, SMTN, SNX27, SOD2, SORL1, SP100, SP3, SPAG9, SPATA5, SPEF2, SPHK2, SPOP, SRPK1, SRPK2, SSH3, STAT3, STEAP4, STX3, SUSD6, SWAP70, SYK, SYNE4, SYT17, SZT2, TAF1, TAF7, TAGLN2, TBC1D12, TBC1D8, TBC1D9, TBL1X, TBX5, TCAIM, TCF4, TCP1, TDGF1, TDRD1, TERF2IP, TET2, TFRC, THBS2, THEG, THRAP3, TLR4, TLR5, TM2D2, TM7SF3, TMEM140, TMEM43, TMEM70, TMTC2, TNFRSF1A, TNFSF10, TNNC1, TOR1B, TPM3, TRIM46, TRIM5, TRIM65, TRIO, TRIP10, TRIP12, TRMT9B, TRPM6, TSG101, TSHZ3, TTI2, TUBA1A, TUBA1B, TUBA1C, TUBB2A, U2AF1/U2AF1L5, UBAP2L, UBE2E3, UBE4B, USP15, USP19, USP32, UTP4, VCAN, VCPIP1, VDAC2, VDR, VIM, VTCN1, VTI1B, WASF2, WASF3, WDFY3, WDR19, WIPF1, WNK1, WNK3, WSB1, XPNPEP3, XRCC5, YBX1, YPEL5, YWHAZ, ZAN, ZBTB21, ZEB2, ZFPM2, ZMPSTE24, ZMYM3, ZNF10, ZNF143, ZNF165, ZNF217, ZNF229, ZNF235, ZNF24, ZNF281, ZNF3, ZNF33B, ZNF398, ZNF41, ZNF45, ZNF461, ZNF516, ZNF525, ZNF555, ZNF570, ZNF606, ZNF615, ZNF667, ZNF677, ZNF684, ZNF711, ZNF738, ZNF746, ZNFX1, ZSCAN2
Cancer, Hematological Disease, Organismal Injury and Abnormalities	Philadelphia chromosome negative chronic myeloproliferative neoplasm	5.84E-04			25	APC, ARNTL, ASXL1, BCL2L11, CCL5, CSF1R, CSF3R, CUX1, HBA1/HBA2, HCK, IFNAR1, JAK1, KRAS, let-7, LYN, mir-26, NR3C1, PDE4B, PDE8A, PTGS2, SF3B1, SH2B3, STK24, TET2, U2AF1/U2AF1L5
Cell-To-Cell Signaling and Interaction, Hematological System Development and Function	Interaction of lymphocytes	6.04E-04	Decreased	-3.655	41	APBB1IP, APOA1, ATRN, BTK, CCL5, CCR1, CD86, CLEC4M, CTLA4, CXCL9, CXCR3, DOCK2, DOCK8, EZR, FUT7, FYB1, ICAM1, IFNGR1, IL1B, ITGA4, JAK1, LCP2, LTBR, MAP3K2, MSN, NEDD9, NR3C1, PECAM1, PRL, PTPN6, RAC2, RAP1A, RHOA, RICTOR, STK4, SWAP70, TFRC, THBS2, TLR2, TLR4, TNFSF14

Cell Signaling	Viral life cycle	6.05E-04		1.502	29	APOBEC3A, APOBEC3B, CALCOCO2, CCL5, CHMP2A, CHMP3, CHMP4B, CHMP6, CXCR3, DDX5, IFI16, ILF3, mir-122, mir-24, NUP160, NUP50, NUP58, NUP62, NUP93, PTGS2, RAD52, SEC13, SRPK1, SRPK2, TLR4, TNIP1, TNPO3, TSG101, VPS4B
Organismal Survival	Organismal death	6.11E-04	Increased	14.151	470	ACTG1, ADAM10, ADAM15, ADAM17, ADM, AGO2, AKAP12, ALDH5A1, ALKBH3, AMPH, ANGPTL4, ANTXR2, ANXA2, AP1G1, APBA1, APC, APLP2, APOA1, APOB, APP, ARF1, ARF4, ARHGDIB, ARID4B, ARIH2, ARNT, ARNT2, ARNTL, ARPP19, ASAH1, ASXL1, ATG3, ATG7, ATN1, ATOX1, ATP7B, ATXN3, AURKB, B2M, B4GALT1, BBS7, BCAS3, BCL2L11, BCL7A, BECN1, BEST1, BGN, BID, BNIP3L, BRCA1, BRIP1, C1GALT1C1, C7orf25, CA4, CAP1, CAPZB, CASP8, CBY1, CCDC47, CCNK, CCP110, CCR1, CCR10, CD14, CDA, CDK2AP1, CELF1, CELF2, CELSR3, CFLAR, CHD4, CHM, CHMP2A, CKS2, CLCN3, CLEC1B, CLEC4M, CLEC7A, CLIC4, CNP, CNPY3, CNR1, COL7A1, CPLX2, CPT1A, CREB1, CRKL, CSAD, CSF1R, CTLA4, CTNND1, CTSB, CUX1, CX3CR1, CXCL9, CXCR2, CXCR3, CYBB, CYP24A1, CYP26B1, CYP51A1, DAB2, DCLRE1C, DDIT3, DDX17, DDX3X, DDX5, DGAT2, DHCR7, DLD, DMTF1, DNAJB4, DNAJB6, DNM3, DOCK2, DPH3, DPP10-AS1, DPYD, DYRK1A, ECE1, EEF1E1, EEF2K, EHD3, ELK3, ELN, ELOA, EPB41L3, EPO, ERCC5, ERO1A, ETV6, EVC2, EWSR1, EXT1, EXTL3, F10, F13A1, F2R, F8, FAH, FOXL2, FOXO3, FOXP3, FPR2, FRS2, FTH1, FTL, FTX, FUS, GAB1, GALNT1, GATA5, GATAD2A, GBE1, GCLC, GLCE, GLT8D2, GNG7, GRB2, GSE1, H2AC18/H2AC19, H3-3A/H3-3B, HAMP, HCK, HDAC7, HFE, HLA-G, HMOX1, HNRNPA1, HOTAIR, HOXA3, HOXA4, HOXA7, HSBP1, HSD17B12, HSP90AA1, HSP90AB1, HSP90B1, HSPA5, HSPB7, ICAM1, IFNAR1, IFNGR1, IGF1, IGF1R, IGF2BP3, IGF2R, IL1B, IL1RN, ILF3, IP6K2, IPMK, IREB2, IRF8, ITGA4, ITGB8, JAK1, JPH4, JPX, KAT6A, KCNAB2, KCNJ2, KDM5A, KIDINS220, KIF1A, KLF6, KLF7, KMT5B, KRAS, L3MBTL2, L3MBTL3, LAT2, LCP1, LCP2, LEFTY1, LGR5, LIAS, LIMS1, LIN7A, LMTK2, LRP2, LRP8, LRPAP1, LTBR, LUCAT1, LY6K, LYN, LYZ, M6PR, MAFF, MAN2A2, MAP3K1, MAP4, MAP4K4, MAPKAPK3, MAX, MCL1, MCM3, MDM2, MED23, MEF2C, MERTK, MEX3B, mir-122, mir-133, mir-137, mir-154, mir-202, mir-26, mir-299, MLKL, MMP14, MORF4L1, MSH6, MSN, MTDH, MTF1, MTHFD2, MTOR, MTTP, MUC1, MYBBP1A, MYCNOS, MYH14, MYOF, MYOG, NAMPT, NAPB, NASP, NCOA1, NDC80, NDEL1, NFATC4, NFE2L2, NFKBIZ, NIN, NINJ1, NLGN3, NOTCH2, NR3C1, NTRK1, NUAK2, NUBP1, NUMB, NUP62, OAT, OPA1, OXT, P2RX1, PAK2, PCK1, PCLO, PCYT1A, PDCD4, PDE4B, PDGFRA, PDIA3, PDS5B, PER2, PHF12, PIGA, PIP5K1A, PITPNA, PITX2, PLAGL2, PLCB3, PLCG2, PLCL1, PLP1, PNN, PNO1, PPIF, PPM1D, PPP6C, PRKAR1A, PRKCD, PRKG1, PSAP, PSMC1, PSMC2, PSMD4, PTEN, PTF1A, PTGS2, PTPN6, PURA, RAB11A, RAB27A, RAB31, RAB5A, RAB8A, RAC2, RAD51C, RAD51D, RAD52, RAF1, RALB, RAMP2, RAP1A, RAPGEF2, RASSF2, RBMS1, RBPJ, RFX3, RGMA, RHOA, RICTOR, RIPK3, RNASET2, RPL4, RPL5, RPS6KA5, RTEL1, RTN4, RUFY3, S100A9, SAV1, SCARB2, SCN9A, SCRIB, SDHD, SEL1L, SERTAD1, SESTD1, SETDB1, SF3B1, SFRP4, SH2B3, SHC3, SIAH1, SKP2, SLC22A4, SLC22A5, SLC25A37, SLC31A1, SLC4A2, SLC8A1, SMAD1, SMARCC2, SMTN, SNAP23, SNAP91, SNAPIN, SNX13, SNX27, SOD2, SP1, SPINK5, SPOP, SPOUT1, SPRTN, SRGAP3, SRGN, SRSF1, SRSF3, SSBP2, ST8SIA4, STAMBP, STAT3, STEAP4, STIP1, STK35, STK4, SUDS3, SUFU, SUPT4H1, SUSD6, SYK, SYT5, TAF7, TASP1, TBK1, TBX5, TCF15, TCF4, TDGF1, TET2, TFRC, THAP1, THBS2, THOC5, TIFA, TLR2, TLR4, TLR5, TLR7, TMEM107, TMOD3, TNFRSF1A, TNFSF10, TNIP1, TPM3, TRAF3, TREM1, TRIM55, TRIO, TRIP12, TRPM6, TSG101, TSHZ3, TXNRD1, U2AF1/U2AF1L5, UBA3, UBE2B, UBE2L3, UBE4B, UBR2, USP17L2 (includes others), USP4, VCAN, VDAC1, VDR, VIM, VPS41, VTCN1, VTI1A, VTI1B, WASF2, WIPF1, XRCC5, YBX1, YBX3, YWHAE, ZDHHC16, ZEB2, ZFPM2, ZMPSTE24, ZNF148, ZNF24, ZNF281
Cell-To-Cell Signaling and Interaction, Inflammatory Response	Immune response of dendritic cells	6.17E-04	Decreased	-2.008	14	CD86, CLEC9A, FCAMR, FCGR2A, HMOX1, HSP90AA1, mir-24, MUC1, PSMB8, SEMA4A, SWAP70, SYK, TLR4, TNFSF4
Cell-To-Cell Signaling and Interaction, Hematological System Development and Function	Binding of T lymphocytes	6.20E-04	Decreased	-3.539	32	APBB1IP, APOA1, CCL5, CCR1, CD86, CTLA4, CXCL9, CXCR3, DOCK2, EZR, FUT7, FYB1, ICAM1, IFNGR1, IL1B, ITGA4, JAK1, LCP2, LTBR, MAP3K2, MSN, NR3C1, PECAM1, PRL, RAC2, RAP1A, RHOA, RICTOR, STK4, THBS2, TLR4, TNFSF14
Cell-To-Cell Signaling and Interaction, Cellular Movement	Recruitment of blood cells	6.33E-04	Decreased	-3.512	71	ADAM10, ADAM17, ALOX5AP, APOA1, APOB, APP, ATG7, B4GALT1, CASP8, CCL23, CCL5, CCR1, CD14, CD93, CLEC1B, CLEC7A, CNR1, CSF1R, CTSC, CX3CR1, CXCL1, CXCL16, CXCL6, CXCL9, CXCR2, CXCR3, ENTPD1, F13A1, FCGR2A, FPR2, FUT7, GAB2, GC, HCK, HMOX1, HSPA1A/HSPA1B, ICAM1, IFNAR1, IL1B, IL1RN, ITGA4, KRAS, LSP1, LYN, LYZ, MGAT5, NFE2L2, NINJ1, P2RX1, PDE4B, PECAM1, PTEN, RAP1A, RHOA, RHOB, RIPK2, RTN4, SIGLEC9, SOD2, ST3GAL6, STAT3, SWAP70, SYK, THBS2, TLR2, TLR4, TLR5, TLR7, TNFRSF1A, TREML2, VDR
Cellular Movement, Hematological System Development and	Migration of mononuclear leukocytes	6.35E-04	Decreased	-4.165	82	ADAM10, ADAM17, ANXA2, APBB1IP, APP, BTK, CCL23, CCL5, CCR1, CCR10, CD86, CTLA4, CUX1, CX3CR1, CXCL1, CXCL16, CXCL9, CXCR2, CXCR3, CYP26B1, DEF6, DEFB103A/DEFB103B, DOCK2, DOCK8, DPYSL2, EFS, EZR, F11R, FOXP3, FUT7, FYB1, HCLS1, HLA-A, HLA-G, HSPD1, ICAM1, IFNAR1, IFNGR1, IL1B, ITGA4, ITGAX, JAK1, LCP1, LCP2, LTBR, MAP3K2, MAPKAP1, mir-133, MMP14, MSN, MTOR, MYLK, NINJ1, NR3C1, PECAM1, PILRA, PLCB3, PRKAA1, PROK2, PTEN, PTGS2, RAC2, RAP1A, RHOA, RICTOR, SCRIB, SERPINB3, SIRPA, SOS2, SPHK2, STAT3, STK4, SWAP70, THBS2, TLR2, TLR4, TLR7, TNFRSF1A, TNFSF14, TNFSF4, TNIP1, VTCN1

Function, Immune Cell Trafficking						
Cellular Movement, Hematological System Development and Function, Immune Cell Trafficking	Cell movement of antigen presenting cells	6.35E-04	Decreased	-4.517	82	ADAM17, APOA1, APP, B4GALT1, BECN1, BID, CASP8, CCDC88A, CCL5, CCR1, CD86, CLEC1B, CLEC4M, CNP, CNR1, CRKL, CSF1R, CTSZ, CX3CR1, CXCL1, CXCL9, CXCR2, CXCR3, CYBB, DEFB103A/DEFB103B, DOCK2, DOCK8, ELN, EPO, FPR1, FPR2, GAL3ST1, HAMP, HCK, HCLS1, HEBP1, HMOX1, HSPA5, ICAM1, IFNGR1, IL1B, IL1RN, KLF6, LITAF, LSP1, MYLK, NARS1, NFE2L2, NFKBIZ, NINJ1, OPA1, PF4, PLCG2, PLP1, PRKCD, PSMB8, PTEN, PTGS2, PTMA, PTPN6, RAC2, RHOA, RHOB, RPL13A, SCN9A, SEMA4A, SH2B3, SIRPA, STAT3, STK4, SWAP70, TAFA4, THBS2, TLR2, TLR4, TLR7, TNFSF4, TNIP1, TYROBP, VCAN, VTCN1, YBX1
Protein Synthesis	Metabolism of protein	6.37E-04	Decreased	-2.387	233	A1CF, ACO1, ADAM10, ADAM15, ADAM17, ADM, AGO2, ALDH3A1, ALKBH1, APC, APLP2, APOA1, APOA2, APOB, APOL1, APP, AREL1, ARIH2, ARNTL, ATF5, ATF7IP, ATG7, ATXN3, AURKB, B2M, B4GALT1, BTG2, BTK, C4BPB, CALU, CASC3, CASP8, CAV3, CCDC47, CCT2, CDC23, CDKL2, CGA, CGB3 (includes others), CHMP6, CNBP, CPEB1, CPN1, CPQ, CREB1, CTNND1, CTSB, CTSC, CTSZ, CYP51A1, DDIT3, DDX3X, DLD, DNAJB12, ECE1, ECPAS, EDEM3, EEF2K, EIF1AX, EIF3A, EIF3G, EIF3I, EIF4G3, EIF4H, FOXO3, FTH1, FUS, GAB2, GAPDH, GNL3L, H3-3A/H3-3B, H3C1, H3C13, H4C14, HCK, HELZ, HERPUD1, HSP90AA1, HSP90AB1, HSP90B1, HSPA1A/HSPA1B, HSPA5, HSPD1, ICAM1, IFNAR1, IGF1, IGF2BP3, IGFBP4, IL1B, IL1RN, ILF3, IREB2, ITM2B, JAK1, KCTD21, KLHL15, KRAS, LARP4, LARP4B, LARP6, let-7, LGMN, LYN, LYZ, MAP3K1, MAP4K4, MARS1, MDM2, mir-101, MKNK1, MMP14, MRPL15, MRPL18, MRPL28, MRPL55, MRPS10, MRPS18A, MRRF, MTOR, MTRF1L, MTTP, MYBBP1A, MYCNOS, NCBP1, NCL, NLK, NLN, NPC1L1, NR3C1, NRDC, OAZ1, OAZ2, OS9, OTUD3, OXA1L, PABPC1, PARK7, PCOLCE, PDCD4, PEX19, PHLPP1, PIWIL1, PPM1G, PRKAA1, PRKCG, PRL, PSMC2, PSMD11, PSMD2, PSME3, PTCD3, PTEN, RASSF2, RBM4, RFFL, RGS2, RNASET2, RNF11, RNF149, RNF185, RNF20, RNF40, RNF6, RPL13A, RPL18, RPL18A, RPL28, RPL38, RPL39, RPL4, RPL5, RPS15, RTN4, S100A9, SAT1, SAV1, SCG3, SEL1L, SENP2, SENP8, SERPINB3, SIAH1, SKP2, SOD2, SORL1, SP1, SPINK5, SPOP, SRSF3, STAT3, STAU1, STIP1, STK4, STX12, SUFU, SVBP, SWAP70, SYK, TAF1, TASP1, TBL1X, TCP1, TGOLN2, TLR4, TMPRSS7, TMPRSS9, TNFSF10, TNIP1, TOPORS, TRABD2B, TRAF3, TRIP12, TSG101, TSPAN1, TTF1, TYROBP, UBA3, UBE2B, UBE2L3, UBE4B, UBR2, USP19, USP4, VCAN, VDR, VIM, VPS35, WARS1, XPNPEP3, YBX1, YTHDF3, ZFPM2, ZMPSTE24
Cellular Movement	Invasion of cells	6.39E-04	Decreased	-5.271	212	ADAM10, ADAM15, ADAM17, ADM, AGO2, AKAP12, ALKBH3, ANGPTL4, ANTXR2, ANXA2, APC, APP, ARF4, ARRDC3, ATG7, BARX2, BRCA1, BTG2, CAP1, CAVIN2, CCDC88A, CCR1, CD14, CELF2, CGA, CGB3 (includes others), CLCA2, CLEC4M, CNR1, COL7A1, CRKL, CTNND1, CTNND2, CTSB, CTSZ, CXCL1, CXCL6, CXCR2, CXCR3, CYP2J2, DAB2, DEF6, DEFB103A/DEFB103B, DKK3, DNAJB4, DNAJB6, DPP10-AS1, DPYSL2, DSE, ECE1, EIF3A, ELF3, ETV6, EYA3, EZR, F11R, F2R, FAIM2, FBLN2, FGD4, FNBP1L, FOXO3, FOXP3, FTX, GAB1, GAB2, GIT2, GMFG, GRB2, GSE1, HBP1, HDLBP, HMOX1, HNRNPA2B1, HOTAIR, HSBP1, HSP90AA1, HSP90AB1, HSP90B1, HSPA1A/HSPA1B, HSPA5, HTATIP2, IFNAR1, IGF1, IGF1R, IGF2BP3, IL1B, ILF3, ITGA4, ITGAX, ITGB8, JPX, KDM5A, KLF6, KMT5B, KRAS, LAMA5, LASP1, LCP1, let-7, LGMN, LIMK2, LINC00887, LRPAP1, LUCAT1, LYN, MACIR, MAP4, MAP4K4, MAPRE3, MDM2, MERTK, MGAT5, mir-103, mir-122, mir-133, mir-138, mir-154, mir-24, mir-26, mir-28, mir-515, MMP14, MTDH, MTOR, MUC1, MUC13, MYLK, NAMPT, NCOA1, NCOA4, NEDD9, NFATC4, NFE2L2, NKD2, NONO, NOTCH2, NUAK2, NUMB, PAK2, PARK7, PDCD4, PDGFRA, PECAM1, PHLPP1, PIP5K1A, PPIF, PRKAA1, PRKCD, PRL, PSMD10, PTEN, PTGS2, PTPN6, RAB5A, RALB, RALBP1, RAP1A, RHOA, RHOB, RICTOR, RIOK3, S100A14, S100A9, SCRIB, SDCBP, SEC24D, SEL1L, SETDB1, SKP2, SMAD1, SNAP23, SOD2, SP1, SP100, SPHK2, SRGN, SSX2IP, STAT3, STK24, STK38L, SYK, TAGLN2, TCF4, TDGF1, THBS2, TJP1, TLR2, TLR4, TM9SF4, TMBIM6, TNFSF10, TRAF3, TRIO, TRIP10, UNC5C, USP4, VCAN, VDAC1, VDR, VIM, WASF2, WASF3, WNK1, WSB1, WWTR1, YBX1, ZEB2, ZFYVE21, ZMPSTE24, ZNF24, ZNF350
Cellular Function and Maintenance	Function of blood cells	6.39E-04		-0.861	106	AQP9, ARHGDIB, ARIH2, ATG7, B2M, B4GALT1, BCL2L11, BTK, CASP8, CCL5, CCR1, CD14, CD84, CD86, CLCN3, CLEC1B, CLEC6A, CLEC7A, CNPY3, CREB1, CSF1R, CTLA4, CTSC, CTSZ, CX3CR1, CXCR2, CYBB, DCLRE1C, DMTF1, DOCK8, DUSP5, EFS, ENTPD1, EPO, F11R, FCAMR, FCGR2A, FOXP3, FPR2, FUT7, FYB1, GAB2, GIMAP4, HCK, HLA-A, HLA-E, HLA-G, HSP90AA1, ICAM1, IFNAR1, IFNGR1, IFNGR2, IL1B, IRF8, LAT2, LCP1, LGMN, LILRB3, LSP1, LYN, MCL1, MERTK, MMP14, MTOR, NEDD9, NFE2L2, NFKBIZ, NINJ1, PECAM1, PER2, PLCB3, PLCG2, PPM1D, PSAP, PTEN, PTGS2, RAC2, RAP1A, RAPGEF2, RHOA, RIPK2, RIPK3, SEMA4A, SH3BP2, SIGLEC9, SIRPA, SKP2, SPHK2, STAT3, STK4, TAGLN2, TBK1, TCF4, TET2, TLR2, TLR4, TLR5, TLR7, TNFRSF1A, TNFSF10, TNFSF14, TRAF3, TRIP10, TYROBP, VDR, WIPF1
Cell Death and Survival	Cell death of phagocytes	6.39E-04		-0.561	49	ADAM17, APP, BCL2L11, BID, BTK, CASP8, CCL5, CD14, CFLAR, CNR1, CTSB, CXCL1, CYBB, DDIT3, DFFA, ENTPD1, FOXO3, HMOX1, HSP90AB1, IFNAR1, IL1B, IL1RN, IRF8, LYZ, MCL1, MEFV, MLKL, MTOR, NAMPT, NFE2L2, NR3C1, PELI2, PF4, PRKCD, PTEN, PTPN6, RALBP1, RIPK3, SH3BP2, SIGLEC9, SOD2, STAT3, STK4, SYK, TLR2, TLR4, TNFRSF1A, TNFSF10, TREM1
Organismal Injury and Abnormalities, Reproductive System Disease	Adenomyosis	6.40E-04			17	AIG1, ANXA2, CD14, HBA1/HBA2, LGMN, MTHFD2, NR3C1, OXT, PDS5B, PRL, PRRC2C, STX7, STXBP6, TBL1X, TCF4, UBE2B, VDAC1

Cell-To-Cell Signaling and Interaction, Hematological System Development and Function	Binding of lymphocytes	6.47E-04	Decreased	-3.478	38	APBB1IP, APOA1, BTK, CCL5, CCR1, CD86, CTLA4, CXCL9, CXCR3, DOCK2, DOCK8, EZR, FUT7, FYB1, ICAM1, IFNGR1, IL1B, ITGA4, JAK1, LCP2, LTBR, MAP3K2, MSN, NEDD9, NR3C1, PECAM1, PRL, PTPN6, RAC2, RAP1A, RHOA, RICTOR, STK4, SWAP70, TFRC, THBS2, TLR4, TNFSF14
Cell Death and Survival	Apoptosis of myeloid cells	6.57E-04		-0.517	46	ADAM17, APP, ASAH1, BID, BNIP3L, BTK, CASP8, CCL5, CD14, CFLAR, CXCL1, CYBB, DDIT3, DFFA, EPO, FOXO3, IGF1, IL1B, IL1RN, IRF8, LYN, MCL1, MDM2, MEFV, mir-154, MTOR, NAMPT, NFE2L2, PELI2, PF4, PRKCD, PTEN, PTPN6, RAF1, RIPK3, SH3BP2, SIGLEC9, SOD2, STAT3, SYK, TLR2, TLR4, TMOD3, TNFRSF1A, TNFSF10, TREM1
Cellular Movement, Hematological System Development and Function, Immune Cell Trafficking, Inflammatory Response	Cell movement of monocytes	6.59E-04	Decreased	-3.457	40	ADAM17, AIF1, ANXA2, APOA1, APP, ATRN, CCL23, CCL5, CCR1, CX3CR1, CXCL9, CXCR2, DEFB103A/DEFB103B, ELN, F11R, F2R, FPR1, FPR2, HEBP1, ICAM1, IL1B, ITGA4, ITGAX, JAK1, LGMN, mir-133, MMP14, NFKBIZ, NINJ1, PECAM1, PF4, PILRA, PROK2, RAC2, RHOA, S100A14, SIRPA, TLR7, TNFRSF1A, TNIP1
Cellular Movement, Hematological System Development and Function, Immune Cell Trafficking	Cell movement of peripheral blood leukocytes	6.63E-04	Decreased	-3.509	21	ADAM10, ADAM17, ANXA2, APOA1, APP, CCL5, CCR1, CXCL16, CXCL9, CXCR3, F2R, FPR1, FPR2, FYB1, ICAM1, LCP1, LCP2, NR3C1, PECAM1, PLCB3, TLR2
Cell-To-Cell Signaling and Interaction	Response of macrophage cancer cell lines	6.68E-04		-0.113	9	CLIP1, HMOX1, MCL1, NR3C1, PIP5K1A, PRKCD, PTPN6, SIAH1, TLR4
Infectious Diseases	Assembly of virus	6.77E-04		-0.277	4	ANXA2, CNP, IRF8, TSG101
Cellular Assembly and Organization, Cellular Function and Maintenance	Quantity of filopodia-like projection	6.77E-04		1	4	ACTR2, ARPC2, CAPZB, NINJ1
Cell-To-Cell Signaling and Interaction, Cellular Function and Maintenance, Hematological System Development and Function, Inflammatory Response	Phagocytosis of monocytes	6.77E-04			4	CD93, FCGR2A, PF4, SYK
Amino Acid Metabolism, Molecular Transport, Small Molecule Biochemistry	Release of L-cysteine	6.77E-04		1	4	IL1B, IL1RN, TLR2, TLR4
Inflammatory Response	Secretion by mast cells	6.77E-04			4	BTK, LYN, SNAP23, YWHAZ

Cell-To-Cell Signaling and Interaction, Hematological System Development and Function, Immune Cell Trafficking	Adhesion of granulocytes	6.83E-04		-1.951	29	ADAM10, ADAM17, ADGRE2, APOA1, CSF3R, CXCL1, CXCR2, CYBB, F10, FPR2, HCK, ICAM1, IL1B, ITGA4, ITGAX, LCP1, LGALS8, LILRB3, LYN, MGAT5, PECAM1, PF4, PLCB3, PTPN6, S100A9, SWAP70, TLR2, TLR4, TLR5
Cancer, Gastrointestinal Disease, Organismal Injury and Abnormalities	Small intestine tumor	6.85E-04			27	ADAM17, APC, B2M, BCL7A, CTLA4, FCRLA, GSE1, HSP90AA1, HSP90AB1, HSP90B1, HVCN1, IRF8, KBTBD12, KRAS, MCL1, MSH6, NOTCH2, NTRK1, PRKCD, PRKCG, PTEN, PTGS2, RHOA, SSBP2, TET2, TRAF3, U2AF1/U2AF1L5
Cancer, Organismal Injury and Abnormalities, Respiratory Disease	Adenocarcinoma of accessory sinus	6.87E-04			5	APC, HSP90AA1, HSP90AB1, HSP90B1, KRAS
Cellular Development	Lifespan of red blood cells	6.87E-04		-1.342	5	EPO, FOXO3, NCKAP1L, PER2, PRKAA1
Cell Morphology, Cellular Assembly and Organization, Cellular Function and Maintenance, Inflammatory Response	Formation of phagocytic cups	6.87E-04			5	APPL2, HCK, LYN, RAB31, RHOA
Cellular Function and Maintenance	Function of leukocytes	6.96E-04		-0.609	96	ARHGDIB, ARIH2, B2M, B4GALT1, BCL2L11, BTK, CASP8, CCL5, CCR1, CD14, CD84, CD86, CLCN3, CLEC6A, CLEC7A, CNPY3, CREB1, CSF1R, CTLA4, CTSC, CTSZ, CX3CR1, CXCR2, CYBB, DCLRE1C, DMTF1, DOCK8, DUSP5, EFS, F11R, FCAMR, FCGR2A, FOXP3, FPR2, FUT7, FYB1, GAB2, GIMAP4, HCK, HLA-A, HLA-E, HLA-G, HSP90AA1, ICAM1, IFNAR1, IFNGR1, IFNGR2, IL1B, IRF8, LAT2, LCP1, LGMN, LILRB3, LSP1, LYN, MCL1, MERTK, MMP14, MTOR, NEDD9, NFKBIZ, NINJ1, PECAM1, PLCG2, PPM1D, PSAP, PTEN, PTGS2, RAC2, RAP1A, RIPK2, RIPK3, SEMA4A, SH3BP2, SIGLEC9, SIRPA, SKP2, SPHK2, STAT3, STK4, TAGLN2, TBK1, TCF4, TET2, TLR2, TLR4, TLR5, TLR7, TNFRSF1A, TNFSF10, TNFSF14, TRAF3, TRIP10, TYROBP, VDR, WIPF1
Cancer, Hematological Disease, Immunological Disease, Organismal Injury and Abnormalities	B-cell lymphoma	7.08E-04		1.258	174	AMPH, ANKLE2, ANO5, ANXA2, APBA1, APOBEC3A, APOBEC3B, APP, ARHGAP17, ASB10, ASMTL, ATN1, ATRN, ATXN3, B2M, BAZ2B, BBS7, BCL2L11, BCL7A, BECN1, BOD1L1, BTG2, BTK, CARMIL1, CARNS1, CASP8, CAV3, CCNDBP1, CDC23, CFLAR, CGB1/CGB2, CHD4, CMSS1, CNR1, CPSF7, CRNN, CSDE1, CSF1R, CSF3R, CYP2A6 (includes others), DCLRE1C, DMTF1, DNAJB14, DOCK2, DPYD, DYRK1A, ETV6, EWSR1, F11R, FAM131C, FCGR2A, FOXP3, FUS, FYB1, GPRIN1, GPSM2, GRB2, GSE1, HAO2, HCLS1, HDAC7, HDAC9, HECA, HLA-G, HMOX1, HSP90AA1, HSP90AB1, HSP90B1, HVCN1, ICAM1, IDH3A, IFNGR1, IL1B, IRF8, JAK1, KAT6A, KRAS, LCT, let-7, LRRFIP1, LSM3, MAD2L1BP, MAX, MCL1, MDM2, mir-154, mir-28, MPEG1, MS4A1, MTOR, MUC1, MYO5B, MYOF, NACA2, NDUFS1, NETO2, NONO, NOTCH2, NR3C1, NUBP1, NUDT6, NXPE4, PCLO, PDCD4, PDGFRA, PECAM1, PLCG2, PLEKHA7, POLR3B, POTEH (includes others), PPM1D, PPP1R12B, PPP6R3, PSMB8, PSMD1, PSMD2, PSME3, PTEN, PTGS2, PTPRE, PWWP3A, RAB38, RAB4A, RAF1, RBM4, RESF1, RHOA, RICTOR, RPS15, RTTN, SCN9A, SEC14L1, SF3B1, SGK1, SH2B3, SHROOM3, SMARCA2, SORL1, SRPK2, SSBP2, STAT3, STIP1, STXBP6, SWAP70, TAF1, TDRD1, TET2, THBS2, TLR2, TLR7, TNFRSF10C, TNIP1, TRAF3, TRIM55, TRIP12, TRPM6, TTC21B, TUBA1A, TUBA1C, TUBB2A, TXLNA, UBE2F, UNC5C, VDAC1, WASF2, WDFY3, XRCC5, YAE1, YWHAE, YWHAZ, ZMYM3, ZNF615, ZNF700, ZNF714
Cell Death and Survival, Embryonic Development	Cell death of embryonic cell lines	7.23E-04		-1.892	80	ADIPOR1, APOBEC3B, APOL1, APP, ARNT, ATG3, ATG7, ATXN3, BCL2L11, BECN1, BID, BNIP3L, BRCA1, CARD8, CASP8, CDK2AP1, CFLAR, CRADD, CTSB, DDIT3, DDN, DDX17, DFFA, DMTF1, FOXL2, FOXO3, GAPDH, GAS7, HNRNPA1, HSPA5, IFI16, IFNAR1, IGF1, IGF1R, IP6K2, KRAS, L3MBTL2, LYN, MAP3K1, MCL1, MDM2, MEFV, MLKL, MTCH2, MTF1, MTOR, NAMPT, NDEL1, NFATC4, NFE2L2, OPA1, PAK2, PARK7, PDIA3, PITX2, PPM1D, PRKAA1, PRKCD, PTEN, RALBP1, RHOA, RHOB, RIPK3, RPS6KA5, RTN4, SENP2, SH3RF1, SIAH1, SKP2, SOD2, STAT3, STK35, STK4, TBK1, TCF4, TFRC, TLR2, TNFRSF1A, TNFSF10, VDAC1
Hematological System Development and Function,	Quantity of phagocytes	7.23E-04		-1.208	95	ADAM10, ADAM17, ADM2, APOB, ARID4B, ARNTL, B2M, B4GALT1, BCL2L11, BID, CASP8, CCR1, CD86, CFLAR, CLEC4D, CLEC4M, CLEC7A, CLIC4, CSF1R, CSF3R, CTLA4, CTSB, CX3CR1, CXCR2, CYBB, DDIT3, DOCK8, DUSP3, EPO, FCGR2A, FOXP3, FPR1, FPR2, FUT7, GAB2, HCK, HMOX1, HOXA3, ICAM1, IFNAR1, IFNGR1, IGF1R, IL1B, IL1RN, IRF8, KDM5A, KRAS, LHCGR, LITAF, LSP1, LTBR, LYN, MCL1, mir-122, MPP1, MSN, MTOR, NFE2L2, NOTCH2, PDE4B, PILRA, PLP1, PPM1D, PRKCD, PRL, PROK2, PTEN, PTPN6, RAC2,

Inflammatory Response, Tissue Morphology						RHOA, RICTOR, RIOX2, S100A9, SH2B3, SIGLEC9, SIRPA, SOS2, ST3GAL6, STAT3, STEAP4, STK4, SWAP70, TLR2, TLR4, TLR5, TLR7, TNFRSF1A, TNFSF10, TYROBP, VDR, VTCN1, WIPF1, YBX1, ZBTB46, ZEB2
Cancer, Hematological Disease, Immunological Disease, Organismal Injury and Abnormalities	Recurrent plasma cell myeloma	7.43E-04			11	F10, HSP90AA1, HSP90AB1, HSP90B1, mir-154, NR3C1, PSMB8, PSMD1, PSMD2, PTGS2, SLAMF7
Cellular Movement, Immune Cell Trafficking	Migration of lymphatic system cells	7.67E-04	Decreased	-3.994	80	ADAM10, ADAM17, ADGRG3, APBB1IP, APP, BTK, CCL23, CCL5, CCR1, CCR10, CD86, CLEC1B, CTLA4, CUX1, CX3CR1, CXCL1, CXCL16, CXCL9, CXCR2, CXCR3, CYP26B1, DEF6, DEFB103A/DEFB103B, DOCK2, DOCK8, DPYSL2, EFS, EPO, EZR, F11R, FOXP3, FRS2, FUT7, FYB1, HCLS1, HLA-A, HLA-G, HSPD1, ICAM1, IFNAR1, IFNGR1, IGF1, IL1B, ITGA4, JAK1, LCP1, LCP2, LTBR, MAP3K2, MAPKAP1, MSN, MTOR, MYLK, NR3C1, PECAM1, PLCB3, PLCG2, PRKAA1, PTEN, PTGS2, RAC2, RAP1A, RHOA, RICTOR, SCRIB, SERPINB3, SOS2, SPHK2, STAT3, STK4, SWAP70, SYK, THBS2, TLR2, TLR4, TNFRSF1A, TNFSF14, TNFSF4, TNIP1, VTCN1
Cell-To-Cell Signaling and Interaction, Cellular Movement, Hematological System Development and Function, Immune Cell Trafficking	Recruitment of leukocytes	7.67E-04	Decreased	-3.545	69	ADAM10, ADAM17, ALOX5AP, APOA1, APOB, APP, ATG7, B4GALT1, CASP8, CCL23, CCL5, CCR1, CD14, CD93, CLEC7A, CNR1, CSF1R, CTSC, CX3CR1, CXCL1, CXCL16, CXCL6, CXCL9, CXCR2, CXCR3, F13A1, FCGR2A, FPR2, FUT7, GAB2, GC, HCK, HMOX1, HSPA1A/HSPA1B, ICAM1, IFNAR1, IL1B, IL1RN, ITGA4, KRAS, LSP1, LYN, LYZ, MGAT5, NFE2L2, NINJ1, P2RX1, PDE4B, PECAM1, PTEN, RAP1A, RHOA, RHOB, RIPK2, RTN4, SIGLEC9, SOD2, ST3GAL6, STAT3, SWAP70, SYK, THBS2, TLR2, TLR4, TLR5, TLR7, TNFRSF1A, TREML2, VDR
Lipid Metabolism, Small Molecule Biochemistry	Binding of lipid	7.74E-04	Decreased	-2.202	21	ANXA2, APOA1, APOB, APP, CD14, CPT1A, F2R, FKBP5, HSP90AB1, IGF1, MAP4, NCOA4, NPC1L1, NR3C1, PACSIN2, PRKCG, PSAP, STIP1, TLR4, TNFSF10, VDR
Connective Tissue Disorders, Inflammatory Disease, Inflammatory Response, Organismal Injury and Abnormalities, Skeletal and Muscular Disorders	Polyarthritis	7.92E-04		-0.745	39	ADM, CCL5, CCR1, CD86, CDA, DDIT3, F11R, F13A1, FOXP3, FPR2, HDAC7, HLA-G, HNMT, HNRNPA1, HSPA1A/HSPA1B, IFNGR1, IL1B, IL1R2, IL1RN, LTBR, MCL1, NR3C1, NUMB, P2RY13, PECAM1, PTEN, PTGS2, PTMA, PTPRE, RALB, S100A9, SORL1, SPHK2, STAT3, TLR2, TLR4, TNFRSF10C, TNFRSF1A, VTCN1
Cellular Compromise	Respiratory burst	7.93E-04	Decreased	-2.484	19	APP, CD14, CLEC4D, CXCL1, CYBB, FPR1, HCK, ICAM1, IRF8, ITGA4, LILRB3, LYN, NCF2, PF4, SLC11A1, SYK, TLR4, TREM1, TYROBP
Cancer, Hematological Disease, Immunological Disease, Organismal Injury and Abnormalities	Relapsed CD20 positive diffuse large B-cell non-Hodgkin lymphoma	8.10E-04			8	CSF3R, HSP90AA1, HSP90AB1, HSP90B1, MS4A1, NR3C1, PSMD1, PSMD2
Cancer, Hematological Disease, Immunological Disease, Organismal Injury and Abnormalities	Plasma cell neoplasm	8.15E-04			96	AMPH, ANXA2, ANXA5, APBA1, APC, ASXL1, ATRN, B2M, BCL2L11, BECN1, BRCA1, BTK, CASP8, CAVIN2, CCL5, CCNDBP1, CSF1R, CSF3R, CTSC, CXCL1, CXCL6, EPHA8, F10, F11R, FCGR2A, FOXO3, FRMD4B, FUS, FYB1, GPD1, GRB2, HCLS1, HDAC9, HLA-A, HMOX1, HSP90AA1, HSP90AB1, HSP90B1, IFNAR1, IGF1, IGF2R, IL1B, IL1RN, JAK1, KAT6A, KRAS, LCP1, LCP2, MAP4K4, MAX, MCL1, mir-154, MPEG1, MS4A1, MTOR, NDC80, NONO, NOTCH2, NR3C1, NUBP1, PDGFRA, PF4, PLCG2, PPP4R2, PRKAA1, PSMB8, PSMD1, PSMD2, PSME3, PTEN, PTGS2, PTPRE, RAB4A, RICTOR, SF3B1, SLAMF7, SMARCA2, SOD2, SRPK2, STAT3, STIP1, TET2, TFRC, TLR4, TNNI3K, TRAF3, TRIO, TUBA1A, TUBA1C, TUBB2A, U2AF1/U2AF1L5, UBA3, VIM, YWHAE, YWHAZ, ZRSR2

Cellular Development, Cellular Growth and Proliferation, Hematological System Development and Function, Hematopoiesis, Lymphoid Tissue Structure and Development, Tissue Development	Development of phagocytes	8.53E-04		-1.551	56	ADAM10, ADAM17, APP, BTK, CCL23, CDA, CLEC4M, CSF1R, CSF3R, DYRK1A, EPO, GMPR2, HOXA10, HOXA7, IFI16, IFNAR1, IFNGR1, IL1B, IL1RN, IRF8, KRAS, LILRA2, LILRB3, LTBR, LYN, MAPKAP1, MEF2C, MTOR, NFE2L2, NOTCH2, PF4, PLCG2, PPM1D, PRKAA1, PROK2, PTEN, RALB, RBPJ, RFFL, S100A9, SH2B3, SP3, STAT3, TET2, THOC5, TLR2, TLR4, TLR5, TLR7, TMEM178A, TNFRSF1A, TNFSF10, TREM1, VDR, ZBTB46, ZRSR2
Cancer, Organismal Injury and Abnormalities, Reproductive System Disease	Breast cancer	8.59E-04		0.419	425	ABCC2, ACBD3, ADAM15, ADAM17, ADGRA1, AGO2, AIG1, AIPL1, AK9, AKAP12, ALDH3A1, AMPH, ANAPC13, ANKRD42, ANXA3, AOPEP, AP5M1, APC, APOA1, APOBEC3B, APP, ARF4, ARHGAP19, ARHGDIB, ARMC3, ARMCX5-GPRASP2/GPRASP2, ARNT, ARNTL, ASB10, ASXL1, ATG7, ATL3, ATN1, BCL2L11, BECN1, BGN, BLVRA, BNIPL, BOD1L1, BRCA1, BRIP1, BTG2, C17orf80, C18orf25, C1GALT1C1, C1RL, C7orf25, CACNA1E, CAMK2A, CAPZB, CARNS1, CASP8, CCP110, CDC5L, CDH12, CEP128, CEP72, CHCHD5, CHD4, CKMT2, CLK2, CNPY3, COG2, COG5, COL1A2, COL7A1, CPEB1, CPQ, CPT1A, CRY2, CRYBG3, CSDE1, CSF1R, CSF3R, CT45A10/CT45A5, CTBS, CTLA4, CTNND1, CTNND2, CTSB, CTSC, CTSZ, CUX1, CXCL1, CXCL9, CXCR3, CYBB, CYP2A6 (includes others), CYTH4, DDX17, DDX27, DDX5, DGLUCY, DHCR7, DLGAP4, DNAJB6, DNAJC7, DOCK8, DOK5, DPYD, DSE, DYRK1A, EBLN2, ECE1, EFS, EIF1AX, EIF3A, EIF4G3, ELF3, EOGT, EPM2AIP1, ETV6, EZR, F10, F11R, F8, FAM214B, FBLN2, FEZ1, FKBP5, FNBP1L, FOXL2, FOXO3, FOXP3, FRMD4B, FTH1, FUS, FUT7, FZD1, G3BP2, GAL3ST1, GAS7, GASK1B, GBE1, GLE1, GLUL, GLYR1, GPATCH4, H2BC21, H3-3A/H3-3B, HAMP, HBA1/HBA2, HBB, HBP1, HCCS, HCK, HLA-A, HLA-G, HMOX1, HNRNPA1, HNRNPH2, HOTAIR, HOXA3, HSP90AA1, HSP90AB1, HSP90B1, HSPA5, HSPB7, HSPD1, HTATIP2, HVCN1, IDH3A, IER2, IGF1, IGF1R, IL1B, ILF3, ITGAX, JAK1, JAML, JMJD4, JPH4, KAT6A, KDM1B, KDM5A, KIF1C, KIF26B, KLF6, KLHL15, KRAS, L3MBTL3, LAMA5, LAMTOR5, LARP4, LAS1L, let-7, LETM2, LGALS8, LHCGR, LILRA1, LINC00511, LONRF3, LRP2, LRRFIP1, LSP1, LY6K, LY9, LYVE1, MAFF, MAP3K1, MAP4, MAPRE1, MAPRE3, MARF1, MAX, MCCC2, MCL1, MDM2, MED23, MEF2C, mir-101, mir-103, mir-122, mir-154, mir-202, mir-24, mir-26, mir-28, MMP14, MORN5, MRPL15, MS4A1, MT1A, MT1F, MT1X, MTCH2, MTDH, MTHFD2, MTOR, MTTP, MUC1, MYBBP1A, MYH15, MYLK, MYO5B, NABP1, NAMPT, NASP, NCF2, NCKAP1L, NCL, NDE1, NFATC4, NFE2L2, NHSL1, NIN, NLGN3, NLGN4X, NONO, NOTCH2, NR3C1, NTRK1, NUMB, NUP93, OPA1, OR2A14, OR4D10, OR5AC2, OSBPL11, OTUD3, PAK2, PCOLCE, PDGFRA, PDLIM5, PDS5B, PECAM1, PEX19, PF4, PILRA, PIN4, PITX2, PLXDC2, POTEH (includes others), PPM1D, PPP1R12B, PQBP1, PRC1, PRKCD, PRL, PRR12, PRSS55, PSMD12, PSMD4, PTEN, PTGS2, PTPRE, PUDP, PWWP3A, RAB31, RAB3GAP2, RAD51C, RAF1, RALBP1, RALGPS1, RAP1A, RASEF, RBMXL3, RFPL2, RFX3, RGS2, RHBG, RHOA, RHOB, RICTOR, RIN2, RPGRIP1, RPL4, RPL5, RTCB, RTN4, RTTN, S100A14, S100A9, SBF2, SCRIB, SEC14L1, SEC61A2, SENP2, SETDB1, SF3B1, SKP2, SLC22A18, SLC22A4, SLC24A4, SLC25A32, SLC31A1, SLC43A3, SLC4A2, SLC6A6, SLC8A1, SLITRK6, SMTN, SOD2, SORL1, SP100, SP3, SPAG9, SPATA5, SPEF2, SPHK2, SPOP, SRPK1, STAT3, STX3, SUSD6, SYK, SYT17, SZT2, TAF1, TAGLN2, TBC1D12, TBC1D9, TCAIM, TCF4, TCP1, TDGF1, TERF2IP, TET2, THRAP3, TLR4, TLR5, TM2D2, TM7SF3, TMEM43, TMTC2, TNFRSF1A, TNFSF10, TNNC1, TOR1B, TRIM46, TRIM5, TRIM65, TRIO, TRIP10, TRIP12, TRMT9B, TRPM6, TSG101, TSHZ3, TTI2, TUBA1A, TUBA1B, TUBA1C, TUBB2A, U2AF1/U2AF1L5, UBAP2L, UBE4B, USP19, USP32, UTP4, VCAN, VCPIP1, VDAC2, VDR, VTI1B, WASF2, WDFY3, WNK1, WNK3, WSB1, XPNPEP3, YPEL5, YWHAZ, ZAN, ZBTB21, ZMPSTE24, ZMYM3, ZNF10, ZNF165, ZNF217, ZNF229, ZNF235, ZNF24, ZNF3, ZNF398, ZNF45, ZNF461, ZNF516, ZNF525, ZNF570, ZNF615, ZNF677, ZNF711, ZNFX1, ZSCAN2
Cell-To-Cell Signaling and Interaction, Cellular Movement	Recruitment of cells	8.83E-04	Decreased	-4.029	76	ADAM10, ADAM17, ALOX5AP, APOA1, APOB, APP, ATG7, B4GALT1, CASP8, CCL23, CCL5, CCR1, CD14, CD93, CLEC1B, CLEC7A, CNR1, CSF1R, CTSC, CX3CR1, CXCL1, CXCL16, CXCL6, CXCL9, CXCR2, CXCR3, ENTPD1, F13A1, FCGR2A, FPR2, FUT7, GAB2, GC, GLCE, HCK, HMOX1, HSPA1A/HSPA1B, ICAM1, IFNAR1, IL1B, IL1RN, ITGA4, KRAS, LSP1, LYN, LYZ, MGAT5, MTOR, NFE2L2, NINJ1, P2RX1, PDE4B, PECAM1, PTEN, PTGS2, RAP1A, RHOA, RHOB, RIPK2, RTN4, SIGLEC9, SLC11A1, SOD2, ST3GAL6, STAT3, SWAP70, SYK, THBS2, TLR2, TLR4, TLR5, TLR7, TNFRSF1A, TREML2, VDR, VIM
Hematological System Development and Function	Coagulation	8.84E-04		-1.217	62	ANXA2, ANXA5, APLP2, APP, ARNTL, C1GALT1C1, C4BPB, CALU, CAPZA1, CAPZB, CARMIL1, CCL5, CLEC1B, COL1A2, CYP4F2, DOCK8, EHD3, ENTPD1, EPO, F10, F13A1, F2R, F8, FCGR2A, GATA5, H3-3A/H3-3B, H3C1, H3C13, HBB, JMJD1C, LCP2, LYN, MAFF, MERTK, P2RX1, PDGFRA, PECAM1, PF4, PLCB3, PLCG2, PLEK, PRKAR1A, PRKCD, PRKCG, PRKG1, PTEN, PTGS2, PTPN6, RAB27A, RAB5A, RAD51C, RAF1, SGK1, SH2B3, SYK, THBS2, TLR2, TLR4, TREM1, VCAN, YWHAZ, ZFPM2
Cell-To-Cell Signaling and Interaction,	Interaction of T lymphocytes	9.07E-04	Decreased	-3.716	34	APBB1IP, APOA1, ATRN, CCL5, CCR1, CD86, CTLA4, CXCL9, CXCR3, DOCK2, EZR, FUT7, FYB1, ICAM1, IFNGR1, IL1B, ITGA4, JAK1, LCP2, LTBR, MAP3K2, MSN, NR3C1, PECAM1, PRL, RAC2, RAP1A, RHOA, RICTOR, STK4, THBS2, TLR2, TLR4, TNFSF14

Hematological System Development and Function						
Cellular Development, Cellular Growth and Proliferation, Embryonic Development, Hematological System Development and Function, Hematopoiesis, Lymphoid Tissue Structure and Development, Organ Development, Organismal Development, Tissue Development	Lymphopoiesis	9.16E-04	Decreased	-4.657	134	ADAM10, ADAM17, ADGRG3, APC, ARNTL, ASXL1, B2M, BCL2L11, BRCA1, BTK, CASP8, CCL5, CD14, CD86, CFLAR, CHD4, CLEC6A, CREB1, CTLA4, CXCL1, CXCR2, CXCR3, CYP26B1, DCLRE1C, DEF6, DNAJA2, DOCK2, DOCK8, DUSP5, ELF1, ELF3, ENTPD1, EPHB1, EPO, EZR, FCAMR, FCGR2A, FOXO3, FOXP3, FUT7, FYB1, GIMAP4, GRB2, HDAC7, HDAC9, HLA-A, HLA-G, HSP90AA1, HSP90B1, HSPD1, ICAM1, IFNAR1, IFNGR1, IFNGR2, IGF1, IGF1R, IGF2R, IL1B, IL1RN, IRF8, ITGA4, ITGB8, JAK1, KRAS, LAT2, LCP1, LCP2, let-7, LGALS8, LSP1, LTBR, LY9, LYN, MAPKAP1, MBP, MCL1, MDM2, MEF2C, MERTK, mir-24, MMP14, MPZL2, MS4A1, MSN, MTOR, NCKAP1L, NFKBIZ, NMT1, NOTCH2, NTRK1, PF4, PHLPP1, PLCG2, PLP1, PRKAA1, PRKCD, PRL, PSAP, PSMB8, PTEN, PTGS2, PTPN6, RAD52, RAF1, RBPJ, RGCC, RHOA, RICTOR, RIPK2, RIPK3, SEMA4A, SKP2, SP3, SPINK5, STAT3, SWAP70, SYK, TCF4, TDP2, THEMIS2, TLR2, TLR4, TLR5, TLR7, TNFRSF1A, TNFSF10, TNFSF4, TRAF3, TYROBP, USP15, USP4, WIPF1, XRCC5, ZEB2
Cellular Development, Cellular Growth and Proliferation, Embryonic Development, Hematological System Development and Function, Hematopoiesis, Humoral Immune Response, Lymphoid Tissue Structure and Development, Organ Development, Organismal Development, Tissue Development	Development of marginal-zone B lymphocytes	9.20E-04			7	ADAM10, ADAM17, BTK, DOCK8, LYN, NOTCH2, RICTOR
Cell Death and Survival, Skeletal and Muscular System Development and Function	Cell viability of vascular smooth muscle cells	9.23E-04		-1.664	6	APP, FOXO3, HMOX1, IGF1, IGF1R, PDGFRA
Nervous System Development and Function	Protection of cortical neurons	9.23E-04		0.975	6	APP, CNR1, DDIT3, NFATC4, PHLPP1, WDFY3

Molecular Transport, Protein Synthesis, Protein Trafficking	Localization of anti-DNA antibody	9.23E-04		1.109	6	ATG7, BECN1, CYBB, MERTK, STAT3, TNIP1
Infectious Diseases	Infection of cells	9.29E-04	Decreased	-8.87	153	ACP3, ACTR2, ADAM10, ALG14, ALKBH3, ALKBH8, AMPH, ANXA2, APOBEC3B, APP, ARF1, ARPC5, ATG7, ATOX1, ATP6AP2, B2M, BMP2K, BRCA1, BRINP2, BTG2, CALCOCO1, CAMK1D, CARD16, CCL5, CCR1, CCT2, CD86, CD93, CHORDC1, CHST1, CLEC4M, CLIP1, COG2, COG5, CTSB, CTSZ, CYB5B, CYBB, DAZAP2, DCP1A, DDX23, DDX3X, DEFB103A/DEFB103B, DLGAP4, DNAJA2, EIF3A, EIF3G, EIF3I, ELOA, ERCC5, ETV3, F10, F2R, FAM228B, FGD6, FPR1, FRS2, FUT7, GATAD2A, H3-3A/H3-3B, HCK, HMCN2, HNRNPH1, HSP90B1, HSPA5, ICAM1, IFNAR1, IGF2R, INTS6, ITGA4, JAK1, KAT6A, KDM7A, KMT5B, LCP2, LEFTY1, let-7, LIMK2, LSM3, MAP4, MAPRE1, MDM2, MED31, MERTK, MGAT5, MT1X, MYO1F, MYOF, NACA2, NLGN3, NMT1, NPSR1-AS1, NUP160, NUP50, NUP62, OTUD3, PCK1, PDE8A, PDGFRA, PDIA3, PDZD8, PHF12, PIP5K1A, PRKAA1, PRPF38A, PRPF6, PSMD12, PSMD4, PURA, RAB5A, RAB8A, RALB, RBM25, RBM5, RHOB, RPL18, RPL5, RTN3, SBF2, SEC14L1, SENP5, SESTD1, SF3B1, SLC31A1, SLU7, SNRPD3, SP110, SPAST, SSR1, STAU1, STIP1, TAGLN2, TBK1, TFRC, TLR2, TLR4, TNPO3, TRIM5, TRIM55, TRMT5, TRPT1, TSG101, UBE2B, UBE2L3, UTP11, VDR, WASF2, WNK1, YBX1, ZMPSTE24, ZNF148, ZNF417/ZNF587, ZNF720
Cancer, Hematological Disease, Immunological Disease, Organismal Injury and Abnormalities	Refractory CD20 positive aggressive non-Hodgkin lymphoma	9.43E-04			9	BTK, CSF3R, HSP90AA1, HSP90AB1, HSP90B1, MS4A1, NR3C1, PSMD1, PSMD2
Cell-To-Cell Signaling and Interaction, Cellular Movement, Hematological System Development and Function, Immune Cell Trafficking, Inflammatory Response	Recruitment of neutrophils	9.55E-04	Decreased	-2.55	41	ADAM10, ADAM17, ALOX5AP, APOA1, ATG7, B4GALT1, CCL5, CCR1, CD14, CTSC, CX3CR1, CXCL1, CXCL6, CXCR2, FCGR2A, FPR2, FUT7, GC, HCK, HSPA1A/HSPA1B, ICAM1, IFNAR1, IL1B, IL1RN, LSP1, LYN, LYZ, MGAT5, P2RX1, PDE4B, PTEN, RIPK2, RTN4, SIGLEC9, SOD2, ST3GAL6, TLR2, TLR4, TLR5, TNFRSF1A, TREML2
Cellular Movement, Hematological System Development and Function, Immune Cell Trafficking, Inflammatory Response	Influx of neutrophils	9.56E-04	Decreased	-2.2	14	ADAM17, CTSC, CX3CR1, CXCL1, CXCL6, CXCR2, CYBB, FCGR2A, IFNAR1, IL1B, PTEN, TLR2, TLR4, TNFRSF1A
Molecular Transport	Export of metal	9.56E-04		-1.425	14	ACO1, APOL1, APP, ATOX1, ATP7B, F2R, FTH1, HAMP, HMOX1, PLCB3, SGK1, SLC11A1, SLC8A3, YWHAE
Cancer, Connective Tissue Disorders, Ophthalmic Disease, Organismal Injury and Abnormalities, Skeletal and Muscular Disorders	Retinoblastoma	9.56E-04			14	AKAP12, APC, CSF3R, CYP51A1, EWSR1, HSP90AA1, HSP90AB1, HSP90B1, KRAS, MAX, RAF1, TUBA1A, TUBA1C, TUBB2A
Cellular Assembly and Organization, Cellular Function and Maintenance	Organization of cytoplasm	9.62E-04	Decreased	-6.574	313	ABITRAM, ACTG1, ACTN1, ACTR2, ADAM10, ADM, AKAP12, ALKBH1, ALS2, ANGPTL4, AP1G1, APBA1, APBB1IP, APC, APC2, APLP2, APP, APPL2, ARF1, ARHGAP17, ARHGAP25, ARHGEF25, ARHGEF9, ARPC2, ARPC5, ASB7, ATAT1, ATG7, ATL3, ATRN, ATXN3, AURKB, BASP1, BCAS3, BECN1, BLZF1, BMP2K, BRCA1, BRWD3, BTBD3, BTG2, BTK, CALML3, CALU, CAMK1D, CAMK1G, CAMK2A, CAMSAP2, CAP1, CAPZB, CARMIL1, CAV3, CBY1, CCDC47, CCDC88A, CCL5, CCP110, CDKL5, CELSR3, CEP72, CIBAR1, CLASP1, CLEC1B, CLIP1,

						CNP, CNR1, COG2, CORO1C, CREB1, CRKL, CSF1R, CTLA4, CTNND1, CTNND2, CUX1, CX3CR1, CXCL1, CXCL9, CXCR2, CXCR3, CYBB, DKK3, DLG3, DNAJB6, DNM3, DOCK2, DPYSL2, DRG1, DYNC1LI1, DYNLL1, DYRK1A, EIF4G3, EMC10, EPB41L3, EPB41L5, EPHA8, EPHB1, EPO, ERC2, EVI5L, EZR, F11R, F13A1, F2R, FARP2, FCGR2A, FEZ1, FGD4, FNBP1L, GAB1, GAPDH, GAS7, GMFG, GOLGB1, GPRIN1, GPSM2, HBP1, HCK, HOXA4, HSP90AA1, HSP90AB1, ICAM1, IFT88, IGF1, IGF1R, IL1B, ITGA4, ITGB8, KCNJ2, KIDINS220, KIF13B, KIF1C, KLF7, KNSTRN, KRAS, KRT6C, LAMA5, LARP4, LASP1, LCP1, LCP2, LHCGR, LIMK2, LRP2, LRP8, LRPAP1, LSP1, LYN, MAP3K1, MAP4, MAPRE1, MAPRE3, MAST3, MBP, MERTK, MGAT5, mir-138, mir-26, MPP1, MSN, MTFR2, MTOR, MYLK, MYO1F, MYO5A, MYO5B, NCF2, NCKAP1L, NDC80, NDE1, NDEL1, NEDD1, NEDD9, NFATC4, NIN, NINJ1, NLGN3, NSFL1C, NTRK1, NUAK2, NUMB, NUP160, NUP62, OPA1, P2RX1, PACSIN2, PAK2, PARK7, PCLO, PDGFRA, PDIA3, PDZD8, PEX19, PF4, PHACTR1, PHETA2, PIP5K1A, PITPNA, PITPNM1, PJA2, PLCG2, PLEK, PLXNA4, POU4F2, PQBP1, PRC1, PRKAA1, PRKCD, PRKCG, PRKG1, PTEN, PTF1A, PTGS2, PTPRE, RAB11A, RAB21, RAB22A, RAB31, RAB38, RAB5A, RAB8A, RAC2, RAF1, RALB, RALBP1, RAP1A, RAPGEF2, RFX3, RGMA, RHOA, RHOB, RICTOR, RIT1, RNF6, RPL4, RTN3, RTN4, RUFY3, S100A9, SEMA3G, SEMA4A, SEMA4F, SGK1, SHROOM3, SIAH1, SIRPA, SIRPB1, SLC22A5, SLITRK6, SMAD1, SNAP29, SNAP91, SNAPIN, SOD2, SP1, SPAST, SPATA13, SPTBN4, SPTSSB, SRGAP3, SSH1, SSH3, SSX2IP, STAT3, STIP1, STK24, STK35, STK38L, STX3, SURF4, SWAP70, SYK, TACC1, TBC1D30, TBK1, TESK2, TJP1, TLR4, TLR7, TMEM107, TMOD3, TNFRSF1A, TNFSF10, TOR1AIP2, TOR1B, TPM3, TRAF3IP1, TRIM46, TRIO, TRIOBP, TRIP10, TTC26, TUBGCP3, TXNRD1, TYROBP, UBAP2L, UBE4B, VAMP4, VCPIP1, VIM, VPS35, VPS4B, VTCN1, WASF2, WASF3, WDR19, WDR60, WIPF1, WWTR1, YBX1, ZEB2, ZMYM3, ZRANB1
Cell Morphology, Cellular Movement, Hematological System Development and Function, Immune Cell Trafficking, Inflammatory Response	Cell spreading of phagocytes	9.76E-04	Decreased	-3.132	11	ATRN, CLEC1B, HCK, ICAM1, LYN, PECAM1, PLCG2, RHOA, RHOB, SIRPA, SYK
Cell Death and Survival	Cell death of brain	9.83E-04		-0.305	63	APP, ATG7, ATXN3, BCL2L11, BECN1, BID, CAMK2A, CASP8, CDC25C, CFLAR, CXCL1, DDIT3, EPO, FAIM2, FOXO3, FUS, GAPDH, GCLC, HDAC9, HSPA5, HSPD1, IGF1, IGF1R, IL1B, IL1RN, KLF6, LILRB3, LRPAP1, MAP3K1, MCL1, MEF2C, mir-26, MTOR, NFATC4, NFE2L2, NTRK1, PARK7, PITX2, PRKCD, PRKCG, PTEN, PTGS2, RHOA, RIT1, RPS6KA5, SGK1, SHC3, SKP2, SP1, SP3, SRPK2, STIP1, STK4, TCP1, TLR2, TLR4, TLR7, TNFRSF1A, TNFSF10, TRIP10, UBE2L3, WNK3, YWHAB
Cell-mediated Immune Response, Cellular Movement, Hematological System Development and Function, Immune Cell Trafficking, Lymphoid Tissue Structure and Development	Homing of T lymphocytes	9.93E-04	Decreased	-2.393	27	ADAM10, ADAM17, CCL23, CCL5, CCR1, CUX1, CXCL16, CXCL9, CXCR2, CXCR3, DEFB103A/DEFB103B, FOXP3, FYB1, HSPD1, ITGA4, JAK1, LCP1, LTBR, MAPKAP1, NR3C1, PTEN, RAC2, STAT3, STK4, THBS2, TLR4, TNFSF14
Molecular Transport, RNA Trafficking	Transport of RNA	9.93E-04			25	CASC3, CPEB1, CPSF4, DDX39A, DDX3X, DHX38, FUS, HNRNPA2B1, MAGOHB, NCBP1, NUP160, NUP50, NUP58, NUP62, NUP93, SEC13, SLU7, SRSF1, SRSF3, SRSF4, SRSF5, THOC5, U2AF1/U2AF1L5, WDR33, YBX1
Cancer, Hematological Disease, Immunological Disease, Organismal Injury and Abnormalities	Mature B cell malignant tumor	1.00E-03		-0.246	193	AMPH, ANKLE2, ANO5, ANXA2, ANXA5, APBA1, APC, APOBEC3A, APOBEC3B, APP, ARHGAP17, ASB10, ASMTL, ASXL1, ATN1, ATRN, ATXN3, B2M, BAZ2B, BBS7, BCL2L11, BCL7A, BECN1, BOD1L1, BRCA1, BTK, CARMIL1, CARNS1, CASP8, CAV3, CAVIN2, CCL5, CCNDBP1, CDC23, CFLAR, CGB1/CGB2, CHD4, CMSS1, CNR1, CPSF7, CRNN, CSDE1, CSF1R, CSF3R, CTSC, CXCL1, CXCL6, CYP2A6 (includes others), DMTF1, DNAJB14, DOCK2, DPYD, DYRK1A, EPHA8, ETV6, EWSR1, F10, F11R, FAM131C, FCGR2A, FOXO3, FRMD4B, FUS, FYB1, GPD1, GPRIN1, GPSM2, GRB2, GSE1, HAO2, HCLS1, HDAC9, HECA, HLA-A, HLA-G, HMOX1, HSP90AA1, HSP90AB1, HSP90B1, HVCN1, IDH3A, IFNAR1, IGF1, IGF2R, IL1B, IL1RN, IRF8, JAK1, KAT6A, KRAS, LCP1, LCP2, LCT, let-7, LRRFIP1, LSM3, MAP4K4, MAX, MCL1, MDM2, mir-154, mir-28, MPEG1, MS4A1, MTOR, MYO5B, MYOF, NACA2, NDC80, NDUFS1, NETO2, NONO, NOTCH2, NR3C1, NUBP1, NUDT6, NXPE4, PCLO, PDGFRA, PECAM1, PF4, PLCG2, PLEKHA7, POLR3B, POTEH (includes others), PPM1D, PPP1R12B, PPP4R2, PPP6R3, PRKAA1, PSMB8, PSMD1, PSMD2, PSME3, PTEN, PTGS2, PTPRE, PWWP3A, RAB38, RAB4A, RAF1, RBM4, RESF1, RHOA, RICTOR, RPS15, RTTN, SCN9A, SEC14L1, SF3B1, SHROOM3, SLAMF7, SMARCA2, SOD2, SORL1,

						SRPK2, STAT3, STIP1, STXBP6, SWAP70, TDRD1, TET2, TFRC, THBS2, TLR2, TLR4, TLR7, TNFRSF10C, TNIP1, TNNI3K, TRAF3, TRIM55, TRIO, TRIP12, TRPM6, TTC21B, TUBA1A, TUBA1C, TUBB2A, U2AF1/U2AF1L5, UBA3, UBE2F, UNC5C, VDAC1, VIM, YAE1, YWHAE, YWHAZ, ZMYM3, ZNF615, ZNF700, ZNF714, ZRSR2
Cell Death and Survival, Neurological Disease, Organismal Injury and Abnormalities	Apoptosis of cortical neurons	1.01E-03		-0.782	22	APP, BCL2L11, BECN1, DDIT3, EPO, GCLC, HSPA5, IGF1, LRPAP1, MAP3K1, MCL1, MEF2C, mir-26, NFATC4, PTGS2, RHOA, SHC3, SRPK2, TLR7, TNFRSF1A, WNK3, YWHAB
Hematological System Development and Function, Humoral Immune Response, Lymphoid Tissue Structure and Development, Tissue Morphology	Quantity of marginal-zone B lymphocytes	1.01E-03	Decreased	-2.851	22	ADGRG3, APBB1IP, ARHGDIB, BCL2L11, CASP8, DOCK2, DOCK8, HVCN1, IFNAR1, IRF8, KRAS, LYN, MTOR, NEDD9, NOTCH2, PRKCD, SH3BP2, STK4, TET2, TLR2, TLR4, WIPF1
Hematological System Development and Function, Lymphoid Tissue Structure and Development, Tissue Morphology	Morphology of lymphoid tissue	1.02E-03			109	ADAM17, ADGRG3, ANGPTL4, ARHGDIB, ARID4B, ARL6IP5, ARNTL, ASXL1, ATP6AP2, B4GALT1, BCL2L11, BECN1, BNIP3L, BRCA1, BTK, CASP8, CD84, CD86, CFLAR, CLEC1B, CLEC4D, CLEC4M, CSF1R, CTLA4, CUX1, CXCR2, CYP51A1, DCLRE1C, DOCK2, EPO, ERCC5, EWSR1, EZR, F2R, FCAMR, FOXO3, FOXP3, FPR2, FUT7, GIT2, HCK, HMOX1, HOXA3, HOXA7, HSP90B1, IFNAR1, IFNGR1, IGF1, IL1RN, IRF8, JAK1, KAT6A, KRAS, LCP1, LCP2, LGMN, LHCGR, LTBR, LYN, LYPLA2, MCL1, MDM2, MERTK, MTOR, NFE2L2, NFKBIZ, NR3C1, NUMB, PITX2, PPM1D, PRKAA1, PRKCD, PRKG1, PSAP, PTEN, PTF1A, PTGS2, PTPN6, PURA, RAB27A, RAMP2, RASSF2, RIPK3, SH2B3, SH3BP2, SNX27, SPHK2, ST6GALNAC2, STAT3, STK4, SYK, TDP2, TET2, TLR4, TLR5, TLR7, TNFRSF1A, TNFSF14, TNIP1, TRAF3, TRIP10, TYROBP, VDR, VTCN1, WIPF1, XRCC5, ZBTB46, ZEB2, ZMPSTE24
Cell Death and Survival	Cell death of macrophage cancer cell lines	1.03E-03		-1.653	16	ANTXR2, BCL2L11, BNIP3L, CASP8, CFLAR, DDIT3, FOXO3, HSP90AB1, ITGA4, MCL1, MVP, PRKCD, RIPK3, TLR4, TNFRSF1A, TREM1
Post-Translational Modification	Autophosphorylation of protein	1.03E-03			36	AURKB, BTK, CAMK2A, CDKL5, CLK2, CLK3, CSF1R, DYRK1A, EEF2K, EPHA8, EPHB1, GRK7, HCK, HTATIP2, IGF1R, LMTK2, LYN, MAK, MAP3K1, MTOR, MYLK2, NLK, NTRK1, PAK2, PDGFRA, PEAK1, PRKCD, PRKCG, RIOK2, RIPK3, STK24, STK4, SYK, TAF1, WNK1, WNK3
Cell Morphology	Polarization of cells	1.04E-03	Decreased	-2.822	42	APC, AQP9, CCL5, CLIP1, CTLA4, CXCL9, CYBB, CYP26B1, DOCK2, DPYSL2, GAB1, HLA-G, HOXA3, HSBP1, IL1B, IL1RN, ITGA4, KIF26B, KRAS, LAMA5, LCP1, let-7, LSP1, MSN, MYLK, NAMPT, PRKAA1, PRKG1, PTEN, RAP1A, RBPJ, RHOA, RICTOR, SCRIB, STAT3, STK4, SVIL, SWAP70, TLR2, TLR4, WIPF1, WWTR1
Cell-To-Cell Signaling and Interaction, Nervous System Development and Function	Long-term potentiation of brain	1.04E-03		0.2	31	APP, ARHGEF9, ATXN3, B2M, CAMK2A, CCDC88A, CNR1, CREB1, CYBB, IGF2R, IL1B, IL1RN, ITM2B, JPH4, KIDINS220, KRAS, LGMN, LILRB3, LRP8, LRPAP1, NLGN3, NPTN, PJA2, PRKAR1A, PSAP, RHOB, RTN4, ST8SIA4, STIP1, TCF4, TLR4
Cardiovascular Disease, Cell Death and Survival, Organismal Injury and Abnormalities, Skeletal and Muscular Disorders	Apoptosis of cardiomyocytes	1.04E-03		1.553	41	ACSL1, ADM, APOA1, BACH1, BCL2L11, BECN1, BNIP3L, CASP8, CAV3, CYBB, CYP2J2, EPO, FOXO3, GAPDH, HMOX1, HSPD1, IGF1, IL1B, IL1RN, KRAS, MAP3K1, MCL1, MDM2, mir-133, mir-154, mir-24, MT1A, NAMPT, PARK7, PRKAA1, PRKCD, PTEN, RAF1, RHOA, RTN4, SLC8A1, SOD2, STAT3, STK4, TLR4, UBE4B
Cell Cycle	Cell cycle progression of carcinoma cell lines	1.07E-03		0.64	13	DNAJB4, EMSLR, FOXO3, HMOX1, IFI16, MDM2, mir-138, NASP, PRKCD, RAF1, SMARCA2, TCF4, YWHAE
Cardiovascular Disease,	Formation of thrombus	1.08E-03		-1.223	16	ANXA2, CXCR2, EPO, F8, FCGR2A, LCP2, LRP8, P2RX1, PF4, PLCB3, RAP1A, RHOA, SGK1, SYK, TNFRSF1A, VDR

Hematological Disease, Organismal Injury and Abnormalities						
Cancer, Organismal Injury and Abnormalities, Reproductive System Disease	Mammary tumor	1.08E-03		-0.279	445	ABCC2, ACBD3, ADAM15, ADAM17, ADGRA1, AGO2, AIG1, AIPL1, AK9, AKAP12, ALDH3A1, AMPH, ANAPC13, ANKRD42, ANXA3, AOPEP, AP5M1, APC, APOA1, APOBEC3B, APP, ARF4, ARHGAP19, ARHGDIB, ARMC3, ARMCX5-GPRASP2/GPRASP2, ARNT, ARNTL, ARRDC3, ASB10, ASXL1, ATG7, ATL3, ATN1, BCL2L11, BECN1, BGN, BLVRA, BNIPL, BOD1L1, BRCA1, BRIP1, BTG2, BTK, C17orf80, C18orf25, C1GALT1C1, C1RL, C7orf25, CACNA1E, CAMK2A, CAPZB, CARNS1, CASP8, CCDC88A, CCL5, CCP110, CDC5L, CDH12, CEP128, CEP72, CGB3 (includes others), CHCHD5, CHD4, CKMT2, CLK2, CNPY3, COG2, COG5, COL1A2, COL7A1, CPEB1, CPQ, CPT1A, CRY2, CRYBG3, CSDE1, CSF1R, CSF3R, CT45A10/CT45A5, CTBS, CTLA4, CTNND1, CTNND2, CTSB, CTSC, CTSZ, CUX1, CXCL1, CXCL9, CXCR3, CYBB, CYP2A6 (includes others), CYTH4, DAB2, DDX17, DDX27, DDX5, DGLUCY, DHCR7, DLGAP4, DMTF1, DNAJB6, DNAJC7, DOCK8, DOK5, DPYD, DSE, DYRK1A, EBLN2, ECE1, EFS, EIF1AX, EIF3A, EIF4G3, ELF3, EOGT, EPM2AIP1, EPO, ETV6, EZR, F10, F11R, F2R, F8, FAM214B, FBLN2, FEZ1, FKBP5, FNBP1L, FOXL2, FOXO3, FOXP3, FRMD4B, FTH1, FUS, FUT7, FZD1, G3BP2, GAL3ST1, GAS7, GASK1B, GBE1, GLE1, GLUL, GLYR1, GPATCH4, H2BC21, H3-3A/H3-3B, HAMP, HBA1/HBA2, HBB, HBP1, HCCS, HCK, HDLBP, HLA-A, HLA-G, HMOX1, HNRNPA1, HNRNPH2, HOTAIR, HOXA3, HSP90AA1, HSP90AB1, HSP90B1, HSPA5, HSPB7, HSPD1, HTATIP2, HVCN1, IDH3A, IER2, IGF1, IGF1R, IL1B, ILF3, ITGAX, JAK1, JAML, JMJD1C, JMJD4, JPH4, KAT6A, KDM1B, KDM5A, KIF1C, KIF26B, KLF6, KLHL15, KLHL20, KRAS, L3MBTL3, LAMA5, LAMTOR5, LARP4, LAS1L, let-7, LETM2, LGALS8, LHCGR, LILRA1, LINC00511, LONRF3, LRP2, LRRFIP1, LSP1, LY6K, LY9, LYN, LYVE1, MAFF, MAP3K1, MAP4, MAPRE1, MAPRE3, MARF1, MAX, MCCC2, MCL1, MDM2, MED23, MEF2C, mir-101, mir-103, mir-122, mir-1260a, mir-138, mir-154, mir-202, mir-24, mir-26, mir-28, mir-551, MMP14, MORN5, MRPL15, MS4A1, MT1A, MT1F, MT1X, MTCH2, MTDH, MTHFD2, MTOR, MTTP, MUC1, MYBBP1A, MYH15, MYLK, MYO5B, NABP1, NAMPT, NASP, NCF2, NCKAP1L, NCL, NDE1, NEDD9, NFATC4, NFE2L2, NHSL1, NIN, NLGN3, NLGN4X, NONO, NOTCH2, NR3C1, NTRK1, NUMB, NUP93, OPA1, OR2A14, OR4D10, OR5AC2, OSBPL11, OTUD3, PAK2, PCOLCE, PDGFRA, PDLIM5, PDS5B, PECAM1, PEX19, PF4, PILRA, PIN4, PITX2, PLXDC2, POTEH (includes others), PPM1D, PPP1CB, PPP1R12B, PQBP1, PRC1, PRKCD, PRL, PRR12, PRSS55, PSMD10, PSMD12, PSMD4, PTEN, PTGS2, PTPRE, PUDP, PWWP3A, RAB31, RAB3GAP2, RAD51C, RAF1, RALBP1, RALGPS1, RAP1A, RASEF, RBMXL3, RFPL2, RFX3, RGS2, RHBG, RHOA, RHOB, RICTOR, RIN2, RPGRIP1, RPL4, RPL5, RTCB, RTN4, RTTN, S100A14, S100A9, SBF2, SCRIB, SEC14L1, SEC61A2, SENP2, SETDB1, SF3B1, SKP2, SLC22A18, SLC22A4, SLC24A4, SLC25A32, SLC31A1, SLC43A3, SLC4A2, SLC6A6, SLC8A1, SLITRK6, SMTN, SOD2, SORL1, SP100, SP3, SPAG9, SPATA5, SPEF2, SPHK2, SPOP, SRPK1, STAT3, STIP1, STX3, SUSD6, SYK, SYT17, SZT2, TAF1, TAGLN2, TBC1D12, TBC1D9, TCAIM, TCF4, TCP1, TDGF1, TERF2IP, TET2, THRAP3, TLR4, TLR5, TM2D2, TM7SF3, TMEM43, TMTC2, TNFRSF1A, TNFSF10, TNNC1, TOR1B, TRIM46, TRIM5, TRIM65, TRIO, TRIP10, TRIP12, TRMT9B, TRPM6, TSG101, TSHZ3, TTI2, TUBA1A, TUBA1B, TUBA1C, TUBB2A, U2AF1/U2AF1L5, UBAP2L, UBE4B, USP19, USP32, UTP4, VCAN, VCPIP1, VDAC2, VDR, VTI1B, WASF2, WDFY3, WNK1, WNK3, WSB1, XPNPEP3, YPEL5, YWHAZ, ZAN, ZBTB21, ZMPSTE24, ZMYM3, ZNF10, ZNF165, ZNF217, ZNF229, ZNF235, ZNF24, ZNF3, ZNF398, ZNF45, ZNF461, ZNF516, ZNF525, ZNF570, ZNF615, ZNF677, ZNF711, ZNFX1, ZSCAN2
Cell Death and Survival	Cell death of lymphoma cell lines	1.09E-03		1.486	46	ADAM17, ANXA2, ARNT, ATG3, ATG7, BCL2L11, BTG2, BTK, CARD8, CASP8, CEACAM3, CFLAR, DDIT3, EZR, FTH1, HCK, HNRNPA1, IGF1R, IGFBP4, IRF8, ITGA4, JAK1, LSP1, LYN, MAX, MCL1, MS4A1, MTOR, MUC1, MXD1, NCL, NR3C1, PDE4B, PLCG2, PRL, PTPN6, RAET1E, RIPK3, SMAD1, SOD2, STAT3, SYK, TNFRSF1A, TNFSF10, TRAF3, YWHAZ
Infectious Diseases	Replication of HIV-1	1.09E-03		-1.803	29	ADAM10, ANXA5, APOBEC3B, ARHGDIB, ARNTL, ATG7, BECN1, CCL5, CCNK, CFLAR, DDX5, DYRK1A, FAS-AS1, GALC, HCK, IL1B, MED30, NUP62, P2RX1, PACSIN2, PDE8A, RAF1, S100A9, SNAPIN, STAT3, TLR2, TNFSF10, TRIM5, TSG101
Cellular Movement, Hematological System Development and Function, Immune Cell Trafficking, Lymphoid Tissue Structure and Development	Homing of lymphocytes	1.13E-03	Decreased	-2.673	35	ADAM10, ADAM17, CCL23, CCL5, CCR1, CUX1, CXCL16, CXCL6, CXCL9, CXCR2, CXCR3, DEFB103A/DEFB103B, FOXP3, FUT7, FYB1, HLA-G, HSPD1, ITGA4, JAK1, LCP1, LTBR, MAPKAP1, MYLK, NEDD9, NR3C1, PF4, PTEN, RAC2, RHOA, STAT3, STK4, THBS2, TLR4, TNFSF14, WIPF1
Cell Death and Survival	Apoptosis of central nervous system cells	1.14E-03		-0.307	37	APP, BCL2L11, BECN1, DDIT3, EPO, F2R, FOXO3, GAPDH, GCLC, HSP90AA1, HSPA5, IGF1, IGF1R, IL1B, KLF6, LRPAP1, MAP3K1, MCL1, MEF2C, mir-26, NFATC4, NFE2L2, PITX2, PRKAA1, PTEN, PTGS2, RHOA, SEL1L, SGK1, SHC3, SKP2, SRPK2, TLR7, TNFRSF1A, TNFSF10, WNK3, YWHAB
Cell Death and Survival	Cell viability of blood cells	1.15E-03	Decreased	-5.3	61	ADGRE2, APOB, APP, ARNT, ATG3, BCL2L11, BID, BRCA1, BTK, CASP8, CD86, CFLAR, CSF1R, CTLA4, CX3CR1, DEF6, DOCK8, ELF1, EPO, F2R, FOXO3, GAB2, HBB, HCK, ICAM1, IGF1, IL1B, JAK1, KIF1C, KRAS, LAT2, LY9, LYN, MCL1, MEF2C, MGAT5, mir-24, MTOR,

						MVP, NCF2, PF4, PLCG2, PRKAA1, PROK2, PTPN6, RAC2, RAF1, RBPJ, RHOA, RICTOR, RIPK3, SOD2, STAT3, SYK, TLR4, TNFSF10, TRAF3, TYROBP, WIPF1, YWHAZ, ZEB2
Hematological System Development and Function, Organismal Functions	Coagulation of blood	1.15E-03		-1.844	60	ANXA2, ANXA5, APLP2, APP, ARNTL, C1GALT1C1, C4BPB, CALU, CAPZA1, CAPZB, CARMIL1, CCL5, CLEC1B, COL1A2, CYP4F2, DOCK8, EHD3, ENTPD1, F10, F13A1, F2R, F8, FCGR2A, GATA5, H3-3A/H3-3B, H3C1, H3C13, HBB, JMJD1C, LCP2, LYN, MAFF, MERTK, P2RX1, PDGFRA, PECAM1, PF4, PLCB3, PLCG2, PLEK, PRKAR1A, PRKCD, PRKCG, PRKG1, PTEN, PTGS2, PTPN6, RAB27A, RAB5A, RAD51C, RAF1, SGK1, SH2B3, SYK, THBS2, TLR2, TLR4, VCAN, YWHAZ, ZFPM2
Connective Tissue Disorders, Organismal Injury and Abnormalities, Skeletal and Muscular Disorders	Non-traumatic arthropathy	1.15E-03		0.826	172	ABCC2, ACO1, ACSL1, ADAM10, ADAM15, ADAM17, ADGRA1, ADIPOR1, ADM, AIF1, APLP2, APOA1, AQP9, ARF1, ARHGDIB, ATAT1, B2M, BGN, C9orf78, CARD8, CASC3, CCL23, CCL5, CCR1, CD86, CDA, CELF2, CLEC1B, CLEC4D, CLIC2, CNR1, CSF3R, CTLA4, CTSB, CTSC, CX3CR1, CXCL1, CXCL16, CXCL6, CXCL9, CXCR2, CXCR3, CYP4F3, DEF6, DNAJA4, DYNLL1, ECHDC1, EEF1E1, EIF1B, ELF3, EPO, F10, F11R, FCGR2A, FGL2, FKBP5, FOXO3, FOXP3, FPR2, FTH1, GALNT1, GLIPR2, GLUL, H3-3A/H3-3B, HAMP, HBA1/HBA2, HBB, HCK, HCLS1, HDAC7, HLA-A, HLA-C, HLA-G, HMOX1, HNMT, HNRNPA1, HSP90B1, HSPA1A/HSPA1B, HSPD1, ICAM1, IFNAR1, IGF1, IGFBP4, IL1B, IL1R2, IL1RN, JAK1, JMJD1C, KCTD20, KRAS, LCP1, LINC00922, LYZ, MACIR, MAP3K2, MAP4K4, MAPRE1, MCL1, MDM2, MEFV, MMP14, MRFAP1, MS4A1, MS4A7, MTOR, NAMPT, NOM1, NONO, NR3C1, NTRK1, NUMB, OXT, P2RY13, PDIA3, PECAM1, PHTF1, PLAC4, PSMB8, PTGS2, PTMA, PTPRE, RALB, RAMP2, RBPJ, RFX3, RGCC, RNF149, RNF169, RPL18A, RTF2, S100A9, SCN9A, SEC14L3, SEL1L, SF3B6, SLC22A4, SOD2, SORL1, SPOCK1, STAT3, STEAP4, STK19, SWT1, SYK, TALDO1, TCF4, TFRC, TJP1, TLR2, TLR4, TLR7, TNFRSF10C, TNFRSF10D, TNFRSF1A, TNFSF10, TNFSF4, TNNC1, TRIO, TUBA1A, TUBA1C, TUBB2A, TUT7, UQCRC2, USP15, VDR, VIM, VTCN1, WNK1, ZNF143, ZNF281, ZNF326, ZNF331
Cancer, Hematological Disease, Immunological Disease, Organismal Injury and Abnormalities	Stage III-IV mantle cell lymphoma	1.18E-03			12	BTK, CSF3R, HSP90AA1, HSP90AB1, HSP90B1, MS4A1, NR3C1, PSMD1, PSMD2, TUBA1A, TUBA1C, TUBB2A
Cell Morphology, Inflammatory Response	Shape change of phagocytes	1.18E-03	Decreased	-2.791	12	ATRN, CLEC1B, HCK, ICAM1, LYN, PECAM1, PLCG2, RHOA, RHOB, SIRPA, SYK, WIPF1
Cancer	Sphere formation of carcinoma cell lines	1.18E-03		-1.316	8	DPP10-AS1, HTATIP2, let-7, MTHFD2, MYOF, PTEN, SRGN, STAT3
Cell Death and Survival	Cell death of mesothelioma cell lines	1.18E-03		0.111	8	BID, CASP8, CFLAR, MCL1, NOTCH2, SFRP4, STAT3, TNFSF10
Cell-mediated Immune Response, Cellular Movement, Hematological System Development and Function, Immune Cell Trafficking	Cell movement of peripheral T lymphocyte	1.18E-03	Decreased	-2.191	8	CCL5, CXCL16, FYB1, ICAM1, LCP1, LCP2, NR3C1, PLCB3
Cancer, Organismal Injury and Abnormalities, Tumor Morphology	Progressive recurrent neoplasm	1.21E-03			21	CSF1R, CTLA4, HSP90AA1, HSP90AB1, HSP90B1, IGF1R, LHCGR, MERTK, MS4A1, NR3C1, NTRK1, PDGFRA, PSMB8, PSMD1, PSMD2, PTGS2, RAF1, SLAMF7, TUBA1A, TUBA1C, TUBB2A
Protein Synthesis	Translation of protein	1.23E-03		0.675	70	ACO1, AGO2, ALKBH1, APP, ATF5, BTG2, BTK, CASC3, CNBP, CPEB1, DDX3X, EEF2K, EIF1AX, EIF3A, EIF3G, EIF3I, EIF4G3, EIF4H, FOXO3, FUS, GAPDH, HELZ, HSPA1A/HSPA1B, HSPA5, IGF1, IGF2BP3, ILF3, IREB2, KRAS, LARP4B, let-7, MARS1, MKNK1, MRPL15, MRPL18, MRPL28, MRPL55, MRPS10, MRPS18A, MRRF, MTOR, MTRF1L, NCBP1, NCL, OXA1L, PABPC1, PDCD4, PIWIL1, PRKAA1, PTCD3, RBM4, RGS2, RNASET2, RPL13A, RPL18, RPL18A, RPL28, RPL38, RPL39, RPL4, RPL5, RPS15, S100A9, SRSF3, STAU1, SYK, TNFSF10, TNIP1, WARS1, YBX1

Cancer, Organismal Injury and Abnormalities	Development of head and neck tumor	1.26E-03		1.234	30	AKAP12, ANXA2, APC, BTG2, CSF3R, CYP51A1, EWSR1, FOXO3, HSP90AA1, HSP90AB1, HSP90B1, KDM5A, KRAS, MAX, MTDH, NAMPT, PER2, PPM1D, PRKAR1A, PRL, PTEN, RAF1, SDHD, SSBP2, TDGF1, TUBA1A, TUBA1C, TUBB2A, VDR, XRCC5
Connective Tissue Disorders, Immunological Disease, Inflammatory Disease, Organismal Injury and Abnormalities, Skeletal and Muscular Disorders	Lupus erythematosus	1.26E-03		1.423	79	APOBEC3A, BCL2L11, CASP8, CD86, CFLAR, CPT1A, CRB1, CREB1, CTLA4, CXCL16, CXCL9, DDIT3, DGAT2, DKK3, DUSP5, ERCC5, FCGR2A, FOXO3, HLA-A, HOXA7, ICAM1, IFI16, IFNGR1, IGF1R, IL1B, IL1R2, IRF8, ITGAX, JAK1, LINC-PINT, LY9, LYN, MCL1, MERTK, mir-154, mir-24, mir-299, MS4A1, MTOR, NCF2, NR3C1, OPA1, PLA2G4C, PROK2, PSME3, PTGS2, PTPN6, RAB27A, RAB31, RAB5A, RABGAP1L, RFPL2, RIPK3, S100A9, SGK1, SKP2, SLAMF7, SLU7, SOD2, SOS2, SP1, SRSF1, STAT3, STXBP6, TBK1, TLR2, TLR4, TLR5, TLR7, TMEM39A, TNFRSF1A, TNFSF10, TNFSF4, TNIP1, TNPO3, UBE2L3, VDR, WARS1, ZNF148
Cell Death and Survival	Necroptosis	1.28E-03		-1.062	25	ATP6AP2, CASP8, CD14, CFLAR, CWC15, GAPDH, GRB2, HOXA3, HSP90AA1, HSP90AB1, IFNAR1, INSM2, IPMK, JAK1, MLKL, MTOR, NUDT13, PPIF, RIPK3, SF3B6, SOD2, TLR4, TMEM107, TNFRSF1A, TNFSF10
Cell Death and Survival	Apoptosis of macrophages	1.28E-03		-1.109	25	APP, CASP8, CCL5, CD14, CFLAR, CYBB, DDIT3, DFFA, IL1B, MCL1, MEFV, MTOR, NAMPT, NFE2L2, PELI2, PTEN, PTPN6, RIPK3, SOD2, STAT3, TLR2, TLR4, TNFRSF1A, TNFSF10, TREM1
Cancer, Organismal Injury and Abnormalities, Respiratory Disease	Malignant neoplasm of pleura	1.28E-03			19	ADAM10, CTLA4, DDX3X, FOXO3, HSP90AA1, HSP90AB1, HSP90B1, KRAS, LYN, MYBBP1A, PDGFRA, PDZD8, SETDB1, SP1, THBS2, TTF1, TUBA1A, TUBA1C, TUBB2A
Cancer, Organismal Injury and Abnormalities, Tumor Morphology	Progression of malignant tumor	1.29E-03		0.822	45	ADAM10, ANXA3, BTK, COL1A2, CSF1R, CSF3R, CTLA4, CYP51A1, FOXO3, FUS, HSP90AA1, HSP90AB1, HSP90B1, IGF1R, ITGA4, KRAS, LHCGR, LIMK2, LYN, MERTK, mir-26, MKNK1, MS4A1, MTOR, NR3C1, NTRK1, OAZ1, PDGFRA, PSMB8, PSMD1, PSMD2, PTEN, PTGS2, RAF1, S100A9, SAT1, SF3B1, SKP2, SLAMF7, STAT3, TDGF1, TLR2, TUBA1A, TUBA1C, TUBB2A
Cancer, Neurological Disease, Organismal Injury and Abnormalities	Desmoplastic medulloblastoma	1.30E-03			9	HSP90AA1, HSP90AB1, HSP90B1, IGF1R, MSH6, SUFU, TUBA1A, TUBA1C, TUBB2A
Cellular Assembly and Organization	Accumulation of lysosome	1.30E-03		-0.558	9	APP, ATG7, BECN1, BORCS5, CTSB, IGF1, IGF1R, MYOF, VTI1B
Cancer, Hematological Disease, Immunological Disease, Organismal Injury and Abnormalities, Tumor Morphology	Progressive multiple myeloma	1.30E-03			9	HSP90AA1, HSP90AB1, HSP90B1, NR3C1, PSMB8, PSMD1, PSMD2, PTGS2, SLAMF7
Cancer, Hematological Disease, Immunological Disease, Organismal Injury and Abnormalities	Stage I Hodgkin disease	1.30E-03			9	CSF3R, HSP90AA1, HSP90AB1, HSP90B1, MS4A1, NR3C1, TUBA1A, TUBA1C, TUBB2A
Cancer, Gastrointestinal Disease, Hematological Disease,	Small intestinal lymphoma	1.32E-03			10	B2M, BCL7A, HVCN1, IRF8, MCL1, NOTCH2, PTEN, RHOA, TET2, TRAF3

Immunological Disease, Organismal Injury and Abnormalities						
Cell-To-Cell Signaling and Interaction, Cellular Compromise, Cellular Function and Maintenance, Inflammatory Response	Respiratory burst of phagocytes	1.33E-03		-1.903	13	APP, CXCL1, FPR1, HCK, ICAM1, IRF8, ITGA4, LILRB3, LYN, PF4, SYK, TREM1, TYROBP
Cellular Assembly and Organization	Remodeling of actin cytoskeleton	1.33E-03	Decreased	-2.891	13	APP, BTG2, CCDC88A, CXCR2, F2R, GAB1, MSN, PAK2, RAB5A, RHOA, RICTOR, TLR4, TRIO
Cancer, Gastrointestinal Disease, Organismal Injury and Abnormalities, Respiratory Disease	Stage IVA nasopharyngeal cancer	1.35E-03			3	HSP90AA1, HSP90AB1, HSP90B1
Cellular Development, Hematological System Development and Function, Lymphoid Tissue Structure and Development	Maturation of peripheral dendritic cells	1.35E-03			3	TLR2, TLR4, TNFRSF1A
Cancer, Organismal Injury and Abnormalities, Respiratory Disease	Stage IV ALK mutation negative EGFR mutation negative nonsquamous non-small cell lung carcinoma	1.35E-03			3	HSP90AA1, HSP90AB1, HSP90B1
Cancer, Gastrointestinal Disease, Organismal Injury and Abnormalities, Respiratory Disease	Stage 4a unresectable hypopharyngeal squamous cell carcinoma	1.35E-03			3	HSP90AA1, HSP90AB1, HSP90B1
Cancer, Organismal Injury and Abnormalities, Reproductive System Disease	Stage IV BRCA mutation positive triple negative breast cancer	1.35E-03			3	HSP90AA1, HSP90AB1, HSP90B1
Cancer, Organismal Injury and Abnormalities, Reproductive System Disease	Stage IA cervical cancer	1.35E-03			3	HSP90AA1, HSP90AB1, HSP90B1

Cancer, Organismal Injury and Abnormalities, Respiratory Disease	Stage 3 inoperable non-small cell lung cancer	1.35E-03			3	HSP90AA1, HSP90AB1, HSP90B1
Cancer, Organismal Injury and Abnormalities, Respiratory Disease	Stage 4a undifferentiated laryngeal carcinoma	1.35E-03			3	HSP90AA1, HSP90AB1, HSP90B1
Cell Morphology, Cellular Development, Cellular Growth and Proliferation, Tissue Development	Morphogenesis of stem cells	1.35E-03			3	ATP6AP2, FZD3, PIGA
Cancer, Organismal Injury and Abnormalities	Stage IVB loco-regionally advanced squamous cell carcinoma of the head and neck	1.35E-03			3	HSP90AA1, HSP90AB1, HSP90B1
Cancer, Gastrointestinal Disease, Organismal Injury and Abnormalities, Respiratory Disease	Stage III CDKN2A positive oropharyngeal carcinoma	1.35E-03			3	HSP90AA1, HSP90AB1, HSP90B1
Cancer, Organismal Injury and Abnormalities, Reproductive System Disease	Stage IB2-IVA invasive cervical squamous cell carcinoma	1.35E-03			3	HSP90AA1, HSP90AB1, HSP90B1
Cancer, Organismal Injury and Abnormalities	High-risk occult primary cancer of head and neck	1.35E-03			3	HSP90AA1, HSP90AB1, HSP90B1
Cancer, Organismal Injury and Abnormalities, Respiratory Disease	Stage 4b undifferentiated laryngeal carcinoma	1.35E-03			3	HSP90AA1, HSP90AB1, HSP90B1
Inflammatory Response	Sjogren's-syndrome like inflammation	1.35E-03			3	FOXO3, NFKBIZ, STAT3
Cancer, Organismal Injury and Abnormalities, Reproductive System Disease	Stage IB2-IVA primary cervical adenocarcinoma	1.35E-03			3	HSP90AA1, HSP90AB1, HSP90B1
Cellular Movement, Tissue Morphology	Formation of endothelial dome	1.35E-03			3	CXCL1, IL1B, LSP1
Cancer, Organismal Injury and Abnormalities, Respiratory Disease	TNM stage T3 glottis cancer	1.35E-03			3	HSP90AA1, HSP90AB1, HSP90B1

Cancer, Connective Tissue Disorders, Gastrointestinal Disease, Organismal Injury and Abnormalities, Respiratory Disease, Skeletal and Muscular Disorders	High-risk squamous cell cancer of the maxillary sinus	1.35E-03			3	HSP90AA1, HSP90AB1, HSP90B1
Cancer, Organismal Injury and Abnormalities, Respiratory Disease	Locally advanced glottis cancer	1.35E-03			3	HSP90AA1, HSP90AB1, HSP90B1
Cancer, Gastrointestinal Disease, Hepatic System Disease, Organismal Injury and Abnormalities	Non-well differentiated fetal hepatoblastoma	1.35E-03			3	HSP90AA1, HSP90AB1, HSP90B1
Cancer, Gastrointestinal Disease, Organismal Injury and Abnormalities, Respiratory Disease	High-risk squamous cell cancer of the oropharynx	1.35E-03			3	HSP90AA1, HSP90AB1, HSP90B1
Cancer, Organismal Injury and Abnormalities, Respiratory Disease	Stage III locally advanced laryngeal squamous cell carcinoma	1.35E-03			3	HSP90AA1, HSP90AB1, HSP90B1
Cancer, Gastrointestinal Disease, Organismal Injury and Abnormalities, Respiratory Disease	Stage 3 resectable hypopharynx carcinoma	1.35E-03			3	HSP90AA1, HSP90AB1, HSP90B1
Cancer, Organismal Injury and Abnormalities, Respiratory Disease	Stage IVA locally advanced laryngeal squamous cell carcinoma	1.35E-03			3	HSP90AA1, HSP90AB1, HSP90B1
Cancer, Organismal Injury and Abnormalities, Respiratory Disease	Ethmoid sinus adenocarcinoma	1.35E-03			3	HSP90AA1, HSP90AB1, HSP90B1
Cancer, Organismal Injury and Abnormalities, Respiratory Disease	High-risk squamous cell cancer of the ethmoid sinus	1.35E-03			3	HSP90AA1, HSP90AB1, HSP90B1
Cancer, Organismal Injury and	Resectable glottis cancer	1.35E-03			3	HSP90AA1, HSP90AB1, HSP90B1

Abnormalities, Respiratory Disease						
Cancer, Organismal Injury and Abnormalities, Respiratory Disease	Stage 3 resectable laryngeal squamous cell carcinoma	1.35E-03			3	HSP90AA1, HSP90AB1, HSP90B1
Cancer, Organismal Injury and Abnormalities, Respiratory Disease	Stage 4a resectable laryngeal squamous cell carcinoma	1.35E-03			3	HSP90AA1, HSP90AB1, HSP90B1
Cancer, Organismal Injury and Abnormalities, Respiratory Disease	Stage 3 undifferentiated laryngeal carcinoma	1.35E-03			3	HSP90AA1, HSP90AB1, HSP90B1
Cancer, Dermatological Diseases and Conditions, Gastrointestinal Disease, Organismal Injury and Abnormalities	High-risk squamous cell cancer of the lip	1.35E-03			3	HSP90AA1, HSP90AB1, HSP90B1
Cancer, Organismal Injury and Abnormalities, Respiratory Disease	High-risk squamous cell cancer of the glottis	1.35E-03			3	HSP90AA1, HSP90AB1, HSP90B1
Cancer, Organismal Injury and Abnormalities, Respiratory Disease	Resectable supraglottis cancer	1.35E-03			3	HSP90AA1, HSP90AB1, HSP90B1
Cancer, Gastrointestinal Disease, Organismal Injury and Abnormalities	Adenoid cystic carcinoma of parotid gland	1.35E-03			3	HSP90AA1, HSP90AB1, HSP90B1
Cancer, Gastrointestinal Disease, Organismal Injury and Abnormalities, Respiratory Disease	Stage IVA locally advanced hypopharyngeal squamous cell carcinoma	1.35E-03			3	HSP90AA1, HSP90AB1, HSP90B1
Cancer, Organismal Injury and Abnormalities, Respiratory Disease	High-risk squamous cell cancer of the supraglottis	1.35E-03			3	HSP90AA1, HSP90AB1, HSP90B1
Cancer, Organismal Injury and Abnormalities, Reproductive System Disease	Invasive cervical adenocarcinoma	1.35E-03			3	HSP90AA1, HSP90AB1, HSP90B1

Cancer, Organismal Injury and Abnormalities, Respiratory Disease	Advanced stage primary laryngeal cancer	1.35E-03			3	HSP90AA1, HSP90AB1, HSP90B1
Cancer, Organismal Injury and Abnormalities, Respiratory Disease	Locally advanced supraglottis cancer	1.35E-03			3	HSP90AA1, HSP90AB1, HSP90B1
Cancer, Organismal Injury and Abnormalities	Stage IVA loco-regionally advanced squamous cell carcinoma of the head and neck	1.35E-03			3	HSP90AA1, HSP90AB1, HSP90B1
Infectious Diseases	Candidemia	1.35E-03			3	CYP51A1, IFNGR1, IFNGR2
Hematopoiesis, Lymphoid Tissue Structure and Development	Frequency of transitional type 3 B lymphocytes	1.35E-03			3	BCL2L11, CTLA4, LYN
Inflammatory Disease, Organismal Injury and Abnormalities, Respiratory Disease	Pleurisy	1.35E-03			3	IL1RN, TLR2, TLR4
Cancer, Organismal Injury and Abnormalities, Reproductive System Disease	FIGO stage IIA2 cervical cancer	1.35E-03			3	HSP90AA1, HSP90AB1, HSP90B1
Cancer, Gastrointestinal Disease, Organismal Injury and Abnormalities, Respiratory Disease	Primary oropharyngeal squamous-cell carcinoma	1.35E-03			3	HSP90AA1, HSP90AB1, HSP90B1
Cancer, Gastrointestinal Disease, Organismal Injury and Abnormalities, Respiratory Disease	Stage III locally advanced hypopharyngeal squamous cell carcinoma	1.35E-03			3	HSP90AA1, HSP90AB1, HSP90B1
Cancer, Organismal Injury and Abnormalities, Reproductive System Disease	Primary cervical adenosquamous carcinoma	1.35E-03			3	HSP90AA1, HSP90AB1, HSP90B1
Cancer, Gastrointestinal Disease, Organismal Injury and Abnormalities, Respiratory Disease	Undifferentiated oropharyngeal carcinoma	1.35E-03			3	HSP90AA1, HSP90AB1, HSP90B1

Hematological System Development and Function, Immune Cell Trafficking, Inflammatory Response, Tissue Development	Accumulation of effector memory T lymphocytes	1.35E-03			3	CCR1, DEF6, FOXO3
Cancer, Gastrointestinal Disease, Organismal Injury and Abnormalities, Respiratory Disease	TNM stage T2-4 nasopharyngeal cancer	1.35E-03			3	HSP90AA1, HSP90AB1, HSP90B1
Hematological Disease, Respiratory Disease	Hyperoxia	1.35E-03			3	SOD2, TLR2, TLR4
Cancer, Gastrointestinal Disease, Organismal Injury and Abnormalities, Respiratory Disease	CDKN2A overexpression negative hypopharyngeal squamous cell carcinoma	1.35E-03			3	HSP90AA1, HSP90AB1, HSP90B1
Renal and Urological System Development and Function, Tissue Development	Multilayering of kidney cell lines	1.35E-03			3	CTNND2, RAF1, RHOA
Cancer, Organismal Injury and Abnormalities, Reproductive System Disease	Primary cervical squamous cell carcinoma	1.35E-03			3	HSP90AA1, HSP90AB1, HSP90B1
Cancer, Organismal Injury and Abnormalities, Respiratory Disease	CDKN2A overexpression negative laryngeal squamous cell carcinoma	1.35E-03			3	HSP90AA1, HSP90AB1, HSP90B1
Cell-To-Cell Signaling and Interaction	Degradation of synapse	1.35E-03			3	APP, JAK1, STAT3
Cell-To-Cell Signaling and Interaction, Hematological System Development and Function, Immune Cell Trafficking, Inflammatory Response	Adhesion of phagocytes	1.35E-03	Decreased	-2.41	34	ADAM10, ADAM17, ADGRE2, APOA1, BTK, CCL5, CNR1, CSF3R, CTSZ, CXCL1, CXCR2, CYBB, F2R, HCK, ICAM1, IL1B, ITGA4, ITGAX, LCP1, LGALS8, LILRB3, LYN, MGAT5, PAK2, PF4, PLCB3, PTGS2, PTPN6, RAC2, RHOB, S100A9, TLR2, TLR4, TLR5

Cancer, Organismal Injury and Abnormalities	Secondary tumor	1.37E-03	Decreased	-2.883	206	ADAM10, ADAM15, ADAM17, ADM, AKAP12, ALDH5A1, ANGPTL4, ANTXR2, ANXA2, ANXA5, APC, APOA1, ARF4, ASAH1, ASXL1, AURKB, B2M, BACH1, BCL2L11, BRCA1, BRIP1, BTK, CALU, CASP8, CCDC88A, CCL5, CCR10, CD86, CDA, CFLAR, CHD4, CLCN3, CLEC4D, CLEC6A, CPEB1, CREB1, CSF1R, CSF3R, CTLA4, CTNNA1, CTNND1, CTNND2, CTSB, CTSZ, CUX1, CXCL1, CXCL6, CXCL9, CXCR2, CXCR3, CYBB, CYP51A1, DNAJB6, DPP10-AS1, DPYD, DPYSL2, EFS, ENTPD1, EPB41L3, EPO, ERCC5, ETV6, EXT1, EZR, F10, F2R, FCGR2A, FCGR2C, FNBP1L, FOXO3, FRS2, FTL, FUS, FUT7, G3BP2, GAS7, GLUL, GSE1, HCLS1, HLA-E, HLA-G, HMOX1, HOTAIR, HOXA10, HSP90AA1, HSP90AB1, HSP90B1, HSPA1A/HSPA1B, HTATIP2, ICAM1, IFNAR1, IFNGR1, IGF1, IGF1R, IL1B, IL1RN, IP6K2, ITGA4, JAK1, KAT6A, KCNJ2, KDM5A, KIDINS220, KLF6, KRAS, LAMA5, let-7, LGALS8, LHCGR, LIMK2, LYN, MAP3K1, MAP4, MCM3, MDM2, MERTK, mir-101, mir-122, mir-133, mir-137, mir-138, mir-154, mir-24, mir-26, mir-28, mir-450, mir-550, MKNK1, MMP14, MS4A1, MSH6, MTOR, MUC1, MYOF, NCF2, NEDD9, NKD2, NR3C1, NTRK1, NUMB, NUP93, OTUD3, PCOLCE, PDCD4, PDGFRA, PECAM1, PFKFB4, PHLPP1, PITX2, PPM1D, PRL, PSAP, PSMD1, PSMD10, PSMD2, PTEN, PTGS2, RAB31, RAC2, RAD51C, RAD51D, RAF1, RALB, RALBP1, RHOA, RHOB, RICTOR, RIOK3, RIPK3, RNF19B, RTN1, SCRIB, SDCBP, SERTAD2, SF3B1, SKP2, SOD2, SRD5A2, SSX2IP, STAT3, TET2, TLR2, TLR4, TLR7, TMBIM6, TNFRSF1A, TNNC1, TRIO, TUBA1A, TUBA1C, TUBB2A, U2AF1/U2AF1L5, USP4, VCAN, VDR, VIM, VTCN1, WNK1, WSB1, WWTR1, YWHAE, YWHAZ, ZEB2, ZFYVE21, ZNF350, ZRSR2
Cellular Movement, Hair and Skin Development and Function	Cell movement of epithelial cell lines	1.37E-03	Decreased	-2.675	35	APOA1, APP, ARF4, CCL5, CCR1, CLASP1, CNR1, CPEB1, CXCL1, CXCR2, EPHA8, F10, FBLN2, FPR2, FUT7, GAB1, GRB2, ICAM1, IGF1, IGF1R, KRAS, MAPRE1, MAPRE3, MSN, NARS1, PEAK1, PTEN, PTGS2, RHOA, RICTOR, SRSF1, TLR2, TXNRD1, VIM, WWTR1
Cellular Movement, Immune Cell Trafficking, Lymphoid Tissue Structure and Development	Homing of lymphatic system cells	1.37E-03	Decreased	-2.781	36	ADAM10, ADAM17, CCL23, CCL5, CCR1, CUX1, CXCL16, CXCL6, CXCL9, CXCR2, CXCR3, DEFB103A/DEFB103B, FOXP3, FUT7, FYB1, HLA-G, HSPD1, ITGA4, JAK1, LCP1, LTBR, MAPKAP1, MMP14, MYLK, NEDD9, NR3C1, PF4, PTEN, RAC2, RHOA, STAT3, STK4, THBS2, TLR4, TNFSF14, WIPF1
Lymphoid Tissue Structure and Development, Tissue Morphology	Quantity of lymphatic system cells	1.39E-03	Decreased	-4.66	157	ADAM10, ADGRG3, AP1G1, APBB1IP, APOA1, APP, ARHGDIB, ARID4B, ARNTL, ASXL1, ATG7, ATP6AP2, B2M, B4GALT1, BCL2L11, BID, BNIP3L, BTK, CASP8, CCL5, CCR1, CD84, CD86, CFLAR, CLEC4D, CLEC4M, CREB1, CSF1R, CSF3R, CTLA4, CTSB, CX3CR1, CXCL16, CXCR2, CXCR3, DCLRE1C, DDIT3, DEF6, DKK3, DMTF1, DOCK2, DOCK8, DUSP5, ELF1, EPO, FCGR2A, FOXO3, FOXP3, FUT7, FYB1, GAB2, GALNT1, GCNT2, HCK, HCLS1, HLA-A, HLA-G, HOXA3, HSP90B1, HSPD1, HVCN1, ICAM1, IFNAR1, IFNGR1, IGF1, IGF1R, IGF2R, IL1B, IPMK, IRF8, ITGB8, JAK1, KAT6A, KIDINS220, KRAS, LCP2, let-7, LGALS8, LGMN, LHCGR, LILRB3, LSP1, LTBR, LY9, LYN, MBP, MCL1, MDM2, MERTK, mir-24, MPZL2, MS4A1, MSN, MTOR, MTTP, MUC1, MXD1, NEDD9, NLK, NMT1, NOTCH2, NR3C1, NTRK1, PABPC1, PAK2, PECAM1, PLCG2, PLP1, PPM1D, PRKCD, PRL, PSAP, PSMB8, PSME3, PTEN, PTPN6, RAC2, RAF1, RAPGEF2, RASSF2, RBPJ, RGCC, RICTOR, RIPK2, RIPK3, RPS6KA5, SH2B3, SH3BP2, SIRPA, SLC6A6, SOD2, SPHK2, SSBP2, ST3GAL6, ST6GALNAC2, STAT3, STK4, SYK, TCF4, TDP2, TET2, THBS2, TLR2, TLR4, TMOD3, TNFRSF1A, TNFSF10, TNFSF4, TNIP1, TRAF3, TYROBP, VDR, VTCN1, WIPF1, XRCC5, ZEB2, ZRANB1
Cell Death and Survival	Apoptosis of pheochromocytoma cell lines	1.39E-03		-0.673	21	APP, ATN1, BCL2L11, BNIP3L, BTG2, DYNLL1, FFAR4, GAB1, IGF1, MAP3K1, PSAP, PTGS2, PTPN6, RIT1, SIAH1, SIRPA, SOD2, SPHK2, STAT3, TNFRSF1A, VCAN
Cell-To-Cell Signaling and Interaction, Hematological System Development and Function, Inflammatory Response	Binding of blood platelets	1.39E-03		-0.179	21	ANXA5, APP, C1GALT1C1, CD84, CLEC1B, CYBB, ENTPD1, F10, F8, FYB1, HCK, ICAM1, LCP2, LRP8, LRPAP1, PECAM1, PLCG2, RHOA, ST6GALNAC2, TLR2, WIPF1
Cell Death and Survival	Cell death of tumor cell lines	1.39E-03	Increased	2.501	318	ACO2, ADAM10, ADAM17, ADIPOR1, ADM, AKAP12, ALKBH3, ALS2, ANGPTL4, ANTXR2, ANXA2, ANXA5, APC, APOB, APP, ARL6IP1, ARNT, ASAH1, ATF5, ATG13, ATG14, ATG3, ATG7, ATN1, ATXN3, AURKB, B2M, BACH1, BCL2L11, BECN1, BID, BNIP2, BNIP3L, BRCA1, BTG2, BTK, CARD8, CASP8, CCP110, CCT2, CD14, CDA, CEACAM3, CELF1, CFLAR, CGB3 (includes others), CIBAR1, CKS2, CLASP1, CLCA2, CLK3, CNR1, CREB1, CSF1R, CTNND1, CTSB, CUX1, CXCR3, CYP2J2, DAB2, DDIT3, DDX3X, DFFA, DHCR7, DKK2, DKK3, DNAJB12, DPH2, DPYD, DTD2, DYNLL1, EEF2K, EIF1AX, EIF3G, EPO, EWSR1, EZR, FAIM2, FASTKD2, FFAR4, FKBP5, FOXL2, FOXO3, FOXP3, FTH1, GAB1, GAPDH, GAS7, GBE1, GIMAP4, GLIPR1, GSTA1, GUCA2A, GUCA2B, HCK, HCLS1, HDAC9, HERPUD1, HFE, HLA-G, HMOX1, HNRNPA1, HNRNPH1, HOTAIR, HSP90AA1, HSP90AB1, HSPA1A/HSPA1B, HSPA5, HSPD1, HTATIP2, IFI16, IFNAR1, IGF1, IGF1R, IGF2R, IGFBP4, IL1B, IL1RN, ING3, IP6K2, IPMK, IRF8, ITGA4, JAK1, KIDINS220, KIF1C, KLF6, KRAS, LAMA5, LCE1E, let-7,

						LGALS8, LGR5, LIMS1, LINC00887, LSP1, LTBR, LUCAT1, LYN, LYPLA2, MAP3K1, MAPKAP1, MAX, MBP, MCL1, MCM7, MDM2, MEF2C, MEFV, MERTK, mir-101, mir-103, mir-122, mir-133, mir-138, mir-154, mir-26, mir-299, mir-515, MIR4728, MKNK1, MLKL, MMP14, MOB3A, MS4A1, MSN, MT1F, MT1X, MTDH, MTOR, MUC1, MVP, MXD1, MYBBP1A, NABP1, NAMPT, NASP, NBR2, NCL, NCOA4, NDC80, NEDD9, NFE2L2, NFKBIZ, NOTCH2, NR3C1, NTRK1, NUMB, NUP58, NUP93, OAZ1, OPA1, PAK2, PARK7, PCK1, PDCD4, PDE4B, PDGFRA, PECAM1, PHLPP1, PIWIL1, PLAGL2, PLCG2, PLEKHA7, PLXNA4, PPM1D, PRKAA1, PRKAR1A, PRKCD, PRKG1, PRL, PSAP, PSMD2, PSMD4, PSMD7, PSME3, PTEN, PTGS2, PTMA, PTPN6, PTPRE, PUS10, RAB22A, RAD51C, RAET1E, RAF1, RALB, RALBP1, RAPGEF2, RASD1, RASSF3, RBM5, RHOA, RHOB, RICTOR, RIPK2, RIPK3, RIT1, RTN1, RTN4, S100A9, SAT1, SENP8, SFR1, SFRP4, SGK1, SH2B3, SH3RF1, SIAH1, SIRPA, SKP2, SLU7, SMAD1, SOD2, SP1, SPHK2, SPOCK1, SPOP, SRGN, SRPK1, SRPK2, SRSF1, STAT3, STAU1, STIP1, STK4, SUDS3, SVIL, SYK, TACC1, TAGLN2, TASP1, TBK1, TCF4, TCP1, TDGF1, TDP2, TERF2IP, TESK2, TFRC, THAP1, THOC5, TLR2, TLR4, TM9SF4, TMBIM6, TMCC3, TNFRSF10C, TNFRSF10D, TNFRSF1A, TNFSF10, TNFSF14, TRAF3, TREM1, TSG101, TTF1, TUBA1A, TXNRD1, UBA3, UBE2L3, UBE2V1, USP17L2 (includes others), VCAN, VDAC1, VDAC2, VDR, VPS35, WDR19, WSB1, WWTR1, XAF1, XRCC5, YBX1, YWHAE, YWHAZ, ZMYM3, ZNF148, ZNF229
Organismal Survival	Survival of organism	1.40E-03		-1.468	157	ACSL1, ADAM15, ADM, ALDH5A1, ALKBH1, ANTXR2, APC, APC2, APOA1, APP, AQP9, ARHGDIB, ARNT, ASAH1, ASXL1, ATG7, ATN1, ATP7B, AURKB, B2M, BARHL1, BCL2L11, BECN1, BGN, BID, BRCA1, CASP8, CBY1, CCNK, CCR1, CD14, CDA, CLEC4D, CLIC4, CNPY3, CNR1, CREB1, CTLA4, CTNND1, CTSB, CTSC, CXCL9, CXCR2, CXCR3, CYBB, DDX3X, DEFB103A/DEFB103B, DEFB114, DNM3, DUSP3, EEF2K, ELK3, ELN, EPO, F2R, FCGR2A, FGL2, FTL, GALC, GLUL, GSTA1, HFE, HLA-A, HMOX1, HOXA3, HSP90B1, HSPA1A/HSPA1B, HTATIP2, ICAM1, IFI16, IFNAR1, IFNGR1, IFT88, IGF1, IGF1R, IGF2R, IL1B, IL1RN, IRF8, ITGA4, KLF6, KRAS, LAT2, let-7, LITAF, LTBR, LYZ, MCL1, MDM2, mir-122, mir-515, MMP14, MS4A1, MTDH, MTTP, MUC13, MVP, MYOG, NAMPT, NCL, NCOA1, NDC80, NFE2L2, NINJ1, NOTCH2, NR3C1, NTRK1, PCLO, PER2, PPIF, PPM1D, PPP1CB, PRKAR1A, PRKG1, PTEN, PTGS2, PWWP3A, RAB5A, RAC2, RALBP1, RAMP2, RHOA, RIPK2, RIPK3, RPL13A, S100A9, SAV1, SF3B1, SKP2, SLC11A1, SLC31A1, SNX27, SOD2, SP1, STAT3, STIP1, SYK, TCF4, TET2, THBS2, TLR2, TLR4, TLR5, TLR7, TNFRSF1A, TNFSF10, TNFSF14, TNFSF4, TNIP1, TRAF3, TREM1, TSG101, VTCN1, WARS1, XRCC5, ZDHHC16, ZMPSTE24
Antimicrobial Response, Inflammatory Response	Antibacterial response	1.40E-03			30	ADM, ANXA3, APP, B2M, CLEC4D, DEFB103A/DEFB103B, FPR2, H2BC12, H2BC21, H2BC4, HCK, HLA-A, HLA-E, HLA-G, IRF8, LYZ, MPEG1, MYO1F, NOTCH2, PPM1D, PRKCD, RBPJ, RPL39, S100A9, SLC11A1, SYK, TBK1, TLR2, TLR4, TNFRSF1A
Cell Death and Survival	Apoptosis of uterine cell lines	1.41E-03		0	5	KRAS, MCL1, mir-24, PTGS2, RAF1
Infectious Diseases	Aspergillosis	1.41E-03			5	CLEC1A, CLEC7A, CYBB, CYP51A1, NR3C1
Cellular Movement, Hematological System Development and Function, Immune Cell Trafficking	Cell movement of lymphocytes	1.41E-03	Decreased	-4.27	86	ADAM10, ADAM17, APBB1IP, APC, APP, ATG7, BGN, BTK, CCL23, CCL5, CCR1, CCR10, CD86, CTLA4, CUX1, CX3CR1, CXCL1, CXCL16, CXCL6, CXCL9, CXCR2, CXCR3, CYP26B1, DEF6, DEFB103A/DEFB103B, DOCK2, DOCK8, DPYSL2, EFS, EZR, F11R, FOXP3, FUT7, FYB1, HCLS1, HLA-A, HLA-G, HMOX1, HSPD1, ICAM1, IFNAR1, IFNGR1, IL1B, ITGA4, JAK1, KCNE3, KRAS, LCP1, LCP2, LTBR, MAP3K2, MAPKAP1, MMP14, MSN, MTOR, MYLK, NEDD9, NR3C1, PECAM1, PF4, PLCB3, PRKAA1, PRKCD, PTEN, PTGS2, RAC2, RAP1A, RHOA, RICTOR, SCRIB, SERPINB3, SOS2, SPHK2, STAT3, STK4, SWAP70, THBS2, TLR2, TLR4, TNFRSF1A, TNFSF10, TNFSF14, TNFSF4, TNIP1, VTCN1, WIPF1
Cancer, Hematological Disease, Immunological Disease, Organismal Injury and Abnormalities	B cell cancer	1.43E-03		0.834	230	AMPH, ANKLE2, ANO5, ANXA2, ANXA5, APBA1, APC, APOBEC3A, APOBEC3B, APP, ARHGAP17, ASB10, ASMTL, ASXL1, ATF7IP, ATN1, ATRN, ATXN3, B2M, BAZ2B, BBS7, BCL2L11, BCL7A, BECN1, BOD1L1, BRCA1, BTG2, BTK, CARMIL1, CARNS1, CASP8, CAV3, CAVIN2, CCL5, CCNDBP1, CD86, CDC23, CFLAR, CGB1/CGB2, CHD4, CMSS1, CNR1, CPSF7, CRNN, CSDE1, CSF1R, CSF3R, CTSC, CXCL1, CXCL6, CXCL9, CYP2A6 (includes others), DCLRE1C, DDC, DMTF1, DNAJB14, DOCK2, DPYD, DYRK1A, EPHA8, ETV6, EWSR1, F10, F11R, FAM131C, FCGR2A, FOXO3, FOXP3, FRMD4B, FUS, FYB1, FZD3, GPD1, GPRIN1, GPSM2, GRB2, GSE1, HAO2, HCLS1, HDAC7, HDAC9, HECA, HLA-A, HLA-G, HMOX1, HSP90AA1, HSP90AB1, HSP90B1, HVCN1, ICAM1, IDH3A, IFNAR1, IFNGR1, IGF1, IGF2R, IL1B, IL1RN, IRF8, ITGA4, JAK1, KAT6A, KRAS, LCP1, LCP2, LCT, let-7, LRRFIP1, LSM3, LYN, LYPLA2, MAD2L1BP, MAP4K4, MAX, MCL1, MDM2, mir-138, mir-154, mir-28, MPEG1, MS4A1, MSH6, MTOR, MUC1, MYO5B, MYOF, NACA2, NAMPT, NDC80, NDUFS1, NETO2, NFE2L2, NONO, NOTCH2, NR3C1, NUBP1, NUDT6, NXPE4, PCLO, PDCD4, PDGFRA, PECAM1, PF4, PLCG2, PLEKHA7, POLR3B, POTEH (includes others), PPM1D, PPP1R12B, PPP4R2, PPP6C, PPP6R3, PRKAA1, PSMB8, PSMD1, PSMD2, PSME3, PTEN, PTGS2, PTPRE, PWWP3A, RAB38, RAB4A, RAF1, RBM4, RESF1, RHOA, RICTOR, RPS15, RTTN, S100A9, SCN9A, SEC14L1, SF3B1, SGK1, SH2B3, SHROOM3, SLAMF7, SMARCA2, SNRPD3, SOD2, SORL1, SRPK2, SSBP2, STAT3, STIP1, STXBP6, SWAP70, SYK, TAF1, TDRD1, TET2, TFRC, THBS2, TLR2, TLR4, TLR5, TLR7, TNFRSF10C, TNIP1, TNNI3K, TRAF3, TRIM55, TRIO, TRIOBP, TRIP12, TRPM6, TTC21B, TUBA1A, TUBA1C, TUBB2A, TXLNA, U2AF1/U2AF1L5, UBA3, UBE2F, UNC5C, VDAC1, VIM, WASF2, WDFY3, XRCC5, YAE1, YWHAE, YWHAZ, ZEB2, ZMYM3, ZNF217, ZNF615, ZNF700, ZNF714, ZRSR2

Molecular Transport, RNA Trafficking	Transport of mRNA	1.44E-03			23	CASC3, CPEB1, CPSF4, DDX39A, DHX38, FUS, HNRNPA2B1, MAGOHB, NCBP1, NUP160, NUP50, NUP58, NUP62, NUP93, SEC13, SLU7, SRSF1, SRSF3, SRSF4, SRSF5, THOC5, U2AF1/U2AF1L5, WDR33
Cardiovascular Disease, Cell Death and Survival, Organismal Injury and Abnormalities, Skeletal and Muscular Disorders	Necrosis of cardiac muscle	1.47E-03		0.937	47	ACSL1, ADM, APOA1, BACH1, BCL2L11, BECN1, BNIP3L, CASP8, CAV3, CYBB, CYP2J2, EPO, FOXO3, GAPDH, HMOX1, HSPD1, IGF1, IL1B, IL1RN, KRAS, MAP3K1, MCL1, MDM2, mir-133, mir-154, mir-24, MT1A, NAMPT, NCL, NFE2L2, PARK7, PPIF, PRKAA1, PRKCD, PTEN, RAF1, RHOA, RIPK3, RTN4, SGCA, SLC8A1, SOD2, STAT3, STK4, THBS2, TLR4, UBE4B
Cell Death and Survival	Cell survival	1.48E-03	Decreased	-8.529	296	ABCB5, ADAM17, ADGRE2, ADIPOR1, ADM, AGO2, AKAP8L, ALDH3A1, ALKBH3, ALKBH8, ALS2, ANTXR2, ANXA5, APC, APOB, APOBEC3A, APP, APPL2, ARNT, ASAH1, ATF5, ATG3, ATG7, ATP7B, AURKB, B2M, BABAM2, BCL2L11, BECN1, BID, BNIP2, BRCA1, BRIP1, BTG2, BTK, CAMK1G, CARS1, CASP8, CCL5, CCNK, CCR1, CD86, CDK2AP1, CFLAR, CHD4, CLCA2, CLK2, CLK3, CREB1, CRKL, CSF1R, CSF3R, CTLA4, CTSB, CUX1, CX3CR1, CXCL1, CXCL9, CXCR3, CYBB, DAB2, DAZ2, DCLRE1C, DDIT3, DDX3X, DDX5, DEF6, DEFB103A/DEFB103B, DEFB114, DHX38, DHX8, DNAJB6, DOCK8, DPH2, DPYD, DUSP5, DYRK1A, EEF2K, EIF3A, ELF1, ELF3, EPHB1, EPO, ERCC5, EWSR1, EYA3, EZR, F2R, FAIM2, FKBP5, FOXO3, FOXP3, FTH1, FTL, FUS, GAB1, GAB2, GCLC, GMFG, GRB2, HBA1/HBA2, HBB, HCK, HERPUD1, HLA-A, HLA-G, HMOX1, HNRNPUL2, HOTAIR, HSP90AB1, HSP90B1, HSPA1A/HSPA1B, HSPA5, HSPD1, HTATIP2, ICAM1, IGF1, IGF1R, IGF2BP3, IGF2R, IK, IL1B, IL1RN, JAK1, JMJD1C, KIF1A, KIF1C, KLF6, KRAS, LAMA5, LAT2, LIMK2, LIMS1, LRPAP1, LSM6, LUCAT1, LY9, LYN, LYZ, MAP3K1, MBP, MCFD2, MCL1, MCM7, MDM2, MEF2C, MEFV, MERTK, MGAT5, mir-133, mir-137, mir-154, mir-24, mir-26, mir-299, mir-515, MKNK1, MSH6, MTDH, MTOR, MVP, NAMPT, NCF2, NDC80, NEDD9, NFE2L2, NLK, NOTCH2, NR3C1, NTRK1, NUP58, NUP62, NUP93, OPN3, PAK2, PARK7, PDCD4, PDGFRA, PDIA3, PER2, PF4, PFKFB2, PHLPP1, PITX2, PLCG2, PLP1, POLDIP2, POLR1A, POU4F2, PPM1D, PPM1G, PPP1CB, PPP1R12B, PPP1R17, PPP6C, PRKAA1, PRKAR1A, PRKCD, PRKCG, PRKG1, PRL, PROK2, PSAP, PSMA4, PSMC2, PSMD1, PSMD12, PSME3, PTEN, PTGS2, PTPN6, PTPRE, RAB11A, RAB5A, RAC2, RAD51C, RAD51D, RAD52, RAF1, RALB, RBPJ, RHOA, RICTOR, RIPK2, RIPK3, RIT1, RPGRIP1, RPL38, RPN2, RPS6KA5, S100A9, SAT1, SDCBP, SEL1L, SETDB1, SF3B1, SFR1, SGK1, SHC3, SHLD1, SIAH1, SLC11A1, SLC31A1, SLC8A3, SMAD1, SMARCA2, SMARCC2, SNRPB, SNRPF, SOD2, SOS2, SPOP, SRGN, SRSF3, STAMBP, STAT3, STIP1, STX3, SUFU, SVIL, SYK, TBC1D9, TBK1, TCP1, TDP2, TET2, THBS2, THOC5, TLR2, TLR4, TNFRSF1A, TNFSF10, TRAF3, TRIM68, TSG101, TYROBP, U2AF1/U2AF1L5, UBE2L3, UBE2V1, USP15, VCAN, VDAC1, VDR, VIM, VTCN1, WIPF1, XAF1, XRCC5, YBX1, YPEL3, YWHAZ, ZEB2, ZNF257, ZNF429, ZNF431, ZNF528
Cellular Movement, Hematological System Development and Function, Immune Cell Trafficking	Cell rolling of granulocytes	1.49E-03	Decreased	-2	12	ADAM17, BTK, CXCL1, FUT7, HCK, ICAM1, IL1B, ITGA4, LYN, MGAT5, RAC2, SWAP70
Cancer, Organismal Injury and Abnormalities, Respiratory Disease	Lung metastasis by tumor	1.49E-03		-1.213	12	ANGPTL4, CTNND1, G3BP2, KRAS, MDM2, mir-24, NKD2, PTEN, RHOA, SKP2, STAT3, TMBIM6
Connective Tissue Disorders, Hematological Disease, Organismal Injury and Abnormalities	Erythrocytosis	1.49E-03		1.546	12	BNIP3L, BPGM, EPO, HAMP, HBA1/HBA2, HBB, NFE2L2, PTPN6, RAC2, RHOA, SH2B3, TRNT1
Cancer, Gastrointestinal Disease, Hepatic System Disease, Organismal Injury and Abnormalities	Hepatoblastoma	1.49E-03			12	APC, CELF1, HSP90AA1, HSP90AB1, HSP90B1, MSH6, MTOR, RAF1, SAV1, TUBA1A, TUBA1C, TUBB2A
Cell-To-Cell Signaling and	Recruitment of myeloid cells	1.49E-03	Decreased	-2.984	58	ADAM10, ADAM17, ALOX5AP, APOA1, APOB, APP, ATG7, B4GALT1, CASP8, CCL23, CCL5, CCR1, CD14, CNR1, CSF1R, CTSC, CX3CR1, CXCL1, CXCL6, CXCL9, CXCR2, FCGR2A, FPR2, FUT7, GAB2, GC, HCK, HSPA1A/HSPA1B, ICAM1, IFNAR1, IL1B, IL1RN, KRAS, LSP1, LYN,

Interaction, Cellular Movement						LYZ, MGAT5, NFE2L2, P2RX1, PDE4B, PECAM1, PTEN, RHOA, RHOB, RIPK2, RTN4, SIGLEC9, SOD2, ST3GAL6, STAT3, SWAP70, THBS2, TLR2, TLR4, TLR5, TNFRSF1A, TREML2, VDR
Cellular Movement, Hematological System Development and Function, Immune Cell Trafficking, Inflammatory Response	Cell movement of macrophages	1.50E-03	Decreased	-3.798	64	ADAM17, APOA1, APP, B4GALT1, BID, CASP8, CCDC88A, CCL5, CCR1, CNP, CNR1, CRKL, CSF1R, CX3CR1, CXCL1, CXCL9, CXCR3, CYBB, DEFB103A/DEFB103B, DOCK2, ELN, EPO, FPR2, GAL3ST1, HAMP, HCK, HMOX1, HSPA5, ICAM1, IFNGR1, IL1B, IL1RN, KLF6, LITAF, MYLK, NFE2L2, NFKBIZ, NINJ1, OPA1, PF4, PLP1, PRKCD, PSMB8, PTEN, PTGS2, PTMA, PTPN6, RAC2, RHOA, RHOB, RPL13A, SEMA4A, SH2B3, SIRPA, STAT3, TAFA4, THBS2, TLR2, TLR7, TNFSF4, TYROBP, VCAN, VTCN1, YBX1
Cell Signaling	Protein kinase cascade	1.51E-03	Decreased	-2.834	104	ADIPOR1, APC, APP, ATP6AP2, BNIP2, BTK, CARD16, CARD6, CARD8, CASC2, CASP8, CAV3, CCL5, CD14, CD84, CFLAR, CLEC6A, CRKL, DDX21, DLG3, DOK5, DUSP3, DUSP5, EPHA8, EPHB1, F2R, FRS2, GAB1, GFRAL, GPRC5B, GRB2, HACD3, IFNAR1, IGF1, IGF1R, IGFBP4, IL1B, JAK1, KRAS, LAMTOR5, LTBR, MAP3K1, MAP4K4, MBP, MEF2C, mir-26, MTDH, MTURN, MYLK2, NUP62, PAQR3, PDGFRA, PELI2, PHLPP1, POU4F2, PRKCD, PRL, PSAP, PSMA4, PSMB8, PSMC1, PSMC2, PSMD1, PSMD10, PSMD11, PSMD12, PSMD2, PSMD4, PSMD7, PSME3, PTPN6, RAF1, RAPGEF2, RASSF2, RHOA, RIOK3, RIPK2, RIPK3, RNF149, SFRP4, SH2B3, SH3RF1, SHC3, SIRPA, SLC11A1, SMAD1, SPAG9, SPHK2, SPTBN4, STAMBP, STAT3, TBK1, TERF2IP, TFRC, TIFA, TLR4, TNFRSF1A, TNFSF10, TNIP1, TPD52L1, TRIM5, UBE2V1, YWHAB, YWHAE
Cell Death and Survival, Skeletal and Muscular Disorders	Apoptosis of vascular smooth muscle cells	1.53E-03	Decreased	-2.128	18	APP, CASP8, DDIT3, FOXO3, HMOX1, IGF1, IGF1R, LIMS1, mir-138, PDCD4, PRKCD, PTEN, RIPK3, SP1, STAT3, STK4, TNFSF10, XAF1
Cancer, Organismal Injury and Abnormalities	Advanced stage tumor	1.53E-03	Decreased	-2.85	224	ADAM10, ADAM15, ADAM17, ADM, AKAP12, ALDH5A1, ANGPTL4, ANTXR2, ANXA2, ANXA5, APC, APOA1, ARF4, ASAH1, ASXL1, AURKB, B2M, BACH1, BCL2L11, BRCA1, BRIP1, BTK, CALCOCO2, CALU, CARD16, CASP8, CCDC88A, CCL5, CCR10, CD86, CDA, CFLAR, CHD4, CLCN3, CLEC4D, CLEC6A, CPEB1, CREB1, CSF1R, CSF3R, CTLA4, CTNNA1, CTNND1, CTNND2, CTSB, CTSZ, CUX1, CXCL1, CXCL6, CXCL9, CXCR2, CXCR3, CYBB, CYP51A1, DAB2, DNAJB6, DPP10-AS1, DPYD, DPYSL2, EFS, ENTPD1, EPB41L3, EPO, ERCC5, ETV6, EXT1, EZR, F10, F2R, FCGR2A, FCGR2C, FNBP1L, FOXO3, FRS2, FTL, FUS, FUT7, FZD3, G3BP2, GAS7, GLUL, GSE1, H2AC18/H2AC19, HCK, HCLS1, HLA-E, HLA-G, HMOX1, HOTAIR, HOXA10, HSP90AA1, HSP90AB1, HSP90B1, HSPA1A/HSPA1B, HTATIP2, ICAM1, IFNAR1, IFNGR1, IGF1, IGF1R, IGFBP4, IL1B, IL1RN, IP6K2, IRF8, ITGA4, JAK1, KAT6A, KCNJ2, KDM5A, KIDINS220, KLF6, KRAS, LAMA5, let-7, LGALS8, LHCGR, LIMK2, LRP2, LRP8, LYN, MAP3K1, MAP4, MCM3, MDM2, MERTK, mir-101, mir-122, mir-133, mir-137, mir-138, mir-154, mir-24, mir-26, mir-28, mir-450, mir-550, MKNK1, MMP14, MS4A1, MSH6, MTFR2, MTOR, MUC1, MYOF, NCF2, NEDD9, NKD2, NOTCH2, NR3C1, NTRK1, NUMB, NUP93, OTUD3, PCOLCE, PDCD4, PDGFRA, PECAM1, PF4, PFKFB4, PHLPP1, PITX2, PPM1D, PRL, PSAP, PSMD1, PSMD10, PSMD2, PTEN, PTGS2, RAB31, RAC2, RAD51C, RAD51D, RAF1, RALB, RALBP1, RGCC, RHOA, RHOB, RICTOR, RIOK3, RIPK3, RNF19B, RTN1, SCRIB, SDCBP, SERTAD2, SF3B1, SH2B3, SKP2, SOD2, SRD5A2, SSX2IP, STAT3, STK24, TET2, TLR2, TLR4, TLR7, TMBIM6, TNFRSF1A, TNNC1, TRIO, TUBA1A, TUBA1C, TUBB2A, U2AF1/U2AF1L5, USP4, VCAN, VDR, VIM, VTCN1, WNK1, WSB1, WWTR1, YWHAE, YWHAZ, ZEB2, ZFPM2, ZFYVE21, ZNF350, ZNF738, ZRSR2
Infectious Diseases	Infection by Herpesviridae	1.54E-03		0.108	24	APP, CCL5, CLIP1, CXCL9, CXCR2, DEFB103A/DEFB103B, F10, F2R, FCGR2A, FCGR2C, IFNAR1, IFNGR1, IGF2R, IL1RN, MAGT1, MAPRE1, MTOR, NFKBIZ, NR3C1, PDGFRA, PLCG2, TBK1, TLR2, TRAF3
Cellular Movement, Hematological System Development and Function, Immune Cell Trafficking, Inflammatory Response	Cellular infiltration by phagocytes	1.54E-03	Decreased	-2.251	65	ADAM17, ADM, ALOX5AP, ANXA2, APOA1, APP, ARHGAP25, B4GALT1, BECN1, BID, CASP8, CCR1, CD14, CD86, CNP, CNR1, CSF1R, CTSB, CTSC, CX3CR1, CXCR2, CYBB, CYP2J2, DOCK2, EPO, FPR1, FPR2, FUT7, GAL3ST1, HAMP, HMOX1, HSPA5, ICAM1, IFNGR1, IL1B, IL1RN, NFE2L2, NFKBIZ, NINJ1, OPA1, PF4, PLCB3, PLP1, PPM1D, PRKCD, PRKG1, PSMB8, PTEN, PTGS2, PTMA, PTPN6, RAB27A, RAC2, RPL13A, SGK1, STAT3, TLR2, TLR4, TLR7, TNFRSF1A, TNFSF4, TNIP1, TREM1, VTCN1, YBX1
Cell Death and Survival	Cell viability of leukocytes	1.55E-03	Decreased	-4.652	55	ADGRE2, APOB, APP, ATG3, BCL2L11, BRCA1, BTK, CASP8, CD86, CFLAR, CSF1R, CTLA4, CX3CR1, DEF6, DOCK8, ELF1, F2R, FOXO3, GAB2, HCK, ICAM1, IL1B, JAK1, KIF1C, KRAS, LAT2, LY9, LYN, MCL1, MEF2C, MGAT5, mir-24, MTOR, MVP, NCF2, PF4, PLCG2, PRKAA1, PTPN6, RAC2, RAF1, RBPJ, RHOA, RICTOR, RIPK3, SOD2, STAT3, SYK, TLR4, TNFSF10, TRAF3, TYROBP, WIPF1, YWHAZ, ZEB2
Hereditary Disorder, Organismal Injury and Abnormalities	X-linked hereditary disease	1.58E-03			76	ARHGEF9, ARMCX5-GPRASP2/GPRASP2, ATP6AP2, BGN, BNIP3L, BRWD3, BTK, CA4, CAP1, CCL5, CDKL5, CELF2, CHM, CLIC2, COL1A2, CPQ, CYBB, DAB2, DDX3X, DLG3, F10, F8, FCGR2A, FCGR2C, FOXO3, FOXP3, FRMD4B, GK, GLA, HCCS, HLA-A, HLA-C, HNRNPH2, IQSEC2, KLHL15, LAS1L, LHCGR, MAGT1, mir-133, mir-154, mir-299, MS4A1, MSN, MYLK2, NLGN3, NLGN4X, NONO, NR3C1, PDK3, PIGA, PLP1, PQBP1, PSAP, PTGS2, RP2, RS1, SCN9A, SDCBP, SMS, SZT2, TAF1, TAZ, TLR2, TLR4, TLR5, TMLHE, TMOD4, TSR2, TUBB2A, TXNRD1, VDR, VMA21, ZNF41, ZNF674, ZNF711, ZNF81

Hematological System Development and Function, Tissue Morphology	Quantity of leukocytes	1.60E-03	Decreased	-4.746	185	ADAM10, ADAM17, ADGRG3, ADM2, AP1G1, APBB1IP, APOA1, APOB, APP, ARHGDIB, ARID4B, ARNTL, ASXL1, ATG7, ATP6AP2, B2M, B4GALT1, BCL2L11, BID, BTK, CASP8, CCL5, CCR1, CD84, CD86, CFLAR, CHST1, CLEC4D, CLEC4M, CLEC7A, CLIC4, CREB1, CSF1R, CSF3R, CTLA4, CTSB, CX3CR1, CXCL16, CXCL6, CXCR2, CXCR3, CYBB, DCLRE1C, DDIT3, DEF6, DKK3, DMTF1, DOCK2, DOCK8, DUSP3, DUSP5, ELF1, EPO, F13A1, FCGR2A, FOXO3, FOXP3, FPR1, FPR2, FUT7, FYB1, GAB2, GALNT1, GCNT2, HBB, HCK, HCLS1, HLA-A, HLA-G, HMOX1, HOXA3, HSP90B1, HSPD1, HVCN1, ICAM1, IFNAR1, IFNGR1, IGF1, IGF1R, IGF2R, IL1B, IL1RN, IPMK, IRF8, ITGB8, JAK1, KAT6A, KDM5A, KIDINS220, KRAS, LCP2, LGALS8, LGMN, LHCGR, LILRB3, LITAF, LSP1, LTBR, LY9, LYN, MBP, MCL1, MDM2, MERTK, mir-122, mir-24, MPP1, MPZL2, MS4A1, MSN, MTOR, MTTP, MUC1, MXD1, NEDD9, NFE2L2, NMT1, NOTCH2, NR3C1, NTRK1, PABPC1, PDE4B, PECAM1, PILRA, PLCG2, PLP1, PPM1D, PRKCD, PRL, PROK2, PSAP, PSMB8, PSME3, PTEN, PTGS2, PTPN6, RAC2, RAF1, RASSF2, RBPJ, RGCC, RHOA, RICTOR, RIOX2, RIPK2, RIPK3, RPS6KA5, S100A9, SH2B3, SH3BP2, SIGLEC9, SIRPA, SLC6A6, SOS2, SPHK2, SSBP2, ST3GAL6, ST6GALNAC2, STAT3, STEAP4, STK4, SWAP70, SYK, TCF4, TDP2, TET2, TLR2, TLR4, TLR5, TLR7, TNFRSF1A, TNFSF10, TNFSF4, TNIP1, TRAF3, TYROBP, VCAN, VDR, VTCN1, WIPF1, XRCC5, YBX1, ZBTB46, ZEB2, ZRANB1
Cellular Assembly and Organization	Development of cytoplasm	1.60E-03	Decreased	-3.902	107	ABITRAM, ACTG1, ACTR2, AIF1, ANKRD13A, ANKRD13B, APC, APOA1, APOL1, APP, ARF1, ARHGAP19, ARPC2, ASB7, ATG13, ATG14, ATG3, ATG7, BECN1, CAMSAP2, CAPZB, CARMIL1, CAV3, CCDC88A, CLASP1, CLIP1, CNP, CREB1, CTNND1, CXCR2, DAB2, DPYSL2, DRG1, DYNLL1, DYRK1A, ELN, F11R, F2R, FCGR2A, FEZ1, GAS7, GLUL, GNG12, GNG7, GRB2, HCK, HCLS1, ICAM1, IFT88, IGF1, IL1B, KRAS, LAS1L, LAT2, LIMK2, MAP1LC3A, MAP3K1, MAPRE1, MAPRE3, MGAT5, mir-138, mir-24, MSRB1, MTOR, MYH14, MYLK, NCKAP1L, NIN, NTRK1, OPA1, PACSIN2, PAK2, PARK7, PECAM1, PEX19, PHACTR1, PID1, PITPNM1, PLCB3, POLDIP2, PRKCD, PTEN, RAB22A, RAB33B, RAB5A, RGCC, RHOA, RHOB, RICTOR, SGK1, SIRPA, SLC4A2, SNAPIN, STAT3, STK4, STX12, TESK2, TJP1, TLR2, TPM3, TRIP10, TTC17, UBAP2L, VMP1, WASF2, WASF3, WIPF1
Cellular Function and Maintenance, Molecular Transport, Small Molecule Biochemistry	Homeostasis of transition metal ion	1.60E-03			21	ACO1, APLP2, APP, ARF1, ATOX1, ATP6V1A, ATP6V1G1, ATP7B, B2M, FTH1, FTL, HAMP, HFE, HMOX1, IREB2, NCOA4, NUBP1, SLC11A1, SLC31A1, SOD2, TFRC
Infectious Diseases	Replication of virus	1.61E-03	Decreased	-3.627	109	ADAM10, AGO2, AMPH, ANXA5, APBB1IP, APC2, APOBEC3B, ARHGDIB, ARNTL, ATG7, ATP6AP2, ATP6V1A, ATP6V1B2, ATP6V1G1, B2M, BCL2L11, BECN1, BNIP2, CCL5, CCNK, CCR1, CFLAR, CLIC4, CPSF4, CREB1, DDIT3, DDX3X, DDX5, DTX2, DUSP3, DYRK1A, EIF3A, EIF3G, F11R, F13A1, FAS-AS1, GAB1, GALC, GCLC, GLYR1, HCK, HERPUD1, HMOX1, HSP90AA1, HSP90AB1, HSP90B1, HSPD1, IFNAR1, IL1B, ILF3, JAK1, MAP1LC3A, MAP3K7CL, MAP4K4, MDM2, MED30, mir-122, mir-515, MKNK1, MTOR, MVP, MX2, MYO5B, NCL, NUP62, P2RX1, PACSIN2, PAK2, PDE8A, PRKCD, PSMD2, PTGS2, RAB11A, RAB33B, RAB9A, RABEP1, RAF1, RFFL, RIPK2, RPL13A, S100A9, SF3B1, SF3B6, SGCA, SGK1, SNAP23, SNAPIN, SNRPF, SP100, SP110, SRPK1, SRPK2, SRSF1, STAT3, TBK1, TLR2, TLR4, TLR7, TNFRSF1A, TNFSF10, TNPO3, TRAF3IP1, TRIM5, TSG101, UBE2E2, USP15, VNN2, YBX1, ZEB2
Cardiovascular Disease, Cell Death and Survival, Organismal Injury and Abnormalities, Skeletal and Muscular Disorders	Cell death of cardiomyocytes	1.61E-03		1.063	46	ACSL1, ADM, APOA1, BACH1, BCL2L11, BECN1, BNIP3L, CASP8, CAV3, CYBB, CYP2J2, EPO, FOXO3, GAPDH, HMOX1, HSPD1, IGF1, IL1B, IL1RN, KRAS, MAP3K1, MCL1, MDM2, mir-133, mir-154, mir-24, MT1A, NAMPT, NCL, NFE2L2, PARK7, PPIF, PRKAA1, PRKCD, PTEN, RAF1, RHOA, RIPK3, RTN4, SLC8A1, SOD2, STAT3, STK4, THBS2, TLR4, UBE4B
Cellular Function and Maintenance, Hematological System Development and Function, Humoral Immune Response	Function of B lymphocytes	1.63E-03		-1.067	11	BTK, FCAMR, FCGR2A, LCP1, MTOR, NEDD9, PPM1D, PTEN, SH3BP2, STAT3, TET2
Cellular Development, Cellular Growth and Proliferation, Hematological System Development and Function,	Differentiation of phagocytes	1.65E-03	Decreased	-2.703	59	ADAM10, ADAM17, APP, BTK, CASP8, CCL23, CDA, CLEC4M, CSF1R, CSF3R, CYBB, DEF6, DMTF1, DUSP5, EPO, GAB2, GIT2, GMPR2, HCLS1, HOXA7, IFI16, IFNAR1, IL1B, IL1RN, IRF8, L3MBTL3, LILRA2, LILRB3, LTBR, LYN, MAPKAP1, MEF2C, MTOR, NOTCH2, PF4, PTEN, PTPN6, RBPJ, RFFL, S100A9, SFRP4, SH2B3, SIGLEC9, SP3, STAT3, TET2, THOC5, TLR2, TLR4, TLR5, TLR7, TNFRSF1A, TNFSF10, TREM1, TYROBP, VDR, VTCN1, ZBTB46, ZRSR2

Hematopoiesis, Lymphoid Tissue Structure and Development, Tissue Development						
Cell Morphology, Nervous System Development and Function, Organ Morphology, Organismal Development	Morphology of brain cells	1.65E-03			35	ALS2, APP, ATAT1, ATG7, BID, CAMK2A, CASP8, CCR10, CGA, CHMP4B, CLCN3, CTSB, DYRK1A, FAIM2, IGF1, IL1B, IREB2, KLF7, LRP8, MDM2, NCOA1, NDE1, NDEL1, PITPNA, PRL, PSAP, PTF1A, PURA, RICTOR, SMAD1, ST8SIA4, TLR7, TUBA1A, UBE4B, VPS35
Infectious Diseases	Replication of RNA virus	1.67E-03	Decreased	-3.935	99	ADAM10, AGO2, AMPH, ANXA5, APBB1IP, APC2, APOBEC3B, ARHGDIB, ARNTL, ATG7, ATP6AP2, ATP6V1A, ATP6V1B2, ATP6V1G1, B2M, BECN1, BNIP2, CCL5, CCNK, CCR1, CFLAR, CLIC4, CPSF4, CREB1, DDX3X, DDX5, DTX2, DUSP3, DYRK1A, EIF3A, EIF3G, F11R, F13A1, FAS-AS1, GAB1, GALC, GCLC, GLYR1, HCK, HERPUD1, HMOX1, HSP90AA1, HSP90AB1, HSPD1, IFNAR1, IL1B, ILF3, JAK1, MAP1LC3A, MAP3K7CL, MAP4K4, MDM2, MED30, mir-122, mir-515, MTOR, MVP, MYO5B, NCL, NUP62, P2RX1, PACSIN2, PAK2, PDE8A, PRKCD, PSMD2, PTGS2, RAB11A, RAB33B, RAB9A, RABEP1, RAF1, RFFL, RPL13A, S100A9, SF3B1, SF3B6, SGCA, SGK1, SNAPIN, SNRPF, SP110, SRPK1, SRPK2, SRSF1, STAT3, TBK1, TLR2, TLR4, TLR7, TNFSF10, TNPO3, TRAF3IP1, TRIM5, TSG101, UBE2E2, USP15, VNN2, YBX1
Cellular Assembly and Organization, Cellular Function and Maintenance	Assembly of multivesicular bodies	1.68E-03			8	CHMP2A, CHMP3, CHMP4B, CHMP6, RAB11A, TSG101, VPS25, VPS4B
Cell-To-Cell Signaling and Interaction, Hematological System Development and Function, Immune Cell Trafficking, Inflammatory Response	Activation of leukocytes	1.69E-03	Decreased	-2.979	135	ADAM10, ALS2, ANXA2, APOA1, APP, ATG7, B2M, BCL2L11, BID, BMP2K, BTK, CASP8, CCL23, CCL5, CD14, CD1E, CD84, CD86, CD93, CEACAM3, CLEC1B, CLEC4M, CLEC7A, CNR1, CRY2, CSF1R, CTLA4, CX3CR1, CXCL1, CXCL6, CXCL9, CXCR2, CYBB, DDIT3, DEF6, DOCK2, DUSP3, EPO, F10, FCGR2A, FOXO3, FOXP3, FPR1, FPR2, GC, HBP1, HCK, HDAC7, HDAC9, HEBP1, HLA-A, HLA-E, HLA-G, HMOX1, HSP90B1, HSPD1, ICAM1, IFNAR1, IGF1, IGF2R, IL1B, IL1RN, IRF8, ITGA4, JPH4, KIDINS220, LAT2, LCP2, let-7, LILRA2, LILRB3, LTBR, LYN, MAGT1, MBP, MERTK, MGAT5, MICB, mir-515, MS4A1, MTOR, NFE2L2, NFKBIZ, NOTCH2, PAK2, PECAM1, PF4, PILRB, PLCG2, PLP1, PPM1D, PRKAA1, PRKCD, PRL, PSAP, PSMB8, PTEN, PTGS2, PTPN6, PTPRE, RAB27A, RAB4A, RBPJ, RGMA, RHOA, RHOB, RIPK2, S100A9, SCN9A, SEMA4A, SIGLEC9, SIRPA, SLC11A1, SPHK2, STAT3, SWAP70, SYK, TBK1, TLR2, TLR4, TLR5, TLR7, TNFRSF1A, TNFSF10, TNFSF14, TNFSF4, TRAF3, TREM1, TREML2, TXLNA, TYROBP, VCAN, VTCN1, VTI1B, ZBTB46
Cell Death and Survival	Cell death of carcinoma cell lines	1.73E-03		1.151	78	ACO2, ADAM17, ANGPTL4, ANXA2, APP, ATG7, BCL2L11, BECN1, BID, CASP8, CCP110, CELF1, CFLAR, CREB1, CTSB, CXCR3, CYP2J2, DDIT3, DPYD, FAIM2, FOXO3, GAB1, GAPDH, HMOX1, HNRNPH1, HOTAIR, HSPA5, HTATIP2, IGF1, IGF1R, IP6K2, KLF6, KRAS, let-7, LINC00887, MCL1, MDM2, MEF2C, mir-103, mir-138, mir-154, mir-26, MTDH, MTOR, NR3C1, PDCD4, PDGFRA, PPM1D, PRKAR1A, PRKCD, PTEN, PTGS2, PTPN6, RAF1, RASD1, RHOB, RICTOR, RIPK3, SH2B3, SH3RF1, SKP2, SLU7, SPOCK1, SRGN, SRPK1, SRPK2, SRSF1, STAT3, TBK1, TLR4, TNFRSF1A, TNFSF10, TTF1, TXNRD1, VDAC1, YBX1, YWHAZ, ZNF148
Cellular Development, Cellular Growth and Proliferation, Connective Tissue Development and Function, Hematological System Development and Function, Hematopoiesis, Lymphoid Tissue Structure and Development,	Osteoclastogenesis of bone marrow-derived macrophages	1.73E-03	Increased	2.36	10	ADAM17, DYRK1A, IL1RN, IRF8, NOTCH2, PLCG2, PRKAA1, RBPJ, TMEM178A, TNFRSF1A

Skeletal and Muscular System Development and Function, Tissue Development						
Cardiovascular System Development and Function, Cell Death and Survival	Cell viability of endothelial cell lines	1.73E-03	Decreased	-2.985	10	ATG7, CXCR3, HMOX1, IGF1, MCL1, PDGFRA, SF3B1, SNRPF, THBS2, U2AF1/U2AF1L5
Dermatological Diseases and Conditions, Inflammatory Disease, Organismal Injury and Abnormalities	Acne	1.74E-03			22	AIF1, APOA1, APOBEC3A, CCR1, CD14, ICAM1, IGF1, IL1B, IL1R2, ITGA4, LYZ, NTRK1, PTGS2, RAC2, S100A9, SGK1, SOD2, SRD5A2, TLR2, TLR4, TRAF3, VDR
Cell-To-Cell Signaling and Interaction	Interaction of lymphoma cell lines	1.74E-03	Decreased	-2.2	22	ANXA2, APP, CLEC4M, CXCR3, ELN, EZR, F2R, FUT7, HCK, HSP90B1, ICAM1, IL1B, ITGA4, ITGAX, LCP2, MTOR, NCL, PECAM1, PTPN6, RHOA, TFRC, VCAN
Cancer, Organismal Injury and Abnormalities	Advanced malignant tumor	1.75E-03	Decreased	-2.85	223	ADAM10, ADAM15, ADAM17, ADM, AKAP12, ALDH5A1, ANGPTL4, ANTXR2, ANXA2, ANXA5, APC, APOA1, ARF4, ASAH1, ASXL1, AURKB, B2M, BACH1, BCL2L11, BRCA1, BRIP1, BTK, CALCOCO2, CALU, CARD16, CASP8, CCDC88A, CCL5, CCR10, CD86, CDA, CFLAR, CHD4, CLCN3, CLEC4D, CLEC6A, CPEB1, CREB1, CSF1R, CSF3R, CTLA4, CTNNA1, CTNND1, CTNND2, CTSB, CTSZ, CUX1, CXCL1, CXCL6, CXCL9, CXCR2, CXCR3, CYBB, CYP51A1, DAB2, DNAJB6, DPP10-AS1, DPYD, DPYSL2, EFS, ENTPD1, EPB41L3, EPO, ERCC5, ETV6, EXT1, EZR, F10, F2R, FCGR2A, FCGR2C, FNBP1L, FOXO3, FRS2, FTL, FUS, FUT7, FZD3, G3BP2, GAS7, GLUL, GSE1, H2AC18/H2AC19, HCK, HCLS1, HLA-E, HLA-G, HMOX1, HOTAIR, HOXA10, HSP90AA1, HSP90AB1, HSP90B1, HSPA1A/HSPA1B, HTATIP2, ICAM1, IFNAR1, IFNGR1, IGF1, IGF1R, IGFBP4, IL1B, IL1RN, IP6K2, IRF8, ITGA4, JAK1, KAT6A, KCNJ2, KDM5A, KIDINS220, KLF6, KRAS, LAMA5, let-7, LGALS8, LHCGR, LIMK2, LRP2, LRP8, LYN, MAP3K1, MAP4, MCM3, MDM2, MERTK, mir-101, mir-122, mir-133, mir-137, mir-138, mir-154, mir-24, mir-26, mir-28, mir-450, mir-550, MKNK1, MMP14, MS4A1, MSH6, MTFR2, MTOR, MUC1, MYOF, NCF2, NEDD9, NKD2, NOTCH2, NR3C1, NTRK1, NUMB, NUP93, OTUD3, PCOLCE, PDCD4, PDGFRA, PECAM1, PF4, PFKFB4, PHLPP1, PITX2, PPM1D, PRL, PSAP, PSMD1, PSMD10, PSMD2, PTEN, PTGS2, RAB31, RAC2, RAD51C, RAD51D, RAF1, RALB, RALBP1, RGCC, RHOA, RHOB, RICTOR, RIOK3, RIPK3, RNF19B, RTN1, SCRIB, SDCBP, SERTAD2, SF3B1, SKP2, SOD2, SRD5A2, SSX2IP, STAT3, STK24, TET2, TLR2, TLR4, TLR7, TMBIM6, TNFRSF1A, TNNC1, TRIO, TUBA1A, TUBA1C, TUBB2A, U2AF1/U2AF1L5, USP4, VCAN, VDR, VIM, VTCN1, WNK1, WSB1, WWTR1, YWHAE, YWHAZ, ZEB2, ZFPM2, ZFYVE21, ZNF350, ZNF738, ZRSR2
Cellular Development, Cellular Growth and Proliferation	Cell proliferation of tumor cell lines	1.75E-03	Decreased	-4.895	344	ABCB5, ABCC2, ACP3, ACTN1, ADAM10, ADAM15, ADAM17, ADIPOR1, ADM, AKAP12, ALKBH3, ANGPTL4, ANXA2, AP1G1, APC, APOB, APP, ARF1, ARID4B, ARIH2, ARNT, ARRDC3, ASAH1, ATF5, ATG7, AURKB, BARX2, BCL2L11, BECN1, BID, BNIPL, BRCA1, BTG2, BTK, CASC2, CASP8, CBX1, CCDC88A, CCNK, CDC42SE2, CDK2AP1, CELF2, CFLAR, CHCHD5, CHD4, CIBAR1, CKS2, CLCA2, CLCN3, CLIP1, CNR1, CPSF4, CREB1, CRKL, CSF1R, CTNND1, CTSB, CXCL1, CXCR2, CXCR3, CYP2A6 (includes others), CYP2J2, DAB2, DDIT3, DDX17, DDX21, DDX3X, DDX5, DKK3, DLX6-AS1, DMTF1, DNAJB4, DNAJB6, DPF2, DUSP5, EEF2K, EIF3A, EIF4H, ELF1, ELF3, EMC10, ENTPD1, EPB41L3, EPO, ERAS, ETV6, EWSR1, EXT1, EXTL3, EZR, F2R, FAIM2, FBLN2, FBXL15, FKBP5, FOXO3, FOXP3, FPR2, FTH1, FTL, FTX, FUS, GAB1, GAB2, GAPDH, GLIPR1, GNG7, GRB2, GSE1, GUCA2B, HBP1, HCK, HFE, HMOX1, HNRNPA1, HNRNPA2B1, HNRNPH2, HOTAIR, HSP90AA1, HSPA5, HTATIP2, HVCN1, IFI16, IFNAR1, IFT88, IGF1, IGF1R, IGF2BP3, IGF2R, IGFBP4, IL1B, IL1RN, ILF3, IRF8, ITGB8, JAK1, JPX, KAT6A, KCTD5, KDM5A, KIDINS220, KIF13A, KIF1A, KLF6, KRAS, LAMA5, LAMTOR5, LAS1L, LCOR, LCP1, let-7, LILRB3, LINC00511, LINC00887, LUCAT1, LUZP2, LYN, MAP1LC3A, MAP4K4, MAPRE1, MAX, MCL1, MCM7, MDM2, MEF2C, MGAT5, mir-101, mir-103, mir-122, mir-133, mir-154, mir-202, mir-24, mir-26, mir-28, mir-299, mir-515, MKNK1, MMP14, MSMB, MT1A, MTDH, MTOR, MUC1, MUC13, MYBBP1A, MYH14, MYLK, MYOF, MYOG, NABP1, NAMPT, NASP, NCL, NCOA1, NCOA4, NDRG4, NEDD9, NFATC4, NFE2L2, NFS1, NINJ1, NKD2, NONO, NOTCH2, NR3C1, NTRK1, NUBP1, NUDT6, NUMB, NUP62, OAZ1, OXT, PACSIN2, PBLD, PCOTH, PDCD4, PDGFRA, PDIA3, PDS5B, PEAK1, PECAM1, PHLPP1, PIWIL1, PLCG2, PLXNA4, POLDIP2, POU4F2, PPIF, PRKAA1, PRKAR1A, PRKCD, PRKG1, PRL, PRPF6, PRRC2C, PSMA4, PSMC2, PSMD2, PSMD4, PTEN, PTGS2, PTMA, PTPN6, PTPRE, PURA, RAB8A, RAF1, RALB, RALBP1, RAPGEF2, RASD1, RASSF3, RBM25, RBM5, RHOA, RHOB, RICTOR, RIOX2, RNF11, RNF149, RNF20, RNF40, S100A9, SAT1, SDCBP, SEL1L, SERTAD1, SETDB1, SFRP4, SGK1, SH2B3, SHC3, SIAH1, SKP2, SLC10A5, SLC36A1, SMAD1, SMARCA2, SNX27, SOD2, SP1, SP110, SPAST, SPHK2, SPOP, ST8SIA4, STAT3, STK24, STK38L, STK4, STX3, SUDS3, SUFU, SYK, TACC1, TAF7, TAGLN2, TASP1, TAZ, TBX5, TBXAS1, TCF4, TCN2, TCP1, TDGF1, TET2, TFRC, THBS2, TIFA, TIMM10B, TIPRL,

						TLR2, TLR4, TLR5, TNFRSF1A, TNFSF10, TNFSF14, TP53TG5, TPD52L1, TRAF3, TRIO, TRPM6, TSG101, TTF1, TUBB2A, UBE2J2, UNC5C, USP17L2 (includes others), VCAN, VDAC1, VDR, VMP1, VPS35, VTCN1, WSB1, WWTR1, XRCC5, YBX1, YWHAZ, ZEB2, ZNF267, ZNF282, ZNF350
Organ Morphology, Reproductive System Development and Function	Morphology of placenta	1.76E-03			32	ADAM17, ADM, AGO2, ALKBH1, ARNT, ARNTL, BCAS3, CHM, CRKL, DDX3X, DPH3, EPO, IGF1R, IGF2R, ITGA4, ITGB8, MMP14, NCOA1, NUMB, PTEN, RAF1, RAPGEF2, RBPJ, RGS2, RICTOR, RTEL1, SLC8A1, SMAD1, SNX13, STK4, TLK2, TRIP12
Cancer, Organismal Injury and Abnormalities	Complex adenocarcinoma	1.76E-03			9	DPYD, HSP90AA1, HSP90AB1, HSP90B1, KRAS, STAT3, TUBA1A, TUBA1C, TUBB2A
Cancer, Hematological Disease, Immunological Disease, Organismal Injury and Abnormalities	Stage II Hodgkin disease	1.76E-03			9	CSF3R, HSP90AA1, HSP90AB1, HSP90B1, MS4A1, NR3C1, TUBA1A, TUBA1C, TUBB2A
Cancer, Hematological Disease, Immunological Disease, Organismal Injury and Abnormalities	Stage III Hodgkin lymphoma	1.76E-03			9	CSF3R, HSP90AA1, HSP90AB1, HSP90B1, MS4A1, NR3C1, TUBA1A, TUBA1C, TUBB2A
Cancer, Hematological Disease, Immunological Disease, Organismal Injury and Abnormalities	Stage IV Hodgkin lymphoma	1.76E-03			9	CSF3R, HSP90AA1, HSP90AB1, HSP90B1, MS4A1, NR3C1, TUBA1A, TUBA1C, TUBB2A
Inflammatory Response	Innate immune response	1.78E-03	Decreased	-3.951	45	ADAM15, APOBEC3A, APOL1, APP, B2M, BTK, CAPZA1, CCL5, CD300E, CLEC4D, CLEC6A, CXCL1, CYBB, DDX3X, DEFB114, FPR1, FPR2, HLA-E, HLA-G, HSP90B1, let-7, MSRB1, NCF2, OTULIN, POLR3B, RIOK3, RIPK2, SIAH1, SIGLEC16, SIRPB1, SKP2, SYK, TBK1, TIFA, TKFC, TLR2, TLR4, TLR7, TRAF3, TREM1, TRIM23, TRIM5, TRIM55, TRIM65, TYROBP
Protein Synthesis	Translation	1.79E-03		0.523	71	ACO1, AGO2, ALKBH1, APP, ATF5, BTG2, BTK, CASC3, CNBP, CPEB1, DDX3X, EEF2K, EIF1AX, EIF3A, EIF3G, EIF3I, EIF4G3, EIF4H, FOXO3, FTH1, FUS, GAPDH, HELZ, HSPA1A/HSPA1B, HSPA5, IGF1, IGF2BP3, ILF3, IREB2, KRAS, LARP4B, let-7, MARS1, MKNK1, MRPL15, MRPL18, MRPL28, MRPL55, MRPS10, MRPS18A, MRRF, MTOR, MTRF1L, NCBP1, NCL, OXA1L, PABPC1, PDCD4, PIWIL1, PRKAA1, PTCD3, RBM4, RGS2, RNASET2, RPL13A, RPL18, RPL18A, RPL28, RPL38, RPL39, RPL4, RPL5, RPS15, S100A9, SRSF3, STAU1, SYK, TNFSF10, TNIP1, WARS1, YBX1
Hematological System Development and Function, Tissue Morphology	Quantity of blood cells	1.80E-03	Decreased	-4.917	205	ADAM10, ADAM17, ADGRG3, ADM2, AP1G1, APBB1IP, APOA1, APOB, APP, ARHGDIB, ARID4B, ARNTL, ASXL1, ATG7, ATP6AP2, B2M, B4GALT1, BCL2L11, BID, BNIP3L, BTK, CASP8, CCL5, CCR1, CD84, CD86, CFLAR, CHST1, CLEC4D, CLEC4M, CLEC7A, CLIC4, CREB1, CSF1R, CSF3R, CTLA4, CTSB, CX3CR1, CXCL16, CXCL6, CXCR2, CXCR3, CYBB, DCLRE1C, DDIT3, DEF6, DKK3, DMTF1, DOCK2, DOCK8, DUSP3, DUSP5, ELF1, EPO, F13A1, FCGR2A, FOXO3, FOXP3, FPR1, FPR2, FUT7, FYB1, GAB2, GALNT1, GCNT2, HBA1/HBA2, HBB, HCK, HCLS1, HLA-A, HLA-G, HMOX1, HOXA3, HSP90B1, HSPD1, HVCN1, ICAM1, IFNAR1, IFNGR1, IGF1, IGF1R, IGF2R, IL1B, IL1RN, IPMK, IREB2, IRF8, ITGB8, JAK1, KAT6A, KDM5A, KIDINS220, KRAS, L3MBTL3, LCP2, let-7, LGALS8, LGMN, LHCGR, LILRB3, LITAF, LSP1, LTBR, LY9, LYN, MBP, MCL1, MDM2, MERTK, mir-122, mir-24, MPP1, MPZL2, MS4A1, MSN, MTOR, MTTP, MUC1, MXD1, NEDD9, NFE2L2, NMT1, NOTCH2, NR3C1, NTRK1, PABPC1, PAK2, PARK7, PDE4B, PECAM1, PER2, PF4, PILRA, PITX2, PLCG2, PLP1, PPM1D, PRKAA1, PRKCD, PRKG1, PRL, PROK2, PSAP, PSMB8, PSME3, PTEN, PTGS2, PTPN6, PURA, RAB27A, RAC2, RAF1, RAPGEF2, RASSF2, RBPJ, RGCC, RHOA, RICTOR, RIOX2, RIPK2, RIPK3, RPS6KA5, S100A9, SH2B3, SH3BP2, SIGLEC9, SIRPA, SLC6A6, SLC8A1, SOS2, SPHK2, SSBP2, ST3GAL6, ST6GALNAC2, STAT3, STEAP4, STK4, SWAP70, SYK, TCF4, TDP2, TET2, TFRC, THBS2, TLR2, TLR4, TLR5, TLR7, TMOD3, TNFRSF1A, TNFSF10, TNFSF4, TNIP1, TPM3, TRAF3, TYROBP, VCAN, VDR, VTCN1, WIPF1, XRCC5, YBX1, ZBTB46, ZEB2, ZRANB1

Hematological System Development and Function, Tissue Morphology	Quantity of antigen presenting cells	1.81E-03	Decreased	-2.136	64	ADAM10, ADM2, APOB, APP, B2M, BCL2L11, BID, CD86, CFLAR, CLEC4D, CLIC4, CSF1R, CTLA4, CX3CR1, CYBB, DDIT3, DOCK8, DUSP3, EPO, FPR2, HCK, HMOX1, HOXA3, IFNAR1, IFNGR1, IGF1, IGF1R, IL1B, IL1RN, IRF8, KRAS, LHCGR, LITAF, LSP1, LTBR, MCL1, mir-122, MTOR, NFE2L2, NOTCH2, PILRA, PLP1, PRKCD, PTEN, PTPN6, RHOA, RICTOR, RIOX2, SH2B3, SIGLEC9, SIRPA, STAT3, STEAP4, STK4, TLR2, TLR4, TLR7, TNFRSF1A, TYROBP, VDR, VTCN1, YBX1, ZBTB46, ZEB2
Cellular Assembly and Organization	Remodeling of cytoskeleton	1.81E-03	Decreased	-2.891	15	APP, BTG2, CCDC88A, CSF1R, CXCR2, F2R, GAB1, MSN, PAK2, RAB5A, RHOA, RICTOR, TLR4, TRIO, VNN2
Cellular Movement, Hematological System Development and Function, Immune Cell Trafficking	Migration of granulocytes	1.81E-03	Decreased	-2.935	34	ADAM10, ADAM15, BTK, CCL5, CCR1, CXCL1, CXCL6, CXCL9, CXCR2, CYBB, F10, FPR1, HCK, ICAM1, IL1B, LAMA5, LSP1, MGAT5, mir-133, MYLK, MYO1F, PDE4B, PECAM1, PPM1D, PTEN, PTPN6, RTN4, S100A9, SIRPA, SWAP70, TLR2, TLR4, TLR7, TNFRSF1A
Cancer, Hematological Disease, Immunological Disease, Organismal Injury and Abnormalities	Enteropathy-associated T-cell lymphoma	1.82E-03			17	BRIP1, HDAC7, HDAC9, HSP90AA1, HSP90AB1, HSP90B1, JAK1, KRAS, MS4A1, NR3C1, PSMD1, PSMD2, STAT3, TET2, TUBA1A, TUBA1C, TUBB2A
Molecular Transport, RNA Trafficking	Nuclear export of mRNA	1.83E-03			21	CASC3, CPSF4, DDX39A, DHX38, HNRNPA2B1, MAGOHB, NCBP1, NUP160, NUP50, NUP58, NUP62, NUP93, SEC13, SLU7, SRSF1, SRSF3, SRSF4, SRSF5, THOC5, U2AF1/U2AF1L5, WDR33
Cell Morphology	Orientation of cells	1.84E-03	Decreased	-2.547	43	APC, AQP9, CCL5, CLIP1, CTLA4, CXCL9, CYBB, CYP26B1, DOCK2, DPYSL2, ELN, GAB1, HLA-G, HOXA3, HSBP1, IL1B, IL1RN, ITGA4, KIF26B, KRAS, LAMA5, LCP1, let-7, LSP1, MSN, MYLK, NAMPT, PRKAA1, PRKG1, PTEN, RAP1A, RBPJ, RHOA, RICTOR, SCRIB, STAT3, STK4, SVIL, SWAP70, TLR2, TLR4, WIPF1, WWTR1
Cancer, Gastrointestinal Disease, Hereditary Disorder, Organismal Injury and Abnormalities	Hereditary diffuse malignant gastric tumor	1.85E-03			4	CTNNA1, IL1B, IL1RN, KRAS
Cancer, Gastrointestinal Disease, Organismal Injury and Abnormalities	Resectable oral squamous cell carcinoma	1.85E-03			4	CTLA4, HSP90AA1, HSP90AB1, HSP90B1
Cancer, Organismal Injury and Abnormalities, Respiratory Disease	TNM stage T2 laryngeal squamous cell carcinoma	1.85E-03			4	CTLA4, HSP90AA1, HSP90AB1, HSP90B1
Cancer, Organismal Injury and Abnormalities, Tissue Morphology, Tumor Morphology	Volume of benign tumor	1.85E-03			4	APC, ATG7, KRAS, NR3C1
Cancer, Gastrointestinal Disease, Organismal Injury and Abnormalities, Respiratory Disease	CDKN2A negative oropharyngeal squamous cell carcinoma	1.85E-03			4	CTLA4, HSP90AA1, HSP90AB1, HSP90B1

Hematological System Development and Function, Immune Cell Trafficking, Inflammatory Response, Tissue Development	Accumulation of effector T lymphocytes	1.85E-03			4	CCR1, CXCR3, DEF6, FOXO3
Cancer, Organismal Injury and Abnormalities, Respiratory Disease	Resectable laryngeal squamous cell carcinoma	1.85E-03			4	CTLA4, HSP90AA1, HSP90AB1, HSP90B1
Cancer, Cell Death and Survival, Organismal Injury and Abnormalities, Tumor Morphology	Cell death of non-small-cell lung cancer cells	1.85E-03			4	BID, CTSB, PTEN, TNFSF10
Cancer, Gastrointestinal Disease, Organismal Injury and Abnormalities, Respiratory Disease	TNM stage T3 oropharyngeal squamous-cell carcinoma	1.85E-03			4	CTLA4, HSP90AA1, HSP90AB1, HSP90B1
Cancer, Gastrointestinal Disease, Organismal Injury and Abnormalities, Respiratory Disease	TNM stage T2 oropharyngeal squamous-cell carcinoma	1.85E-03			4	CTLA4, HSP90AA1, HSP90AB1, HSP90B1
Lipid Metabolism, Small Molecule Biochemistry	Binding of sterol	1.85E-03			4	APOA1, APP, NPC1L1, VDR
Cancer, Gastrointestinal Disease, Organismal Injury and Abnormalities, Respiratory Disease	TNM stage N2-3 oropharyngeal squamous-cell carcinoma	1.85E-03			4	CTLA4, HSP90AA1, HSP90AB1, HSP90B1
Cancer, Gastrointestinal Disease, Organismal Injury and Abnormalities	Primary oral squamous cell carcinoma	1.85E-03			4	HSP90AA1, HSP90AB1, HSP90B1, PTGS2
Tissue Development	Accumulation of cells	1.86E-03		-0.402	70	APC, APOA1, B2M, BCL2L11, BGN, BID, BRCA1, CASP8, CCL5, CCR1, CD86, CNR1, CTLA4, CTSC, CX3CR1, CXCL1, CXCL16, CXCR2, CXCR3, CYBB, DDIT3, DEF6, DOCK2, DOCK8, ENTPD1, F2R, FCGR2A, FOXO3, GATAD2A, HCK, HMOX1, ICAM1, IFI16, IFNAR1, IGF1, IL1B, IL1RN, ITGA4, ITGAX, KRAS, L3MBTL3, LAMA5, LRP8, LTBR, LYN, MAPKAP1, MERTK, NOTCH2, NR3C1, PPM1G, PTEN, PTGS2, RAC2, RBPJ, RHOB, RIPK3, S100A9, SH3BP2, SOS2, STAT3, STK4, TLR2, TLR4, TLR7, TNFRSF1A, TNFSF10, TNFSF4, TYROBP, UQCRC2, WASF2
Cell-To-Cell Signaling and Interaction, Hematological	Adhesion of neutrophils	1.86E-03		-1.545	23	ADAM10, ADAM17, ADGRE2, APOA1, CSF3R, CXCL1, CXCR2, CYBB, ICAM1, IL1B, ITGAX, LCP1, LGALS8, LILRB3, LYN, MGAT5, PF4, PLCB3, PTPN6, S100A9, TLR2, TLR4, TLR5

System Development and Function, Immune Cell Trafficking, Inflammatory Response						
Cell-To-Cell Signaling and Interaction, Hematological System Development and Function	Interaction of mononuclear leukocytes	1.92E-03	Decreased	-4.047	45	APBB1IP, APOA1, ATRN, B4GALT1, BTK, CCL5, CCR1, CD14, CD86, CLEC4M, CTLA4, CXCL9, CXCR3, DOCK2, DOCK8, EZR, FUT7, FYB1, ICAM1, IFNGR1, IL1B, ITGA4, ITGAX, JAK1, LCP1, LCP2, LTBR, MAP3K2, MSN, NEDD9, NR3C1, PECAM1, PRL, PTPN6, RAC2, RAP1A, RHOA, RICTOR, STK4, SWAP70, TFRC, THBS2, TLR2, TLR4, TNFSF14
Cell Death and Survival, Neurological Disease, Organismal Injury and Abnormalities	Cell death of cortical neurons	1.94E-03		0.072	39	APP, ATXN3, BCL2L11, BECN1, CASP8, CFLAR, DDIT3, EPO, FUS, GAPDH, GCLC, HSPA5, HSPD1, IGF1, IL1B, IL1RN, LRPAP1, MAP3K1, MCL1, MEF2C, mir-26, NFATC4, NFE2L2, NTRK1, PARK7, PTGS2, RHOA, SHC3, SP1, SP3, SRPK2, TCP1, TLR2, TLR4, TLR7, TNFRSF1A, UBE2L3, WNK3, YWHAB
Cellular Movement, Renal and Urological System Development and Function	Cell movement of kidney cell lines	1.98E-03	Decreased	-3.465	35	ANXA2, APC, APOA1, APP, CCL5, CCR1, CNR1, CTNND2, CXCL1, CXCR2, DAB2, EPHA8, EZR, F10, FBLN2, FPR2, FUT7, GAB1, GLIPR2, GRB2, ICAM1, MMP14, NARS1, PEAK1, RAC2, RAF1, RALB, RHOA, SNX27, SRSF1, STK24, TLR2, TXNRD1, VIM, WASF3
Cancer, Organismal Injury and Abnormalities, Reproductive System Disease	Uterine corpus cancer	2.01E-03			13	CSF3R, HSP90AA1, HSP90AB1, HSP90B1, KRAS, MTOR, NFE2L2, PTEN, SPOP, TUBA1A, TUBA1C, TUBB2A, U2AF1/U2AF1L5
Antigen Presentation, Inflammatory Response	Antigen presentation by leukocytes	2.01E-03		-1.274	13	CD86, CLEC9A, FCAMR, FCGR2A, HSP90AA1, IFNAR1, LILRA2, PSMB8, SEMA4A, SWAP70, SYK, TLR4, TNFSF4
Cell Morphology	Shape change of myeloid cells	2.01E-03	Decreased	-2.96	13	ATRN, CCL5, FYB1, HCK, ICAM1, LYN, PECAM1, PLCG2, RHOA, RHOB, SIRPA, SYK, WIPF1
Cell Death and Survival, Skeletal and Muscular Disorders	Apoptosis of muscle cells	2.05E-03		-0.475	54	ACSL1, ADM, APOA1, APP, BACH1, BCL2L11, BECN1, BNIP3L, CASP8, CAV3, CNR1, CYBB, CYP2J2, DDIT3, EPO, FOXO3, GAPDH, HMOX1, HSPD1, IGF1, IGF1R, IL1B, IL1RN, KRAS, let-7, LIMS1, MAP3K1, MCL1, MDM2, mir-133, mir-138, mir-154, mir-24, MT1A, NAMPT, PARK7, PDCD4, PRKAA1, PRKCD, PTEN, RAF1, RHOA, RIPK3, RTN4, SLC8A1, SOD2, SP1, STAT3, STK4, TLR4, TNFRSF1A, TNFSF10, UBE4B, XAF1
Hematological System Development and Function, Immune Cell Trafficking, Inflammatory Response, Tissue Development	Accumulation of leukocytes	2.06E-03		-0.545	56	APOA1, B2M, BCL2L11, BGN, CASP8, CCL5, CCR1, CD86, CTLA4, CTSC, CX3CR1, CXCL1, CXCR2, CXCR3, CYBB, DDIT3, DEF6, DOCK2, DOCK8, ENTPD1, F2R, FCGR2A, FOXO3, GATAD2A, HCK, HMOX1, ICAM1, IFNAR1, IL1B, IL1RN, ITGA4, ITGAX, KRAS, LAMA5, LTBR, LYN, MAPKAP1, NOTCH2, NR3C1, PTEN, PTGS2, RBPJ, RIPK3, S100A9, SH3BP2, SOS2, STAT3, STK4, TLR2, TLR4, TLR7, TNFRSF1A, TNFSF10, TNFSF4, TYROBP, UQCRC2
Cancer, Organismal Injury and Abnormalities, Reproductive System Disease	Metastatic prostate carcinoma	2.06E-03			11	CSF3R, CXCR3, HSP90AA1, HSP90AB1, HSP90B1, KLF6, LHCGR, NR3C1, TUBA1A, TUBA1C, TUBB2A

Cellular Development, Cellular Growth and Proliferation, Hematological System Development and Function, Hematopoiesis, Lymphoid Tissue Structure and Development, Tissue Development	Hematopoiesis of phagocytes	2.07E-03	Decreased	-2.485	45	ADAM10, ADAM17, APP, BTK, CCL23, CDA, CLEC4M, CSF1R, CSF3R, EPO, GMPR2, HOXA7, IFI16, IFNAR1, IL1B, IL1RN, IRF8, LILRA2, LILRB3, LTBR, LYN, MAPKAP1, MEF2C, MTOR, NOTCH2, PF4, PTEN, RBPJ, RFFL, S100A9, SH2B3, SP3, STAT3, TET2, THOC5, TLR2, TLR4, TLR5, TLR7, TNFRSF1A, TNFSF10, TREM1, VDR, ZBTB46, ZRSR2
Hematological Disease	Thrombocytosis	2.08E-03		1.767	18	ARNTL, ASXL1, BCL2L11, BNIP3L, CUX1, HBA1/HBA2, IFNAR1, JAK1, LILRB3, PDE4B, PDE8A, PF4, PTGS2, SF3B1, SH2B3, TET2, THBS2, U2AF1/U2AF1L5
Cell Morphology	Cell polarity formation	2.08E-03			18	APC, CRB1, CRKL, CTNNA1, CYP26B1, DOCK2, EPHB1, EZR, FEZ1, FZD3, GAB1, HSP90AA1, HSP90AB1, IGF1R, KIF26B, KRAS, LIMS1, SCRIB
Protein Synthesis	Synthesis of protein	2.09E-03		-1.055	106	ACO1, ADM, AGO2, ALDH3A1, ALKBH1, APP, ATF5, B4GALT1, BTG2, BTK, CASC3, CAV3, CDKL2, CNBP, CPEB1, CREB1, DDIT3, DDX3X, EEF2K, EIF1AX, EIF3A, EIF3G, EIF3I, EIF4G3, EIF4H, FOXO3, FTH1, FUS, GAB2, GAPDH, HCK, HELZ, HSPA1A/HSPA1B, HSPA5, ICAM1, IFNAR1, IGF1, IGF2BP3, IL1B, ILF3, IREB2, KRAS, LARP4, LARP4B, LARP6, let-7, LYN, MAP4K4, MARS1, MKNK1, MMP14, MRPL15, MRPL18, MRPL28, MRPL55, MRPS10, MRPS18A, MRRF, MTOR, MTRF1L, MTTP, MYBBP1A, NCBP1, NCL, NR3C1, OXA1L, PABPC1, PARK7, PDCD4, PHLPP1, PIWIL1, PPM1G, PRKAA1, PRL, PTCD3, PTEN, RBM4, RGS2, RNASET2, RPL13A, RPL18, RPL18A, RPL28, RPL38, RPL39, RPL4, RPL5, RPS15, S100A9, SAT1, SOD2, SRSF3, STAT3, STAU1, STIP1, SWAP70, SYK, TLR4, TNFSF10, TNIP1, VDR, VIM, WARS1, YBX1, YTHDF3, ZFPM2
Cellular Function and Maintenance, Molecular Transport, Small Molecule Biochemistry	Exocytosis of catecholamine	2.10E-03		0.239	7	CPLX2, ENTPD1, SNAP23, SNAP29, STX3, STXBP6, VAMP4
Connective Tissue Disorders, Developmental Disorder, Hematological Disease, Hereditary Disorder, Organismal Injury and Abnormalities	Familial erythrocytosis	2.10E-03			7	BPGM, EPO, HAMP, HBA1/HBA2, HBB, SH2B3, TRNT1
Cell Death and Survival, Skeletal and Muscular Disorders	Apoptosis of smooth muscle cells	2.11E-03	Decreased	-2.555	23	APP, CASP8, DDIT3, FOXO3, HMOX1, IGF1, IGF1R, IL1B, let-7, LIMS1, mir-138, PDCD4, PRKCD, PTEN, RHOA, RIPK3, SP1, STAT3, STK4, TLR4, TNFRSF1A, TNFSF10, XAF1
Cardiovascular Disease	Pericardial effusion	2.11E-03	Increased	2.53	15	ADM, CYP51A1, ECE1, F2R, HDAC7, NR3C1, RAMP2, RTEL1, TBX5, TFRC, TUBA1A, TUBA1C, TUBB2A, UBE4B, UBR2
Cell-To-Cell Signaling and Interaction, Cellular Compromise	Respiratory burst of cells	2.11E-03	Decreased	-2.09	14	APP, CD14, CXCL1, FPR1, HCK, ICAM1, IRF8, ITGA4, LILRB3, LYN, PF4, SYK, TREM1, TYROBP
Organismal Development	Growth of vessel	2.12E-03		-1.679	55	ADM, AIF1, ALOX5AP, ARNT, CAMK2A, CCDC88A, CLEC1B, CNP, CNR1, CTSB, CX3CR1, CYBB, ELN, EPO, ERO1A, FRS2, HDAC9, HMOX1, HSPD1, IFNGR1, IGF1, IGF1R, IGFBP4, IL1B, let-7, LRRFIP1, MMP14, NCF2, NCOA1, NFE2L2, NOTCH2, PRKAA1, PRKG1, PRL,

						PTEN, PTGS2, PTPN6, RAP1A, RHOA, RIPK3, RTN4, S100A9, SIRPA, SKP2, SNAP23, SOD2, SP1, STAT3, STK4, SYK, TCF4, TLR2, TLR4, TNFRSF1A, TNFSF10
Cell Death and Survival	Cell death of bone marrow cell lines	2.13E-03		0.677	17	AOPEP, ASAH1, BID, CFLAR, EPO, IGF1, IGF1R, IRF8, MAX, NOTCH2, NTRK1, PAK2, PRKCD, PTPN6, RAF1, RIPK3, STAT3
Cell Death and Survival	Cell death of kidney cancer cell lines	2.16E-03		-1.197	15	BCL2L11, CASP8, CFLAR, CXCR3, DDIT3, DKK2, HMOX1, HOTAIR, LINC00887, NBR2, PRKAR1A, SH3RF1, STAT3, TNFSF10, TXNRD1
Cancer, Neurological Disease, Organismal Injury and Abnormalities, Tumor Morphology	Progressive central nervous system tumor	2.16E-03			16	CSF1R, CTLA4, HSP90AA1, HSP90AB1, HSP90B1, MS4A1, NR3C1, NTRK1, OAZ1, PDGFRA, RAF1, STAT3, TUBA1A, TUBA1C, TUBB2A, YWHAZ
Cancer, Organismal Injury and Abnormalities, Respiratory Disease	Neoplasm of pleura	2.21E-03			20	ADAM10, ARSD, CTLA4, DDX3X, FOXO3, HSP90AA1, HSP90AB1, HSP90B1, KRAS, LYN, MYBBP1A, PDGFRA, PDZD8, SETDB1, SP1, THBS2, TTF1, TUBA1A, TUBA1C, TUBB2A
Cancer, Hematological Disease, Immunological Disease, Organismal Injury and Abnormalities	Refractory BCR-ABL E255K-positive Philadelphia-positive acute lymphoblastic leukemia	2.24E-03			10	CSF1R, HCK, LYN, MS4A1, NR3C1, PDGFRA, STK24, TUBA1A, TUBA1C, TUBB2A
Cancer, Hematological Disease, Immunological Disease, Organismal Injury and Abnormalities	Relapsed BCR-ABL F359V-positive Philadelphia-positive acute lymphoblastic leukemia	2.24E-03			10	CSF1R, HCK, LYN, MS4A1, NR3C1, PDGFRA, STK24, TUBA1A, TUBA1C, TUBB2A
Cancer, Organismal Injury and Abnormalities, Reproductive System Disease	Endometrial clear cell carcinoma	2.24E-03			10	CSF3R, FAM136A, HSP90AA1, HSP90AB1, HSP90B1, KRAS, SPOP, TUBA1A, TUBA1C, TUBB2A
Cancer, Hematological Disease, Immunological Disease, Organismal Injury and Abnormalities	Refractory BCR-ABL F359C-positive Philadelphia-positive acute lymphoblastic leukemia	2.24E-03			10	CSF1R, HCK, LYN, MS4A1, NR3C1, PDGFRA, STK24, TUBA1A, TUBA1C, TUBB2A
Cancer, Hematological Disease, Immunological Disease, Organismal Injury and Abnormalities	Relapsed BCR-ABL E255V-positive Philadelphia-positive acute lymphoblastic leukemia	2.24E-03			10	CSF1R, HCK, LYN, MS4A1, NR3C1, PDGFRA, STK24, TUBA1A, TUBA1C, TUBB2A
Cancer, Gastrointestinal	Anal carcinoma	2.24E-03			10	HSP90AA1, HSP90AB1, HSP90B1, KRAS, MTOR, NFE2L2, SERPINB3, TUBA1A, TUBA1C, TUBB2A

Disease, Organismal Injury and Abnormalities						
Cancer, Hematological Disease, Immunological Disease, Organismal Injury and Abnormalities	Refractory BCR-ABL F359V-positive Philadelphia-positive acute lymphoblastic leukemia	2.24E-03			10	CSF1R, HCK, LYN, MS4A1, NR3C1, PDGFRA, STK24, TUBA1A, TUBA1C, TUBB2A
Cancer, Hematological Disease, Immunological Disease, Organismal Injury and Abnormalities	Refractory BCR-ABL E255V-positive Philadelphia-positive acute lymphoblastic leukemia	2.24E-03			10	CSF1R, HCK, LYN, MS4A1, NR3C1, PDGFRA, STK24, TUBA1A, TUBA1C, TUBB2A
Cancer, Hematological Disease, Immunological Disease, Organismal Injury and Abnormalities	Relapsed BCR-ABL F359C-positive Philadelphia-positive acute lymphoblastic leukemia	2.24E-03			10	CSF1R, HCK, LYN, MS4A1, NR3C1, PDGFRA, STK24, TUBA1A, TUBA1C, TUBB2A
Cancer, Hematological Disease, Immunological Disease, Organismal Injury and Abnormalities	Relapsed BCR-ABL F359I-positive Philadelphia-positive acute lymphoblastic leukemia	2.24E-03			10	CSF1R, HCK, LYN, MS4A1, NR3C1, PDGFRA, STK24, TUBA1A, TUBA1C, TUBB2A
Cell Death and Survival	Apoptosis of macrophage cancer cell lines	2.24E-03		-0.965	11	BCL2L11, CASP8, CFLAR, DDIT3, FOXO3, HSP90AB1, MCL1, MVP, TLR4, TNFRSF1A, TREM1
Cancer, Hematological Disease, Immunological Disease, Organismal Injury and Abnormalities	Refractory BCR-ABL F359I-positive Philadelphia-positive acute lymphoblastic leukemia	2.24E-03			10	CSF1R, HCK, LYN, MS4A1, NR3C1, PDGFRA, STK24, TUBA1A, TUBA1C, TUBB2A
Cancer, Hematological Disease, Immunological Disease, Organismal Injury and Abnormalities	Refractory BCR-ABL Y253H-positive Philadelphia-positive acute lymphoblastic leukemia	2.24E-03			10	CSF1R, HCK, LYN, MS4A1, NR3C1, PDGFRA, STK24, TUBA1A, TUBA1C, TUBB2A
Hematological Disease, Immunological Disease	Lymphoid immunodeficiency	2.24E-03			10	BCL2L11, CASP8, CTLA4, EXTL3, FOXP3, KRAS, MAGT1, PRKCD, STK4, TRNT1

Cell-To-Cell Signaling and Interaction, Hematological System Development and Function, Hypersensitivity Response, Inflammatory Response	Binding of mast cells	2.24E-03		-1.671	10	BTK, F2R, HCK, ICAM1, IL1B, ITGA4, LYN, NOTCH2, PAK2, RAC2
Cancer, Hematological Disease, Immunological Disease, Organismal Injury and Abnormalities	Relapsed BCR-ABL E255K-positive Philadelphia-positive acute lymphoblastic leukemia	2.24E-03			10	CSF1R, HCK, LYN, MS4A1, NR3C1, PDGFRA, STK24, TUBA1A, TUBA1C, TUBB2A
Cancer, Hematological Disease, Immunological Disease, Organismal Injury and Abnormalities	Relapsed BCR-ABL Y253H-positive Philadelphia-positive acute lymphoblastic leukemia	2.24E-03			10	CSF1R, HCK, LYN, MS4A1, NR3C1, PDGFRA, STK24, TUBA1A, TUBA1C, TUBB2A
Cell Morphology	Area of cells	2.25E-03			22	APP, C10orf71, CNP, CUX1, DYRK1A, HOTAIR, IGF1, IGF1R, IL1B, ITM2B, MYO5A, NLGN3, NR3C1, NTRK1, PF4, PLP1, PRKCD, PTEN, SCARB2, TJP1, TLR4, VDR
Cell-To-Cell Signaling and Interaction, Hematological System Development and Function, Hematopoiesis	Binding of hematopoietic progenitor cells	2.30E-03	Decreased	-2.791	12	BTK, CXCL9, CXCR3, F2R, FYB1, ICAM1, IRF8, ITGA4, PECAM1, PRL, RAC2, RHOA
Developmental Disorder, Hereditary Disorder, Metabolic Disease, Organismal Injury and Abnormalities	Glycogen storage disease	2.30E-03			12	ENO3, FCGR2A, FCGR2C, FOXP3, GBE1, GYG1, M6PR, MS4A1, PGAM2, PHKB, PSMD1, PSMD2
Cancer, Hematological Disease, Immunological Disease, Organismal Injury and Abnormalities	CD20 positive aggressive mature B-cell lymphoma	2.30E-03			12	BTK, CSF3R, HSP90AA1, HSP90AB1, HSP90B1, MS4A1, NR3C1, PSMD1, PSMD2, TUBA1A, TUBA1C, TUBB2A
Cellular Assembly and Organization	Rearrangement of actin cytoskeleton	2.30E-03	Decreased	-2.009	12	DEF6, FPR1, FYB1, IGF1, ITGA4, PACSIN2, PIP5K1A, PLXNA4, RAB5A, RAC2, RHOA, RICTOR
Cell-To-Cell Signaling and Interaction	Response of neuroglia	2.30E-03		-1.844	12	APP, BECN1, CD14, DOCK2, HMOX1, IL1B, LILRB3, MERTK, PARK7, S100A9, TLR2, TLR4

Cancer, Organismal Injury and Abnormalities, Reproductive System Disease	Cervical cancer	2.31E-03			79	ALDH5A1, ANXA2, ANXA5, APOBEC3B, ARHGDIB, CASP8, CDKL5, CLEC4F, CLEC4M, CSF1R, CTSB, CTSC, DPYD, EIF3A, F10, FOXO3, GOLGB1, HBA1/HBA2, HBB, HLA-A, HSP90AA1, HSP90AB1, HSP90B1, IGF1, ITGA4, ITGAX, ITGB8, KRAS, let-7, MAP4, MAX, MCM7, MDM2, mir-133, mir-154, mir-202, mir-24, mir-26, mir-28, mir-515, MTOR, MYO15A, NEDD9, NOTCH2, NUBP1, NUP62, OR2A14, PITPNA, PSME3, PTEN, PTGFRN, PTGS2, RAMP2, RTTN, SERPINB3, SERPINB4, SF3B1, SMARCC2, STAT3, TET2, TFRC, THBS2, TLR7, TPM3, TRIO, TRIOBP, TTF1, TUBA1A, TUBA1C, TUBB2A, U2AF1/U2AF1L5, UGT2B11, USP4, VCAN, VIM, YWHAE, ZEB2, ZNF350, ZNF677
Infectious Diseases, Inflammatory Disease, Organismal Injury and Abnormalities, Respiratory Disease	Severe acute respiratory syndrome	2.31E-03			25	ACSL1, ACTN1, CTSZ, F10, FOXO3, FPR1, G3BP2, GAPDH, GLUL, GYG1, H2AC18/H2AC19, H2BC12, H2BC21, IMPA2, IRF8, ITGA4, KRAS, LILRA2, MXD1, NR3C1, RAB31, S100A9, SH2B3, TALDO1, TLR2
Cancer, Hematological Disease, Immunological Disease, Organismal Injury and Abnormalities	Mature B-cell neoplasm	2.33E-03		-0.246	238	AIG1, AMPH, ANKLE2, ANO5, ANXA2, ANXA5, APBA1, APC, APOBEC3A, APOBEC3B, APP, ARHGAP17, ARL6IP5, ASB10, ASMTL, ASXL1, ATN1, ATRN, ATXN3, B2M, BAZ2B, BBS7, BCL2L11, BCL7A, BECN1, BOD1L1, BRCA1, BTG2, BTK, CARMIL1, CARNS1, CASP8, CAV3, CAVIN2, CCL5, CCNDBP1, CD300E, CD86, CDC23, CFLAR, CGB1/CGB2, CHD4, CKMT2, CMSS1, CNR1, COL1A2, COL7A1, CPSF7, CRNN, CSDE1, CSF1R, CSF3R, CTNND2, CTSC, CXCL1, CXCL6, CXCL9, CYP2A6 (includes others), DMTF1, DNAJB14, DOCK2, DPYD, DYRK1A, EPHA8, ETV6, EWSR1, F10, F11R, FAM131C, FCGR2A, FOXO3, FRMD4B, FUS, FYB1, FZD3, GATA5, GPD1, GPRIN1, GPSM2, GRB2, GSE1, HAO2, HCLS1, HDAC9, HECA, HLA-A, HLA-G, HMOX1, HSP90AA1, HSP90AB1, HSP90B1, HVCN1, IDH3A, IFNAR1, IGF1, IGF2R, IL1B, IL1RN, IRF8, ITGA4, ITGAX, JAK1, KAT6A, KRAS, LCP1, LCP2, LCT, let-7, LHCGR, LRP2, LRRFIP1, LSM3, LYN, LYPLA2, MAP4K4, MAX, MCL1, MDM2, mir-138, mir-154, mir-28, MLX, MPEG1, MS4A1, MTOR, MYO15A, MYO5B, MYOF, NACA2, NAMPT, NDC80, NDUFS1, NETO2, NFE2L2, NONO, NOTCH2, NR3C1, NUBP1, NUDT6, NXPE4, PCLO, PDGFRA, PECAM1, PF4, PLCG2, PLEKHA7, POLR3B, POTEH (includes others), PPM1D, PPP1R12B, PPP4R2, PPP6C, PPP6R3, PRKAA1, PSMB8, PSMD1, PSMD2, PSME3, PTEN, PTGFRN, PTGS2, PTPRE, PWWP3A, RAB38, RAB4A, RAF1, RBM4, RESF1, RHOA, RICTOR, RPS15, RTTN, S100A9, SCN9A, SCRT2, SEC14L1, SEC24D, SF3B1, SHROOM3, SLAMF7, SLC25A32, SMARCA2, SNRPD3, SOD2, SORL1, SPAG9, SRPK2, STAT3, STEAP4, STIP1, STXBP6, SUCLG1, SWAP70, SYK, TDRD1, TET2, TFRC, THBS2, TLR2, TLR4, TLR5, TLR7, TMEM45B, TMPRSS9, TNFRSF10C, TNIP1, TNNI3K, TOX4, TRAF3, TRIM5, TRIM55, TRIO, TRIP12, TRPM6, TTC21B, TUBA1A, TUBA1C, TUBB2A, U2AF1/U2AF1L5, UBA3, UBE2F, UBE2J2, UNC5C, VCAN, VDAC1, VIM, YAE1, YWHAE, YWHAZ, ZMYM3, ZNF10, ZNF211, ZNF212, ZNF229, ZNF615, ZNF700, ZNF714, ZRSR2
Cancer, Hematological Disease, Immunological Disease, Organismal Injury and Abnormalities	Refractory CD20 positive diffuse large B-cell non-Hodgkin lymphoma	2.33E-03			8	CSF3R, HSP90AA1, HSP90AB1, HSP90B1, MS4A1, NR3C1, PSMD1, PSMD2
Cancer, Hematological Disease, Immunological Disease, Organismal Injury and Abnormalities	CD20 positive refractory non Hodgkin lymphoma	2.35E-03			9	BTK, CSF3R, HSP90AA1, HSP90AB1, HSP90B1, MS4A1, NR3C1, PSMD1, PSMD2
Cell-To-Cell Signaling and Interaction, Cellular Function and Maintenance, Hematological System Development and Function, Inflammatory Response	Phagocytosis by microglia	2.35E-03		-1.407	9	APP, BECN1, CD14, DOCK2, IL1B, MERTK, S100A9, TLR2, TLR4

Cancer, Hematological Disease, Immunological Disease, Organismal Injury and Abnormalities	Refractory CD20-positive B-cell non-Hodgkin lymphoma	2.35E-03			9	BTK, CSF3R, HSP90AA1, HSP90AB1, HSP90B1, MS4A1, NR3C1, PSMD1, PSMD2
Cancer, Hematological Disease, Immunological Disease, Organismal Injury and Abnormalities	Refractory BCR-ABL F317L-positive Philadelphia-positive acute lymphoblastic leukemia	2.35E-03			9	CSF1R, HCK, LYN, MS4A1, NR3C1, STK24, TUBA1A, TUBA1C, TUBB2A
Cancer, Hematological Disease, Immunological Disease, Organismal Injury and Abnormalities	Refractory BCR-ABL F317I-positive Philadelphia-positive acute lymphoblastic leukemia	2.35E-03			9	CSF1R, HCK, LYN, MS4A1, NR3C1, STK24, TUBA1A, TUBA1C, TUBB2A
Cancer, Hematological Disease, Immunological Disease, Organismal Injury and Abnormalities	High-risk CD20 positive diffuse large B-cell non-Hodgkin lymphoma	2.35E-03			9	CSF3R, HSP90AA1, HSP90AB1, HSP90B1, MS4A1, NR3C1, TUBA1A, TUBA1C, TUBB2A
Cancer, Hematological Disease, Immunological Disease, Organismal Injury and Abnormalities	Relapsed BCR-ABL F317V-positive Philadelphia-positive acute lymphoblastic leukemia	2.35E-03			9	CSF1R, HCK, LYN, MS4A1, NR3C1, STK24, TUBA1A, TUBA1C, TUBB2A
Cancer, Hematological Disease, Immunological Disease, Organismal Injury and Abnormalities	Relapsed BCR-ABL F317I-positive Philadelphia-positive acute lymphoblastic leukemia	2.35E-03			9	CSF1R, HCK, LYN, MS4A1, NR3C1, STK24, TUBA1A, TUBA1C, TUBB2A
Cancer, Hematological Disease, Immunological Disease, Organismal Injury and Abnormalities	Relapsed BCR-ABL T315A-positive Philadelphia-positive acute lymphoblastic leukemia	2.35E-03			9	CSF1R, HCK, LYN, MS4A1, NR3C1, STK24, TUBA1A, TUBA1C, TUBB2A
Cancer, Hematological Disease, Immunological	Refractory BCR-ABL T315A-positive Philadelphia-	2.35E-03			9	CSF1R, HCK, LYN, MS4A1, NR3C1, STK24, TUBA1A, TUBA1C, TUBB2A

Disease, Organismal Injury and Abnormalities	positive acute lymphoblastic leukemia					
Cancer, Hematological Disease, Immunological Disease, Organismal Injury and Abnormalities	Relapsed BCR-ABL F317C-positive Philadelphia-positive acute lymphoblastic leukemia	2.35E-03			9	CSF1R, HCK, LYN, MS4A1, NR3C1, STK24, TUBA1A, TUBA1C, TUBB2A
Cancer, Hematological Disease, Immunological Disease, Organismal Injury and Abnormalities	Relapsed BCR-ABL F317L-positive Philadelphia-positive acute lymphoblastic leukemia	2.35E-03			9	CSF1R, HCK, LYN, MS4A1, NR3C1, STK24, TUBA1A, TUBA1C, TUBB2A
Cancer, Hematological Disease, Immunological Disease, Organismal Injury and Abnormalities	Refractory BCR-ABL F317C-positive Philadelphia-positive acute lymphoblastic leukemia	2.35E-03			9	CSF1R, HCK, LYN, MS4A1, NR3C1, STK24, TUBA1A, TUBA1C, TUBB2A
Cancer, Hematological Disease, Immunological Disease, Organismal Injury and Abnormalities	Refractory BCR-ABL F317V-positive Philadelphia-positive acute lymphoblastic leukemia	2.35E-03			9	CSF1R, HCK, LYN, MS4A1, NR3C1, STK24, TUBA1A, TUBA1C, TUBB2A
Cardiovascular System Development and Function, Cellular Movement	Migration of endothelial cells	2.37E-03	Decreased	-3.516	75	ADAM10, ADAM15, ADAM17, ADGRG3, ADM, ANGPTL4, ANXA2, ANXA3, APC, APOA1, ARNT, BCAS3, CAVIN2, CCL5, CLEC1B, CXCL1, CXCR2, ELN, EMC10, EPO, EYA3, F11R, F2R, FOXO3, FRS2, GAB1, GLUL, HSP90AB1, HSPA5, ICAM1, IGF1, IGF2R, IL1B, ITGA4, KIF26B, KLHL20, MAX, MDM2, MEF2C, mir-133, mir-137, mir-24, mir-26, MMP14, MTOR, MXD1, NCL, NFE2L2, PAQR3, PECAM1, PF4, PRKAA1, PRKG1, PROK2, PRSS55, PTEN, PTGS2, PTPN6, RGCC, RHOA, RHOB, RIN2, RTN4, SFRP4, SP1, SP100, STAT3, STK35, TAZ, TDGF1, TJP1, TNFSF10, VIM, WARS1, YWHAZ
Cellular Development, Cellular Growth and Proliferation	Proliferation of lung cancer cell lines	2.44E-03	Decreased	-2.922	64	ADAM17, ANXA2, BID, CREB1, CRKL, CYP2J2, DAB2, DDX3X, DNAJB4, DPF2, DUSP5, ELF3, EPB41L3, FAIM2, FOXO3, GAPDH, HMOX1, HNRNPA2B1, IGF1, IGF1R, IL1B, ITGB8, KDM5A, KRAS, let-7, LINC00511, LUCAT1, MCL1, MDM2, mir-103, mir-154, mir-24, mir-515, MTOR, MUC1, MYH14, NASP, NFE2L2, NOTCH2, NUMB, PDGFRA, PRKCD, PRRC2C, PTEN, PTGS2, RAF1, RALB, RALBP1, RASD1, RHOB, SETDB1, SKP2, SMARCA2, STAT3, TASP1, TAZ, TBXAS1, THBS2, TLR2, TLR5, TNFSF10, TTF1, TUBB2A, YWHAZ
Cancer, Organismal Injury and Abnormalities	Medullary carcinoma	2.45E-03			13	AMY1C (includes others), CSF1R, KRAS, MAPKAP1, MERTK, NR3C1, NTRK1, PDGFRA, PTEN, PTGS2, RAF1, STAT3, TBC1D9
Nervous System Development and Function	Neuroprotection of hippocampus	2.47E-03		-1.318	6	APP, EPO, IGF1, PTGS2, STAT3, STIP1
Cellular Movement, Hematological System Development and Function, Immune Cell Trafficking	Cell movement of lymphoblasts	2.47E-03	Decreased	-2.213	6	CCL5, DEFB103A/DEFB103B, HCLS1, ICAM1, SOS2, STK4

Supplementary Table 4: Table illustrates significantly (p-value<0.05) enriched diseases and functions from ΔD64 differentially expressed gene list analysis using IPA®. For each disease and function term the cluster category is reported together with p-value, z-score, predicted activation state (z-score>2= increased activation, z-score<-2=decreased activation), number of transcripts and their IDs. Data were analyzed through the use of IPA (QIAGEN Inc., https://www.qiagenbioinformatics.com/products/ingenuitypathway-analysis)

Categories	Diseases or Functions Annotation	p-value	Predicted Activation State	Activation z-score	# genes	Genes
Cell-To-Cell Signaling and Interaction, Cellular Growth and Proliferation, Hematological System Development and Function	Induction of mononuclear leukocytes	9.96E-05		0.896	8	CAT, CRH, CSF1, CTLA4, KLRC1, MERTK, MUC1, PRKG1
Hematological Disease, Immunological Disease	Eosinophilia of tissue	1.25E-04		0.762	4	IL1RL1, IL9, PTGDR2, SIGLEC8
Molecular Transport	Transmembrane transport of ion	1.97E-04			17	ANO5, ATP6V0A2, ATP6V0D2, ATP6V1A, BSND, CACNA1E, GLRA1, HCN3, KCNG3, KCNH2, SCN4B, SLC17A3, SLC46A1, SLC8A3, SLC9B1, STOML3, TRPC6
Cell-To-Cell Signaling and Interaction, Hematological System Development and Function, Hypersensitivity Response, Immune Cell Trafficking, Inflammatory Response	Activation of eosinophils	2.29E-04			5	CXCL9, IL1RL1, IL5RA, IL9, PTGDR2
Cell Death and Survival	Killing of Candida albicans	2.48E-04			3	CCL28, CFHR1, PRTN3
Cellular Movement, Reproductive System Development and Function	Cell movement of sperm	2.65E-04			10	ANO5, APOB, CACNA1E, DDHD1, MET, PRSS55, SLC9B1, SORD, SPAG16, VPS13A
Nervous System Development and Function, Organ Morphology, Tissue Morphology, Visual System Development and Function	Quantity of starburst amacrine cells	3.84E-04			2	BARHL2, PTF1A

Cellular Development, Cellular Growth and Proliferation, Hematological System Development and Function, Lymphoid Tissue Structure and Development	Proliferation of tumor-infiltrating lymphocytes	3.84E-04			2	IDO1, IL9
Cancer, Gastrointestinal Disease, Organismal Injury and Abnormalities, Respiratory Disease	Nasal polyp	3.90E-04			3	CYSLTR2, IL5RA, PTGDR2
Endocrine System Development and Function, Molecular Transport, Small Molecule Biochemistry	Secretion of peptide hormone derivative	4.94E-04		-1.982	5	CACNA1E, CRH, FFAR4, GPER1, SMPD3
Cancer, Organismal Injury and Abnormalities, Renal and Urological Disease	Metastatic kidney carcinoma	5.22E-04			6	CTLA4, IDO1, IL2RA, MERTK, MET, MUC1
Cell-To-Cell Signaling and Interaction, Cellular Growth and Proliferation	Induction of lymphatic system cells	5.23E-04		0.447	7	CRH, CSF1, CTLA4, KLRC1, MERTK, MUC1, PRKG1
Cell-mediated Immune Response, Cellular Development, Cellular Function and Maintenance, Cellular Growth and Proliferation, Embryonic Development, Hematological System Development and Function, Hematopoiesis, Lymphoid Tissue Structure and Development, Organ Development, Organismal	Differentiation of follicular T helper cells	6.71E-04		0.784	4	CTLA4, GPR183, IL2RA, MERTK

Development, Tissue Development						
Cell-To-Cell Signaling and Interaction, Cellular Growth and Proliferation, Hematological System Development and Function	Suppression of TREG cells	6.71E-04			4	CTLA4, FOXP3, IDO1, IL9
Cancer, Organismal Injury and Abnormalities, Renal and Urological Disease	Advanced kidney carcinoma	7.08E-04			6	CTLA4, IDO1, IL2RA, MERTK, MET, MUC1
Cancer, Organismal Injury and Abnormalities, Renal and Urological Disease	Advanced renal cancer	7.08E-04			6	CTLA4, IDO1, IL2RA, MERTK, MET, MUC1
Cellular Development, Cellular Growth and Proliferation, Connective Tissue Development and Function, Hematological System Development and Function, Hematopoiesis, Organismal Development, Tissue Development	Maturation of erythroid precursor cells	8.08E-04			4	ALOX15, FOXO3, IL9, RNF112
Cell-To-Cell Signaling and Interaction, Hematological System Development and Function, Immune Cell Trafficking, Inflammatory Response	Activation of regulatory T lymphocytes	8.36E-04		0.555	5	CD2, CLC, CTLA4, FOXP3, MERTK
Cell-To-Cell Signaling and Interaction	Signal transduction	9.36E-04			54	ADGRE1, ATOH8, CCL23, CCL27, CCL28, CCR3, CD101, CD2, CGB3 (includes others), CORO2A, CRH, CXCL9, CYSLTR2, FFAR4, GHRHR, GNG4, GPER1, GPR183, HCAR1, HRH4, IL1RL1, IL2RA, IL5RA, KLRC1, LGR5, MERTK, MET, MOK, NAMPT, NPY4R/NPY4R2, OR10A6, OR12D3, OR2A14, OR2D2, OR2V2, OR2Y1, OR4K5, OR5H2, OR9Q1, P2RY10, P2RY14, PIK3R6, PMCH, PRKG1, PTGDR2, RGS1, RXFP2, SIGLEC8, SMAD5, SMPD3, SRI, STOML3, TLE1, VLDLR
Endocrine System Development and Function, Organ	Morphology of pituitary gland	1.13E-03			6	ARNT2, CDKN2C, CGB3 (includes others), FOXL2, GSX1, SMPD3

Morphology, Organismal Development						
Cell-mediated Immune Response, Cellular Movement, Hematological System Development and Function, Immune Cell Trafficking, Lymphoid Tissue Structure and Development	Homing of helper T lymphocytes	1.14E-03			4	CCL27, CCR3, FOXP3, PTGDR2
Cancer, Endocrine System Disorders, Hereditary Disorder, Organismal Injury and Abnormalities	Familial thyroid carcinoma	1.14E-03			4	HABP2, MERTK, MET, MSH2
Cancer, Endocrine System Disorders, Hereditary Disorder, Organismal Injury and Abnormalities	Hereditary thyroid cancer	1.14E-03			4	HABP2, MERTK, MET, MSH2
Nucleic Acid Metabolism, Small Molecule Biochemistry	Depletion of NADPH	1.14E-03			2	CD38, MET
Cancer, Cellular Development, Organismal Injury and Abnormalities, Tumor Morphology	Transdifferentiation of tumor	1.14E-03			2	CGB3 (includes others), MUC1
Cell-To-Cell Signaling and Interaction, Cellular Compromise	Oxidative stress response of islet cells	1.14E-03			2	CAT, HFE
Embryonic Development, Nervous System Development and Function, Organ Development, Organ Morphology, Organismal Development, Tissue Development, Visual System Development and Function	Thickness of cornea	1.14E-03			2	AQP5, CHST6
Hematological Disease,	Eosinophilia of bone marrow	1.14E-03			2	IL9, SIGLEC8

Immunological Disease						
Amino Acid Metabolism, Small Molecule Biochemistry	Binding of L-amino acid	1.14E-03			2	CAT, IDO1
Amino Acid Metabolism, Post-Translational Modification, Small Molecule Biochemistry	Modification of glycine	1.14E-03			2	GATM, GLDC
Embryonic Development, Organ Development, Organismal Development, Reproductive System Development and Function, Tissue Development	Development of lobules of mammary gland	1.14E-03			2	CGB3 (includes others), ID2
Amino Acid Metabolism, Small Molecule Biochemistry	Binding of aromatic amino acid	1.14E-03			2	CAT, IDO1
Cancer, Cell Death and Survival, Organismal Injury and Abnormalities, Tumor Morphology	Cell death of acute myeloid leukemia blast cells	1.14E-03			2	MUC1, RPS3A
Cellular Function and Maintenance	Ion homeostasis of cells	1.21E-03		1.269	30	AQP5, ATP6V0A2, ATP6V1A, BSND, CCL23, CCL28, CCR3, CD101, CD2, CD24, CD38, CLIC5, CRH, CTLA4, FFAR4, GLRA1, GPER1, GPR183, HFE, HTR3A, HTR3C, IL1RL1, KLHL3, MON1A, PMCH, PRKG1, SLC46A1, SLC8A3, TEC, TRPC6
Immunological Disease, Inflammatory Response	Abnormal inflammatory response	1.23E-03			6	CRH, FOXP3, GCNT1, LUM, MSH2, PTGDR2
Inflammatory Response	Function of immune system	1.27E-03			10	ALOX15, CCR3, CYSLTR2, GCNT1, HRH4, ID2, IL1RL1, IL5RA, IL9, SIGLEC8
Organ Morphology	Size of secretory structure	1.47E-03			8	AQP5, CDKN2C, CGB3 (includes others), FOXL2, HFE, NFIB, PMCH, PRLR
Cancer, Organismal Injury and Abnormalities, Renal and Urological Disease	Metastatic renal clear cell adenocarcinoma	1.48E-03			5	CTLA4, IL2RA, MERTK, MET, MUC1
Cell-mediated Immune Response, Cellular Development, Cellular Function	Differentiation of effector T lymphocytes	1.55E-03		0.882	4	CTLA4, FOXP3, ID2, IL2RA

and Maintenance, Cellular Growth and Proliferation, Embryonic Development, Hematological System Development and Function, Hematopoiesis, Lymphoid Tissue Structure and Development, Organ Development, Organismal Development, Tissue Development						
Endocrine System Development and Function, Molecular Transport, Small Molecule Biochemistry	Secretion of hormone	1.63E-03		-0.842	9	ARNT2, CACNA1E, CGB3 (includes others), CRH, FFAR4, GHRHR, GPER1, GSX1, SMPD3
Drug Metabolism, Endocrine System Development and Function, Molecular Transport, Small Molecule Biochemistry	Secretion of glucagon	1.79E-03		-1.982	4	CACNA1E, CRH, FFAR4, GPER1
Cellular Function and Maintenance, Hematological System Development and Function	Function of regulatory T lymphocytes	1.82E-03			5	ADGRE1, CTLA4, FOXP3, IL2RA, IL9
Endocrine System Disorders, Gastrointestinal Disease, Hereditary Disorder, Metabolic Disease, Organismal Injury and Abnormalities	Neonatal diabetes mellitus	1.85E-03			3	FOXP3, GATA6, PTF1A
Cellular Development, Digestive System Development and Function	Differentiation of enterocytes	1.85E-03			3	GATA5, GATA6, ID2
Cell-To-Cell Signaling and Interaction, Cellular	Suppressive capacity of TREG cells	1.85E-03			3	CTLA4, IDO1, IL9

Growth and Proliferation, Hematological System Development and Function						
Cell-To-Cell Signaling and Interaction, Cellular Growth and Proliferation	Stimulation of leukemia cell lines	1.85E-03			3	CD2, CSF1, IL9
Hematological Disease, Immunological Disease	Eosinophilia	1.97E-03		1.605	12	ALOX15, CCR3, CLC, CXCL9, IL1RL1, IL2RA, IL5RA, IL9, PMCH, PTGDR2, RNASE2, SIGLEC8
Cell-To-Cell Signaling and Interaction	Communication of cells	1.99E-03		-0.922	56	ADGRE1, ATOH8, CCL23, CCL27, CCL28, CCR3, CD101, CD2, CGB3 (includes others), CORO2A, CRH, CTLA4, CXCL9, CYSLTR2, FFAR4, GHRHR, GNG4, GPER1, GPR183, HCAR1, HRH4, IL1RL1, IL2RA, IL5RA, KLRC1, LGR5, MERTK, MET, MOK, NAMPT, NPY4R/NPY4R2, OR10A6, OR12D3, OR2A14, OR2D2, OR2V2, OR2Y1, OR4K5, OR5H2, OR9Q1, P2RY10, P2RY14, PIK3R6, PMCH, PRKG1, PTGDR2, RGS1, RXFP2, SIGLEC8, SMAD5, SMPD3, SRI, STOML3, THEM4, TLE1, VLDLR
Endocrine System Development and Function, Molecular Transport, Small Molecule Biochemistry	Concentration of hormone	2.02E-03		0.931	21	APOB, ARNT2, CACNA1E, CD38, CDKN2C, CGB3 (includes others), CRH, FFAR4, FOXL2, FOXO3, GATA6, GATM, GPER1, LGR5, PRKG1, PRLR, SERPINA6, SMPD3, SPR, SRGAP3, SRI
Cancer, Organismal Injury and Abnormalities, Reproductive System Disease	Development of mammary tumor	2.08E-03		0.478	10	CDKN2C, CGB3 (includes others), CSF1, CTLA4, FOXP3, GPR34, IL2RA, MET, PRLR, WEE1
Gastrointestinal Disease, Hematological Disease, Immunological Disease, Inflammatory Disease, Inflammatory Response, Organismal Injury and Abnormalities	Eosinophilia of esophagus	2.16E-03			6	ALOX15, CCR3, CLC, IL2RA, PMCH, SIGLEC8
Cell Signaling, Molecular Transport, Vitamin and Mineral Metabolism	Quantity of Ca2+	2.16E-03		1.838	21	CCR3, CD2, CD38, CRH, CSF1, CXCL9, CYSLTR2, FFAR4, FOXO3, GPER1, HRH4, KCNH2, PMCH, PMP22, PRKG1, PRLR, PTGDR2, RGS1, TEC, TNNC1, TRPC6
Cellular Assembly and Organization, Nervous System Development and Function	Complexity of apical processes	2.25E-03			2	HTR3A, MET

Cancer, Organismal Injury and Abnormalities, Renal and Urological Disease	Bilateral renal cell carcinoma	2.25E-03			2	CTLA4, MET
Cell Death and Survival	Survival of endometrial cancer cell lines	2.25E-03			2	CRH, MSH2
Auditory and Vestibular System Development and Function, Auditory Disease, Connective Tissue Development and Function, Connective Tissue Disorders, Organ Morphology, Organismal Development, Organismal Injury and Abnormalities, Skeletal and Muscular Disorders, Skeletal and Muscular System Development and Function, Tissue Development	Abnormal morphology of spiral ligament	2.25E-03			2	SLC4A7, SLC7A8
Cellular Compromise	Depletion of regulatory T lymphocytes	2.25E-03			2	CTLA4, IL2RA
Connective Tissue Disorders, Dermatological Diseases and Conditions, Developmental Disorder, Hereditary Disorder, Metabolic Disease, Organismal Injury and Abnormalities, Skeletal and Muscular Disorders	Autosomal recessive cutis laxa type 2	2.25E-03			2	ATP6V0A2, ATP6V1A
Digestive System Development and Function, Gastrointestinal Disease, Hepatic System Development and Function, Hepatic	Hepatitis A	2.25E-03			2	CSF1, IL2RA

System Disease, Infectious Diseases, Inflammatory Disease, Inflammatory Response, Organ Development, Organismal Injury and Abnormalities						
Cell Cycle, Cell-To-Cell Signaling and Interaction, Cellular Growth and Proliferation	Contact growth inhibition of colorectal cancer cell lines	2.33E-03			3	CD24, FOXO3, PRKG1
Endocrine System Development and Function, Organ Morphology, Organismal Development, Reproductive System Development and Function	Size of pituitary gland	2.33E-03			3	CDKN2C, CGB3 (includes others), FOXL2
Cell-To-Cell Signaling and Interaction, Cellular Growth and Proliferation, Hematological System Development and Function	Induction of lymphocytes	2.33E-03		0	6	CRH, CTLA4, KLRC1, MERTK, MUC1, PRKG1
Organ Morphology	Morphology of gland	2.35E-03			22	AQP5, ARNT2, CD38, CDKN2C, CGB3 (includes others), CRH, ELOVL3, FFAR4, FOXL2, FOXP3, GATA6, GSX1, HFE, IL2RA, IL9, MET, NFIB, PMCH, PRLR, PTF1A, SMPD3, TFF3
Cancer, Organismal Injury and Abnormalities, Renal and Urological Disease	Unresectable renal cancer	2.35E-03			4	CTLA4, IDO1, IL2RA, MET
Cellular Movement, Hematological System Development and Function, Hypersensitivity Response, Immune Cell Trafficking	Cell movement of eosinophils	2.42E-03		1.687	8	CCL28, CCR3, CD2, CXCL9, IL1RL1, IL9, PTGDR2, SIGLEC8
Cellular Movement	Cell movement	2.71E-03		1.657	92	ADARB1, ADGRL3, AJAP1, ALOX15, ANO5, APOB, AQP5, ATOH8, BARHL2, CACNA1E, CAT, CCL23, CCL27, CCL28, CCR3, CD2, CD24, CD38, CDKN2B-AS1, CDKN2C, CFHR1, CGB3 (includes others), COL7A1, CRH, CSF1, CTLA4, CXCL9, CYP2C8, CYSLTR2, DDHD1, DLX3, FFAR4, FKBPL, FOXO3, FOXP3, GATA6, GCNT1, GPER1, GPR183, GPR34, HABP2, HOTAIR, HOXB9, HRH4, ID2, IDO1, IGF2BP3, IGLL1/IGLL5, IL1RL1, IL2RA, IL5RA, IL9, KCNH2, KLF17, KLRC1, LDB2, LIMCH1, LUM, MAPRE3, MEOX2, MERTK, MET, mir-183, mir-28,

						mir-31, mir-515, MUC1, NAMPT, NKD2, NOVA1, PIK3R6, PMCH, PMP22, PRKG1, PRLR, PRSS55, PRTN3, PTGDR2, RAPH1, RGS1, RNASE2, SIGLEC8, SLC7A8, SLC9B1, SMAD5, SORD, SPAG16, TFF3, TNS4, TRPC6, VLDLR, VPS13A
Hematological Disease, Immunological Disease, Inflammatory Disease	Eosinophilic inflammation	2.76E-03			9	ALOX15, CCR3, CLC, IL1RL1, IL2RA, IL5RA, IL9, PMCH, SIGLEC8
Cell-To-Cell Signaling and Interaction, Cellular Growth and Proliferation, Hematological System Development and Function	Stimulation of mononuclear leukocytes	2.78E-03		1	10	CAT, CD2, CD24, CRH, CSF1, CTLA4, KLRC1, MERTK, MUC1, PRKG1
Cancer, Organismal Injury and Abnormalities, Renal and Urological Disease	Stage IV metastatic renal clear cell cancer	2.86E-03			3	CTLA4, MERTK, MET
Cellular Function and Maintenance, Hematological System Development and Function, Hypersensitivity Response, Inflammatory Response	Function of eosinophils	2.86E-03			3	CCR3, IL1RL1, SIGLEC8
Embryonic Development, Organ Development, Organismal Development, Reproductive System Development and Function, Tissue Development	Lactation	2.89E-03			6	CSF1, GHRHR, HOXB9, ID2, PRLR, RXFP2
Hematological System Development and Function, Tissue Morphology	Quantity of granulocytes	2.91E-03		-1.534	18	ALOX15, B4GALNT2, CCL28, CCR3, CD101, CSF1, FOXP3, GCNT1, GSX1, IL1RL1, IL2RA, IL5RA, IL9, LUM, PIK3R6, PRTN3, PTGDR2, SIGLEC8
Cell-mediated Immune Response, Cellular Movement, Hematological System	Cell movement of helper T lymphocytes	2.91E-03		0.762	5	CCL27, CCR3, FOXP3, GCNT1, PTGDR2

Development and Function, Immune Cell Trafficking						
Cancer, Neurological Disease, Organismal Injury and Abnormalities	Glioblastoma	3.01E-03			22	ADARB1, CDKN2C, CSF1, CTLA4, CXCL9, EEF2K, FOXO3, FRRS1, GLDC, GPER1, HFE, IL2RA, IL9, KLRC1, LGR5, MAL, MERTK, MSH2, NAMPT, PMP22, RAPGEF5, TRPC6
Cell-To-Cell Signaling and Interaction, Cellular Movement, Hematological System Development and Function, Hypersensitivity Response, Immune Cell Trafficking	Recruitment of eosinophils	3.18E-03	Increased	2.17	5	CCR3, CSF1, CXCL9, IL9, PTGDR2
Cancer, Dermatological Diseases and Conditions, Organismal Injury and Abnormalities	Skin cancer	3.42E-03			217	ABCB5, ACSM3, ACSM5, ADARB1, ADGRE1, ADGRL3, ALDH7A1, ALOX15, ANO5, APOB, ARHGAP29, ARNT2, ARVCF, ASL, ATOH8, ATP6V0A2, B3GNT4, B4GALNT2, BATF2, BSND, C11orf88, C1QL2, CACNA1E, CAPSL, CASC1, CCL27, CCR3, CD101, CD2, CD38, CD96, CENPE, CFHR1, CHST6, CLC, CLINT1, COL7A1, CORO2A, CSF1, CT45A10/CT45A5, CT62, CTLA4, CXCL9, CYP2C8, CYSLTR2, DEFB119, DHX36, DLX3, DYNAP, DZIP3, EDDM3A, EEF2K, EFCAB5, EPN2, FAM227B, FAM47A, FFAR4, FOXO3, FRMD7, FSCB, GAPT, GASK1A, GCNT1, GHRHR, GLDC, GLRA1, GLYATL2, GPER1, GPR34, HABP2, HOXB8, HRH4, HS3ST6, HTR3A, HTR3C, IDO1, IGF2BP3, IGLL1/IGLL5, IKZF2, IL1RL1, IL2RA, IL5RA, IL9, ITM2C, KCNH2, KIAA1217, KLF12, KLF17, KLRC1, KLRC3, KRT33B, KRT36, LDB2, LGR5, LHFPL6, LILRA6, LIMCH1, LRRC17, LUM, MACC1, MAGEA11, MAGEC2, MEDAG, MEOX2, MERTK, MET, MKRN3, MON2, MS4A6E, MSH2, MUC1, NEK2, NFIB, NKAIN3, NKD2, NOVA1, NPHS2, NPY4R/NPY4R2, NRAP, OR10A6, OR10G8, OR10R2, OR12D3, OR2A14, OR2D2, OR2V2, OR2Y1, OR4K5, OR5F1, OR5H2, OR9Q1, P2RY10, P2RY14, PAPOLB, PARP6, PGAM2, PKLR, PNPLA7, PPP1R12B, PPP4R1, PRICKLE1, PRKG1, PRLR, PTF1A, PTGDR2, PTPN5, PWWP3B, PXDNL, RAPGEF5, RAPH1, RASIP1, RBAK, REM2, RFPL4B, RGS1, RNF112, RXFP2, SEL1L3, SERPINA6, SFMBT2, SIGLEC8, SIGLECL1, SIPA1L2, SLC15A5, SLC17A1, SLC17A3, SLC25A2, SLC25A38, SLC29A1, SLC36A3, SLC46A1, SLC4A7, SLC7A8, SLC8A3, SLC9B1, SMPD3, SNAP91, SPAG16, SPATA7, SPINDOC, SPINK5, SPNS3, SRGAP3, SSMEM1, SSX2/SSX2B, STARD8, STK19, STOML3, SYNE1, SYT17, SYT5, TBC1D8, TDRD9, TEC, TECTA, TENT5D, TEPP, TEX47, TFF3, TKTL2, TLCD3B, TLE1, TMEM108, TMEM132B, TMEM270, TMEM273, TMEM30B, TNS4, TRMT9B, TRPC6, VLDLR, VPS13A, VSTM1, WDR87, ZNF391, ZNF462, ZSCAN5A
Cell-To-Cell Signaling and Interaction, Cellular Growth and Proliferation, Hematological System Development and Function	Suppression of lymphocytes	3.46E-03	Increased	2.169	5	CRH, CTLA4, FOXP3, IDO1, IL9
Molecular Transport	Quantity of metal ion	3.54E-03		1.838	22	CCR3, CD2, CD38, CRH, CSF1, CXCL9, CYSLTR2, FFAR4, FOXO3, GPER1, HRH4, KCNH2, KLHL3, PMCH, PMP22, PRKG1, PRLR, PTGDR2, RGS1, TEC, TNNC1, TRPC6
Organ Morphology	Size of endocrine gland	3.54E-03			6	CDKN2C, CGB3 (includes others), FOXL2, HFE, PMCH, PRLR
Organ Morphology	Morphology of endocrine gland	3.55E-03			15	ARNT2, CD38, CDKN2C, CGB3 (includes others), CRH, FFAR4, FOXL2, GATA6, GSX1, HFE, MET, PMCH, PRLR, SMPD3, TFF3
Gastrointestinal Disease	Severe chemotherapy sickness	3.70E-03			2	HTR3A, HTR3C

Cell Cycle	Senescence of ovarian cancer cell lines	3.70E-03			2	HOTAIR, MET
Gastrointestinal Disease	Moderate chemotherapy sickness	3.70E-03			2	HTR3A, HTR3C
Cellular Function and Maintenance, Hematological System Development and Function	Function of natural T-regulatory cells	3.70E-03			2	FOXP3, IL9
Cellular Development, Tissue Development	Differentiation of Clara cells	3.70E-03			2	GATA6, NFIB
Cell-To-Cell Signaling and Interaction, Cellular Growth and Proliferation, Hematological System Development and Function, Inflammatory Response	Induction of monocytes	3.70E-03			2	CAT, CSF1
Gastrointestinal Disease, Organismal Injury and Abnormalities	Motility disorder of intestine	3.70E-03			2	HTR3A, HTR3C
Gastrointestinal Disease, Immunological Disease, Inflammatory Disease, Inflammatory Response, Organismal Injury and Abnormalities	Autoimmune metaplastic atrophic gastritis	3.70E-03			2	CTLA4, IL2RA
Cell Morphology, Cell-mediated Immune Response, Cellular Development, Cellular Function and Maintenance, Cellular Growth and Proliferation, Embryonic Development, Hematological System	Conversion of TREG cells	3.70E-03			2	FOXP3, IKZF2

Development and Function, Hematopoiesis, Lymphoid Tissue Structure and Development, Organ Development, Organismal Development, Tissue Development						
Developmental Disorder, Digestive System Development and Function, Endocrine System Disorders, Gastrointestinal Disease, Organ Morphology, Organismal Development, Organismal Injury and Abnormalities	Congenital pancreatic agenesis	3.70E-03			2	GATA6, PTF1A
Reproductive System Development and Function	Reproductive function	3.70E-03			2	CGB3 (includes others), CSF1
Cellular Development, Cellular Growth and Proliferation, Hematological System Development and Function, Humoral Immune Response, Lymphoid Tissue Structure and Development	Expansion of B-1a lymphocytes	3.70E-03			2	CDKN2C, GPR183
Protein Trafficking	Signaling of protein	3.70E-03			2	CTLA4, KLRC1
Cancer, Organismal Injury and Abnormalities	Malignant solid organ tumor	3.75E-03			220	ABCB5, ACSM3, ACSM5, ADARB1, ADGRE1, ADGRL3, ALDH7A1, ALOX15, ANO5, APOB, ARHGAP29, ARNT2, ARVCF, ASL, ATOH8, ATP6V0A2, ATXN7L2, B3GNT4, B4GALNT2, BATF2, BSND, C11orf88, C1QL2, CACNA1E, CAPSL, CASC1, CCL27, CCR3, CD101, CD2, CD38, CD96, CENPE, CFHR1, CHST6, CLC, CLINT1, COL7A1, CORO2A, CSF1, CT45A10/CT45A5, CT62, CTLA4, CXCL9, CYP2C8, CYSLTR2, DEFB119, DHX36, DLX3, DYNAP, DZIP3, EDDM3A, EEF2K, EFCAB5, EPN2, FAM227B, FAM47A, FFAR4, FOXO3, FRMD7, FSCB, GAPT, GASK1A, GCNT1, GHRHR, GLDC, GLRA1, GLYATL2, GPER1, GPR34, HABP2, HOXB8, HRCT1, HRH4, HS3ST6, HTR3A, HTR3C, IDO1, IGF2BP3, IGLL1/IGLL5, IKZF2, IL1RL1, IL2RA, IL5RA, IL9, ITM2C, KCNH2, KIAA1217, KLF12, KLF17, KLRC1, KLRC3, KRT33B, KRT36, LDB2, LGR5, LHFPL6, LILRA6, LIMCH1, LRRC17, LUM, MACC1, MAGEA11, MAGEC2, MEDAG, MEOX2, MERTK, MET, mir-183, MKRN3, MON2, MS4A6E, MSH2, MUC1, NEK2, NFIB, NKAIN3, NKD2, NOVA1, NPHS2, NPY4R/NPY4R2, NRAP, OR10A6, OR10G8, OR10R2, OR12D3, OR2A14, OR2D2, OR2V2, OR2Y1, OR4K5, OR5F1, OR5H2, OR9Q1, P2RY10, P2RY14, PAPOLB, PARP6, PGAM2, PKLR, PNPLA7, PPP1R12B, PPP4R1, PRICKLE1, PRKG1, PRLR, PTF1A, PTGDR2, PTPN5, PWWP3B, PXDNL, RAPGEF5, RAPH1, RASIP1, RBAK, REM2, RFPL4B, RGS1, RNF112, RXFP2, SEL1L3, SERPINA6, SFMBT2, SIGLEC8, SIGLECL1, SIPA1L2, SLC15A5, SLC17A1, SLC17A3, SLC25A2,

						SLC25A38, SLC29A1, SLC36A3, SLC46A1, SLC4A7, SLC7A8, SLC8A3, SLC9B1, SMPD3, SNAP91, SPAG16, SPATA7, SPINDOC, SPINK5, SPNS3, SRGAP3, SSMEM1, SSX2/SSX2B, STARD8, STK19, STOML3, SYNE1, SYT17, SYT5, TBC1D8, TDRD9, TEC, TECTA, TENT5D, TEPP, TEX47, TFF3, TKTL2, TLCD3B, TLE1, TMEM108, TMEM132B, TMEM270, TMEM273, TMEM30B, TNS4, TRMT9B, TRPC6, VLDLR, VPS13A, VSTM1, WDR87, ZNF391, ZNF462, ZSCAN5A
Hematological System Development and Function, Inflammatory Response, Tissue Morphology	Quantity of plasmacytoid dendritic cells	3.81E-03		0.218	4	CSF1, GPR183, ID2, IDO1
Cancer, Dermatological Diseases and Conditions, Organismal Injury and Abnormalities	Cutaneous melanoma	3.84E-03			207	ABCB5, ACSM3, ACSM5, ADARB1, ADGRE1, ADGRL3, ALDH7A1, ALOX15, ANO5, APOB, ARHGAP29, ARNT2, ARVCF, ASL, ATP6V0A2, B3GNT4, B4GALNT2, BATF2, BSND, C11orf88, C1QL2, CACNA1E, CAPSL, CASC1, CCR3, CD101, CD2, CD38, CD96, CENPE, CFHR1, CLC, CLINT1, COL7A1, CORO2A, CSF1, CT45A10/CT45A5, CT62, CTLA4, CXCL9, CYP2C8, DEFB119, DHX36, DYNAP, EDDM3A, EEF2K, EFCAB5, EPN2, FAM227B, FAM47A, FFAR4, FOXO3, FRMD7, FSCB, GASK1A, GCNT1, GHRHR, GLDC, GLRA1, GLYATL2, GPER1, GPR34, HABP2, HOXB8, HRH4, HS3ST6, HTR3A, HTR3C, IDO1, IGLL1/IGLL5, IKZF2, IL1RL1, IL2RA, IL5RA, IL9, ITM2C, KCNH2, KIAA1217, KLF12, KLF17, KLRC3, KRT33B, KRT36, LDB2, LGR5, LHFPL6, LILRA6, LIMCH1, LRRC17, LUM, MACC1, MAGEA11, MAGEC2, MEDAG, MEOX2, MERTK, MET, MKRN3, MON2, MS4A6E, MSH2, MUC1, NEK2, NFIB, NKAIN3, NKD2, NOVA1, NPHS2, NPY4R/NPY4R2, NRAP, OR10A6, OR10G8, OR10R2, OR12D3, OR2A14, OR2D2, OR2V2, OR2Y1, OR4K5, OR5F1, OR5H2, OR9Q1, P2RY10, P2RY14, PAPOLB, PARP6, PGAM2, PKLR, PNPLA7, PPP1R12B, PPP4R1, PRICKLE1, PRKG1, PRLR, PTF1A, PTGDR2, PTPN5, PWWP3B, PXDNL, RAPGEF5, RAPH1, RASIP1, RBAK, REM2, RFPL4B, RGS1, RNF112, RXFP2, SEL1L3, SERPINA6, SFMBT2, SIGLEC8, SIGLECL1, SIPA1L2, SLC15A5, SLC17A1, SLC17A3, SLC25A2, SLC25A38, SLC29A1, SLC36A3, SLC46A1, SLC4A7, SLC7A8, SLC8A3, SLC9B1, SMPD3, SNAP91, SPAG16, SPATA7, SPINDOC, SPINK5, SPNS3, SRGAP3, SSMEM1, STARD8, STK19, STOML3, SYNE1, SYT17, SYT5, TBC1D8, TDRD9, TEC, TECTA, TENT5D, TEPP, TEX47, TFF3, TKTL2, TLCD3B, TLE1, TMEM108, TMEM132B, TMEM270, TMEM273, TMEM30B, TNS4, TRMT9B, TRPC6, VLDLR, VPS13A, VSTM1, WDR87, ZNF391, ZNF462, ZSCAN5A
Cancer, Organismal Injury and Abnormalities	Metastatic RET mutation positive malignant solid tumor	4.16E-03			3	CTLA4, MERTK, MET
Cellular Function and Maintenance, Cellular Growth and Proliferation, Hematological System Development and Function	Production of phagocytes	4.16E-03			3	CSF1, ID2, IL9
Developmental Disorder, Hematological Disease, Immunological Disease	Autoimmune lymphoproliferative syndrome	4.16E-03			3	CTLA4, FOXP3, IL2RA
Cellular Movement, Hematological System Development and Function, Immune Cell Trafficking	Cell movement of granulocytes	4.16E-03		1.941	20	CCL23, CCL28, CCR3, CD2, CFHR1, CRH, CSF1, CXCL9, CYP2C8, GCNT1, IL1RL1, IL2RA, IL9, LUM, MET, PRKG1, PRTN3, PTGDR2, SIGLEC8, TRPC6
Molecular Transport	Quantity of metal	4.25E-03		1.725	24	CCR3, CD2, CD38, CRH, CSF1, CXCL9, CYSLTR2, FFAR4, FOXO3, GPER1, HFE, HRH4, KCNH2, KLHL3, mir-183, PMCH, PMP22, PRKG1, PRLR, PTGDR2, RGS1, TEC, TNNC1, TRPC6
Cellular Movement, Hematological	Chemotaxis of eosinophils	4.26E-03		0.555	4	CCL28, CCR3, CXCL9, PTGDR2

System Development and Function, Hypersensitivity Response, Immune Cell Trafficking, Inflammatory Response						
Cell-mediated Immune Response, Cellular Movement, Hematological System Development and Function, Immune Cell Trafficking, Lymphoid Tissue Structure and Development	Homing of T lymphocytes	4.37E-03		0.051	8	CCL23, CCL27, CCL28, CCR3, CXCL9, FOXP3, PTGDR2, RGS1
Cell-mediated Immune Response, Cellular Movement, Hematological System Development and Function, Immune Cell Trafficking, Inflammatory Response, Lymphoid Tissue Structure and Development	Chemotaxis of T lymphocytes	4.91E-03		0.179	7	CCL23, CCL27, CCL28, CCR3, CXCL9, PTGDR2, RGS1
Hematological System Development and Function, Hypersensitivity Response, Tissue Morphology	Quantity of eosinophils	5.23E-03		0.144	8	CCL28, CCR3, FOXP3, IL1RL1, IL5RA, IL9, PTGDR2, SIGLEC8
Endocrine System Development and Function, Nervous System Development and Function, Organ Morphology, Tissue Morphology	Quantity of corticotroph cells	5.47E-03			2	CRH, SMPD3
Cell Cycle	Termination of cell cycle progression	5.47E-03			2	CDKN2C, ID2
Post-Translational Modification	Association of protein	5.47E-03			2	CD38, PLIN2

Hematological System Development and Function, Lymphoid Tissue Structure and Development, Organ Morphology, Tissue Morphology	Abnormal size of lymphoid organ	5.47E-03			2	CDKN2C, IL2RA
Gastrointestinal Disease	Radiation induced nausea and vomiting	5.47E-03			2	HTR3A, HTR3C
Cell Morphology, Connective Tissue Development and Function, Tissue Morphology	Abnormal morphology of spiral ligament fibrocyte	5.47E-03			2	SLC4A7, SLC7A8
Cell Morphology	Polarization of monocytes	5.47E-03			2	CSF1, NAMPT
Cardiovascular System Development and Function, Cell Cycle	Arrest in cell cycle progression of endothelial cells	5.47E-03			2	FOXO3, REM2
Free Radical Scavenging	Scavenging of reactive oxygen species	5.47E-03			2	CAT, FOXO3
Lipid Metabolism, Molecular Transport, Small Molecule Biochemistry	Quantity of lipoxin A4	5.47E-03			2	ALOX15, MERTK
Immunological Disease, Neurological Disease	Experimental autoimmune neuritis	5.47E-03			2	ALOX15, PMP22
Cancer, Developmental Disorder, Endocrine System Disorders, Organismal Injury and Abnormalities, Reproductive System Disease	Thecoma	5.47E-03			2	FOXL2, GATA6
Cell Morphology, Cellular Assembly and Organization	Polarization of membrane rafts	5.47E-03			2	CD2, MAL
Nervous System Development and Function	Response of chorda tympani	5.47E-03			2	FFAR4, HTR3A
Dermatological Diseases and Conditions,	Skin lesion	5.53E-03		-0.323	218	ABCB5, ACSM3, ACSM5, ADARB1, ADGRE1, ADGRL3, ALDH7A1, ALOX15, ANO5, APOB, ARHGAP29, ARNT2, ARVCF, ASL, ATOH8, ATP6V0A2, B3GNT4, B4GALNT2, BATF2, BSND, C11orf88, C1QL2, CACNA1E, CAPSL, CASC1, CCL27, CCR3, CD101, CD2, CD38, CD96, CENPE, CFHR1, CHST6, CLC, CLINT1, COL7A1, CORO2A, CSF1, CT45A10/CT45A5, CT62, CTLA4, CXCL9, CYP2C8, CYSLTR2, DEFB119, DHX36, DLX3, DYNAP, DZIP3, EDDM3A, EEF2K, EFCAB5, EPN2, FAM227B, FAM47A, FFAR4, FOXO3, FOXP3, FRMD7, FSCB, GAPT,

Organismal Injury and Abnormalities						GASK1A, GCNT1, GHRHR, GLDC, GLRA1, GLYATL2, GPER1, GPR34, HABP2, HOXB8, HRH4, HS3ST6, HTR3A, HTR3C, IDO1, IGF2BP3, IGLL1/IGLL5, IKZF2, IL1RL1, IL2RA, IL5RA, IL9, ITM2C, KCNH2, KIAA1217, KLF12, KLF17, KLRC1, KLRC3, KRT33B, KRT36, LDB2, LGR5, LHFPL6, LILRA6, LIMCH1, LRRC17, LUM, MACC1, MAGEA11, MAGEC2, MEDAG, MEOX2, MERTK, MET, MKRN3, MON2, MS4A6E, MSH2, MUC1, NEK2, NFIB, NKAIN3, NKD2, NOVA1, NPHS2, NPY4R/NPY4R2, NRAP, OR10A6, OR10G8, OR10R2, OR12D3, OR2A14, OR2D2, OR2V2, OR2Y1, OR4K5, OR5F1, OR5H2, OR9Q1, P2RY10, P2RY14, PAPOLB, PARP6, PGAM2, PKLR, PNPLA7, PPP1R12B, PPP4R1, PRICKLE1, PRKG1, PRLR, PTF1A, PTGDR2, PTPN5, PWWP3B, PXDNL, RAPGEF5, RAPH1, RASIP1, RBAK, REM2, RFPL4B, RGS1, RNF112, RXFP2, SEL1L3, SERPINA6, SFMBT2, SIGLEC8, SIGLECL1, SIPA1L2, SLC15A5, SLC17A1, SLC17A3, SLC25A2, SLC25A38, SLC29A1, SLC36A3, SLC46A1, SLC4A7, SLC7A8, SLC8A3, SLC9B1, SMPD3, SNAP91, SPAG16, SPATA7, SPINDOC, SPINK5, SPNS3, SRGAP3, SSMEM1, SSX2/SSX2B, STARD8, STK19, STOML3, SYNE1, SYT17, SYT5, TBC1D8, TDRD9, TEC, TECTA, TENT5D, TEPP, TEX47, TFF3, TKTL2, TLCD3B, TLE1, TMEM108, TMEM132B, TMEM270, TMEM273, TMEM30B, TNS4, TRMT9B, TRPC6, VLDLR, VPS13A, VSTM1, WDR87, ZNF391, ZNF462, ZSCAN5A
Embryonic Development, Organismal Development, Tissue Development	Specification of embryonic tissue	5.76E-03			3	GATA6, MEOX2, PTF1A
Cellular Development, Nervous System Development and Function	Commitment of neurons	5.79E-03			4	GSX1, ID2, OLIG2, PTF1A
Cellular Development, Cellular Growth and Proliferation, Hematological System Development and Function, Lymphoid Tissue Structure and Development	Proliferation of effector T lymphocytes	5.79E-03		-1.091	4	CTLA4, FOXO3, IDO1, IL2RA
Connective Tissue Development and Function, Skeletal and Muscular System Development and Function	Bone mineral density of femur	6.37E-03			4	CD38, CSF1, GHRHR, PRLR
Cell-To-Cell Signaling and Interaction, Cellular Growth and Proliferation, Hematological System Development and Function	Induction of T lymphocytes	6.40E-03			5	CTLA4, KLRC1, MERTK, MUC1, PRKG1
Cell-To-Cell Signaling and Interaction, Cellular Compromise	Oxidative stress response of cells	6.40E-03		0.937	5	CAT, HFE, MET, MUC1, NAMPT

Cellular Function and Maintenance, Molecular Transport	Flux of ion	6.44E-03		1.482	17	CCL23, CCR3, CD101, CD2, CD38, CLIC5, CRH, CTLA4, GPR183, HTR3A, HTR3C, IL1RL1, PMCH, PRKG1, SLC8A3, TEC, TRPC6
Cancer, Organismal Injury and Abnormalities, Renal and Urological Disease	Advanced sarcomatoid renal cell carcinoma	6.67E-03			3	CTLA4, MERTK, MET
Developmental Disorder, Hereditary Disorder, Organismal Injury and Abnormalities, Renal and Urological Disease	Autosomal dominant polycystic kidney disease	6.93E-03			7	ADGRL3, CD2, MEIS2, MUC1, PPP1R12B, PRKG1, VLDLR
Cancer, Organismal Injury and Abnormalities, Respiratory Disease	Pulmonary adenoma	6.99E-03		-0.283	4	CASC1, CDKN2C, FOXO3, MSH2
Cell-mediated Immune Response, Cellular Development, Cellular Function and Maintenance, Cellular Growth and Proliferation, Embryonic Development, Hematological System Development and Function, Hematopoiesis, Lymphoid Tissue Structure and Development, Organ Development, Organismal Development, Tissue Development	Differentiation of induced regulatory T-lymphocyte	6.99E-03		1.131	4	FOXO3, FOXP3, HCAR1, MERTK
Cellular Function and Maintenance, Hematological System Development and Function	Regulation of mononuclear leukocytes	6.99E-03		1.982	4	CSF1, CTLA4, FOXP3, IL2RA
Hereditary Disorder, Organismal Injury and Abnormalities, Renal and Urological Disease	Autosomal dominant kidney disease	7.04E-03			8	ADGRL3, CD2, KLHL3, MEIS2, MUC1, PPP1R12B, PRKG1, VLDLR

Cancer, Hematological Disease, Immunological Disease, Organismal Injury and Abnormalities	Mature T-cell neoplasm	7.13E-03			23	CCR3, CD2, CD24, CDKN2C, CXCL9, EPC1, FOXO3, GPER1, GPR183, ID2, IDO1, IKZF2, IL2RA, ITM2C, KLF17, MAL, MERTK, MET, mir-28, mir-876, MUC1, NFIB, PRKG1
Humoral Immune Response, Protein Synthesis	Production of antibody	7.18E-03		0.839	15	CCL28, CTLA4, FOXP3, GAPT, HRH4, IGLL1/IGLL5, IL1RL1, IL2RA, IL5RA, IL9, MERTK, MSH2, PTGDR2, RGS1, TEC
Hematological System Development and Function, Immunological Disease, Lymphoid Tissue Structure and Development, Organ Morphology, Organismal Injury and Abnormalities, Tissue Morphology	Abnormal morphology of lymph node	7.29E-03			9	ALOX15, CDKN2C, CTLA4, FOXP3, GCNT1, ID2, IL2RA, IL5RA, MERTK
Molecular Transport, Nucleic Acid Metabolism, Small Molecule Biochemistry	Transport of uric acid	7.56E-03			2	SLC17A1, SLC17A3
Developmental Disorder, Organismal Survival	Sudden infant death syndrome	7.56E-03			2	KCNH2, SCN4B
Cell Death and Survival	Apoptosis of retinoblastoma cell lines	7.56E-03			2	HOTAIR, MET
Digestive System Development and Function, Gastrointestinal Disease, Hepatic System Development and Function, Hepatic System Disease, Infectious Diseases, Inflammatory Disease, Inflammatory Response, Organ Development, Organismal Injury and Abnormalities	Acute hepatitis C	7.56E-03			2	CSF1, CTLA4
Embryonic Development,	Development of eyelid	7.56E-03			2	FOXL2, IKZF2

Organismal Development						
Connective Tissue Development and Function, Tissue Morphology	Mass of perirenal white adipose tissue	7.56E-03			2	GPER1, PMCH
Neurological Disease, Organismal Injury and Abnormalities	Hypothalamus dysfunction	7.56E-03			2	ARNT2, SERPINA6
Cell-To-Cell Signaling and Interaction, Hematological System Development and Function, Inflammatory Response	Antibody response of lymphocytes	7.56E-03			2	IL2RA, MERTK
Embryonic Development, Nervous System Development and Function, Ophthalmic Disease, Organ Development, Organ Morphology, Organismal Development, Organismal Injury and Abnormalities, Tissue Development, Visual System Development and Function	Abnormal morphology of fundus of eye	7.56E-03			2	SLC4A7, VLDLR
Cellular Movement	Movement of osteoclast precursor cells	7.56E-03			2	CSF1, GPR183
Nucleic Acid Metabolism, Small Molecule Biochemistry	Biosynthesis of NAD+	7.56E-03			2	HAAO, NAMPT
Connective Tissue Disorders, Hematological Disease, Immunological Disease, Organismal Injury and Abnormalities	Eosinophilia of blood	7.56E-03			2	IL1RL1, SIGLEC8

Cancer, Endocrine System Disorders, Hereditary Disorder, Organismal Injury and Abnormalities	Familial thyroid gland non-medullary carcinoma	7.56E-03			2	HABP2, MSH2
Connective Tissue Disorders, Inflammatory Disease, Inflammatory Response, Organismal Injury and Abnormalities, Skeletal and Muscular Disorders	Antibody-induced arthritis	7.56E-03			2	FOXP3, IL1RL1
Cellular Development, Cellular Growth and Proliferation, Hematological System Development and Function, Humoral Immune Response, Lymphoid Tissue Structure and Development	Proliferation of B-1 lymphocytes	7.67E-03			3	CDKN2C, GPR183, IGLL1/IGLL5
Hair and Skin Development and Function	Tensile strength of skin	7.67E-03			3	LUM, P3H4, SPINK5
Gastrointestinal Disease, Organismal Injury and Abnormalities	Gastroparesis	7.67E-03			3	HTR3A, HTR3C, KCNH2
Cellular Function and Maintenance, Molecular Transport	Flux of inorganic cation	7.72E-03		1.261	16	CCL23, CCR3, CD101, CD2, CD38, CRH, CTLA4, GPR183, HTR3A, HTR3C, IL1RL1, PMCH, PRKG1, SLC8A3, TEC, TRPC6
Cancer, Organismal Injury and Abnormalities	Melanoma	7.72E-03			236	ABCB5, ACR, ACSM3, ACSM5, ADARB1, ADGRE1, ADGRL3, ALDH7A1, ALOX15, ANO5, APOB, APOBEC4, ARHGAP29, ARNT2, ARVCF, ASL, ATP6V0A2, ATP6V1A, ATXN7L2, B3GNT4, B4GALNT2, BATF2, BSND, C11orf88, C1QL2, CACNA1E, CAPSL, CASC1, CCR3, CD101, CD2, CD38, CD96, CENPE, CFHR1, CLC, CLINT1, CMBL, COL7A1, CORO2A, CSF1, CT45A10/CT45A5, CT62, CTLA4, CXCL9, CYP2C8, CYSLTR2, DEFB119, DHX36, DYNAP, EDDM3A, EEF2K, EFCAB5, EPC1, EPN2, ERMP1, FAM227B, FAM47A, FFAR4, FKBPL, FOXO3, FRMD7, FRRS1, FSCB, GASK1A, GCNT1, GHRHR, GLDC, GLRA1, GLYATL2, GPER1, GPR34, GSG1, HABP2, HOXB8, HRCT1, HRH4, HS3ST6, HTR3A, HTR3C, IDO1, IGLL1/IGLL5, IKZF2, IL1RL1, IL2RA, IL5RA, IL9, ITM2C, KCNG3, KCNH2, KIAA1217, KLF12, KLF17, KLHL3, KLRC1, KLRC3, KRT33B, KRT36, LDB2, LGR5, LHFPL6, LILRA6, LIMCH1, LRFN4, LRRC17, LUM, MACC1, MAGEA11, MAGEC2, MEDAG, MEOX2, MERTK, MET, mir-183, mir-28, MKRN3, MON2, MS4A5, MS4A6E, MSH2, MUC1, NEK2, NFIB, NKAIN3, NKD2, NOVA1, NPHS2, NPY4R/NPY4R2, NRAP, OCIAD2, OR10A6, OR10G8, OR10R2, OR12D3, OR2A14, OR2D2, OR2V2, OR2Y1, OR4K5, OR5F1, OR5H2, OR9Q1, P2RY10, P2RY14, PAPOLB, PARP6, PGAM2, PKLR, PNPLA7, PPP1R12B, PPP4R1, PRICKLE1, PRKG1, PRLR, PTF1A, PTGDR2, PTPN5, PWWP3B, PXDNL, RAPGEF5, RAPH1, RASIP1, RBAK, REG4, REM2, RFPL4B, RGS1, RNF112, RXFP2, SEL1L3, SERPINA6, SFMBT2, SIGLEC8, SIGLECL1, SIPA1L2, SLC10A5, SLC15A5, SLC17A1, SLC17A3, SLC25A2, SLC25A38, SLC29A1, SLC36A3, SLC46A1, SLC4A7, SLC7A8, SLC8A3, SLC9B1, SMPD3, SNAP91, SPAG16, SPATA31A6 (includes others), SPATA7, SPINDOC, SPINK5, SPNS3, SRGAP3, SSMEM1, STARD8, STK19, STOML3, SYNE1, SYT17, SYT5, TBC1D8, TDRD9, TEC, TECTA, TENT5D, TEPP, TEX11, TEX47, TFF3, TKTL2, TLCD3B, TLE1, TMEM108, TMEM132B, TMEM17, TMEM270, TMEM273, TMEM30B, TNNC1, TNS4, TRMT9B, TRPC6, VLDLR, VPS13A, VSTM1, WDR87, XKR7, ZNF154, ZNF391, ZNF462, ZNF551, ZSCAN5A

Cell-To-Cell Signaling and Interaction, Cellular Growth and Proliferation	Stimulation of lymphatic system cells	7.83E-03		0.555	9	CD2, CD24, CRH, CSF1, CTLA4, KLRC1, MERTK, MUC1, PRKG1
Organ Morphology	Abnormal morphology of gland	8.06E-03			16	ARNT2, CD38, CDKN2C, CRH, ELOVL3, FFAR4, FOXP3, GATA6, GSX1, IL2RA, IL9, NFIB, PRLR, PTF1A, SMPD3, TFF3
Connective Tissue Development and Function, Tissue Development	Maturation of connective tissue	8.15E-03		1.387	6	ALOX15, CSF1, FOXO3, IL9, RNF112, SMAD5
Inflammatory Response	Inflammatory response	8.29E-03	Increased	2.527	35	ALOX15, CCL23, CCL27, CCL28, CCR3, CD38, CD96, CRH, CSF1, CXCL9, FFAR4, FOXO3, FOXP3, GCNT1, GPER1, GPR183, HRH4, IDO1, IL1RL1, IL2RA, IL9, LUM, mir-147, MOK, MSH2, MUC1, PLIN2, PMP22, PRKG1, PRTN3, PTGDR2, RAPH1, RGS1, RNASE2, TRPC6
Cell Death and Survival	Cell survival of tumor cell lines	8.34E-03		-0.059	7	CAT, EEF2K, FOXO3, FOXP3, HOTAIR, MET, MSH2
Endocrine System Development and Function, Molecular Transport, Protein Synthesis, Small Molecule Biochemistry	Quantity of IGF1 in blood	8.35E-03		1	4	FOXL2, GPER1, SMPD3, SPR
Cell Signaling, Cellular Function and Maintenance, Vitamin and Mineral Metabolism	Elevation of Ca2+ in cytosol	8.60E-03			8	CCL28, CD24, CD38, FFAR4, GPER1, PMCH, PRKG1, TRPC6
Cell Morphology, Immunological Disease	Abnormal morphology of leukocytes	8.65E-03			12	CCL28, CDKN2C, CSF1, CTLA4, FOXO3, FOXP3, GCNT1, ID2, IGLL1/IGLL5, IL2RA, IL5RA, MERTK
Immunological Disease	Abnormal quantity of cytokine	8.72E-03			7	FOXP3, HRH4, IDO1, IL9, PTGDR2, RAPH1, SERPINA6
Cellular Function and Maintenance	Regulation of cells	8.72E-03	Increased	2.207	7	CRH, CSF1, CTLA4, FOXP3, IL2RA, IL9, SRI
Cellular Movement, Renal and Urological System Development and Function	Scattering of kidney cell lines	8.76E-03			3	CSF1, MET, TFF3
Cancer, Organismal Injury and Abnormalities	Resectable secondary tumor	9.09E-03			4	CTLA4, IL2RA, MERTK, MET
Cell Death and Survival, Connective Tissue Disorders, Hematological Disease, Organismal Injury and Abnormalities	Hemolysis	9.29E-03		-1	8	CAT, CFHR1, CTLA4, FOXO3, IL2RA, PKLR, PRTN3, SLC29A1

Skeletal and Muscular System Development and Function	Contraction of striated muscle	9.29E-03			8	KCNH2, MET, PGAM2, SCN4B, SLC8A3, SMAD5, SRI, TNNC1
Cancer, Hematological Disease, Immunological Disease, Organismal Injury and Abnormalities	Hyperplasia of leukocytes	9.53E-03		0.277	5	CDKN2C, FOXO3, IL2RA, IL9, MERTK
Humoral Immune Response, Protein Synthesis	Quantity of immunoglobulin	9.64E-03		1.061	14	CCL28, FOXP3, GAPT, HRH4, IGLL1/IGLL5, IL1RL1, IL2RA, IL5RA, IL9, MERTK, MSH2, PTGDR2, RGS1, TEC
Endocrine System Development and Function, Endocrine System Disorders, Organ Morphology, Organismal Development, Organismal Injury and Abnormalities, Reproductive System Disease	Abnormal morphology of pituitary gland	9.87E-03			4	ARNT2, CDKN2C, GSX1, SMPD3
Cellular Movement, Hematological System Development and Function, Hematopoiesis	Chemotaxis of hematopoietic progenitor cells	9.93E-03			3	CCR3, CXCL9, RGS1
Digestive System Development and Function, Organ Morphology, Tissue Morphology	Quantity of Paneth cells	9.95E-03			2	CSF1, GATA6
Cancer, Organismal Injury and Abnormalities, Tumor Morphology	Transformation of cancer cells	9.95E-03			2	MET, TFF3
Molecular Transport	Secretion of Vldl-Triglyceride	9.95E-03			2	APOB, PLIN2
Cancer, Organismal Injury and Abnormalities	Recurrent CD274 negative head and neck squamous cell carcinoma	9.95E-03			2	CTLA4, IDO1
Embryonic Development, Organismal Development, Tissue Morphology	Abnormal size of somites	9.95E-03			2	EPN2, MEOX2

Cardiovascular System Development and Function, Cellular Development, Embryonic Development, Organismal Development, Tissue Development	Differentiation of heart precursor cells	9.95E-03			2	DHX36, GATA6
Cellular Function and Maintenance	Homeostasis of dendritic cells	9.95E-03			2	GPR183, ID2
Cellular Development, Respiratory System Development and Function, Tissue Development	Differentiation of type II pneumocytes	9.95E-03			2	GATA6, NFIB
Gastrointestinal Disease, Organismal Injury and Abnormalities	Irritable bowel syndrome with diarrhea	9.95E-03			2	HTR3A, HTR3C
Cellular Development, Cellular Growth and Proliferation	Proliferation of retinoblastoma cell lines	9.95E-03			2	HOTAIR, MET
Cell-To-Cell Signaling and Interaction, Cellular Growth and Proliferation, Hematological System Development and Function	Induction of cytotoxic T cells	9.95E-03			2	CTLA4, MUC1
Cellular Development, Cellular Growth and Proliferation, Hematological System Development and Function, Lymphoid Tissue Structure and Development	Expansion of effector T lymphocytes	9.95E-03			2	FOXO3, IL2RA
Cellular Function and Maintenance, Cellular Growth and Proliferation, Hematological System Development and Function	Production of antigen presenting cells	9.95E-03			2	CSF1, ID2

Organ Morphology, Reproductive System Development and Function	Morphology of gonad	1.01E-02			22	ACR, CDKN2C, CGB3 (includes others), DDHD1, EEF2K, FOXL2, FOXO3, GATA6, GATM, GPER1, IDO1, MERTK, MSH5, PAPOLB, PLIN2, RXFP2, SMAD5, SMPD3, SPAG16, TDRD9, VLDLR, VPS13A
Cell-To-Cell Signaling and Interaction, Cellular Movement, Hematological System Development and Function, Immune Cell Trafficking	Recruitment of leukocytes	1.06E-02	Increased	2.718	16	ALOX15, APOB, CAT, CCL23, CCL27, CCL28, CCR3, CSF1, CXCL9, GCNT1, IDO1, IL2RA, IL9, PRTN3, PTGDR2, TRPC6
Cancer, Organismal Injury and Abnormalities	Metastatic solid tumor	1.06E-02		0.239	29	CAT, CD24, CDKN2C, CFHR1, CSF1, CTLA4, CXCL9, DLX3, GATA6, HOTAIR, ID2, IDO1, IKZF2, IL1RL1, IL2RA, KLHL3, LRRC17, MERTK, MET, mir-183, mir-28, mir-31, mir-450, MSH2, MUC1, NKD2, PRLR, SEL1L3, TNNC1
Cell Death and Survival, Cellular Compromise	Cytotoxicity of lymphocytes	1.07E-02		-0.113	9	CCR3, CD2, CD38, CD96, CRH, CTLA4, FOXP3, IL9, KLRC1
Embryonic Development, Nervous System Development and Function, Neurological Disease, Ophthalmic Disease, Organ Development, Organ Morphology, Organismal Development, Organismal Injury and Abnormalities, Tissue Development, Tissue Morphology, Visual System Development and Function	Abnormal morphology of retinal pigment epithelium	1.07E-02			4	ABCB5, APOB, MERTK, VLDLR
Cell Cycle	Arrest in interphase of epithelial cell lines	1.07E-02			4	ALOX15, DLX3, MET, MSH5
Hematological Disease, Immunological Disease	Hypereosinophilia	1.07E-02			4	IL1RL1, IL2RA, IL5RA, RNASE2
Cellular Movement, Hematological System Development and Function, Hypersensitivity	Cellular infiltration by eosinophils	1.08E-02		1.091	5	CD2, IL1RL1, IL9, PTGDR2, SIGLEC8

Response, Immune Cell Trafficking						
Cell Signaling, Cellular Function and Maintenance, Molecular Transport, Vitamin and Mineral Metabolism	Flux of Ca2+	1.08E-02		0.985	15	CCL23, CCR3, CD101, CD2, CD38, CRH, CTLA4, GPR183, HTR3A, HTR3C, IL1RL1, PRKG1, SLC8A3, TEC, TRPC6
Cell-To-Cell Signaling and Interaction, Cellular Movement	Recruitment of myeloid cells	1.08E-02	Increased	2.465	14	ALOX15, APOB, CAT, CCL23, CCR3, CSF1, CXCL9, GCNT1, IDO1, IL2RA, IL9, PRTN3, PTGDR2, TRPC6
Cellular Function and Maintenance	Cellular homeostasis	1.09E-02	Increased	2.335	64	ABCB5, ALOX15, AQP5, ATP6V0A2, ATP6V1A, BACE2, BNIP3, BSND, CACNA1E, CAT, CCL23, CCL28, CCR3, CD101, CD2, CD24, CD38, CLIC5, CRH, CTLA4, EEF2K, FFAR4, FOXO3, FOXP3, GAPT, GATM, GHRHR, GLRA1, GPER1, GPR183, HAAO, HCAR1, HCN3, HFE, HTR3A, HTR3C, ID2, IDO1, IKZF2, IL1RL1, IL2RA, IL5RA, IL9, KCNH2, KLHL3, MERTK, MET, mir-515, MON1A, MSH2, MUC1, NAMPT, PIK3R6, PMCH, PMP22, PRKG1, RASIP1, SLC46A1, SLC4A7, SLC8A3, SPINK5, TEC, TRPC6, VPS13A
Neurological Disease	Sporadic motor neuron disease	1.11E-02			6	mir-183, mir-28, mir-31, mir-3180, mir-515, PTPN5
Cell-To-Cell Signaling and Interaction	Response of epithelial cells	1.12E-02			3	IL9, MERTK, MET
Cellular Movement, Hematological System Development and Function, Immune Cell Trafficking, Lymphoid Tissue Structure and Development	Homing of lymphocytes	1.14E-02		0.471	9	CCL23, CCL27, CCL28, CCR3, CXCL9, FOXP3, GCNT1, PTGDR2, RGS1
Cellular Function and Maintenance, Molecular Transport, Small Molecule Biochemistry	Homeostasis of iron ion	1.14E-02			5	ATP6V0A2, ATP6V1A, HFE, MON1A, SLC46A1
Organismal Injury and Abnormalities, Tissue Morphology	Abnormal morphology of epithelial tissue	1.16E-02			20	ABCB5, APOB, BACE2, CDKN2C, CRH, FOXP3, GATA5, GATA6, GPR34, IL9, MERTK, NPHS2, PRLR, RASIP1, SLC4A7, SLC7A8, SMAD5, SPAG16, TFF3, VLDLR
Hematological System Development and Function, Immunological Disease, Lymphoid Tissue Structure and Development, Organ Morphology, Organismal Injury and Abnormalities, Tissue Morphology	Abnormal morphology of enlarged lymph node	1.16E-02			6	CDKN2C, CTLA4, FOXP3, IL2RA, IL5RA, MERTK

Organ Morphology, Reproductive System Development and Function	Morphology of genital organ	1.24E-02			24	ACR, CDKN2C, CGB3 (includes others), DDHD1, EEF2K, FOXL2, FOXO3, GATA5, GATA6, GATM, GPER1, IDO1, MERTK, MSH5, PAPOLB, PLIN2, PRLR, RXFP2, SMAD5, SMPD3, SPAG16, TDRD9, VLDLR, VPS13A
Cancer, Gastrointestinal Disease, Organismal Injury and Abnormalities, Tissue Morphology, Tumor Morphology	Size of digestive organ tumor	1.25E-02			3	HABP2, IDO1, MET
Dermatological Diseases and Conditions, Organ Morphology, Organismal Injury and Abnormalities	Abnormal morphology of granular layer of epidermis	1.25E-02			3	CRH, ELOVL3, SPINK5
Hematological System Development and Function, Immune Cell Trafficking, Inflammatory Response, Tissue Development	Accumulation of regulatory T lymphocytes	1.25E-02			3	CTLA4, IL1RL1, IL2RA
Cell Cycle, Skeletal and Muscular System Development and Function	S phase of smooth muscle cells	1.26E-02			2	GATA6, ID2
Cell-To-Cell Signaling and Interaction, Cellular Growth and Proliferation, Connective Tissue Development and Function	Stimulation of osteoclast precursor cells	1.26E-02			2	CSF1, IL9
Cancer, Organismal Injury and Abnormalities, Respiratory Disease	CD274 positive laryngeal squamous cell carcinoma	1.26E-02			2	CTLA4, IDO1
Cancer, Gastrointestinal Disease, Organismal Injury and Abnormalities	CD274 positive oral squamous cell carcinoma	1.26E-02			2	CTLA4, IDO1
Drug Metabolism, Endocrine System Development and Function, Lipid Metabolism,	Concentration of hydrocortisone	1.26E-02			2	CRH, PMCH

Molecular Transport, Small Molecule Biochemistry						
Cancer, Gastrointestinal Disease, Organismal Injury and Abnormalities, Respiratory Disease	CD274 positive hypopharyngeal squamous cell carcinoma	1.26E-02			2	CTLA4, IDO1
Inflammatory Response	Memory T cell response	1.26E-02			2	CTLA4, FOXO3
Cancer, Organismal Injury and Abnormalities, Renal and Urological Disease	Transitional cell carcinoma of the renal pelvis	1.26E-02			2	CTLA4, IDO1
Cancer, Gastrointestinal Disease, Organismal Injury and Abnormalities, Respiratory Disease	CD274 positive oropharyngeal squamous cell carcinoma	1.26E-02			2	CTLA4, IDO1
Cellular Development, Cellular Growth and Proliferation, Hematological System Development and Function, Hematopoiesis, Lymphoid Tissue Structure and Development, Tissue Development	Differentiation of conventional dendritic cells	1.26E-02			2	CSF1, IL2RA
Cancer, Organismal Injury and Abnormalities	Recurrent CD274 positive head and neck squamous cell carcinoma	1.26E-02			2	CTLA4, IDO1
Cellular Movement, Hematological System Development and Function, Hematopoiesis, Humoral Immune Response, Immune Cell Trafficking, Inflammatory Response, Lymphoid Tissue	Chemotaxis of pre-B lymphocytes	1.26E-02			2	CCR3, RGS1

Structure and Development						
Cellular Development, Cellular Growth and Proliferation	Proliferation of kidney cancer cell lines	1.34E-02		-0.644	6	FOXO3, GNG4, HOTAIR, MET, NAMPT, SLC29A1
Gastrointestinal Disease, Organismal Injury and Abnormalities	Metaplasia of intestine	1.40E-02			3	CCL28, FOXO3, GPER1
Cell-To-Cell Signaling and Interaction, Hematological System Development and Function, Hematopoiesis	Adhesion of hematopoietic progenitor cells	1.40E-02			3	CD2, CXCL9, RGS1
Cancer, Organismal Injury and Abnormalities	Embryonal tumor	1.41E-02			28	ADARB1, APOBEC4, CACNA1E, CDKN2C, CSF1, CTLA4, CXCL9, EEF2K, FOXO3, FRRS1, GLDC, GPER1, HFE, IL2RA, IL9, KCNH2, KLRC1, LGR5, MAL, MERTK, MET, MSH2, NAMPT, OLIG2, OR4K5, PMP22, RAPGEF5, TRPC6
Cell-To-Cell Signaling and Interaction, Hematological System Development and Function, Immune Cell Trafficking, Inflammatory Response	Activation of granulocytes	1.43E-02		1.154	7	CCL23, CXCL9, IL1RL1, IL5RA, IL9, PRTN3, PTGDR2
Cell Death and Survival, Cellular Function and Maintenance	Colony survival of cells	1.43E-02		0.152	5	CAT, CDKN2B-AS1, FOXO3, MET, MSH2
Cellular Development, Hematological System Development and Function, Hematopoiesis	Maturation of hematopoietic progenitor cells	1.43E-02			5	ALOX15, FOXO3, IGLL1/IGLL5, IL9, RNF112
Cellular Development, Cellular Growth and Proliferation, Hematological System Development and Function, Lymphoid Tissue Structure and Development	Proliferation of activated T lymphocytes	1.43E-02		1.067	5	CD24, CTLA4, FOXP3, IL2RA, IL9

Cancer, Organismal Injury and Abnormalities	Advanced malignant solid tumor	1.44E-02		0.239	32	CAT, CD24, CDKN2C, CFHR1, CSF1, CTLA4, CXCL9, DLX3, GATA6, HOTAIR, ID2, IDO1, IKZF2, IL1RL1, IL2RA, KLHL3, LRRC17, MAGEA11, MERTK, MET, mir-183, mir-28, mir-31, mir-450, MSH2, MTFR2, MUC1, NKD2, PRLR, PWWP3B, SEL1L3, TNNC1
Inflammatory Response	Cytotoxic T lymphocyte response	1.44E-02			4	CTLA4, FOXO3, IL9, MUC1
Cancer, Organismal Injury and Abnormalities	Hyperplasia of blood cells	1.53E-02		0.728	6	CDKN2C, CSF1, FOXO3, IL2RA, IL9, MERTK
Cellular Movement	Migration of cells	1.54E-02		1.516	79	ADARB1, ADGRL3, ALOX15, APOB, AQP5, ATOH8, BARHL2, CAT, CCL23, CCL27, CCL28, CCR3, CD2, CD24, CD38, CDKN2B-AS1, CDKN2C, CFHR1, CGB3 (includes others), COL7A1, CRH, CSF1, CTLA4, CXCL9, CYP2C8, CYSLTR2, DLX3, FKBPL, FOXO3, FOXP3, GATA6, GCNT1, GPER1, GPR183, GPR34, HABP2, HOTAIR, HOXB9, HRH4, ID2, IDO1, IGF2BP3, IGLL1/IGLL5, IL1RL1, IL2RA, IL9, KCNH2, KLF17, LDB2, LIMCH1, LUM, MEOX2, MERTK, MET, mir-183, mir-28, mir-31, mir-515, MUC1, NAMPT, NKD2, NOVA1, PIK3R6, PMCH, PMP22, PRKG1, PRLR, PRSS55, PRTN3, PTGDR2, RAPH1, RGS1, RNASE2, SIGLEC8, SLC7A8, SMAD5, TNS4, TRPC6, VLDLR
Humoral Immune Response, Inflammatory Disease, Inflammatory Response	Experimentally induced inflammation	1.55E-02			3	CD2, CTLA4, IL1RL1
Small Molecule Biochemistry	Metabolism of alkaloid	1.55E-02			3	CYP2C8, SLC17A1, SLC17A3
Cell Cycle	G1/S phase transition of epithelial cells	1.56E-02			2	ID2, MET
Inflammatory Disease, Inflammatory Response, Organismal Injury and Abnormalities, Renal and Urological Disease	Nephrotoxic nephritis	1.56E-02			2	IDO1, IL9
Lipid Metabolism, Small Molecule Biochemistry	Conversion of arachidonic acid	1.56E-02			2	ALOX15, CYP2C8
Cell-To-Cell Signaling and Interaction, Hematological System Development and Function, Immune Cell Trafficking	Aggregation of leukocyte cell lines	1.56E-02			2	CD2, CD38
Cell Morphology, Cellular Function and Maintenance	Autophagy of muscle	1.56E-02			2	BNIP3, FOXO3
Cellular Development, Hematopoiesis, Tissue Development	Differentiation of bone marrow precursor cells	1.56E-02			2	CSF1, LRRC17

Cancer, Connective Tissue Disorders, Organismal Injury and Abnormalities, Skeletal and Muscular Disorders	Ewing sarcoma in vertebrae	1.56E-02			2	CACNA1E, OR4K5
Organismal Injury and Abnormalities, Renal and Urological Disease	Cortical renal glomerulopathies	1.56E-02			2	CDKN2C, NPHS2
Embryonic Development, Nervous System Development and Function, Ophthalmic Disease, Organ Development, Organ Morphology, Organismal Development, Organismal Injury and Abnormalities, Tissue Development, Visual System Development and Function	Thinning of cornea	1.56E-02			2	CHST6, LUM
Embryonic Development, Nervous System Development and Function, Organ Development, Organ Morphology, Organismal Development, Tissue Development, Tissue Morphology, Visual System Development and Function	Thickness of corneal stroma	1.56E-02			2	CHST6, LUM
Cancer, Organismal Injury and Abnormalities	Eradication of tumor	1.56E-02			2	CTLA4, IL2RA
Organismal Development, Organismal Injury and Abnormalities	Abnormal morphology of abdomen	1.56E-02			39	ALOX15, CAT, CCL28, CD38, CDKN2C, CRH, CSF1, CTLA4, EEF2K, FFAR4, FOXL2, FOXO3, FOXP3, GATA5, GATA6, GCNT1, HOXB9, ID2, IDO1, IL1RL1, IL2RA, IL5RA, IL9, MEIS2, MEOX2, MERTK, MET, MSH5, NPHS2, PRKG1, PRLR, PTF1A, RAPH1, RGS1, RXFP2, SESTD1, SMPD3, TEC, TFF3
Cell Death and Survival, Embryonic Development	Cell death of embryonic cells	1.61E-02		1.664	7	BNIP3, GATA6, MET, MSH2, REM2, SMAD5, SYNE1

Cellular Movement, Hematological System Development and Function, Immune Cell Trafficking	Cellular infiltration by granulocytes	1.63E-02		0.684	12	CD2, CRH, CSF1, CYP2C8, IL1RL1, IL2RA, IL9, MET, PRKG1, PRTN3, PTGDR2, SIGLEC8
Cellular Compromise	Depletion of lymphatic system cells	1.66E-02		-1.951	4	CD38, CSF1, CTLA4, IL2RA
Cancer, Organismal Injury and Abnormalities, Renal and Urological Disease	Stage IV renal cancer	1.66E-02			4	CTLA4, IL2RA, MERTK, MET
Cell Death and Survival	Apoptosis of granulocytes	1.67E-02		0.848	6	CAT, FOXO3, IL9, NAMPT, PRTN3, SIGLEC8
Cancer, Hematological Disease, Immunological Disease, Organismal Injury and Abnormalities	T-cell non-Hodgkin lymphoma	1.70E-02			22	CCR3, CD2, CD24, CDKN2C, CXCL9, EPC1, GPR183, ID2, IDO1, IKZF2, IL2RA, IL9, ITM2C, MAL, MERTK, MET, mir-28, mir-876, MSH2, MUC1, NFIB, PRKG1
Hematological System Development and Function	Anergy of lymphocytes	1.71E-02			3	CTLA4, FOXP3, IDO1
Cancer, Cardiovascular Disease, Organismal Injury and Abnormalities	Hemangiosarcoma	1.71E-02			3	CDKN2C, FOXO3, MSH2
Dermatological Diseases and Conditions, Neurological Disease, Organismal Injury and Abnormalities	Mechanical hyperalgesia	1.71E-02			3	HTR3A, IL1RL1, TRPC6
Cancer, Endocrine System Disorders, Organismal Injury and Abnormalities, Reproductive System Disease	Development of pituitary gland tumor	1.71E-02			3	CDKN2C, FOXO3, PRLR
Cell-To-Cell Signaling and Interaction, Cellular Growth and Proliferation, Hematological System	Stimulation of lymphocytes	1.75E-02		0.152	8	CD2, CD24, CRH, CTLA4, KLRC1, MERTK, MUC1, PRKG1

Development and Function						
Cancer, Hematological Disease, Immunological Disease, Organismal Injury and Abnormalities	T-cell malignant neoplasm	1.76E-02			26	CCR3, CD2, CD24, CDKN2C, CXCL9, EPC1, FOXO3, GPER1, GPR183, ID2, IDO1, IKZF2, IL2RA, IL9, ITM2C, KLF17, MAL, MERTK, MET, mir-28, mir-876, MSH2, MUC1, NFIB, OLIG2, PRKG1
Immunological Disease	Abnormal morphology of immune system	1.78E-02			14	CCL28, CDKN2C, CSF1, CTLA4, FOXO3, FOXP3, GCNT1, HOXB8, ID2, IGLL1/IGLL5, IL2RA, IL5RA, IL9, MERTK
Cellular Development	Transdifferentiation	1.86E-02		0.472	5	CGB3 (includes others), CSF1, FOXO3, MUC1, PTF1A
Cancer, Cardiovascular Disease, Organismal Injury and Abnormalities	Development of angiosarcoma	1.88E-02			3	CDKN2C, FOXO3, MSH2
Cellular Movement, Hematological System Development and Function, Immune Cell Trafficking	Movement of B-lymphocyte derived cell lines	1.88E-02			3	CCL27, PTGDR2, RGS1
Carbohydrate Metabolism, Small Molecule Biochemistry	Biosynthesis of keratan sulfate	1.88E-02			3	B3GNT4, CHST6, LUM
Cancer, Organismal Injury and Abnormalities	Delay in growth of tumor	1.88E-02			3	ATP6V0A2, CTLA4, IKZF2
Cellular Assembly and Organization, Cellular Function and Maintenance	Release of exosomes	1.88E-02			2	MAL, SMPD3
Cell Cycle	Mitotic exit of cervical cancer cell lines	1.88E-02			2	NEK2, WEE1
Cell Morphology, Hematological System Development and Function, Inflammatory Response	Polarization of M2 macrophages	1.88E-02			2	CSF1, NAMPT
Cell Death and Survival, Organismal Injury and Abnormalities	Apoptosis of colonocytes	1.88E-02			2	IL2RA, TFF3

Cell Morphology, Cellular Assembly and Organization, Cellular Function and Maintenance	Permeability of plasma membrane	1.88E-02			2	BNIP3, CCL28
Embryonic Development, Endocrine System Development and Function, Nervous System Development and Function, Organ Development, Organismal Development, Tissue Development	Development of hypothalamus	1.88E-02			2	ARNT2, GSX1
Connective Tissue Development and Function, Skeletal and Muscular System Development and Function	Bone mineral density of tibia	1.88E-02			2	CD38, PRLR
Nucleic Acid Metabolism, Small Molecule Biochemistry	Metabolism of uric acid	1.88E-02			2	SLC17A1, SLC17A3
Hematological System Development and Function, Immune Cell Trafficking, Inflammatory Response, Tissue Development	Accumulation of Th17 cells	1.88E-02			2	DLX3, IL9
Cell Death and Survival	Apoptosis of adenocarcinoma cell lines	1.89E-02		1.969	4	ALOX15, FOXO3, GATA6, MET
Cancer, Hematological Disease, Immunological Disease, Organismal Injury and Abnormalities	Lymphoid hyperplasia	1.89E-02		-0.152	4	CDKN2C, FOXO3, IL2RA, MERTK
Organ Morphology	Quantity of secretory structure	1.92E-02		0.78	9	AQP5, CACNA1E, CDKN2C, CRH, CSF1, GSX1, MET, PTF1A, SMPD3
Cell Signaling, Nucleic Acid Metabolism	Activation of Adenylate cyclase	1.93E-02			7	ACR, CXCL9, GHRHR, GPER1, PTGDR2, RGS1, RXFP2

Lipid Metabolism, Molecular Transport, Small Molecule Biochemistry	Quantity of steroid	1.95E-02		1.087	21	APOB, APOC1, CDKN2C, CGB3 (includes others), CRH, CSF1, FFAR4, FOXO3, GATA6, GATM, GHRHR, GPER1, LGR5, NAMPT, PMCH, PMP22, PRLR, SCIMP, SERPINA6, SRGAP3, VLDLR
Developmental Disorder, Hereditary Disorder, Metabolic Disease, Organismal Injury and Abnormalities	Argininosuccinate lyase deficiency	1.96E-02			1	ASL
Cancer, Developmental Disorder, Endocrine System Disorders, Organismal Injury and Abnormalities, Reproductive System Disease	Adult granulosa cell tumor of the ovary	1.96E-02			1	FOXL2
Cell Morphology	Cellularity of peripheral blood	1.96E-02			1	CSF1
Cell Cycle, Digestive System Development and Function	Arrest in cell cycle progression of enterocytes	1.96E-02			1	ID2
Cell Morphology	Conversion of osteoblasts	1.96E-02			1	MET
Hereditary Disorder, Organismal Injury and Abnormalities, Skeletal and Muscular Disorders	Cerivastatin-induced rhabdomyolysis	1.96E-02			1	CYP2C8
Cell Cycle, Hepatic System Development and Function	Arrest in early/mid Gap 2 phase of hepatocytes	1.96E-02			1	MET
Dermatological Diseases and Conditions, Hereditary Disorder, Inflammatory Disease, Inflammatory Response, Organismal Injury and Abnormalities	Autosomal recessive Netherton syndrome	1.96E-02			1	SPINK5
Cardiovascular Disease, Cellular Development, Cellular Growth and Proliferation,	Development of plaque cells	1.96E-02			1	PRKG1

Organismal Injury and Abnormalities						
Cardiovascular Disease, Hereditary Disorder, Organismal Injury and Abnormalities	Familial thoracic aortic aneurysm type 8	1.96E-02			1	PRKG1
Cell Cycle, Hepatic System Development and Function	Entry into S phase of hepatocytes	1.96E-02			1	MET
Cardiovascular Disease, Developmental Disorder, Organismal Injury and Abnormalities	Atrioventricular septal defect type 5	1.96E-02			1	GATA6
Connective Tissue Disorders, Dermatological Diseases and Conditions, Developmental Disorder, Hereditary Disorder, Organismal Injury and Abnormalities	Dominant neonatal dystrophic epidermolysis bullosa	1.96E-02			1	COL7A1
Cell-To-Cell Signaling and Interaction	Association of peritoneal macrophages	1.96E-02			1	APOC1
Developmental Disorder, Hematological Disease, Hereditary Disorder, Metabolic Disease, Nutritional Disease, Organismal Injury and Abnormalities	Digenic juvenile hemochromatosis	1.96E-02			1	HFE
Metabolic Disease, Organismal Injury and Abnormalities, Renal and Urological Disease	Experimental proteinuric renal disease	1.96E-02			1	CFHR1
Cell-To-Cell Signaling and Interaction, Inflammatory Response	Anti-inflammatory response of peritoneal macrophages	1.96E-02			1	FFAR4
Developmental Disorder, Endocrine System Disorders,	Central precocious puberty type 2	1.96E-02			1	MKRN3

Organismal Injury and Abnormalities, Reproductive System Disease						
Nucleic Acid Metabolism, Small Molecule Biochemistry	Elevation of cyclic GMP	1.96E-02			1	CAT
Amino Acid Metabolism, Post-Translational Modification, Small Molecule Biochemistry	Conversion of glycine	1.96E-02			1	GATM
Gastrointestinal Disease, Immunological Disease, Organismal Injury and Abnormalities	Celiac disease 3	1.96E-02			1	CTLA4
Connective Tissue Disorders, Dermatological Diseases and Conditions, Developmental Disorder, Hereditary Disorder, Immunological Disease, Organismal Injury and Abnormalities	Dominant dystrophic epidermolysis bullosa with absence of skin	1.96E-02			1	COL7A1
Cellular Movement, Hematological System Development and Function	Extravasation of dendritic precursor cells	1.96E-02			1	CSF1
Cell Cycle	Exit from G1 phase of naive T lymphocytes	1.96E-02			1	CTLA4
Cell Death and Survival, Cellular Function and Maintenance	Colony survival of endometrial cancer cell lines	1.96E-02			1	MSH2
Nervous System Development and Function, Organismal Development, Skeletal and Muscular System Development and Function	Coordination of forelimb	1.96E-02			1	NAMPT

Cell-mediated Immune Response, Cellular Development, Cellular Function and Maintenance, Cellular Growth and Proliferation, Connective Tissue Development and Function, Embryonic Development, Hematological System Development and Function, Hematopoiesis, Lymphoid Tissue Structure and Development, Organ Development, Organismal Development, Tissue Development	Development of lamina propria T lymphocytes	1.96E-02			1	ID2
Cellular Development, Embryonic Development, Nervous System Development and Function, Organismal Development, Tissue Development	Differentiation of motor neuron progenitor cells	1.96E-02			1	OLIG2
Organ Development, Visual System Development and Function	Growth of photoreceptor layer	1.96E-02			1	MERTK
Cell Cycle	Exit from quiescence of hepatocytes	1.96E-02			1	MET
Neurological Disease, Organ Morphology, Organismal Injury and Abnormalities, Psychological Disorders	Atrophy of stratum pyramidale	1.96E-02			1	NAMPT
Dermatological Diseases and Conditions,	Blepharophimosis , ptosis, and epicanthus	1.96E-02			1	FOXL2

Developmental Disorder, Hereditary Disorder, Neurological Disease, Ophthalmic Disease, Organismal Injury and Abnormalities	inversus type II with Duane retraction syndrome					
Auditory Disease, Hereditary Disorder, Neurological Disease, Organismal Injury and Abnormalities, Skeletal and Muscular Disorders	Charcot-Marie-Tooth disease type 1e	1.96E-02			1	PMP22
Cardiovascular Disease, Cell Death and Survival, Connective Tissue Disorders, Developmental Disorder, Hematological Disease, Hereditary Disorder, Metabolic Disease, Organismal Injury and Abnormalities	Autosomal recessive pyruvate kinase protein deficiency anemia	1.96E-02			1	PKLR
Neurological Disease, Organismal Injury and Abnormalities, Reproductive System Disease	Functional hypothalamic amenorrhea	1.96E-02			1	SERPINA6
Connective Tissue Disorders, Dermatological Diseases and Conditions, Developmental Disorder, Hereditary Disorder, Metabolic Disease, Organismal Injury and Abnormalities, Skeletal and Muscular Disorders	Autosomal recessive cutis laxa type 2A	1.96E-02			1	ATP6V0A2
Organismal Injury and Abnormalities, Respiratory Disease	Hyperreactivity of bronchia	1.96E-02			1	CCR3

Cell Cycle, Cellular Movement	Cytokinesis of melanoma cell lines	1.96E-02			1	CASC1
Connective Tissue Disorders, Developmental Disorder, Hereditary Disorder, Organismal Injury and Abnormalities, Skeletal and Muscular Disorders	Autosomal recessive primary microcephaly type 13	1.96E-02			1	CENPE
Cell-To-Cell Signaling and Interaction, Inflammatory Response	Cytotoxic T lymphocyte response by monocyte-derived dendritic cells	1.96E-02			1	MUC1
Cardiovascular Disease, Developmental Disorder, Hereditary Disorder, Organismal Injury and Abnormalities	Atrial septal defect type 9	1.96E-02			1	GATA6
Cell Morphology, Cell-mediated Immune Response, Cellular Development, Cellular Function and Maintenance, Cellular Growth and Proliferation, Embryonic Development, Hematological System Development and Function, Hematopoiesis, Lymphoid Tissue Structure and Development, Organ Development, Organismal Development, Tissue Development	Conversion of natural T-regulatory cells	1.96E-02			1	FOXP3
Nervous System Development and Function, Organismal Development,	Coordination of hindlimb	1.96E-02			1	NAMPT

Skeletal and Muscular System Development and Function						
Amino Acid Metabolism, Small Molecule Biochemistry	Binding of L-tryptophan	1.96E-02			1	IDO1
Cell Cycle	Arrest in G0/G1 phase transition of vascular endothelial cells	1.96E-02			1	MEOX2
Skeletal and Muscular System Development and Function, Tissue Development	Delay in initiation of mineralization of cartilage matrix	1.96E-02			1	SMPD3
Developmental Disorder, Hereditary Disorder, Metabolic Disease, Organismal Injury and Abnormalities, Renal and Urological Disease	Argininosuccinic aciduria	1.96E-02			1	ASL
Cardiovascular Disease, Developmental Disorder, Hereditary Disorder, Organismal Injury and Abnormalities	Autosomal dominant long QT syndrome 2	1.96E-02			1	KCNH2
Cellular Compromise, Connective Tissue Disorders, Organismal Injury and Abnormalities	Damage of red blood cells	1.96E-02			1	FOXO3
Cell-To-Cell Signaling and Interaction, Cellular Assembly and Organization	Cell-cell contact of osteoclasts	1.96E-02			1	GPR183
Gastrointestinal Disease, Immunological Disease, Organismal Injury and Abnormalities	Anaphylaxis of intestine	1.96E-02			1	IL9
Molecular Transport	Ejection of H+	1.96E-02			1	PMCH
Cellular Development,	Colony formation of low	1.96E-02			1	CCL23

Cellular Growth and Proliferation, Hematological System Development and Function, Hematopoiesis, Tissue Development	proliferative potential colony-forming cells					
Auditory Disease	Endolymphatic hydrops	1.96E-02			1	TECTA
Hereditary Disorder, Immunological Disease, Organismal Injury and Abnormalities	Deficiency of alpha interleukin 2 receptor	1.96E-02			1	IL2RA
Hereditary Disorder, Metabolic Disease, Organismal Injury and Abnormalities	Autosomal recessive hereditary folate malabsorption	1.96E-02			1	SLC46A1
Cell-To-Cell Signaling and Interaction, Skeletal and Muscular System Development and Function	Delamination of muscle precursor cells	1.96E-02			1	MET
Cancer, Cell Death and Survival, Organismal Injury and Abnormalities, Tumor Morphology	Cell viability of multiple myeloma cells	1.96E-02			1	BNIP3
Drug Metabolism, Small Molecule Biochemistry	Bioactivation of olmesartan medoxomil	1.96E-02			1	CMBL
Cell Morphology, Cellular Function and Maintenance	Autophagy of endoplasmic reticulum	1.96E-02			1	BNIP3
Hereditary Disorder, Immunological Disease, Neurological Disease, Organismal Injury and Abnormalities	Autosomal dominant hyperekplexia type 1	1.96E-02			1	GLRA1
Cancer, Cellular Development, Cellular Growth and Proliferation, Hematological Disease, Organismal Injury and	Arrest in growth of acute myeloid leukemia blast cells	1.96E-02			1	MUC1

Abnormalities, Tumor Morphology						
Cancer, Hematological Disease, Immunological Disease, Organismal Injury and Abnormalities	Formation of peripheral T-cell lymphoma	1.96E-02			1	CDKN2C
Cardiovascular Disease, Hereditary Disorder, Organismal Injury and Abnormalities, Skeletal and Muscular Disorders	Familial hypertrophic cardiomyopathy type 13	1.96E-02			1	TNNC1
Developmental Disorder, Hereditary Disorder, Organismal Injury and Abnormalities, Skeletal and Muscular Disorders	Autosomal dominant Emery-Dreifuss muscular dystrophy type 4	1.96E-02			1	SYNE1
Hematopoiesis	Frequency of high proliferative potential colony-forming cells	1.96E-02			1	SMAD5
Cancer, Organismal Injury and Abnormalities	Extracolonic cancer	1.96E-02			1	MSH2
Cardiovascular Disease, Organismal Injury and Abnormalities	Advanced chronic heart failure	1.96E-02			1	TNNC1
Cancer, Organismal Injury and Abnormalities	Delay in initiation of growth of melanoma	1.96E-02			1	CTLA4
Dermatological Diseases and Conditions, Developmental Disorder, Endocrine System Disorders, Hereditary Disorder, Ophthalmic Disease, Organismal Injury and Abnormalities, Reproductive System Disease	Autosomal dominant type 1 blepharophimosis , epicanthus inversus, and ptosis	1.96E-02			1	FOXL2
Cell Cycle	G1/S phase transition of	1.96E-02			1	CRH

	keratinocyte cancer cell lines					
Auditory Disease, Hereditary Disorder, Neurological Disease, Organismal Injury and Abnormalities	Autosomal recessive deafness type 97	1.96E-02			1	MET
Lipid Metabolism, Small Molecule Biochemistry	Association of oleic acid	1.96E-02			1	APOC1
Connective Tissue Disorders, Dermatological Diseases and Conditions, Developmental Disorder, Hereditary Disorder, Organismal Injury and Abnormalities	Autosomal recessive epidermolysis bullosa dystrophica inversa	1.96E-02			1	COL7A1
Cell-To-Cell Signaling and Interaction	Activation of luminal progenitor cells	1.96E-02			1	MET
Developmental Disorder, Hematological Disease, Hereditary Disorder, Immunological Disease, Organismal Injury and Abnormalities	Autoimmune lymphoproliferative syndrome type V	1.96E-02			1	CTLA4
Developmental Disorder, Hematological Disease, Hereditary Disorder, Metabolic Disease, Nutritional Disease, Organismal Injury and Abnormalities	Autosomal recessive hereditary hemochromatosis	1.96E-02			1	HFE
Dermatological Diseases and Conditions, Hereditary Disorder, Organismal Injury and Abnormalities	Bothnia type palmoplantar keratoderma	1.96E-02			1	AQP5
Lipid Metabolism, Small Molecule Biochemistry	Conjugation of stearoyl-coenzyme A	1.96E-02			1	GLYATL2

Cell Death and Survival, Hepatic System Development and Function	Cell viability of oval cells	1.96E-02			1	MET
Hereditary Disorder, Neurological Disease, Organismal Injury and Abnormalities, Skeletal and Muscular Disorders	Autosomal recessive spastic paraplegia type 28	1.96E-02			1	DDHD1
Cell Morphology, Cellular Function and Maintenance	Depolarization of intrecalated cells	1.96E-02			1	ATP6V0D2
Cancer, Cell-To-Cell Signaling and Interaction, Inflammatory Response	Cytotoxic reaction of endometrial cancer cell lines	1.96E-02			1	MSH2
Developmental Disorder, Organismal Development, Organismal Injury and Abnormalities	Abnormal morphology of inguinal canal	1.96E-02			1	RXFP2
Lipid Metabolism, Small Molecule Biochemistry	Conjugation of lauroyl-coenzyme A	1.96E-02			1	GLYATL2
Cellular Compromise	Disruption of intracellular membranes	1.96E-02			1	CSF1
Cellular Development, Connective Tissue Development and Function, Hematopoiesis, Tissue Development	Differentiation of colony forming unit fibroblasts	1.96E-02			1	CD38
Cell Morphology, Cellular Compromise	Collapse of centrosome	1.96E-02			1	NEK2
Cell Cycle, Cellular Movement	Cytokinesis of ovarian cancer cell lines	1.96E-02			1	GATA6
Neurological Disease, Organismal Injury and Abnormalities	Astrocytosis of dentate gyrus	1.96E-02			1	NAMPT
Dermatological Diseases and Conditions,	Autosomal dominant type 2 blepharophimosis	1.96E-02			1	FOXL2

Developmental Disorder, Hereditary Disorder, Ophthalmic Disease, Organismal Injury and Abnormalities	, epicanthus inversus, and ptosis					
Cancer, Organismal Injury and Abnormalities, Renal and Urological Disease	Bilateral papillary renal cell carcinoma	1.96E-02			1	MET
Embryonic Development, Organ Development, Organismal Development, Reproductive System Development and Function, Tissue Development	Growth of terminal end bud	1.96E-02			1	CSF1
Cell-To-Cell Signaling and Interaction, Drug Metabolism, Molecular Transport, Small Molecule Biochemistry	Delay in secretion of epinephrine	1.96E-02			1	CRH
Nervous System Development and Function	Flexor reflex of spinal cord	1.96E-02			1	PRKG1
Cellular Development, Cellular Growth and Proliferation, Embryonic Development, Organ Development, Organismal Development, Reproductive System Development and Function, Tissue Development	Formation of lobulo-alveolar bud cells	1.96E-02			1	PRLR
Hereditary Disorder, Organismal Injury and Abnormalities	Corticosteroid-binding globulin deficiency	1.96E-02			1	SERPINA6
Small Molecule Biochemistry	Demethylation of rosiglitazone	1.96E-02			1	CYP2C8

Auditory Disease, Hereditary Disorder, Neurological Disease, Organismal Injury and Abnormalities	Autosomal dominant deafness 12	1.96E-02			1	TECTA
Cell Morphology, Cellular Assembly and Organization, Cellular Development, Cellular Function and Maintenance, Cellular Growth and Proliferation, Embryonic Development, Nervous System Development and Function, Organismal Development, Tissue Development	Bifurcation of sensory axons	1.96E-02			1	PRKG1
Cell-To-Cell Signaling and Interaction, Hematological System Development and Function, Humoral Immune Response, Inflammatory Response	Antibody response of Ab-forming cells	1.96E-02			1	MERTK
Tissue Morphology	Density of pituitary cells	1.96E-02			1	FOXL2
Cancer, Gastrointestinal Disease, Hepatic System Disease, Organismal Injury and Abnormalities	Childhood type hepatocellular carcinoma	1.96E-02			1	MET
Connective Tissue Disorders, Developmental Disorder, Hereditary Disorder, Inflammatory Disease, Inflammatory Response, Metabolic Disease, Organismal Injury and Abnormalities,	Gout susceptibility 4	1.96E-02			1	SLC17A3

Skeletal and Muscular Disorders						
Cell Cycle, Hematological System Development and Function	Cell division of bone marrow-derived macrophages	1.96E-02			1	CSF1
Cardiovascular System Development and Function, Embryonic Development, Organ Development, Organismal Development, Tissue Development	Angiogenesis of cardiac valve	1.96E-02			1	CNMD
Cell Death and Survival	Cytolysis of lymphoblastoid cells	1.96E-02			1	MSH2
Lipid Metabolism, Small Molecule Biochemistry	Distribution of glucocorticoid	1.96E-02			1	SERPINA6
Lipid Metabolism, Small Molecule Biochemistry	Conjugation of palmitoyl-coenzyme A	1.96E-02			1	GLYATL2
Cell-To-Cell Signaling and Interaction, Hematological System Development and Function, Immune Cell Trafficking, Inflammatory Response	Activation of exudate macrophages	1.96E-02			1	IL1RL1
Cardiovascular System Development and Function, Embryonic Development, Organismal Development, Tissue Development	Development of aortic sac	1.96E-02			1	KCNH2
Cell Morphology, Cell-mediated Immune Response, Cellular Development, Cellular Function and Maintenance, Cellular Growth and Proliferation,	Conversion of peripheral T lymphocyte	1.96E-02			1	FOXP3

Embryonic Development, Hematological System Development and Function, Hematopoiesis, Lymphoid Tissue Structure and Development, Organ Development, Organismal Development, Tissue Development						
Cardiovascular Disease, Cardiovascular System Development and Function, Hereditary Disorder, Organ Morphology, Organismal Development, Organismal Injury and Abnormalities, Skeletal and Muscular Disorders	Dilated cardiomyopathy 1z	1.96E-02			1	TNNC1
Connective Tissue Disorders, Dermatological Diseases and Conditions, Developmental Disorder, Hereditary Disorder, Immunological Disease, Organismal Injury and Abnormalities	Autosomal recessive epidermolysis bullosa pruriginosa	1.96E-02			1	COL7A1
Cell-To-Cell Signaling and Interaction, Nervous System Development and Function	Firing of cortical neurons	1.96E-02			1	CSF1
Cell Morphology, Cellular Assembly and Organization, Cellular Function and Maintenance	Assembly of autophagosomes	1.96E-02			1	MET

Cardiovascular System Development and Function, Tissue Development	Function of vascular tissue	1.96E-02			1	PRKG1
Hematological System Development and Function, Immunological Disease, Lymphoid Tissue Structure and Development, Organ Morphology, Organismal Injury and Abnormalities, Tissue Morphology	Abnormal morphology of tonsil tissue	1.96E-02			1	ID2
Hereditary Disorder, Metabolic Disease, Organismal Injury and Abnormalities	Elevated adenosine triphosphate of erythrocytes	1.96E-02			1	PKLR
Gastrointestinal Disease, Humoral Immune Response, Inflammatory Disease, Inflammatory Response, Organismal Injury and Abnormalities	Experimentally induced inflammation of intestine	1.96E-02			1	CTLA4
Cellular Movement, Hematological System Development and Function, Humoral Immune Response, Immune Cell Trafficking, Inflammatory Response, Lymphoid Tissue Structure and Development	Chemotaxis of plasma cells	1.96E-02			1	CCL28
DNA Replication, Recombination, and Repair, Nucleic Acid Metabolism, Small Molecule Biochemistry	Chlorination of deoxycytidine	1.96E-02			1	CAT
Cell Cycle, Gene Expression	Binding of E2 box element	1.96E-02			1	ID2
Connective Tissue Development and	Density of osteoclasts	1.96E-02			1	CSF1

Function, Skeletal and Muscular System Development and Function, Tissue Morphology						
Cardiovascular Disease	Ectasia of vascular lesion	1.96E-02			1	CTLA4
Cellular Development, Cellular Growth and Proliferation, Embryonic Development, Hematological System Development and Function, Hematopoiesis, Humoral Immune Response, Lymphoid Tissue Structure and Development, Organ Development, Organismal Development, Tissue Development	Arrest in lymphopoiesis of pro-B lymphocytes	1.96E-02			1	IL2RA
Gastrointestinal Disease, Hepatic System Disease, Inflammatory Disease, Organismal Injury and Abnormalities	AMA-positive primary biliary cirrhosis	1.96E-02			1	CTLA4
Embryonic Development, Hair and Skin Development and Function, Organ Development, Organismal Development, Tissue Development	Formation of hair shaft	1.96E-02			1	mir-31
Cellular Development	Dedifferentiation of gonadal cell lines	1.96E-02			1	GATA6
Embryonic Development, Nervous System Development and Function, Organ	Formation of basis pontis	1.96E-02			1	NFIB

Development, Organismal Development, Tissue Development						
Cancer, Cellular Development, Organismal Injury and Abnormalities, Tumor Morphology	Differentiation of chronic lymphocytic leukemia cells	1.96E-02			1	CD38
Lipid Metabolism, Small Molecule Biochemistry	Conjugation of palmitoleoyl-coenzyme A	1.96E-02			1	GLYATL2
Cardiovascular Disease, Developmental Disorder, Hereditary Disorder, Metabolic Disease, Organismal Injury and Abnormalities, Renal and Urological Disease	Autosomal dominant pseudohypoaldosteronism type IID	1.96E-02			1	KLHL3
Cell Cycle, Hematopoiesis	Arrest in mitosis of erythroid cells	1.96E-02			1	FOXO3
Cell Cycle, Hepatic System Development and Function	Aneuploidy of hepatocytes	1.96E-02			1	CENPE
Carbohydrate Metabolism, Small Molecule Biochemistry	Accumulation of sorbitol	1.96E-02			1	SORD
Connective Tissue Disorders, Dermatological Diseases and Conditions, Developmental Disorder, Hereditary Disorder, Immunological Disease, Organismal Injury and Abnormalities	Autosomal recessive pretibial epidermolysis bullosa	1.96E-02			1	COL7A1
Cellular Assembly and Organization, DNA Replication, Recombination, and Repair	Assembly of heterochromatin	1.96E-02			1	ADARB1
Cell-To-Cell Signaling and Interaction, Nervous	Firing of nucleus accumbens shell	1.96E-02			1	PMCH

System Development and Function						
Hematological Disease, Immunological Disease, Organismal Injury and Abnormalities, Respiratory Disease	Eosinophilia of nasal tissue	1.96E-02			1	PTGDR2
Digestive System Development and Function	Eruption of molar tooth	1.96E-02			1	CSF1
Cell Cycle	Entry into G2/M phase of colorectal cancer cell lines	1.96E-02			1	WEE1
Hereditary Disorder, Neurological Disease, Organismal Injury and Abnormalities	Autosomal dominant hereditary neuropathy with liability to pressure palsies	1.96E-02			1	PMP22
Hematological System Development and Function, Immune Cell Trafficking, Inflammatory Response, Tissue Development	Accumulation of memory precursor T lymphocytes	1.96E-02			1	FOXO3
Cell Cycle	Arrest in early G1 phase of macrophages	1.96E-02			1	CSF1
Cell Cycle	Arrest in G1/S phase transition of skin cell lines	1.96E-02			1	DLX3
Cardiovascular Disease, Developmental Disorder, Hereditary Disorder, Metabolic Disease, Organismal Injury and Abnormalities, Renal and Urological Disease	Autosomal recessive pseudohypoaldosteronism type IID	1.96E-02			1	KLHL3
Cardiovascular Disease, Connective Tissue Disorders, Developmental Disorder,	Cleft palate, cardiac defects and mental retardation	1.96E-02			1	MEIS2

Gastrointestinal Disease, Hereditary Disorder, Neurological Disease, Organismal Development, Organismal Injury and Abnormalities, Skeletal and Muscular Disorders						
Cancer, Organismal Injury and Abnormalities	Dysplasia of tumor	1.96E-02			1	ID2
Hereditary Disorder, Organismal Injury and Abnormalities, Renal and Urological Disease	Focal segmental glomerulosclerosis type 2	1.96E-02			1	TRPC6
Hereditary Disorder, Neurological Disease, Organismal Injury and Abnormalities, Skeletal and Muscular Disorders	Autosomal recessive Charcot-Marie-Tooth disease type 1A	1.96E-02			1	PMP22
Developmental Disorder, Hereditary Disorder, Organismal Functions, Organismal Injury and Abnormalities, Skeletal and Muscular Disorders	Familial limb-girdle muscular dystrophy type 2L	1.96E-02			1	ANO5
Cell Death and Survival	Apoptosis of germinal center	1.96E-02			1	MSH2
Dermatological Diseases and Conditions, Developmental Disorder, Endocrine System Disorders, Hereditary Disorder, Ophthalmic Disease, Organismal Injury and Abnormalities, Reproductive System Disease	Autosomal recessive blepharophimosis , ptosis, and epicanthus inversus type 1	1.96E-02			1	FOXL2
Cellular Compromise	Disappearance of centrosome	1.96E-02			1	NEK2
Cellular Development, Cellular Growth and	Development of commissural neurons	1.96E-02			1	BARHL2

Proliferation, Embryonic Development, Nervous System Development and Function, Organ Development, Organismal Development, Tissue Development						
Cellular Compromise	Depletion of bone marrow cells	1.96E-02			1	CSF1
Cell-To-Cell Signaling and Interaction, Cellular Assembly and Organization, Skeletal and Muscular System Development and Function	Cell-cell adhesion of vascular smooth muscle cells	1.96E-02			1	ALOX15
Cell-To-Cell Signaling and Interaction, Cellular Growth and Proliferation	Co-stimulation of leukemia cell lines	1.96E-02			1	IL9
Lipid Metabolism, Small Molecule Biochemistry	Conjugation of myristoyl-coenzyme A	1.96E-02			1	GLYATL2
Developmental Disorder, Hereditary Disorder, Metabolic Disease, Neurological Disease, Organismal Injury and Abnormalities, Psychological Disorders	Arginine:glycine amidinotransferase deficiency	1.96E-02			1	GATM
Developmental Disorder, Hereditary Disorder, Organismal Injury and Abnormalities	3p- syndrome	1.96E-02			1	SRGAP3
Cell-mediated Immune Response, Hematological System Development and Function	Anergy of regulatory T lymphocytes	1.96E-02			1	CTLA4
Hematological Disease, Immunological	Chronic eosinophilic rhinosinusitis	1.96E-02			1	IL5RA

Disease, Inflammatory Disease, Inflammatory Response, Organismal Injury and Abnormalities, Respiratory Disease						
Nervous System Development and Function	Guidance of osteoclast precursor cells	1.96E-02			1	GPR183
Developmental Disorder, Embryonic Development, Organismal Development, Tissue Morphology	Abnormal morphology of dental follicle	1.96E-02			1	CSF1
Cellular Development, Nervous System Development and Function, Tissue Development, Visual System Development and Function	Arrest in differentiation of amacrine cells	1.96E-02			1	PTF1A
Behavior	Goal-directed behavior	1.96E-02			1	SLC29A1
Digestive System Development and Function	Electrical resistance of jejunum	1.96E-02			1	TFF3
Cell-To-Cell Signaling and Interaction, Cellular Movement, Hematological System Development and Function, Immune Cell Trafficking, Nervous System Development and Function	Delay in recruitment of microglia	1.96E-02			1	CSF1
Cell-To-Cell Signaling and Interaction, Cellular Assembly and Organization	Cell-cell contact of osteoclast precursor cells	1.96E-02			1	GPR183
Hereditary Disorder, Organismal Injury and Abnormalities, Skeletal and Muscular Disorders	Gnathodiaphyseal dysplasia	1.96E-02			1	ANO5

Connective Tissue Disorders, Developmental Disorder, Hereditary Disorder, Neurological Disease, Organismal Injury and Abnormalities, Skeletal and Muscular Disorders	Acquired macrocephaly with impaired intellectual development	1.96E-02			1	NFIB
Cell Signaling, Vitamin and Mineral Metabolism	Delay in initiation of decay of Ca2+	1.96E-02			1	TNNC1
Cellular Assembly and Organization	Dispersal of centrosome	1.96E-02			1	NEK2
Cellular Development, Cellular Growth and Proliferation, Tissue Development	Development of cuboidal cells	1.96E-02			1	FOXL2
Cellular Development	Differentiation of heart cell lines	1.96E-02			1	EPC1
Cell-To-Cell Signaling and Interaction, Hematological System Development and Function, Immune Cell Trafficking, Inflammatory Response	Hyperactivation of helper T lymphocytes	1.96E-02			1	FOXO3
Cell Death and Survival	Apoptosis of bone-marrow-derived monocyte/macrophage precursor cells	1.96E-02			1	CSF1
Endocrine System Disorders, Organismal Injury and Abnormalities, Reproductive System Disease	Hyperprogesteronemia	1.96E-02			1	CGB3 (includes others)
Cellular Compromise	Breakdown of intracellular membranes	1.96E-02			1	ALOX15
Cell Cycle, Hematological System	Entry into S phase of naive T lymphocytes	1.96E-02			1	CTLA4

Development and Function						
Developmental Disorder, Hereditary Disorder, Metabolic Disease, Organismal Injury and Abnormalities	Acatalasemia	1.96E-02			1	CAT
Connective Tissue Disorders, Dermatological Diseases and Conditions, Developmental Disorder, Hereditary Disorder, Organismal Injury and Abnormalities	Generalized dominant dystrophic epidermolysis bullosa	1.96E-02			1	COL7A1
Cell-To-Cell Signaling and Interaction, Inflammatory Response	Anti-inflammatory response of macrophage cancer cell lines	1.96E-02			1	FFAR4
Hematological System Development and Function, Immune Cell Trafficking, Inflammatory Response, Tissue Development	Accumulation of bone marrow-derived macrophages	1.96E-02			1	CSF1
Hematological Disease, Hereditary Disorder, Organismal Injury and Abnormalities	Factor VII Marburg I variant thrombophilia	1.96E-02			1	HABP2
Cellular Movement, Nervous System Development and Function	Guidance of sensory axons	1.96E-02			1	PRKG1
Embryonic Development, Organismal Development, Tissue Development	Development of cardiac loop	1.96E-02			1	KCNH2
Cellular Compromise	Deformation of spindle pole	1.96E-02			1	CENPE
Cancer, Cellular Development, Organismal Injury and Abnormalities, Tumor Morphology	Differentiation of breast cancer epithelial cells	1.96E-02			1	ID2

Developmental Disorder, Hereditary Disorder, Neurological Disease, Organismal Injury and Abnormalities	Cerebellar ataxia and mental retardation with quadrupedal locomotion 1	1.96E-02			1	VLDLR
Dental Disease, Developmental Disorder, Gastrointestinal Disease, Hereditary Disorder, Organismal Injury and Abnormalities	Autosomal dominant amelogenesis imperfecta 4	1.96E-02			1	DLX3
Hematological Disease, Hereditary Disorder, Immunological Disease, Metabolic Disease, Organismal Injury and Abnormalities	Agammaglobulinemia type 2	1.96E-02			1	IGLL1/IGLL5
Cardiovascular Disease, Connective Tissue Disorders, Developmental Disorder, Neurological Disease, Organismal Injury and Abnormalities, Skeletal and Muscular Disorders	C syndrome	1.96E-02			1	CD96
Cellular Development, Cellular Growth and Proliferation, Embryonic Development, Nervous System Development and Function, Organ Development, Organismal Development, Tissue Development	Generation of Purkinje cells	1.96E-02			1	PTF1A
Cell-To-Cell Signaling and Interaction, Embryonic Development,	Cytotoxic reaction of embryonic stem cell lines	1.96E-02			1	MSH2

Inflammatory Response						
Small Molecule Biochemistry	Damage of heme	1.96E-02			1	CAT
Cell-To-Cell Signaling and Interaction	Activation of ear cells	1.96E-02			1	PRKG1
Connective Tissue Disorders, Dermatological Diseases and Conditions, Developmental Disorder, Hereditary Disorder, Metabolic Disease, Organismal Injury and Abnormalities, Skeletal and Muscular Disorders	Autosomal recessive cutis laxa type IID	1.96E-02			1	ATP6V1A
Hereditary Disorder, Neurological Disease, Organismal Injury and Abnormalities, Skeletal and Muscular Disorders	Autosomal dominant Charcot-Marie-Tooth disease type 1A	1.96E-02			1	PMP22
Auditory Disease, Hereditary Disorder, Neurological Disease, Organismal Injury and Abnormalities	Autosomal recessive deafness type 21	1.96E-02			1	TECTA
Carbohydrate Metabolism, Small Molecule Biochemistry	Catabolism of sorbitol	1.96E-02			1	SORD
Cancer, Endocrine System Disorders, Organismal Injury and Abnormalities, Reproductive System Disease	Grade 2 ovarian carcinoma	1.96E-02			1	PRLR
Cancer, Cell-To-Cell Signaling and Interaction, Inflammatory Response	Cytotoxic reaction of gastrointestinal stromal tumor cell lines	1.96E-02			1	MET
Cardiovascular Disease, Cell Death and Survival, Connective Tissue	Amish type pyruvate kinase deficiency	1.96E-02			1	PKLR

Disorders, Developmental Disorder, Hematological Disease, Hereditary Disorder, Metabolic Disease, Organismal Injury and Abnormalities						
Immunological Disease, Inflammatory Response	Early phase nasal response	1.96E-02			1	HRH4
Cellular Development	Dedifferentiation of ovarian cancer cell lines	1.96E-02			1	GATA6
Connective Tissue Disorders, Developmental Disorder, Hematological Disease, Hereditary Disorder, Neurological Disease, Organismal Injury and Abnormalities, Psychological Disorders	Choreaacanthocytosis	1.96E-02			1	VPS13A
Embryonic Development, Organ Development, Organismal Development, Reproductive System Development and Function, Tissue Development	Development of mammary gland tissue	1.96E-02			1	PRLR
Endocrine System Disorders, Organismal Injury and Abnormalities, Reproductive System Disease	Atresia of oocytes	1.96E-02			1	FOXL2
Developmental Disorder, Hereditary Disorder, Organismal Injury and Abnormalities	Autosomal dominant tricho-dento-osseous syndrome	1.96E-02			1	DLX3

Cell Morphology	Contractility of lung cancer cell lines	1.96E-02			1	CD24
Cellular Development, Cellular Growth and Proliferation, Nervous System Development and Function, Tissue Development	Development of V3 interneurons	1.96E-02			1	OLIG2
Small Molecule Biochemistry	Hydroxylation of rosiglitazone	1.96E-02			1	CYP2C8
Cell-To-Cell Signaling and Interaction, Nervous System Development and Function	Activation of projection neurons	1.96E-02			1	CRH
Cellular Development, Embryonic Development, Tissue Development	Differentiation of umbilical cord	1.96E-02			1	CGB3 (includes others)
Connective Tissue Disorders, Dermatological Diseases and Conditions, Developmental Disorder, Hereditary Disorder, Immunological Disease, Organismal Injury and Abnormalities	Autosomal dominant epidermolysis bullosa pruriginosa	1.96E-02			1	COL7A1
Embryonic Development, Organismal Development, Tissue Development	Development of bulbus cordis	1.96E-02			1	KCNH2
Organ Development, Reproductive System Development and Function	Function of oviduct	1.96E-02			1	PRLR
Cellular Function and Maintenance	Cell saturation density of colorectal cancer cell lines	1.96E-02			1	CD24

Cell-To-Cell Signaling and Interaction, Hematological System Development and Function, Hematopoiesis, Immune Cell Trafficking, Inflammatory Response	Activation of pro-T lymphocytes	1.96E-02			1	CD2
Auditory Disease, Hereditary Disorder, Neurological Disease, Organismal Injury and Abnormalities	Autosomal recessive deafness type 103	1.96E-02			1	CLIC5

Supplementary table 5: This table summarises the 35 transcripts which were commonly and significantly correlated with both Treg number and IL2RA expression at D64. In fact, a preliminary predictive biomarker screening was conducted by correlating all transcripts included in the autoimmune discovery NanoString panel with the aforementioned variables measured at D64. In particular, IL2RA expression was also used to reinforce our screening as it is a marker of Tregs and our microarray and qRT-PCR data suggested a time-dependent increase in this transcript over the treatment period which peaked at D64. Fifty-five transcripts showed a significant correlation with the Treg count at D64 while 72 were significantly correlated with IL2RA expression at D64. We then ranked these transcripts by their p-value (rank 1= transcript showing the lowest p-value). Subsequently, a consensus screening was conducted and 35 transcripts were found to be commonly significantly correlated with the two chosen variables (Treg and IL2RA expression) measured at D64. Lastly, a combined score was computed by adding the two ranks computed for the Treg and IL2RA correlations. Low scores reflected transcripts behind most significantly correlated with both the variables.
In this table transcripts names (IDs) are reported and ordered by their combined scores. Moreover, R2, p-value and rank is reported for each correlation analysis with either Treg count or IL2RA expression at D64. The top five transcripts showing the best combined scores are *BTLA*, *SBNO2*, *TRAF2*, *CD27* and *BLNK* which were selected as proposed biomarkers. Importantly, *TLR9* was also included as it is the transcript showing the best correlation with IL2RA at D64 (rank 1). Therefore, these 6 transcripts were used for qRT-PCR screening (highlighted in the table in yellow).

	**Correlation with Treg count at D64**			**Correlation with IL2RA expression at D64**			
	**R^2**	**Pval**	**Rank**	**R2**	**Pval**	**Rank**	**Combined_score**
**BTLA**	0.829	0.002	1	0.808	0.002	2	3
**SBNO2**	0.736	0.006	5	0.762	0.005	5	10
**TRAF2**	0.691	0.010	9	0.785	0.003	3	12
**CD27**	0.794	0.003	2	0.656	0.015	13	15
**BLNK**	0.705	0.009	6	0.651	0.015	15	21
**AFF3**	0.644	0.017	14	0.676	0.012	10	24
**SOX8**	0.641	0.017	15	0.665	0.014	11	26
**RASIP1**	0.783	0.003	3	0.607	0.023	25	28
**LCK**	0.646	0.016	12	0.633	0.018	17	29
**MPV17L2**	0.704	0.009	7	0.619	0.021	22	29
**RABEP2**	0.559	0.033	27	0.783	0.004	4	31
**TLR9**	0.554	0.034	31	0.821	0.002	1	32
**DLD**	0.580	0.028	21	0.656	0.015	12	33
**GMPPB**	0.591	0.026	19	0.655	0.015	14	33
**FKBP5**	0.556	0.034	29	0.728	0.007	6	35
**ZC2HC1A**	0.675	0.012	10	0.586	0.027	32	42
**UCN**	0.338	0.131	17	0.605	0.023	27	44
**PHRF1**	0.503	0.049	49	0.716	0.008	7	56
**TNFSF8**	0.547	0.036	33	0.611	0.022	23	56
**MYC**	0.569	0.031	25	0.581	0.028	33	58
**SPHK2**	0.742	0.006	4	0.541	0.037	54	58
**ADA**	0.590	0.026	20	0.568	0.031	41	61
**FADS3**	0.502	0.049	50	0.625	0.020	21	71
**TRAF3IP2**	0.543	0.037	35	0.571	0.030	38	73
**TNFAIP6**	0.523	0.043	41	0.576	0.029	36	77
**SPRY4**	0.569	0.031	26	0.542	0.037	53	79
**UBASH3A**	0.510	0.047	44	0.577	0.029	35	79

**IL6ST**	0.698	0.010	8	0.501	0.049	72	80
**CD48**	0.645	0.016	13	0.511	0.046	68	81
**TMBIM1**	0.524	0.042	40	0.559	0.033	45	85
**RUNX1**	0.537	0.039	37	0.551	0.035	50	87
**PROCR**	0.545	0.036	34	0.536	0.039	61	95
**CXCL9**	0.555	0.034	30	0.520	0.043	66	96
**WDFY4**	0.513	0.046	42	0.541	0.038	56	98
**IRF8**	0.524	0.042	38	0.521	0.043	65	103

Supplementary table 6: Table summarising correlation scores for the six transcripts investigated by qRT-PCR (*BTLA*, *SBNO2*, *TRAF2*, *CD27*, *BLNK* and *TLR9*). A significant correlation was found only for TLR9 although, when visually inspected CD27 showed a promising pattern (see Figure 6).

ID	R	R^2	P-value
**TLR9**	-0.809	0.654	0.0014
**CD27**	0.416	0.173	0.17
**SBNO2**	0.273	0.074	0.38
**BLNK**	-0.209	0.043	0.51
**BTLA**	0.039	0.0016	0.9
**TRAF2**	0.023	0.0005	0.9
